# A study on the tourism efficiency of tourism destination based on DEA model: A case of ten cities in Shaanxi province

**DOI:** 10.1371/journal.pone.0296660

**Published:** 2024-01-19

**Authors:** Yajun Guo, Zhuo Cao

**Affiliations:** 1 School of Economics and Management, Northwest University, Xi’an, Shaanxi, China; 2 Teaching and Research Department of Political Economy, Party School of Shaanxi Provincial Party Committee of the Communist Party of China, Xi’an, Shaanxi, China; Sichuan University, CHINA

## Abstract

Exploring the of regional tourism efficiency is of great significance in promoting high-quality development of regional tourism. However, there are not many studies that measure the quality development of tourism destinations from the perspective of inputs and output. Based on this, the data envelopment analysis model is used to measure the overall technical efficiency (TE_CRS_), pure technical efficiency (TE_VRS_), and scale efficiency (SE) with the help of DEA-SOLVER software, taking the ten prefecture-level cities in Shaanxi Province as examples, to further analyze and evaluate the spatial differences of different tourism destinations and the reasons for the differences. The results of the study found that: the efficiency indicators explain the differences in the development quality of tourism destinations from different sides; the development quality of tourism destinations in Shaanxi as a whole is low, with excessive inputs and insufficient outputs; and the tourism destinations with relatively high development quality are distributed in the Guanzhong. On this basis, corresponding countermeasure suggestions are put forward to promote the improvement of governance efficiency of tourism destinations in Shaanxi Province, and then optimize the quality of development.

## Introduction

The expression "high-quality development" was first put forward at the the 19th National Congress of the Communist Party of China, indicating that China’s economy has shifted from the stage of high-speed growth to that of high-quality development. As a strategic pillar industry of the national economy and an important dimension in promoting economic and social development, the tourism industry also needs to achieve high-quality development to meet the development needs of industrial transformation and upgrading and modernization of the national governance system. In particular, since the global COVID-19 epidemic, the tourism industry has faced severe tests of its development model and governance capacity, resulting in the necessity and importance of high-quality development becoming more and more prominent. The 14th Five-Year Plan for Tourism Development also put forward the theme of promoting high-quality development of the tourism industry, further reflecting the inadequacy of the current level of development and the urgency of seeking improvement. He [1] defined the high-quality development of tourism industry as a system, which is a synthesis of the interaction of various elements in tourism activities.

Tourism destination is a geographical space formed by integrating various elements, which can trigger tourists’ travel motivation and provide effective products and services [2]. Tourism destination is the most important carrier of tourism activities, the most basic unit for analyzing tourism industry, and the most critical link that distinguishes tourism research from other research. A review of the domestic and foreign research literature reveals that the development quality of tourism destinations can be broadly divided into two categories: theoretical studies and empirical studies. Some scholars focus on conceptualized theoretical research, taking domestic policies and hotspot issues prevalent in major tourism destinations as the research background to explore new models of high-quality tourism destination development, or starting from the overall perspective, critically examining traditional tourism destination development models [3] and tourism destination image promotion models [4,5], or starting from a subdivision of tourism destination types, based on sustainable development theory to consider the development paths of high-quality tourism destinations (eco-tourism destinations [6], rural tourism destinations [7], etc.). Other scholars focus on empirical research, using a combination of qualitative and quantitative methods to conduct in-depth analysis of specific tourist destinations [8], which helps to understand the level of tourism development of the destination in a timely manner, rationally allocate the input-output ratio of tourism resources, promote the destination to formulate appropriate tourism development strategies, explore the path of industrial efficiency enhancement, and at the same time, provide reference for the enhancement of the quality of the tourism industry, either based on the consumer perspective to help tourism destinations develop in a high-quality development from the perspective of tourists [9], or based on social carrying capacity theory and social exchange theory to assess the development quality of tourism destinations from the perspective of the host-guest relationship and make suggestions for high-quality development [10–13], or using various data envelopment analysis (DEA) models to assess tourism efficiency by selecting measurement indicators from multiple perspectives [14,15]. According to the literature search, domestic and foreign studies mainly use tourism efficiency to represent the high-quality development level of regional tourism, which may also be understood as competitiveness, attraction, sustainable development ability, and high-quality service. Foreign tourism efficiency-related research started earlier and accumulated a large number of research results, mainly focusing on the hotel industry, travel agencies and other traditional industrial elements of operational efficiency and management efficiency, such as Morey et al. [16] used DEA to measure and analyze the operational efficiency of 54 hotels in the United States, and found that the hotel operational efficiency is higher; Barros et al. [17] found that the operating time, capital, labor, and sales volume are the determinants of the efficiency of Portuguese travel agencies. Cvelbar et al. [18] selected 139 tourism destinations as research objects, examined the driving factors of tourism destination competitiveness from a global perspective, equated tourism competitiveness with tourism efficiency, and reflected the development level of tourism quality. However, although domestic tourism efficiency-related studies started late, results with reference value emerged from different levels (e.g., national level, regional level, city cluster level, etc.). Deng and Lu [19] calculated the tourism efficiency of 17 cities in the Anhui Province of China, to assess the level of high-quality development of tourism destinations from the provincial level. Tourism efficiency is one of the important issues in tourism scientific research, and it is an important yardstick to measure the ability of tourism economic subjects to utilize resources and maximize the total surplus for all stakeholders. To sum up, although the high-quality development of tourism and tourism efficiency are not the same, tourism efficiency has become a recognized indicator to reflect and evaluate the high-quality development level of tourism destinations. Many researchers explore tourism efficiency from the perspective of economics, tourism and management geography, mainly using DEA to measure overall technical efficiency (TE_CRS_), pure technical efficiency (TE_VRS_), and scale efficiency (SE). Chau and Walker [20] pointed out that TE_CRS_, TE_VRS_, and SE are important factors in measuring the development potential and competitiveness of specific production units.

In general, there are fewer empirical studies than theoretical studies on the quality of tourism destination development. At present, domestic and foreign studies on the efficiency of urban tourism (tourism destination) using the DEA model have rarely been reported. There is even a lack of judgment and analysis of resource utilization in the production process of urban tourism in provincial areas and a lack of explanation based on provincial characteristics. Therefore, this paper applies the DEA model to the empirical evaluation of high-quality development of tourism destinations. This study collected relevant data from all prefecture-level cities (ten in total, namely Xi’an, Tongchuan, Baoji, Xianyang, Weinan, Yan’an, Hanzhong, Yulin, Ankang, Guangzhong) in Shaanxi Province in 2018 (High-quality development related expression was first proposed at the 19th National Congress of the Communist Party of China in 2017. Meanwhile, due to the global spread of the COVID-19 epidemic, the tourism industry has been hit hard after 2019, and the demand side of tourism has been seriously affected. To avoid the impact of the new crown epidemic on the tourism development agent and the resulting cliff fall and lack of relevant statistics, the data year is set at 2018) to measure the tourism efficiency of prefecture-level cities in the province with TE_CRS_, TE_VRS_, and SE, and to evaluate the level of high-quality development. Secondly, it analyzes and evaluates the spatial differences of different tourism destinations and the reasons for the differences according to the DEA effectiveness. Finally, based on the results of the study, it provides scientific basis for the city to formulate relevant tourism countermeasures.

## Overview of the study area

Shaanxi, abbreviated as “Shan” or “Qin”, is one of the province-level administrative units of the People’s Republic of China, with the ancient capital Xi’an as its provincial capital. Geographically, it is situated between east longitude 105°29’ to 111°15’ and north latitude 31°42’ to 39°35’, straddling the divide between the northern and southern regions due to the presence of the Qinling Mountains-Huaihe River line. It lies in the inland hinterland of Northwest China, spanning the central regions of both the Yellow River and Yangtze River basins, serving as a crucial nexus connecting the eastern and central parts of China with the northwest and southwest regions. Shaanxi province has gained increasing attention from researchers and policymakers due to its rich cultural heritage, historical significance, and burgeoning tourism industry. Shaanxi, as an inland province that developed tourism earlier after the reform and opening up, used to be a top ten provincial tourism destination in terms of domestic tourist reception. As of February 2021, there are 502 scenic spots of various types in Shaanxi Province, including 11 5A-level scenic spots, 131 4A-level scenic spots, and 318 3A-level scenic spots [21]. Shaanxi Province is mainly divided into three regions: Guanzhong, southern Shaanxi and Northern Shaanxi. Xi’an is not only a regional center city, but also the first ranked tourist city in the province. Xi ’an as the center and its adjacent area (Guanzhong area) is one of the main birthplaces of Chinese civilization, the long history has left a profound historical and cultural heritage to the area. Xi ’an and its adjacent areas gather a large number of high-grade tourist attractions, accounting for 90 percent of the total number of high-level tourist attractions in Shaanxi. Hanzhong is located in the southern part of southern Shaanxi Province, near the Qinling Mountains in the north and the Bashan Mountains in the south. It has a subtropical monsoon climate. Its long history and culture, special climate and geomorphological features make its tourism resources different from those in adjacent areas, and form the advantages of tourism resources characterized by “Han”, “green” and “water”. Yan ’an is a wonderful flower in the process of developing tourism in northern Shaanxi. Red tourism has become a name card of Yan’an. According to the survey, Shaanxi Province has a total of 486 influential red tourism resources, Yan’an accounted for 360. Overall, Shaanxi province has also been recognized as a province with great cultural and tourism resources. However, the number of tourists and tourism revenue in the country are significantly lower in proportion: the number of domestic and foreign tourists and tourism revenue accounted for 11.67% and 10.87% of the country in 2018, respectively. The development of tourism in Shaanxi has been slowed by the predominance of a single type of tourism product, an unsound tourism management system, and inadequate tourism infrastructure. Since the establishment of Shaanxi Tourism Group in December 1998, Shaanxi has undertaken various exploratory tourism development initiatives (generally referred to in government documents as rationalizing the tourism management system), from the controversial “Qujiang model” to the Shaanxi Tourism Development Commission established early in the country, from the approval of the creation of total region tourism demonstration province to the publication of the implementation plan for the creation of total region tourism demonstration province in Shaanxi, which presents the thrust of high-quality development in three dimensions: politics, polity, and policy. In the context of the new era, the topic of how Shaanxi and other provinces can achieve high-quality development of tourism destinations based on the current situation and looking to the future is of important practical significance.

## Methods

In this section, we introduce the basic principles of the DEA method, the selection of the research model, the selection of research variables, which can provide theoretical basis for the empirical analysis below [22].

### DEA model

The DEA model designed by Charnes et al. [23] used mathematical programming models (e.g., linear programming, multi-objective programming, semi-infinite programming, and stochastic programming, etc.) for analyzing the relative efficiency of decision-making unit (DMU). The DEA method takes multiple input and output data from DMUs and uses mathematical programming models to solve for the points of the efficiency boundary line, which forms the so-called data envelope. This method extends the concept of productivity to production efficiency and gives suggestions on how to reduce the input or increase the output of inefficient DMU to achieve the efficiency value equal to 1, that is, to achieve the most efficient input-output combination. At present, the DEA method has become one of the most widely used mathematical methods in the aspects of relative effectiveness and returns to scale of DMUs under the condition of multi-input and multi-output [24,25]. As a new research field of management science, DEA-related research work (both methodological and practical) is growing at an exponential rate [26]. As tourism is a comprehensive and highly interrelated industry, coupled with the blurred edges of the industry, the evaluation of governance efficiency has been a “black box”[27]. So applying the DEA model to the tourism efficiency of tourism destinations helps the “black box evaluation”.

### A selection of DEA model

O’Neill et al. [28] divided DEA models into two main categories of DEA models for deterministic data and uncertain data and the different sub-categories of models under them. The input-oriented DEA under Charnes-Cooper-Rhodes (CCR) model (constant returns to scale, abbreviated as CRS) and Banker-CharnesCooper (BCC) model (variable returns to scale, abbreviated as VRS) are applied to estimate efficiency levels of tourism destinations [29]. The BCC model is closer to reality. Due to the existence of unequal competition, financial constraints, and other factors, a DMU may not operate in the optimal mode. Therefore, VRS is taken into account, that is, when not all DMU operate at the optimal scale, SE affects the measurement of technical efficiency (TE). Eq (1) is the CCR model and Eq (2) is the BCC model. The model assumes that there are *n* DMUs, each with *s* types of input vectors (i.e., *x*_*i*_) and *t* types of output vectors (i.e., *y*_*i*_). *θ* denotes the input and output variables and the corresponding efficiency score for the *i*-th DMU respectively, and *λ* is a *N* ×1 vector of constant. *I*1 in Eq (2) is a vector of 1’s of *I* x 1. The approach forms a convex hull of intersecting planes which envelops the data points more tightly than the CCR conical hull.


$$\begin{array}{ll} {\text{min}}_{\theta ,\lambda } & \theta \\ st & -y_{i}+Y\lambda \geq 0\\ & \theta x_{i}-X\lambda \geq 0\\ & \lambda \geq 0,i\in n \end{array}$$ (1)

$$\begin{array}{ll} {\text{min}}_{\theta ,\lambda } & \theta \\ st & -y_{i}+Y\lambda \geq 0\\ & \theta x_{i}-X\lambda \geq 0,\\ & I1^{/}\lambda =1\\ & \lambda \geq 0 \end{array}$$ (2)

The quantitative analysis of tourism efficiency of tourism destinations from the perspective of input and output should be seen as a quantitative process of input-oriented analysis, from CCR analysis assuming CRS to BCC analysis assuming VRS, and from analysis of TE_CRS_ and TE_VRS_ to SE, all of which provide a more objective observation of the differences in the tourism efficiency of tourism destination.

### Factor selection and data description

The accuracy of measuring efficiency results using the DEA methodology relies heavily on the input and output indicators used in the measurement process. The ten prefecture-level cities in Shaanxi Province were chosen as the research object of tourism destination, that is, the ten DMUs. The quality evaluation index of a tourism destination is a model similar to the input-output model of an enterprise, in which the resource endowment of the destination and all kinds of supporting facilities are the inputs of the destination system; and the flow of tourists attracted to the destination, the revenues, and the welfare brought to the local residents are the outputs of the destination. The efficiency of a destination’s inputs and outputs is the most intuitive manifestation of quality. In economics, the input of factors of production is generally divided into three parts: land and natural resources, labor, and capital. Given that the attractiveness of the city itself land and natural resources to tourists is an urban tourism production process is an important input, therefore the number of A-grade tourist attractions became one of the input indicators to measure the development quality of tourism destinations. In essence, the tourism industry should satisfy all the needs and services of tourists in the process of traveling, so the inputs to satisfy the needs of tourists in terms of food, accommodation, transportation, and other services should be important inputs in the tourism production process. In this study, we choose the number of star hotels as the capital factor. In addition, due to the characteristics of the blurred boundaries of the tourism industry, the number of direct employment in tourism does not currently have a detailed data compilation in the relevant statistical yearbooks. In terms of the production output of tourism services, most of the tourism development efficiency literature chose tourism revenue or tourist arrivals [30,31]. Tsaur et al. [32] used tourism revenue as output variables. Barros [33] pointed out that the number of guests is an output indicator. Liang and Yang [34] selected tourism revenue and number of tourist as tourism output indicators. Drawing on previous researches, this study selected the number of inbound and domestic tourists and international and domestic tourism revenue as output indicators.

The statistics for the ten prefecture-level cities are taken from the publicly published Shaanxi Regional Tourism Statistical Yearbook-2018 (See supporting information), with some data summation and conversion of units of measurement. For example, inbound tourism numbers and domestic tourism numbers were listed separately and added up as input elements, and international and domestic tourism revenue data were treated in the same way. The software used for data processing was DEA-SOLVER Pro5.

## Data processing and meaning

After extracting the required data from the Shaanxi Provincial Regional Tourism Statistical Yearbook-2018 and creating a database, the data were processed using DEA-SOLVER Pro5 software and selecting the CCR-I (i.e., input perspective) model to obtain Table 1. The difference is the recommended value for increasing or decreasing inputs from an efficiency point of view while keeping output constant. A positive value is a recommended value for an increased input factor, a negative value is a recommended value for a decreased input factor, and zero is a recommended value for no increase or decrease in an input factor. The overall technical efficiency TE_i_ (TE_CRS_) is a key indicator of the quality of development of tourism destinations. As defined by Farrell [29], when a DMU was technically efficient, it would have a TE_i_ score of 1. When it was not technically efficient, it would have a TE_i_ < 1 and fall below the production frontier. SE is obtained by DEA of a CCR model and a BCC model on the same data. If there is a difference between the TE of a particular DMU, under the CCR model and the BCC model, this indicates that the DMU is not SE. In other words, SE can be calculated from the difference between the TE of the BCC model and the TE of the CCR model.

**Table 1:** CCR evaluation of ten cities in Shaanxi (2018).

City	Number of star hotels	Difference	Number of A-grade tourist attractions	Difference	Number of inbound and domestic tourists	Difference	International and domestic tourism revenue	Difference	TE_CRS_
Xi’an	102.00	-25.24	73.00	-18.07	15012.00	2496.33	1213.80	0.00	0.75
Tongchuan	9.00	-4.02	15.00	-6.70	1394.60	0.00	76.56	13.29	0.55
Baoji	28.00	0.00	20.00	0.00	6384.80	0.00	442.69	0.00	1.00
Xianyang	15.00	0.00	46.00	0.00	5342.10	0.00	319.49	0.00	1.00
Weinan	31.00	-9.80	42.00	-13.35	5560.40	746.14	365.99	0.00	0.68
Yan’an	49.00	-31.33	24.00	-11.38	4028.50	0.00	227.98	51.32	0.53
Hanzhong	30.00	-15.99	24.00	-12.79	3260.30	0.00	173.41	50.91	0.47
Yulin	27.00	-16.10	17.00	-9.23	2480.30	0.00	142.02	29.96	0.46
Ankang	26.00	-12.65	27.00	-13.14	3278.90	0.00	171.23	49.88	0.51
Shangluo	18.00	-4.20	27.00	-6.30	3736.60	0.00	199.95	43.49	0.77

Note: Number of inbound and domestic tourists (Unit: 10,000 people); International and domestic tourism revenue (Unit: 100 million RMB).

### Overall technical efficiency

The overall technical efficiency level of Shaanxi Province is poor, and the overall technical efficiency of two cities has reached the optimal efficiency of DEA model, accounting for only 20%. The TE_CRS_ values for both Baoji and Xianyang stand at 1 (as shown in Table 1), signifying that if the number of inbound and domestic tourists, as well as international and domestic tourism revenue, remain unchanged after the year 2018, the indicators for input factors such as the number of star hotels and the number of A-grade tourist attractions cannot be proportionally reduced as a whole.

In fact, they cannot even be partially reduced. The difference values for Baoji and Xianyang are both 0, indicating that their overall technical efficiency is effective within the DEA model. From the perspective of industrial theory, both their pure technical efficiency and scale efficiency are effective. In other words, the quality of development in these two tourist destinations is at a favorable level.

The TE_CRS_ values for the remaining eight cities are less than 1, indicating that if a certain number of star hotels and A-grade tourist attractions (input indicators) are reduced after the year 2018, the number of inbound and domestic tourists and international and domestic tourism revenue can still be maintained at their current levels, without compromising the tourism output of these destinations. It is evident that the tourism development quality of the eight cities, excluding Baoji and Xianyang, is relatively lower (DEA non-efficient), characterized by input redundancy and insufficient output. Furthermore, by examining the difference values in Table 1 (where these values are negative), it becomes clear that there is redundancy in input indicators. For example, the difference value for the count of star hotels in Xi’an is -25.24, while for A-grade tourist attractions, it’s -18.07. This implies that, while keeping the tourism output indicators (inbound and domestic tourists and international and domestic tourism revenue) constant, there is an excess of approximately 26 star hotels and 18 A-grade tourist attractions as input indicators. Examining the specific numerical values, cities like Yan’an, Hanzhong, and Ankang, similar to Xi’an, exhibit significant input redundancy, highlighting a lower quality of development in these tourist destinations.

### Pure technical efficiency

Similarly, utilizing the same database and invoking the DEA-SOLVER Pro5 software, the data could be processed using the BCC-I (input-oriented) model, resulting in the data presented in Table 2. Here, the TE_i_ under this model is referred to as the TE_VRS_, while the interpretations for other data remain consistent with the CCR-I model.

**Table 2:** BCC evaluation of ten cities in Shaanxi (2018).

City	Number of star hotels	Difference	Number of A-grade tourist attractions	Difference	Number of inbound and domestic tourists	Difference	International and domestic tourism revenue	Difference	TE_VRS_
Xi’an	102.00	0.00	73.00	0.00	15012.00	0.00	1213.80	0.00	1.00
Tongchuan	9.00	0.00	15.00	0.00	1394.60	0.00	76.56	0.00	1.00
Baoji	28.00	0.00	20.00	0.00	6384.80	0.00	442.69	0.00	1.00
Xianyang	15.00	0.00	46.00	0.00	5342.10	0.00	319.49	0.00	1.00
Weinan	31.00	-9.43	42.00	-12.78	5560.40	761.22	365.99	0.00	0.70
Yan’an	49.00	-29.97	24.00	-6.36	4028.50	0.00	227.98	41.82	0.73
Hanzhong	30.00	-13.90	24.00	-7.13	3260.30	0.00	173.41	40.84	0.70
Yulin	27.00	-13.87	17.00	-0.91	2480.30	0.00	142.02	14.20	0.95
Ankang	26.00	-9.83	27.00	-10.11	3278.90	0.00	171.23	43.59	0.63
Shangluo	18.00	-2.20	27.00	-3.30	3736.60	0.00	199.95	37.48	0.88

Note: Number of inbound and domestic tourists (Unit: 10,000 people); International and domestic tourism revenue (Unit: 100 million RMB).

Upon comparing Tables 1 and 2, noticeable variations are observed in both TE_i_ and the difference values: the number of cities with effective TE_CRS_ surpasses those with effective TE_VRS_. The cities with TE_VRS_ = 1 increase to include Xi’an and Tongchuan, constituting 40% of all tourism destinations. The absolute values of the difference values for input indicators in generally decrease; for instance, the difference value for the count of star hotels in Yan’an shifts from -31.33 to -29.97.

### Scale efficiency

Based on the TE_CRS_ and TE_VRS_ values obtained from Tables 1 and 2, we can calculate the SE values. According to the formula TE_CRS_ = TE_VRS_×SE, it can be found that SE = TE_CRS_/TE_VRS_ [24], and the relevant values are shown in Table 3. SE values for Baoji and Xianyang are both 1, implying DEA effectiveness and indicating high-quality development of tourism destination. For the other eight cities, SE values are less than 1, in descending order, Weinan, Shangluo, Ankang, Xi’an, Yan’an, Hanzhong, Tongchuan, and Yulin. This suggests that the quality of development in these eight tourism destination cities is not DEA effective, and the smaller the value of SE, the worse their development quality.

**Table 3:** Tourism efficiency of ten cities in Shaanxi (2018).

TE_i_	Xi’an	Tongchuan	Baoji	Xianyang	Weinan	Yan’an	Hanzhong	Yulin	Ankang	Shangluo
TE_CRS_	0.75	0.55	1.00	1.00	0.68	0.53	0.47	0.46	0.51	0.77
TE_VRS_	1.00	1.00	1.00	1.00	0.70	0.73	0.70	0.95	0.63	0.88
SE	0.75	0.55	1.00	1.00	0.98	0.72	0.66	0.48	0.82	0.87

## The spatial distribution characteristics and factors in tourism efficiency

### Spatial distribution characteristics in tourism efficiency

**Fig 1:**
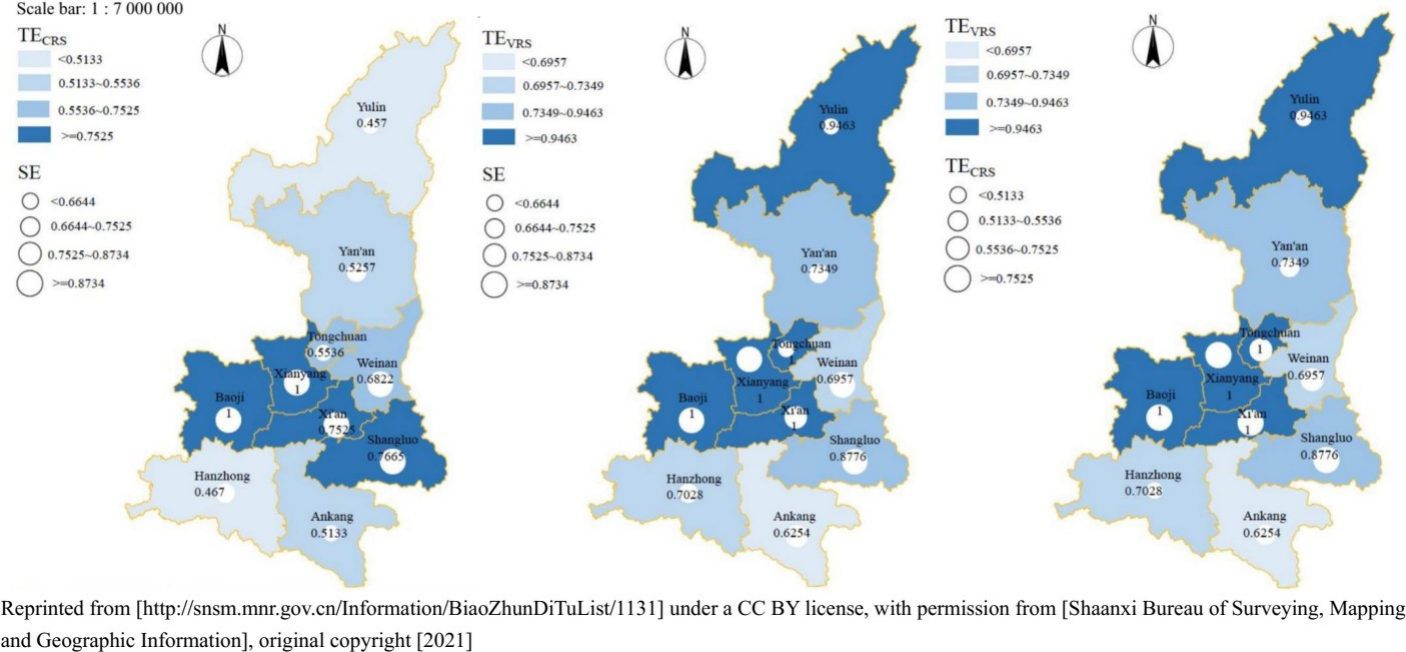
Comparison of TE_CRS_, TE_VRS_, and SE of ten cities in Shaanxi (2018).

Based on the results of the above data analysis, the spatial distribution of differences in the comparison of TE_CRS_, TE_VRS_, and SE of ten prefecture-level cities in Shaanxi province was plotted using SE as the object of comparison (Fig 1), and their spatial distribution characteristics are summarized as follows:

Under the CRS and from the TE_CRS_ perspective, the TE_CRS_ of the ten prefecture-level cities in Shaanxi province exhibits a spatial distribution pattern characterized by higher efficiency in the central region and lower efficiency in the northern and southern parts. The areas demonstrating DEA efficiency are concentrated within the Guanzhong area, including the tourism destinations of Baoji and Xianyang. While Xi’an (0.75) and Shangluo (0.77) do not achieve DEA efficiency, their values are close to being efficient. Conversely, the lower TE_CRS_ values are observed in the southern and northern regions of Shaanxi. Among them, Yulin (0.46) exhibits the lowest efficiency, followed by Hanzhong (0.47), Ankang (0.51), and Yan’an (0.53). A comparison between the distributions of TE_CRS_ and SE reveal that Xianyang and Baoji had the highest levels of tourism development quality, while Yulin (0.48) and Hanzhong (0.66) exhibit lower levels of tourism development quality.

Under the VRS, from the TE_VRS_ perspective, the TE_VRS_ in the ten cities of Shaanxi province is DEA efficiency in the central and western Guanzhong (The TE_VRS_ values for Xi’an, Baoji, Xianyang, and Tongchuan are 1). Although Yulin do not reach DEA efficiency, the value (0.95) is close to 1. Therefore, Shaanxi province as a whole shows a distribution characteristic of low north-south and high middle. The spatial distribution patterns of TE_VRS_ and SE do not exactly match: the range of larger values of SE is smaller than that of TE_VRS_. The former is only found in the central and western of the Guanzhong, while the latter is found both in Yulin (0.95) and Yan’an (0.73) in northern Shaanxi and Shangluo (0.89) in southern Shaanxi.

Comparing the spatial distribution of TE_CRS_ under CRS and TE_VRS_ under VRS, the figure is highly similar to the spatial distribution characteristic figure of TE_VRS_ and SE, with the difference being the difference in size between SE and TE_CRS_ in the same region as indicated by the circles. The tourism destinations with efficient DEA for SE values are Xi’an, Tongchuan, Baoji, and Xianyang, and for TE_CRS_ values DEA efficient tourism destinations are Baoji and Xianyang. In the case of Baoji and Xianyang, this could be explained by the DEA efficiency of the two destinations during the data period; on the other hand, it could be explained by the fact that the tourism products of the two destinations are already "aging" in terms of maturity, i.e., further investment under the CRS would be redundant, and maintaining the status quo without major changes in output is the best option. For Xi’an and Tongchuan, on the other hand, there may be a ’re-growth’ in maturity, which is more likely to result in higher levels of DEA efficiency if measures are taken to increase output levels, as confirmed by the results under VRS.

### Factors influencing the spatial distribution of tourism efficiency

On the whole, the spatial distribution of tourism efficiency and development quality among the ten prefecture-level cities in Shaanxi province reveals a concentration of DEA-efficient or higher-quality development areas in the central Guanzhong region, while areas with lower development quality or non-DEA efficiency are mainly distributed in the southern and northern of Shaanxi. Drawing from tourism industry theories and the practical context of Shaanxi’s tourism industry, the analysis and conclusions regarding the current disparities in tourism development quality and the future direction of input-output relationships are summarized as follows:

The quality of development is related to the endowment of A-grade tourist attractions: The number and endowment of A-grade tourist attractions influence the differentiation of the quality of development of the destination. 4A-grade and above tourist attractions have a strong market appeal and can easily form an effective market attraction circle, despite their high investment. For example, Shaanxi has more than 300 A-grade tourist attractions, of which 119 4A-grade and above tourist attractions with high endowment value (12 5A-grade tourist attractions, 107 4A-grade tourist attractions), tourism destinations located in Guanzhong occupy the majority: including 5 5A-grade tourist attractions such as Famen Temple, Terracotta Warriors, Tang Paradise, Taibai Mountain, and Xi’an City Wall·Forst of Stelae; 46 4A-grade tourist attractions such as Niubeiliang and Louguantai, accounting for 51.7% of the province’s 4A-grade and above tourist attractions. Shaanxi Province 4A grade and above tourist attractions account for less than 30% of the province’s A-grade tourist attractions, but the number of tourists received and the comprehensive tourism revenue are more than 80% of the province’s A-grade tourist attractions. This shows that the quality of development is also closely related to the endowment of A-grade tourist attractions.

The quality of development is related to traffic accessibility: The low quality of development of non-DEA efficient destinations in southern and northern Shaanxi is due to the "rule of tourism distance attenuation" and the travel-time ratio. Xi’an is the transport hub of Shaanxi by land and air: a network of highways, railways, and high-speed trains in the shape of the Chinese character "M" has been formed, and Xi’an airport has 171 waypoints and 313 airlines, making it one of the eight regional hub airports in China. Therefore, the high-traffic accessibility of Xi’an and its surrounding destinations gives them the advantage of being close to tourism-generating regions and having a low travel-time ratio, which allows for better tourism output with less tourism investment.

The quality of development is related to the resource-product types: According to the relationship between tourism resources and products (R-P relationship), tourism product development can be divided into three types: R-P symbiotic type, R-P promotion type, and R-P associated type. R-P symbiotic type relies on the advantages of high taste and attractiveness, which do not need to be vigorously developed, and directly converted into tourism products based on keeping their original ecology, for example, Terracotta Warriors. In contrast, R-P promotion refers to the lower taste of resources, and the development of resources into tourist attractions requires large capital investment and high development intensity, such as Nanhu scenic spot in Hanzhong and Zhenbeitai scenic spot in Yulin. R-P associated types refer to facilities or sites that are other types in nature but combine a certain degree of tourism function, for example, Ankang Hydropower Station. R-P symbiotic type has taken initial shape in the Guanzhong region, while R-P promotion type and R-P associated type are the basic states of tourism scenic areas in the tourism destinations of southern and northern Shaanxi. The lack of attractiveness leads to a lower tourism output, highlighting the non-DEA efficiency of tourism inputs, in other words, the inefficiency of tourism development quality.

## Discussion

Domestic and foreign research on tourism efficiency has made fruitful achievements, which first originated in Europe and America, and the content mainly focuses on the operational efficiency and management efficiency in terms of traditional industrial elements such as hotel industry and travel agencies, especially lacking the research on tourism destinations, so this study expands the research horizons of tourism efficiency. In addition, the study apply DEA ideas and models to the empirical evaluation of tourism sustainability, analyze the evaluation results according to the relationship between DEA effectiveness and indicator set, and provide a scientific basis for cities and destinations to formulate relevant tourism countermeasures. In this respect, it makes up for the lack of empirical research on urban tourism efficiency using DEA model at home and abroad. Specifically, this study explored the governance efficiency of tourism destinations from an input perspective using the CCR and BCC models of the DEA model, taking ten cities in Shaanxi province as the case. Observing the development quality of tourism destinations from four perspectives, namely, improved difference, TE_CRS_, TE_VRS_, and SE, helps to understand the status quo of high-quality development level of tourism destinations and provides a new perspective for evaluating tourism quality methods.

From a general point of view, the development quality of tourism destinations in Shaanxi province is low, with excessive inputs and insufficient outputs, in response to which the government should optimize the allocation of inputs and strengthen the governance of tourism destinations [35]. It is recommended that Shaanxi Province adjust the input allocation in the new development stage. Especially in the southern and northern regions of Shaanxi, the subpar quality of tourism development is attributed to both input redundancy and regional disadvantages such as distance attenuation and travel time ratios. In the future, it is advisable to reduce the construction of star-rated hotels and A-grade attractions. Efforts should be focused on enhancing service management and brand reputation, while also prioritizing the improvement of transportation accessibility to boost output and minimize input redundancy.

The higher quality tourism destinations are concentrated in the central and western parts of the Guanzhong, which serves as the core source of Shaanxi’s appeal to outside tourists. In the post-pandemic era, seizing the opportunity to develop Xi’an as an "International Tourism Hub" is crucial for maintaining and enhancing this core attraction. First and foremost, it is crucial to prioritize the protection and promotion of cultural heritage in Xi’an. Enhance the conservation and restoration of renowned landmarks, while also organizing regular cultural festivals and exhibitions to infuse vitality and attractiveness into the tourism city. Secondly, develop effective marketing strategies encompassing both online and offline promotional activities to elevate Xi’an’s visibility and image. Thirdly, actively expand into the international market to attract more overseas tourists. Establish collaborations with international airlines, travel agencies, and other entities to provide convenient international travel logistics and services.

### Limitations and future research

Based on the identified limitations in the study, the following recommendations are proposed: firstly, the study presents the case of multiple DMUs with TE_CRS_ and TE_VRS_ of 1, and therefore tourism destinations with an efficiency value of 1 cannot be ranked according to their governance efficiency. In response to the above, DEA provides a super-efficiency model [36], which calculates efficiency values that can be greater than one, essentially enabling the ranking of the quality of development of tourism destinations. The economic significance of an efficiency value greater than one is very useful in practice, as it can measure the extent to which the inputs to the development process of a tourism destination can remain DEA efficient when various input indicators are increased in proportion at the same time. Secondly, the disadvantage of SE is that its value does not indicate whether the SE of a destination is increasing or decreasing within a certain range, and it is recommended that analysis be conducted through the decreasing returns to scale and non-increasing returns to scale models to more accurately determine whether SE are increasing or decreasing [37]. In addition, the study examined the quality of destination development from a supply-side perspective, using the CCR and BCC models from an input perspective. Future research can conduct DEA analysis from the output perspective to derive the difference values from the output perspective, which can provide objective targets for the development of the demand-side of tourism. Finally, the study is only based on data from ten cities in Shaanxi for one year in 2018, and the next step can be a more comprehensive study of other provinces or even the whole country based on panel data, which can provide an objective analysis of the changing pattern of the development quality of tourism destinations in the selected range over time, and thus obtain rational development suggestions for the high-quality development of tourism destinations.

### Conclusion

The magnitude of the three tourism efficiency values can effectively reflect the level of qualitative development of tourist destinations. The conclusion of the comparison of tourism efficiency can widely explain the current development status of tourism destinations. On the whole, the quality of tourism destination development in Shaanxi province is low, and there are problems of over-investment and insufficient output. The spatial pattern of development quality in tourism destinations within Shaanxi Province exhibits a characteristic of being higher in the middle and lower in the north and south. This pattern is closely related to the endowment of A-grade tourist attractions, traffic accessibility, and the resource-product type in the Guanzhong, southern Shaanxi, and northern Shaanxi.

## Supporting information

S1 File Supporting information includes statistical yearbooks and models constructed on the basis of statistical yearbook data. (ZIP)
