# Supplementary material for: A study on the tourism efficiency of tourism destination based on DEA model: A case of ten cities in Shaanxi province
Source: PLoS One. 2024 Jan 19;19(1):e0296660. doi: 10.1371/journal.pone.0296660 (PMC10798521; doi:10.1371/journal.pone.0296660)
Supplement: S1 File — (ZIP) [file pone.0296660.s001.zip › Supporting information/Statistical yearbook/Ankang.caj]

十二、安康市

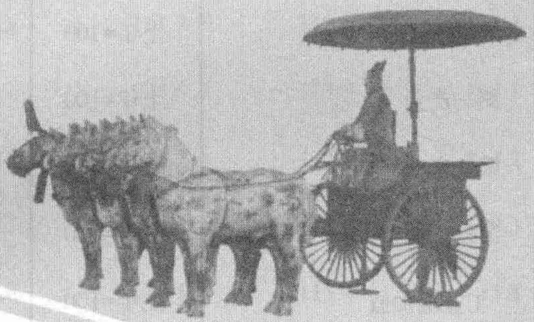

资料整理：栗丰琼　张　梦

?1994-2018 China Academic Journal Electronic Publishing House. All rights reserved.　http://www.cnki.net

12-1 安康市经济

指　　标	单 位	2000年	2005年	2006年	2007年	2008年
年底总人口	万人	292.43	264.81	265.01	265.25	265.71
人口自然增长率	‰		1.55	1.93	2.20	2.15
年底总户数	万户	79.24	88.05	90.45	92.66	94.71
生产总值	亿元	74.80	143.76	163.57	191.37	241.24
第一产业	亿元	22.76	36.71	42.44	48.69	63.79
第二产业	亿元	20.29	42.83	49.05	60.71	79.42
第三产业	亿元	31.75	64.22	72.08	81.97	98.03
# 工业增加值	亿元	12.41	28.23	32.26	40.03	50.40
人均生产总值	元	2561	5413	6175	7218	9087
生产总值指数	上年=100	105.8	109.8	110.4	112.8	115.4
第一产业	上年=100	106.0	110.4	108.0	106.3	107.7
第二产业	上年=100	102.8	109.1	112.5	116.5	121.6
第三产业	上年=100	108.9	109.9	110.4	113.9	115.2
# 工业增加值	上年=100	101.4	108.0	111.1	118.0	119.4
人均生产总值指数	上年=100	105.6	109.6	110.7	112.7	115.3
非公有制经济增加值	亿元		60.58	72.06	85.58	110.62
文化产业增加值	亿元					
单位GDP能耗	吨标准煤/万元		1.380	1.340	1.276	1.198
单位GDP能耗比上年增长	%			-2.90	-4.75	-6.15
就业人员	万人	135.44			155.52	149.72
城镇单位就业人员	万人	13.07	12.15	12.00	11.85	12.35
# 国有单位	万人	10.99	10.23	10.04	10.05	10.28
集体单位	万人	1.20	0.78	0.81	0.63	0.70
# 在岗职工人数	万人	12.76	11.89	11.58	11.31	11.63
城镇单位就业人员平均工资	元					
城镇单位在岗职工平均工资	元	6766	11953	13485	18266	22864

?1994-2018 China Academic Journal Electronic Publishing House. All rights reserved. http://www.cnki.net

社会主要指标

2009年	2010年	2011年	2012年	2013年	2014年	2015年	2016年
263.24	263.08	263.07	263.36	263.76	264.20	265.00	265.60
2.10	2.78	2.57	2.75	2.85	2.72	2.32	2.79
97.02	98.96	100.54	102.69	105.06	105.40	105.03	106.10
274.95	327.06	407.17	496.91	604.55	689.44	755.05	842.86
65.59	67.07	72.01	80.95	88.73	93.01	96.06	100.12
96.83	130.95	183.13	243.47	315.99	371.03	403.39	450.64
112.53	129.04	152.03	172.49	199.83	225.40	255.60	292.10
60.36	86.15	127.13	179.91	238.41	282.41	309.94	348.84
10341	12428	15477	18878	22938	26117	28536	31770
115.0	115.0	115.5	115.2	113.4	111.7	112.1	111.3
106.2	106.4	106.5	105.7	105.1	105.5	104.9	104.1
120.2	121.5	122.9	123.6	119.8	115.7	114.8	114.2
115.5	113.9	112.6	110.5	109.5	109.1	111.3	109.3
116.4	123.3	126.0	130.2	122.8	117.1	115.4	115.8
114.8	115.0	115.5	115.1	113.3	111.5	111.8	111.0
128.81	156.53	198.52	247.20	307.86	364.78	408.86	467.37
					20.62	23.01	25.79
1.148	0.701	0.677	0.652	0.631	0.602	0.578	0.453
-4.20	-3.89	-3.50	-3.63	-3.33	-4.50	-4.00	-4.51
150.92	153.10	152.06	152.70	154.49	154.53	155.25	156.02
12.27	12.87	13.91	14.79	17.16	17.63	18.05	18.77
9.52	9.83	9.78	10.11	9.99	10.25	10.26	10.64
0.68	0.78	0.84	0.80	0.73	0.58	0.46	0.34
11.47	11.95	13.01	13.84	15.71	16.09	16.52	16.87
			39629	41180	42714	48628	52195
27447	32903	37802	40951	42754	44000	50410	54225

?1994-2018 China Academic Journal Electronic Publishing House. All rights reserved. http://www.cnki.net

12-1　续表 1

指　　标	单 位	2000年	2005年	2006年	2007年	2008年
全社会固定资产投资	亿元	27.77	60.57	108.28	147.74	200.69
# 房地产开发	亿元	3.36	6.58	6.53	6.32	9.78
商品房销售面积	万平方米	10.81	50.72	57.72	69.33	82.24
# 住宅	万平方米	10.01	48.73	54.34	67.19	77.71
地方财政收入	亿元	3.36	3.46	4.06	5.81	7.49
地方财政支出	亿元	9.70	19.16	24.84	34.90	58.18
金融机构人民币各项存款余额	亿元	78.25	163.58	195.82	225.43	300.16
金融机构人民币各项贷款余额	亿元	78.27	97.47	109.32	125.50	138.81
农村居民人均纯收入	元	1248	1799	1953	2256	2770
城镇居民人均可支配收入	元	4305	6388	6860	8051	10150
城市人均公园绿地面积	平方米			9.4	12.5	11.2
城市人均道路面积	平方米		14.0	17.1	16.7	15.0
城市用水普及率	%		95.2	96.0	96.3	86.4
城市用气普及率	%		73.4	66.2	70.4	63.2
常用耕地面积	千公顷	225.86	192.40	192.24	191.48	193.21
农林牧渔业总产值	亿元	39.29	62.34	71.37	82.55	108.16
农作物总播种面积	千公顷	492.15	452.54	453.69	401.78	421.30
# 粮食作物	千公顷	398.80	319.57	319.16	266.33	272.95
粮食产量	万吨	98.24	99.88	95.70	75.23	82.12
棉花产量	吨	69	26	16	14	23
油料产量	吨	40243	72788	77929	79606	94859
蔬菜产量	吨	255900	630214	721987	723592	847424

?1994-2018 China Academic Journal Electronic Publishing House. All rights reserved.　http://www.cnki.net

2009年	2010年	2011年	2012年	2013年	2014年	2015年	2016年
272.77	360.05	304.49	380.27	482.56	605.56	758.16	926.37
12.67	16.65	22.32	36.40	50.53	53.48	65.50	95.12
103.13	119.05	133.14	121.69	120.62	138.39	127.26	13.60
95.36	107.19	124.36	117.09	113.85	133.61	121.05	132.28
9.72	13.23	17.34	21.66	25.34	28.09	30.84	30.11
78.54	110.25	127.15	162.28	187.21	204.74	224.60	247.80
383.13	475.54	567.38	673.09	790.35	884.51	1032.71	1172.40
177.39	211.34	258.59	308.97	383.11	474.35	547.20	627.01
3313	3976	5009	5815	6624	7210	7913	8590
12525	14642	17365	20300	22533	22062	23985	25962
10.1	10.0	10.6	9.8	11.1	13.2	13.9	13.3
13.7	13.9	13.9	15.3	16.1	16.1	16.5	15.8
81.6	84.7	80.7	90.5	90.2	94.4	94.6	95.2
59.5	59.6	81.5	88.6	58.8	81.2	97.9	98.1
194.33	195.54	197.10	197.94	197.82	197.30	196.36	196.19
112.64	115.69	124.67	140.39	156.62	163.75	169.55	177.06
439.29	453.80	440.64	449.35	453.45	477.00	482.64	483.57
278.00	287.45	267.81	269.87	269.91	268.70	269.53	268.71
88.46	93.09	80.13	85.18	86.23	86.04	87.15	87.65
51	44	47	36	31	35	27	27
108611	108611	125759	134609	144383	147893	152620	151482
977270	1083087	1137575	1210846	1314567	1400300	1486471	1539100

?1994-2018 China Academic Journal Electronic Publishing House. All rights reserved. http://www.cnki.net

12-1 续表 2

指　　标	单 位	2000年	2005年	2006年	2007年	2008年
水果产量	吨	34463	83009	98197	117205	138560
# 苹果	吨	2323	3693	3795	3776	4123
肉类产量	吨	87908	144176	171348	147899	165600
# 猪牛羊肉	吨	83726	134457	160079	130470	151328
奶类产量	吨	255	577	747	572	592
# 牛奶	吨	255	577	747	572	592
禽蛋产量	吨	7949	13799	17166	20801	23554
水产品产量	吨	4060	4968	5746	1841	2002
规模以上工业企业单位数	个	135	152	173	159	187
规模以上工业总产值	亿元	20.06	43.57	56.28	81.56	97.48
原煤产量	万吨	1.29	14.36	28.24	50.63	52.77
发电量	亿千瓦小时	30.97	40.24	28.98	41.35	43.21
水泥产量	万吨	23.77	69.77	87.38	106.75	126.23
建筑业企业单位数	个	58	51	54	53	52
建筑业企业年末从业人员	万人	0.96	1.20	1.53	1.07	1.34
建筑业总产值	亿元	3.21	7.35	9.31	10.19	15.70
房屋建筑施工面积	万平方米	104.16	151.89	143.77	134.37	234.81
房屋建筑竣工面积	万平方米	47.34	78.14	71.55	59.19	127.51
公路里程	公里	5597	6369	15088	15806	17211
# 等级公路	公里	4699	5252	3905	9639	11734
# 高速公路	公里				91	93
民用汽车拥有量	辆	10473	25844	31748	39406	42728
# 私人汽车	辆	2686	17118	24165	33166	29510
邮电业务总量	亿元	3.29	12.46	17.42	22.18	27.56
邮政业务总量	亿元	0.39	0.91	1.18	1.26	1.45
电信业务总量	亿元	2.90	11.54	16.25	20.92	26.12

?1994-2018 China Academic Journal Electronic Publishing House. All rights reserved. http://www.cnki.net

2009年	2010年	2011年	2012年	2013年	2014年	2015年	2016年
155521	167647	189243	194959	201843	211874	223563	228409
4356	4526	5133	5204	5246	5172	5320	5424
192159	236389	249971	261390	272356	281145	278195	276403
176247	218518	229449	240566	250313	259078	255312	254491
246	330	320	140	80	82	310	314
246	330	320	140	80	82	310	314
27448	30440	34052	35469	37837	37744	38855	39308
3048	15197	28496	35936	42572	42356	45194	46116
264	282	304	363	393	460	505	568
132.47	192.15	309.22	482.20	634.82	787.97	945.07	1129.48
56.69	58.00	75.01	106.30	87.83	48.74	44.44	51.20
56.07	54.36	67.83	61.00	57.93	54.57	66.42	57.75
421.87	617.23	660.91	707.75	698.91	711.41	764.43	807.84
53	54	74	78	78	88	103	122
1.78	2.01	2.29	2.86	3.46	3.63	4.51	5.03
22.12	30.23	41.06	55.28	69.87	89.00	101.58	119.69
282.06	315.23	464.32	599.75	733.69	830.37	958.25	1131.09
99.02	115.74	138.73	211.33	263.24	275.24	290.01	339.79
19458	19973	20859	22182	22543	22695	22790	22981
15482	16550	17619	18417	18748	19065	19201	19464
151	349	499	516	516	516	539	522
53797	63592	73098	84763	95106	93639	111580	131322
38928	47771	56562	67123	78137	79779	96675	116805
26.39	11.89	16.26	16.65	18.14	26.16	37.24	39.64
1.63	1.54	1.80	1.50	1.86	2.21	2.60	3.11
24.76	10.35	14.46	15.15	16.29	23.95	34.64	36.53

?1994-2018 China Academic Journal Electronic Publishing House. All rights reserved. http://www.cnki.net

12-1 续表 3

指 标	单 位	2000年	2005年	2006年	2007年	2008年
固定电话用户	万户	16.11	40.46	46.77	52.96	49.88
移动电话用户	万户	4.76	40.26	52.14	75.94	89.83
互联网宽带用户	万户	0.78	3.33	4.68	6.97	10.65
限额以上企业数	个					
批发业	个					
零售业	个					
住宿业	个					
餐饮业	个					
社会消费品零售总额	亿元	27.68	46.75	53.83	64.12	80.72
进出口总额	万美元		119	271	350	1043
# 出口	万美元		118	271	349	1032
实际外商直接投资额	万美元	31		203	2054	3025
入境旅游人数	万人次	0.18	0.51	0.60	0.80	1.10
# 外国人	万人次	0.18	0.51	0.36	0.42	0.52
国际旅游外汇收入	万美元	54	86	103	144	200
国内旅游人数	万人次	51	130	160	206	312
国内旅游收入	亿元		4.16	5.60	7.21	11.22
星级饭店数	个		12	12	21	21
幼儿园数	所	32	74	78	98	137
在园儿童数	万人	3.49	3.25	3.18	3.33	3.76
普通小学学校数	所	2677	1724	1487	1303	1175
普通小学专任教师数	人	15023	16101	15849	15755	15307
普通小学在校学生数	万人	38.25	27.86	26.38	24.97	23.36
普通中学学校数	所	210	222	225	225	222
普通中学专任教师数	人	7030	9878	10233	10879	11147
普通中学在校学生数	万人	13.96	19.73	20.09	20.38	19.79
卫生机构数	个	479	448	451	382	373
卫生机构床位数	张	4607	5281	5702	6290	6791
卫生技术人员	人	7900	7739	7999	7609	7540
# 执业(助理)医师	人	4333	3972	4345	3691	3528
注册护师、护士	人	1693	1869	1734	1967	1998

?1994-2018 China Academic Journal Electronic Publishing House. All rights reserved. http://www.cnki.net

2009年	2010年	2011年	2012年	2013年	2014年	2015年	2016年
44.62	43.31	40.05	39.09	38.73	36.69	34.52	33.03
107.08	119.85	134.41	152.17	159.26	174.22	186.79	199.92
11.30	15.75	19.39	22.80	25.35	27.41	29.34	40.32
	180	220	288	402	460	544	699
	31	36	41	50	65	67	88
	84	106	149	217	249	306	401
	32	33	40	47	53	59	71
	33	45	58	88	93	112	139
93.56	110.78	129.72	151.49	171.72	193.18	219.20	259.81
1687	1651	1740	2384	2925	2.17(亿元)	2.95(亿元)	2.02(亿元)
1687	1651	1740	2384	2778	2.17(亿元)	2.95(亿元)	1.84(亿元)
575	564	847	3000	3002	3000		
1.31	1.46	1.80	2.00	2.20	2.42	2.62	2.95
0.76	0.22	0.27	0.08	0.30	0.10	0.17	0.25
239	263	360	400	450	532	590	657
600	1218	1560	1835	2164	2527	2849	3276
21.83	47.50	63.15	76.14	95.21	119.76	143.86	170.79
23	26	27	25	25	30	30	26
153	199	295	308	346	393	440	464
3.99	4.88	6.31	7.09	7.65	7.86	8.32	8.70
1000	886	839	773	686	639	631	578
14866	14364	12432	11899	11188	10769	10395	10365
21.96	20.84	19.95	18.15	17.44	17.56	17.83	18.28
216	209	199	196	196	196	200	201
11334	11366	12461	11081	12451	12547	11479	12826
19.13	18.27	17.47	15.97	14.88	14.47	14.11	14.29
373	368	3189	3172	3275	3190	3113	3042
7592	8059	8598	9968	11354	12595	13215	13976
8570	9142	9966	11754	13582	14351	14765	16276
3612	3605	3906	4088	4358	4288	4349	4754
2466	2715	3162	3747	4784	5381	5752	6394

?1994-2018 China Academic Journal Electronic Publishing House. All rights reserved.   http://www.cnki.net

12-2 汉滨区经济

指 标	单 位	2000年	2005年	2006年	2007年	2008年
年底总人口	万人	93.12	88.17	88.28	88.36	88.52
生产总值	亿元	27.96	53.10	60.87	70.29	85.60
第一产业	亿元	4.92	8.78	9.98	11.49	14.84
第二产业	亿元	8.78	16.99	19.24	22.31	27.40
第三产业	亿元	14.26	27.33	31.65	36.49	43.36
# 工业增加值	亿元	5.56	11.06	12.35	13.73	15.55
人均生产总值	元	3019	6057	6899	7958	9679
生产总值指数	上年=100	110.4	109.3	109.1	112.7	114.2
全社会固定资产投资	万元	95874	194752	230614	288156	358994
地方财政收入	万元	9205	8237	9490	12650	15090
地方财政支出	万元	20353	38956	47308	73516	110493
农村居民人均纯收入	元	1237	1861	2013	2295	2821
城镇居民人均可支配收入	元	4700	6365	7090	8360	10491
常用耕地面积	公顷	48471	41699	42230	42525	42902
粮食产量	吨	260386	278788	171529	195349	212912
农林牧渔业总产值	万元	72635	144850	164687	192142	246281
社会消费品零售总额	万元	125801	205965	237813	286578	361615
普通小学专任教师数	人	4637	5219	5135	5196	5145
普通小学在校学生数	人	132121	97450	93659	90919	86644
普通中学专任教师数	人	2427	3283	3385	3732	3817
普通中学在校学生数	人	48384	70611	70773	70493	67761
卫生机构床位数	张	1954	2265	2403	2644	3046
卫生技术人员	人	3801	3222	2849	3269	3416
# 执业(助理)医师	人	1183	1654	1966	1599	1636
注册护师、护士	人	811	965	832	977	1053

?1994-2018 China Academic Journal Electronic Publishing House. All rights reserved. http://www.cnki.net

社会主要指标

2009年	2010年	2011年	2012年	2013年	2014年	2015年	2016年
86.91	87.04	87.03	87.13	87.26	87.41	87.67	87.87
96.24	114.20	136.95	159.28	187.47	210.39	224.69	253.91
15.03	15.29	16.55	19.20	21.64	22.18	22.71	23.70
30.53	39.51	54.70	65.16	81.71	91.60	94.45	108.74
50.68	59.40	65.70	74.93	84.12	96.60	107.53	121.47
16.00	21.24	31.27	37.86	49.12	54.08	56.33	67.19
10859	13130	15735	18291	21500	24090	25667	28929
114.8	114.7	114.8	114.6	112.9	111.0	111.6	110.5
459023	568407	703867	955104	1267116	1844869	2419241	3068262
18165	22152	29790	39920	49158	52206	58546	53861
162875	225967	261279	321967	355397	409899	492528	510102
3364	4020	5099	5920	6731	7572	7849	8506
13268	15683	18710	22115	24835	27890	24332	26389
43122	43137	43457	43255	42932	42740	41938	41582
225418	242364	206118	217363	218628	214162	214262	215201
251298	257321	277629	322257	361282	376875	387029	403961
415107	527087	618102	721536	812667	895950	1008981	1165194
4997	4833	4642	3700	3595	3701	3553	4384
82747	79425	76613	66626	63876	63466	62306	62880
3855	3806	4528	4565	4747	5062	5315	4443
65378	63058	61624	57325	55951	56087	56686	57606
3437	3599	3804	4509	4985	5506	5616	5698
3814	4156	4578	4869	5637	5926	6390	6821
1756	1797	1815	1817	1793	1832	1991	2154
1195	1353	1641	1781	2327	2635	2781	2919

?1994-2018 China Academic Journal Electronic Publishing House. All rights reserved. http://www.cnki.net

12-3 汉阴县经济

指　　标	单 位	2000年	2005年	2006年	2007年	2008年
年底总人口	万人	28.79	24.83	24.86	24.88	24.92
生产总值	亿元	5.29	11.28	13.22	15.66	20.11
第一产业	亿元	2.38	4.48	5.18	5.98	7.76
第二产业	亿元	0.99	2.60	3.22	3.94	5.38
第三产业	亿元	1.93	4.20	4.82	5.74	6.97
# 工业增加值	亿元	0.58	1.88	2.42	3.01	4.11
人均生产总值	元	1634	3855	5312	6304	8089
生产总值指数	上年=100	115.7	112.2	111.3	112.9	116.2
全社会固定资产投资	万元	22891	49014	56273	81302	122614
地方财政收入	万元	2518	1836	2039	3002	4015
地方财政支出	万元	7847	14901	22548	30073	51506
农村居民人均纯收入	元	1206	1839	1990	2285	2772
城镇居民人均可支配收入	元		6593	6957	7961	9988
常用耕地面积	公顷	19853	20997	20991	21206	21800
粮食产量	吨	92358	109878	103119	84700	93728
农林牧渔业总产值	万元	39817	73607	85135	100142	128917
社会消费品零售总额	万元	15999	32910	37502	43778	55124
普通小学专任教师数	人	1255	1805	1775	1716	1632
普通小学在校学生数	人	37600	26500	25700	24600	23700
普通中学专任教师数	人	624	1040	1032	1009	1300
普通中学在校学生数	人	13500	21100	20200	19200	14100
卫生机构床位数	张	314	336	388	407	435
卫生技术人员	人	468	445	504	516	545
# 执业(助理)医师	人	238	327	351	363	392
注册护师、护士	人	90	113	120	134	154

?1994-2018 China Academic Journal Electronic Publishing House. All rights reserved.　http://www.cnki.net

社会主要指标

2009年	2010年	2011年	2012年	2013年	2014年	2015年	2016年
24.60	24.62	24.62	24.65	24.68	24.72	24.79	24.85
23.83	28.45	35.11	45.94	58.61	68.44	77.51	83.98
8.09	8.35	8.88	9.82	11.22	11.68	12.26	12.78
7.46	10.15	14.68	22.94	32.62	39.15	43.43	48.48
8.28	9.94	11.55	13.17	14.78	17.60	19.82	22.72
5.87	8.06	12.06	20.05	29.32	33.72	37.42	41.94
9551	11558	14261	18648	23764	27711	30501	33834
116.3	114.9	115.6	115.2	114.5	113.4	112.4	111.5
172150	223190	243699	321922	410791	520363	659608	834797
6433	8047	10898	14469	17519	19900	21500	21200
64728	82360	106689	135000	157000	171001	189000	192000
3322	4053	5123	5938	6858	7797	8063	8745
12323	14590	17289	20228	22676	25420	23995	26000
22749	22941	23279	23285	23269	23041	22947	22901
102374	110219	92555	100027	102453	103270	101208	101647
136150	141242	150879	167092	189776	200726	208869	218373
65975	73843	86042	100579	116006	133329	153530	200466
1587	1496	1438	1340	1101	1018	986	1102
23100	22268	21730	20694	18584	18526	19489	20509
1057	1034	1073	930	972	987	1061	1026
17800	16845	16304	14798	13626	13309	13310	13965
438	491	650	790	814	1030	1102	1126
597	618	758	807	1063	1058	1199	1335
417	436	461	473	410	313	354	417
180	218	248	289	300	381	435	497

?1994-2018 China Academic Journal Electronic Publishing House. All rights reserved. http://www.cnki.net

12-4 石泉县经济

指　标	单 位	2000年	2005年	2006年	2007年	2008年
年底总人口	万人	18.26	17.49	17.48	17.50	17.53
生产总值	亿元	4.59	9.50	11.30	13.61	17.86
第一产业	亿元	1.18	2.49	2.91	3.37	4.36
第二产业	亿元	1.64	3.40	4.21	5.40	7.85
第三产业	亿元	1.77	3.61	4.17	4.84	5.65
# 工业增加值	亿元	0.98	2.08	2.65	3.53	5.29
人均生产总值	元	2520	5335	6516	7783	10197
生产总值指数	上年=100	114.0	112.1	114.5	115.8	118.1
全社会固定资产投资	万元	36747	85477	111603	104666	167934
地方财政收入	万元	1837	1358	1548	2923	3600
地方财政支出	万元	5602	12737	16856	22100	41795
农村居民人均纯收入	元	1227	1666	1961	2179	2722
城镇居民人均可支配收入	元	3860	6662	7082	8030	10132
常用耕地面积	公顷	15764	14341	13966	12640	12924
粮食产量	吨	67915	73528	78179	59031	64954
农林牧渔业总产值	万元	22685	42858	50138	58838	74730
社会消费品零售总额	万元	13322	24140	27675	32775	41193
普通小学专任教师数	人	1059	985	952	923	923
普通小学在校学生数	人	21471	16399	15935	15046	14238
普通中学专任教师数	人	452	643	660	681	711
普通中学在校学生数	人	8669	10837	11309	11628	11194
卫生机构床位数	张	297	403	493	541	575
卫生技术人员	人	413	425	422	415	387
# 执业(助理)医师	人	178	195	191	182	138
注册护师、护士	人	96	98	111	113	124

?1994-2018 China Academic Journal Electronic Publishing House. All rights reserved.　http://www.cnki.net

社会主要指标

2009年	2010年	2011年	2012年	2013年	2014年	2015年	2016年
17.04	17.12	17.13	17.14	17.17	17.20	17.25	17.29
22.44	27.00	33.65	38.24	47.79	54.76	60.37	68.51
4.46	4.59	5.08	5.61	6.25	6.33	6.55	6.96
11.19	14.95	20.20	23.12	31.04	35.69	38.93	43.71
6.80	7.46	8.38	9.52	10.50	12.75	14.89	17.84
8.27	11.32	16.06	18.58	25.84	29.79	32.58	36.77
12793	15814	19656	22316	27855	31863	35046	39665
118.4	116.1	116.9	117.3	114.7	113.3	113.9	112.5
195181	231250	236336	313083	402384	509597	649036	822196
4499	5430	6853	9378	11368	12985	14696	15004
50084	68310	80695	121498	118995	133666	161888	157006
3338	4026	5101	5948	6786	7675	8011	8753
12666	14794	17573	20525	22988	25747	23905	25854
12980	13036	13051	13086	13099	13100	13111	13118
69430	72011	62700	66459	67048	66859	69911	70668
76985	79063	87323	96428	107031	110801	115214	122292
49337	53490	62751	72349	82491	95063	109174	130942
917	888	868	803	812	796	714	794
13229	12378	11535	10111	9174	9465	9882	10288
718	741	750	738	724	748	822	710
10990	10437	10552	9198	7824	7388	7302	7460
571	580	562	552	589	729	772	963
382	392	517	543	634	642	769	1008
168	195	186	177	241	201	182	279
105	113	112	189	224	247	304	395

?1994-2018 China Academic Journal Electronic Publishing House. All rights reserved. http://www.cnki.net

12-5 宁陕县经济

指　　标	单 位	2000年	2005年	2006年	2007年	2008年
年底总人口	万人	7.37	7.03	7.04	7.04	7.06
生产总值	亿元	3.16	4.16	4.96	6.07	8.30
第一产业	亿元	1.31	1.37	1.65	1.91	2.45
第二产业	亿元	0.90	1.30	1.56	2.08	3.30
第三产业	亿元	0.95	1.50	1.75	2.07	2.55
# 工业增加值	亿元	0.63	0.38	0.50	0.79	1.63
人均生产总值	元	4308	5583	7216	8585	11702
生产总值指数	上年=100	103.4	111.2	113.3	114.9	116.4
全社会固定资产投资	万元	12513	17139	23739	44772	75890
地方财政收入	万元	1222	736	875	1201	1524
地方财政支出	万元	4789	8125	9806	15225	23821
农村居民人均纯收入	元	1210	1511	1662	1940	2431
城镇居民人均可支配收入	元	4539	5683	6178	7426	9253
常用耕地面积	公顷	5679	3212	3216	3238	3294
粮食产量	吨	34618	21882	23213	17446	18708
农林牧渔业总产值	万元	20568	23071	27846	32346	42451
社会消费品零售总额	万元	10596	11183	12706	14997	18783
普通小学专任教师数	人	530	507	468	437	432
普通小学在校学生数	人	8695	6210	5452	4270	3974
普通中学专任教师数	人	170	214	243	264	251
普通中学在校学生数	人	3162	4732	4772	4603	4341
卫生机构床位数	张	219	187	188	216	221
卫生技术人员	人	248	226	225	248	282
# 执业(助理)医师	人	121	97	90	112	112
注册护师、护士	人	51	55	50	61	82

?1994-2018 China Academic Journal Electronic Publishing House. All rights reserved.　http://www.cnki.net

社会主要指标

2009年	2010年	2011年	2012年	2013年	2014年	2015年	2016年
7.10	7.05	7.05	7.05	7.06	7.08	7.10	7.11
9.80	11.61	14.66	18.32	21.35	22.22	24.11	25.85
2.57	2.67	2.99	3.53	3.93	4.06	4.20	4.34
4.24	5.35	7.52	10.03	12.34	12.39	13.08	13.81
3.00	3.59	4.15	4.76	5.09	5.76	6.83	7.70
2.08	2.51	4.14	6.17	7.99	7.47	7.81	8.07
13885	16418	20807	25982	30250	31426	34023	36379
115.8	114.9	116.1	116.6	111.6	109.1	113.8	110.5
103126	129104	105963	148074	193010	257033	316108	292833
2246	3075	3858	5307	6351	7181	8215	8602
31944	44886	50504	68973	80249	80040	89699	95437
3201	3812	4815	5629	6445	7315	7625	8270
12020	14196	16794	19733	21785	24116	23338	25358
3283	3316	3372	3386	3390	3392	3403	3404
21070	21869	19049	19848	19935	19212	19423	19611
45421	47152	52611	62968	70647	74604	77333	80355
22504	25639	29747	34815	38955	41460	46893	53395
412	420	344	306	352	325	333	340
3741	3601	3559	3610	3611	3753	3771	3857
256	246	256	253	306	276	268	250
4079	3823	3579	3185	3041	2971	2818	2878
252	262	280	281	290	305	328	348
318	310	308	342	437	350	464	469
119	110	106	98	129	108	128	137
84	84	83	108	148	113	159	178

?1994-2018 China Academic Journal Electronic Publishing House. All rights reserved.   http://www.cnki.net

12-6 紫阳县经济

指　　标	单 位	2000年	2005年	2006年	2007年	2008年
年底总人口	万人	34.12	28.76	28.79	28.81	28.86
生产总值	亿元	5.77	12.17	13.95	16.66	20.76
第一产业	亿元	3.11	5.16	5.84	7.09	8.50
第二产业	亿元	0.80	1.93	2.42	3.07	4.72
第三产业	亿元	1.86	5.08	5.70	6.50	7.55
# 工业增加值	亿元	0.38	0.96	1.18	1.45	2.21
人均生产总值	元	1673	3621	4849	5784	7198
生产总值指数	上年=100	104.5	109.1	110.3	111.9	115.2
全社会固定资产投资	万元	18380	48521	58017	89597	143212
地方财政收入	万元	2413	1609	1871	3258	6009
地方财政支出	万元	8468	16334	20456	31507	57107
农村居民人均纯收入	元	1147	1669	1837	2167	2667
城镇居民人均可支配收入	元		6182	6559	7740	9868
常用耕地面积	公顷	28055	26183	25096	24658	24397
粮食产量	吨	105318	123006	116447	91612	102045
农林牧渔业总产值	万元	45057	84277	95388	109681	140074
社会消费品零售总额	万元	19858	42684	49042	58052	73275
普通小学专任教师数	人	1954	1728	1667	1675	1606
普通小学在校学生数	人	44542	33376	31460	30940	29190
普通中学专任教师数	人	640	1002	1030	1034	1092
普通中学在校学生数	人	11417	15197	15875	16022	14995
卫生机构床位数	张	715	715	715	715	715
卫生技术人员	人	507	457	455	469	463
# 执业(助理)医师	人	310	376	384	402	416
注册护师、护士	人	90	104	112	119	121

?1994-2018 China Academic Journal Electronic Publishing House. All rights reserved.   http://www.cnki.net

社会主要指标

2009年	2010年	2011年	2012年	2013年	2014年	2015年	2016年
28.45	28.41	28.39	28.42	28.47	28.52	28.60	28.67
23.70	28.11	35.36	44.85	54.38	64.79	74.99	81.23
8.87	8.91	9.49	10.09	10.63	10.95	11.01	11.34
6.06	8.57	13.44	20.24	27.48	34.67	42.07	44.80
8.77	10.63	12.43	14.52	16.26	19.17	21.92	25.09
3.19	4.60	8.40	14.25	20.58	26.72	33.63	35.66
8208	9887	12452	15789	19116	22739	26260	28367
116.0	116.3	116.2	115.3	113.4	111.5	112.0	111.2
203329	289208	256773	346609	445444	561552	693510	848026
8036	10100	13308	18409	18526	21342	21306	16550
69063	112511	110864	155396	178088	192310	210080	227310
3267	4032	5112	5966	6849	7794	8051	8789
12159	14311	17130	20196	22337	24749	23575	25474
24262	24224	24277	24386	24370	24249	24259	24255
111747	117128	100798	106868	108375	109089	111311	111499
148769	153838	165342	175284	184765	193078	196564	201530
85791	96828	113902	133907	153215	176112	198923	226124
1548	1483	1429	1228	1068	1009	934	1133
27349	25357	23590	20972	20857	21213	21903	22409
1107	1128	1269	1220	1230	1236	1232	1036
14008	18000	17035	15382	14963	15054	14696	15067
715	795	596	619	699	834	821	859
466	483	625	1164	1376	1259	1224	1533
430	373	375	375	343	297	314	367
127	100	96	96	289	303	344	421

?1994-2018 China Academic Journal Electronic Publishing House. All rights reserved. http://www.cnki.net

12-7 岚皋县经济

指 标	单 位	2000年	2005年	2006年	2007年	2008年
年底总人口	万人	16.71	15.45	15.43	15.45	15.48
生产总值	亿元	3.80	6.81	7.87	8.88	11.48
第一产业	亿元	1.57	2.50	3.02	3.39	4.14
第二产业	亿元	0.82	1.85	1.99	2.10	2.86
第三产业	亿元	1.41	2.46	2.86	3.39	4.48
# 工业增加值	亿元	0.59	0.97	1.03	1.02	1.38
人均生产总值	元	2234	4025	5097	5751	7425
生产总值指数	上年=100	109.1	109.5	112.8	107.4	118.4
全社会固定资产投资	万元	19680	44920	47190	54326	102995
地方财政收入	万元	1918	1280	1428	1802	2346
地方财政支出	万元	6818	13218	15698	25920	37949
农村居民人均纯收入	元	1305	1810	1961	2152	2648
城镇居民人均可支配收入	元	5300	6310	6878	7722	9830
常用耕地面积	公顷	17941	15372	15299	15160	15485
粮食产量	吨	65696	77748	58891	55261	60920
农林牧渔业总产值	万元	25317	44222	51906	55261	71710
社会消费品零售总额	万元	10608	22638	25022	28112	33145
普通小学专任教师数	人	963	827	825	844	888
普通小学在校学生数	人	19000	13000	12000	11000	10000
普通中学专任教师数	人	445	440	455	467	491
普通中学在校学生数	人	7000	9000	9000	9000	9000
卫生机构床位数	张	439	464	464	468	468
卫生技术人员	人	385	410	425	440	441
# 执业(助理)医师	人	129	125	139	147	148
注册护师、护士	人	84	91	94	98	93

?1994-2018 China Academic Journal Electronic Publishing House. All rights reserved. http://www.cnki.net

社会主要指标

	2009年	2010年	2011年	2012年	2013年	2014年	2015年	2016年
	15.39	15.42	15.42	15.44	15.46	15.49	15.53	15.57
	13.29	16.31	19.81	25.16	30.96	35.11	40.26	45.66
	4.25	4.36	4.68	5.22	5.92	6.03	6.39	6.68
	3.80	6.14	8.61	12.39	16.56	19.08	22.47	25.84
	5.25	5.80	6.52	7.54	8.48	10.00	11.40	13.14
	2.10	3.87	5.82	9.12	12.87	14.90	17.82	20.78
	8583	10584	12844	16306	20037	22689	25954	29360
	116.3	116.7	115.2	115.1	114.0	111.5	113.3	111.7
	160112	207022	155095	203772	260476	329484	406749	532703
	3516	4516	5648	7628	9005	10192	10703	9508
	46856	79278	79249	98712	122156	128389	145268	165134
	3240	3936	4948	5755	6537	7348	7707	8374
	12549	14783	17552	20483	22982	25786	24020	26003
	15363	16141	16844	17168	17184	17180	16807	17050
	67080	69171	60598	64013	65142	65530	67290	67905
	74424	75573	81412	90850	102303	106117	112891	119031
	43753	46962	55067	64049	72773	83284	94441	107091
	859	784	681	627	591	564	553	606
	10000	9611	9857	9433	9374	9457	9865	10068
	497	514	560	577	569	553	561	521
	9000	8476	7997	7440	7334	7258	6998	7019
	483	463	510	587	674	772	773	778
	443	474	459	574	687	642	679	712
	152	110	131	130	216	178	157	187
	101	108	112	145	215	211	198	240

?1994-2018 China Academic Journal Electronic Publishing House. All rights reserved.   http://www.cnki.net

12-8 平利县经济

指 标	单 位	2000年	2005年	2006年	2007年	2008年
年底总人口	万人	22.85	19.07	19.09	19.11	19.15
生产总值	亿元	4.55	8.91	11.20	12.94	16.59
第一产业	亿元	2.25	3.60	4.29	4.90	6.39
第二产业	亿元	0.70	2.02	2.67	3.46	4.62
第三产业	亿元	1.60	3.30	4.24	4.58	5.58
# 工业增加值	亿元	0.46	1.31	1.85	2.37	3.08
人均生产总值	元	2401	3785	5425	6438	8254
生产总值指数	上年=100	107.8	110.4	114.4	114.1	116.5
全社会固定资产投资	万元	12224	43520	49448	78144	118761
地方财政收入	万元	2411	1742	1908	2599	3303
地方财政支出	万元	6779	14173	19046	28720	43269
农村居民人均纯收入	元	1146	1776	2085	2400	2914
城镇居民人均可支配收入	元	3688	6284	6868	7933	10031
常用耕地面积	公顷	21034	17623	18087	18249	18241
粮食产量	吨	79550	86780	94315	67901	72584
农林牧渔业总产值	万元	43488	63455	73821	90610	113174
社会消费品零售总额	万元	12728	26393	30223	35350	43999
普通小学专任教师数	人	1318	1174	1244	1154	1120
普通小学在校学生数	人	28263	18284	17543	17314	15756
普通中学专任教师数	人	463	612	686	703	697
普通中学在校学生数	人	29492	11385	12009	11044	10297
卫生机构床位数	张	264	309	358	364	433
卫生技术人员	人	380	450	468	436	464
# 执业(助理)医师	人	191	202	218	194	191
注册护师、护士	人	82	116	115	111	129

?1994-2018 China Academic Journal Electronic Publishing House. All rights reserved. http://www.cnki.net

社会主要指标

2009年	2010年	2011年	2012年	2013年	2014年	2015年	2016年
19.40	19.30	19.30	19.33	19.36	19.39	19.45	19.49
19.66	23.90	29.33	39.82	50.50	60.59	68.01	74.53
6.60	6.77	7.15	8.43	9.51	9.73	10.04	10.50
7.06	10.56	14.81	23.16	31.85	39.95	45.27	49.58
5.99	6.58	7.36	8.23	9.15	10.92	12.70	14.44
5.37	8.56	12.55	20.81	29.27	34.73	39.53	43.37
10258	12354	15191	20618	26108	31277	35026	38282
116.9	116.1	117.4	119.6	116.0	113.3	112.4	111.6
150145	189103	205904	283771	366426	512501	631695	631288
4529	5450	6860	9299	11252	12807	14245	13803
57361	82846	91805	122745	142723	161892	164530	184149
3544	4272	5362	6247	7165	8147	8130	8859
12340	14487	17159	20007	22408	25097	23810	25728
18305	18302	18149	18379	18372	18332	18287	18152
77037	79868	69502	73628	74041	74132	76898	77631
119266	122193	129839	150436	169512	176836	183856	192619
52694	59688	70063	81643	93507	107381	121163	137496
1102	1072	1051	999	993	971	971	948
14307	13034	12246	12331	12218	12182	12074	12552
742	773	827	825	823	836	732	739
8866	11674	11361	11632	10554	9586	8445	8531
486	492	522	561	570	680	771	920
502	640	604	712	808	761	849	878
186	183	224	248	275	244	267	252
131	171	186	225	267	261	303	337

?1994-2018 China Academic Journal Electronic Publishing House. All rights reserved. http://www.cnki.net

12-9 镇坪县经济

指　　标	单 位	2000年	2005年	2006年	2007年	2008年
年底总人口	万人	5.74	4.89	4.90	4.90	4.91
生产总值	亿元	1.41	2.79	3.17	3.84	4.93
第一产业	亿元	0.47	1.06	1.15	1.37	1.75
第二产业	亿元	0.33	0.63	0.72	0.90	1.24
第三产业	亿元	0.62	1.10	1.30	1.57	1.94
# 工业增加值	亿元	0.20	0.37	0.43	0.57	0.79
人均生产总值	元	2471	5528	6468	7653	10034
生产总值指数	上年=100	109.7	109.7	111.4	113.9	114.8
全社会固定资产投资	万元	6550	17682	20846	27827	39724
地方财政收入	万元	800	760	872	1759	1700
地方财政支出	万元	3326	6524	8744	12142	18480
农村居民人均纯收入	元	1242	1777	1901	2183	2646
城镇居民人均可支配收入	元	4981	6476	7096	7988	10069
常用耕地面积	公顷	5981	4874	4875	4893	4920
粮食产量	吨	25271	28393	29631	23454	25893
农林牧渔业总产值	万元	8281	18481	20010	24050	32777
社会消费品零售总额	万元	5283	7797	8362	9771	12013
普通小学专任教师数	人	427	439	448	438	440
普通小学在校学生数	人	6400	4800	4900	4700	4500
普通中学专任教师数	人	141	173	190	194	219
普通中学在校学生数	人	2600	3400	3300	3400	3500
卫生机构床位数	张	136	164	164	153	156
卫生技术人员	人	148	159	169	170	171
# 执业(助理)医师	人	110	82	90	70	71
注册护师、护士	人	34	43	43	39	41

?1994-2018 China Academic Journal Electronic Publishing House. All rights reserved.   http://www.cnki.net

社会主要指标

2009年	2010年	2011年	2012年	2013年	2014年	2015年	2016年
5.13	5.10	5.10	5.11	5.11	5.12	5.14	5.15
5.71	6.81	8.70	11.74	12.95	12.06	13.45	15.25
1.81	1.93	2.10	2.55	2.87	2.98	3.11	3.22
1.56	2.07	3.24	5.27	5.75	4.09	4.58	5.41
2.33	2.81	3.37	3.92	4.33	4.99	5.76	6.62
1.06	1.49	2.52	4.41	4.74	2.72	3.03	3.70
11607	13324	17060	23007	25339	23559	26215	29644
115.6	114.4	115.6	115.0	110.2	108.3	113.3	110.6
51004	65328	65480	90027	115127	131977	154604	208175
2200	2640	3308	4381	5253	5944	6151	6069
25708	34632	39349	58281	68354	69333	74866	88460
3215	3835	4828	5673	6456	7360	7728	8413
12356	14395	17154	20207	22410	24853	23620	25546
4927	4931	4936	4943	4948	4966	4965	4981
28780	30393	26047	27180	27363	27056	27534	27871
34334	35716	38838	46773	52640	55179	57384	59927
14380	17048	19999	23296	26269	28852	32696	45159
425	419	335	287	273	271	269	284
4400	4314	4199	3997	3764	3600	3746	3904
222	223	220	225	241	238	239	225
3600	3616	3626	3708	3135	2870	2721	2682
242	171	244	262	260	264	283	292
199	192	188	189	374	335	379	385
52	70	53	61	111	88	101	102
51	50	50	55	118	120	128	146

?1994-2018 China Academic Journal Electronic Publishing House. All rights reserved. http://www.cnki.net

12-10 旬阳县经济

指 标	单 位	2000年	2005年	2006年	2007年	2008年
年底总人口	万人	44.81	42.69	42.72	42.76	42.83
生产总值	亿元	13.01	22.35	25.79	32.78	41.13
第一产业	亿元	3.75	4.81	5.36	6.10	8.79
第二产业	亿元	4.68	7.72	9.43	13.90	17.55
第三产业	亿元	4.58	9.82	11.01	12.78	14.79
# 工业增加值	亿元	3.33	5.80	7.01	10.89	13.13
人均生产总值	元	2911	4974	6071	7632	9459
生产总值指数	上年=100	115.0	109.8	111.5	113.7	115.1
全社会固定资产投资	万元	35897	85075	116484	160189	284550
地方财政收入	万元	6124	7180	6661	8618	12102
地方财政支出	万元	12859	26920	33437	43605	72518
农村居民人均纯收入	元	1217	1859	1980	2298	2819
城镇居民人均可支配收入	元	4456	6618	6940	8061	10223
常用耕地面积	公顷	44610	34308	34672	35108	35451
粮食产量	吨	171211	143276	137294	106984	113638
农林牧渔业总产值	万元	64479	81835	91152	105597	151126
社会消费品零售总额	万元	29374	66165	76216	89699	112314
普通小学专任教师数	人	2005	2181	2162	2135	1960
普通小学在校学生数	人	54500	40600	37100	32500	28900
普通中学专任教师数	人	1247	1611	1697	1763	1757
普通中学在校学生数	人	26900	34700	36500	37700	36900
卫生机构床位数	张	515	599	731	745	827
卫生技术人员	人	902	824	857	930	928
# 执业(助理)医师	人	471	469	473	540	477
注册护师、护士	人	185	195	200	215	223

?1994-2018 China Academic Journal Electronic Publishing House. All rights reserved. http://www.cnki.net

社会主要指标

2009年	2010年	2011年	2012年	2013年	2014年	2015年	2016年
42.73	42.68	42.68	42.73	42.79	42.87	43.00	43.09
47.02	55.44	67.77	81.74	100.01	112.37	120.96	135.75
8.99	9.19	9.67	10.50	11.79	12.09	12.44	13.00
20.33	24.74	34.11	44.05	58.40	65.23	67.95	75.95
17.70	21.51	24.00	27.20	29.83	35.05	40.57	46.80
13.12	17.14	24.99	33.97	47.07	52.42	54.28	61.00
10971	12983	15879	19140	23388	26236	28176	31538
113.6	113.6	115.1	114.6	113.5	111.9	111.6	111.0
400006	412773	448665	607197	770025	963669	1162710	1425012
16388	21102	28524	39292	46406	46460	42197	40505
98640	133367	176552	212079	243039	258860	250293	296845
3357	4017	5096	5911	6757	7655	7900	8608
12690	14809	17697	20617	22741	25174	23907	25810
35539	35709	35891	36208	36361	36410	36542	36650
123465	127488	111346	120091	122649	123708	125482	126092
156766	159372	170433	190068	212778	222456	229561	241290
134410	149606	174526	203948	231488	267014	307869	398050
1916	1852	1782	1701	1628	1399	1375	1421
26200	24618	23366	22287	21857	22087	22786	23276
1769	1740	1865	1858	1854	1834	1789	1699
36200	30299	27315	24223	21826	20393	18918	18610
905	1006	1042	1275	1526	1875	2078	2243
1177	1283	1523	1668	1817	1759	2048	2359
390	388	548	563	603	492	614	611
370	422	462	563	660	718	832	991

?1994-2018 China Academic Journal Electronic Publishing House. All rights reserved. http://www.cnki.net

12-11 白河县经济

指　　标	单 位	2000年	2005年	2006年	2007年	2008年
年底总人口	万人	20.67	16.43	16.41	16.43	16.46
生产总值	亿元	3.90	7.91	9.62	11.67	13.70
第一产业	亿元	1.74	2.83	3.25	3.75	4.81
第二产业	亿元	0.58	1.59	2.25	3.28	3.71
第三产业	亿元	1.57	3.49	4.12	4.64	5.18
# 工业增加值	亿元	0.34	1.00	1.54	2.39	2.67
人均生产总值	元	1890	3822	5856	7105	8330
生产总值指数	上年=100	111.6	110.4	112.6	113.0	114.3
全社会固定资产投资	万元	8446	36998	37935	62482	95086
地方财政收入	万元	2009	1689	2178	3208	3828
地方财政支出	万元	6556	13355	17578	25219	38088
农村居民人均纯收入	元	1297	1786	1901	2221	2739
城镇居民人均可支配收入	元		6466	6875	7903	10028
常用耕地面积	公顷	19577	13772	13801	13801	13801
粮食产量	吨	80107	55782	63681	50604	55238
农林牧渔业总产值	万元	30597	46737	53655	62139	80387
社会消费品零售总额	万元	13594	25823	29574	34835	43820
普通小学专任教师数	人	1060	1248	1262	1237	1216
普通小学在校学生数	人	29449	23178	21150	18842	16553
普通中学专任教师数	人	428	938	966	1031	1072
普通中学在校学生数	人	8012	15299	16033	16723	16536
卫生机构床位数	张	284	297	306	306	364
卫生技术人员	人	269	258	254	314	319
# 执业(助理)医师	人	188	185	142	135	147
注册护师、护士	人	62	64	69	75	89

?1994-2018 China Academic Journal Electronic Publishing House. All rights reserved.　http://www.cnki.net

社会主要指标

2009年	2010年	2011年	2012年	2013年	2014年	2015年	2016年
16.51	16.35	16.35	16.36	16.39	16.42	16.47	16.50
16.44	20.04	25.49	32.70	41.56	48.27	52.70	58.18
4.92	5.02	5.42	6.00	6.83	7.00	7.35	7.59
4.91	7.44	11.54	17.12	24.17	29.17	31.16	34.32
6.61	7.58	8.53	9.59	10.56	12.11	14.19	16.27
3.61	5.75	9.33	14.70	21.58	25.86	27.51	30.36
9977	12201	15594	19997	25378	29428	32052	35293
116.9	114.1	116.1	116.6	115.0	113.9	113.4	111.4
145157	202874	181184	244831	318030	396580	488385	600453
4519	5428	7088	9725	11520	13026	13646	11608
54800	77488	88511	124000	134567	142285	165400	167503
3302	3980	5007	5798	6592	7416	7768	8417
12316	14410	17033	19980	22178	24617	23432	25485
13801	13806	13840	13840	13895	13885	14097	14097
58174	60356	52597	56326	56706	57364	58180	58376
82990	85398	92371	101752	115437	120819	126822	131233
51665	57654	67017	78783	89812	103387	118363	134220
1153	1117	1004	908	775	715	707	737
14655	13790	12776	11445	11109	11809	12428	13081
1111	1169	1113	1119	985	777	840	793
16633	16480	15286	12798	10566	9754	9198	9087
384	384	388	532	513	600	671	749
342	377	406	778	749	680	764	776
159	160	179	216	237	213	241	248
102	109	129	158	236	234	268	270

?1994-2018 China Academic Journal Electronic Publishing House. All rights reserved. http://www.cnki.net
