# Supplementary material for: A study on the tourism efficiency of tourism destination based on DEA model: A case of ten cities in Shaanxi province
Source: PLoS One. 2024 Jan 19;19(1):e0296660. doi: 10.1371/journal.pone.0296660 (PMC10798521; doi:10.1371/journal.pone.0296660)
Supplement: S1 File — (ZIP) [file pone.0296660.s001.zip › Supporting information/Statistical yearbook/Baoji.caj]

六、宝鸡市

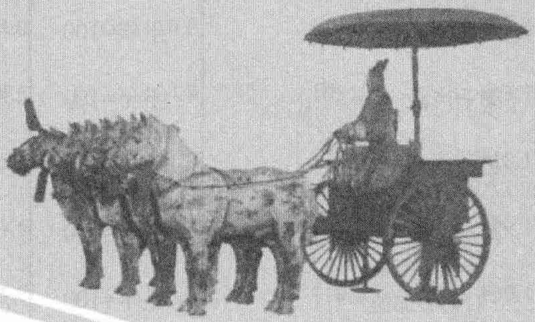

资料整理：徐　刚　李雯佳

?1994-2018 China Academic Journal Electronic Publishing House. All rights reserved.　http://www.cnki.net

6-1 宝鸡市经济

指　　标	单 位	2000年	2005年	2006年	2007年	2008年
年底总人口	万人	363.38	376.39	375.86	375.70	376.25
人口自然增长率	‰		4.33	4.14	4.21	4.22
年底总户数	万户	96.11	104.02	106.07	108.16	109.05
生产总值	亿元	195.34	414.52	490.31	578.78	714.07
第一产业	亿元	25.18	44.30	49.70	60.86	78.30
第二产业	亿元	98.32	240.13	293.45	345.91	434.70
第三产业	亿元	71.84	130.09	147.16	172.01	201.07
# 工业增加值	亿元	78.89	194.72	240.15	281.43	351.59
人均生产总值	元	5425	11103	13082	15402	19071
生产总值指数	上年=100	110.5	113.0	113.1	114.8	115.5
第一产业	上年=100	100.6	110.2	108.0	104.4	107.4
第二产业	上年=100	112.4	116.1	116.4	117.9	118.3
第三产业	上年=100	111.1	108.9	108.8	112.2	112.3
# 工业增加值	上年=100	112.2	117.2	117.2	118.7	119.2
人均生产总值指数	上年=100	109.1	113.1	112.7	114.5	115.9
非公有制经济增加值	亿元		183.72	218.68	262.90	332.43
文化产业增加值	亿元					
单位GDP能耗	吨标准煤/万元		1.460	1.399	1.328	1.249
单位GDP能耗比上年增长	%			-4.20	-5.05	-5.96
就业人员	万人	201.65	205.78	203.98	192.02	189.79
城镇单位就业人员	万人	34.50	30.22	29.87	29.93	29.16
# 国有单位	万人	28.89	22.23	21.85	20.40	19.94
集体单位	万人	3.86	2.41	2.49	2.75	2.40
# 在岗职工人数	万人	33.62	29.27	29.18	29.17	28.40
城镇单位就业人员平均工资	元					
城镇单位在岗职工平均工资	元	7326	12846	14777	18389	22442

?1994-2018 China Academic Journal Electronic Publishing House. All rights reserved.　http://www.cnki.net

社会主要指标

2009年	2010年	2011年	2012年	2013年	2014年	2015年	2016年
371.07	371.93	372.72	373.67	374.46	375.32	376.33	377.50
4.17	3.34	3.35	3.48	3.61	3.55	3.54	3.78
110.78	112.02	113.25	114.57	115.33	115.02	115.64	116.16
806.54	976.09	1175.75	1374.33	1545.91	1642.90	1787.63	1932.14
85.18	104.20	128.56	143.26	157.65	161.33	165.13	171.46
491.08	614.42	749.25	895.92	1007.72	1051.65	1141.43	1227.06
230.28	257.47	297.94	335.15	380.54	429.92	481.07	533.62
391.92	497.40	610.32	735.89	821.52	838.98	900.05	952.37
21525	26274	31579	36826	41327	43824	47565	51262
115.0	114.4	114.5	115.1	113.0	110.8	110.5	109.3
106.2	106.9	106.1	105.7	104.5	104.9	105.4	103.7
116.7	117.5	117.5	118.5	115.3	111.9	111.4	110.1
113.9	109.4	111.0	109.9	109.5	109.4	109.3	109.1
116.0	118.6	119.0	120.0	115.7	111.8	110.6	109.1
115.4	114.0	114.3	114.8	112.7	110.6	110.1	108.9
384.28	471.30	578.48	681.17	773.29	822.60	895.61	974.65
					31.61	34.77	39.27
1.186	0.734	0.708	0.682	0.658	0.631	0.608	0.517
-5.06	-2.01	-3.53	-3.64	-3.53	-4.10	-3.59	-4.41
186.80	203.70	208.90	211.05	205.90	215.90	220.29	226.42
29.08	29.83	30.12	30.21	39.07	40.23	41.00	41.13
17.76	18.45	19.54	17.65	16.26	16.29	16.32	15.74
1.54	1.28	1.45	1.64	1.65	1.67	1.55	2.43
28.38	28.83	29.56	29.45	37.69	38.89	39.61	39.51
			38546	40257	43603	47886	51475
26534	30943	35063	39025	40983	44270	48557	52351

?1994-2018 China Academic Journal Electronic Publishing House. All rights reserved.   http://www.cnki.net

6-1 续表 1

指 标	单 位	2000年	2005年	2006年	2007年	2008年
全社会固定资产投资	亿元	69.21	186.86	224.38	316.21	451.03
# 房地产开发	亿元	5.97	22.09	27.73	38.94	59.50
商品房销售面积	万平方米	51.93	84.78	109.23	122.35	146.55
# 住宅	万平方米	48.37	83.54	108.19	118.16	138.01
地方财政收入	亿元	8.17	11.97	15.07	19.60	23.91
地方财政支出	亿元	13.31	26.45	36.91	53.56	75.27
金融机构人民币各项存款余额	亿元	229.55	459.50	552.92	611.58	737.11
金融机构人民币各项贷款余额	亿元	189.77	212.35	231.47	259.93	272.61
农村居民人均纯收入	元	1543	2233	2454	2888	3500
城镇居民人均可支配收入	元	4754	8540	9424	10993	13225
城市人均公园绿地面积	平方米			9.5	10.5	12.5
城市人均道路面积	平方米		7.8	14.8	15.4	15.6
城市用水普及率	%		98.3	98.6	99.8	99.8
城市燃气普及率	%		81.4	95.0	95.7	98.3
常用耕地面积	千公顷	333.84	305.05	306.62	311.16	311.53
农林牧渔业总产值	亿元	44.84	75.08	83.96	103.57	133.90
农作物总播种面积	千公顷	483.60	465.39	472.60	430.54	441.84
# 粮食作物	千公顷	414.56	384.11	387.69	345.90	357.50
粮食产量	万吨	132.64	150.56	165.91	135.89	160.93
棉花产量	吨	162	129	186	128	109
油料产量	吨	24566	19458	15618	16659	22198
蔬菜产量	吨	547903	782065	844280	857294	936447

?1994-2018 China Academic Journal Electronic Publishing House. All rights reserved.   http://www.cnki.net

2009年	2010年	2011年	2012年	2013年	2014年	2015年	2016年
639.14	835.22	1008.03	1311.69	1669.78	2105.59	2589.88	3199.84
58.49	63.70	72.71	75.88	81.27	93.14	94.78	132.41
149.05	198.37	211.78	220.53	242.72	263.19	262.40	287.76
140.76	193.35	205.10	209.57	231.62	257.13	249.19	269.60
30.14	38.78	51.44	64.85	72.02	78.06	84.47	75.16
102.21	133.06	163.74	203.53	220.39	235.60	264.07	283.04
912.84	1076.77	1220.82	1450.84	1647.11	1834.14	2092.10	2364.41
358.15	436.95	519.84	633.10	758.51	884.69	1039.92	1182.76
4186	5040	6340	7373	8376	8686	9511	10287
16346	18978	22337	25777	28509	27161	29475	31730
14.2	14.2	14.1	13.3	12.3	12.3	12.3	12.3
15.2	15.7	15.5	14.6	16.0	15.9	16.0	15.9
99.8	99.9	99.9	100.0	100.0	100.0	100.0	91.7
98.4	98.6	98.7	98.8	98.8	98.6	99.1	97.9
308.99	306.78	305.72	299.96	299.99	298.36	297.31	295.95
141.14	174.65	215.72	239.97	263.99	280.90	289.89	301.55
443.59	444.77	420.34	420.00	419.21	412.33	411.55	404.64
358.58	357.54	335.39	337.49	337.52	332.97	332.23	327.21
166.99	171.47	142.52	153.59	145.59	144.32	149.65	145.67
177	151	89	103	95	60	52	47
22552	22552	22627	22002	17736	19236	19205	17993
972028	1075344	1126056	1200967	1291600	1363600	1433844	1479809

?1994-2018 China Academic Journal Electronic Publishing House. All rights reserved. http://www.cnki.net

6-1 续表 2

指 标	单 位	2000年	2005年	2006年	2007年	2008年
水果产量	吨	382278	513314	571210	605063	684252
# 苹果产量	吨	289921	310429	333103	354621	391571
肉类产量	吨	99854	166264	182678	98633	114720
# 猪牛羊肉产量	吨	89679	144789	156242	85718	98416
奶类产量	吨	120338	375561	434778	490705	506784
# 牛奶	吨	87072	332789	371734	432436	458440
禽蛋产量	吨	48419	77584	66632	54273	61729
水产品产量	吨	4912	7567	7846	1858	2007
规模以上工业企业单位数	个	293	375	408	413	432
规模以上工业总产值	亿元	139.98	409.95	536.67	672.20	895.77
纱产量	万吨	1.91	4.91	4.89	5.60	5.55
布产量	万米	9062	15464	15254	19064	24482
原煤产量	万吨	32.56	73.22	125.50	145.08	149.36
发电量	亿千瓦小时	21.69	81.53	77.62	80.38	78.05
粗钢产量	万吨	2.65	3.41	5.87	8.37	4.95
钢材产量	万吨	17.76	121.82	145.49	128.86	130.53
水泥产量	万吨	184.01	563.91	642.98	738.06	747.36
汽车产量	辆	2288	11112	9737	6092	9697
建筑业企业单位数	个	122	76	71	71	70
建筑业企业年末从业人员	万人	6.06	4.99	10.81	7.69	10.05
建筑业总产值	亿元	25.00	56.69	92.95	128.04	175.85
房屋建筑施工面积	万平方米	371.83	603.02	852.77	967.45	1169.42
房屋建筑竣工面积	万平方米	193.16	284.03	422.56	259.91	361.26
公路里程	公里	3883	4989	7760	12237	12398
# 等级公路	公里	3647	4746	5548	10170	11240
# 高速公路	公里	82	80	80	80	80
民用汽车拥有量	辆	29208	52983	61445	71079	80770
# 私人汽车	辆	11926	23258	32319	38741	45963
邮电业务总量	亿元	6.89	24.40	31.04	37.14	44.37
邮政业务总量	亿元	0.52	1.84	2.45	2.56	2.93
电信业务总量	亿元	6.38	22.56	28.59	34.58	41.43

?1994-2018 China Academic Journal Electronic Publishing House. All rights reserved. http://www.cnki.net

2009年	2010年	2011年	2012年	2013年	2014年	2015年	2016年
864130	969353	1185605	1227535	1260314	1319702	1408899	1453325
507081	534139	639827	660138	663711	680618	734682	749045
133622	150041	164944	174145	181267	186692	185334	182016
119636	134505	144174	150725	157033	162980	160515	159359
564667	597763	612834	620903	655764	662356	650081	642119
494804	522537	545959	552757	585890	597891	585625	576569
66444	72358	74969	72817	75926	74994	75769	76383
2106	6870	6932	7077	7173	7467	7616	7747
513	501	386	443	454	536	595	662
995.99	1340.45	1701.80	1986.69	2258.52	2274.97	2599.43	2929.65
6.53	7.01	8.28	8.06	10.20	11.98	14.95	12.87
18099	19683	16005	22293	17302	24209	32493	40288
175.80	254.93	463.68	1048.00	721.50	651.70	857.50	814.45
61.87	84.99	111.85	128.82	131.60	135.80	144.17	143.34
2.78	3.01	3.25	1.58	6.24	14.31	20.70	10.26
222.97	219.43	267.79	402.76	413.00	438.85	536.50	182.90
950.98	901.81	1097.07	1044.00	1123.90	1215.78	1067.60	986.40
20558	23699	16069	11705	5061	5085	7329	50206
79	76	100	119	132	146	145	149
12.47	14.04	6.48	5.50	7.13	6.63	9.15	13.89
239.82	264.17	253.21	277.70	351.22	433.53	548.21	637.03
935.95	1997.36	1565.20	1757.00	2094.11	2152.05	2415.84	2433.30
334.98	617.35	491.89	583.68	733.64	806.56	945.28	834.28
14102	14255	14676	15003	15898	15936	16108	16328
13355	13861	14284	14507	14554	14642	14819	15055
132	199	231	231	231	241	259	258
104316	130893	149601	170043	191185	204682	239326	279509
65623	93891	109588	127477	149325	165958	199863	241197
48.45	19.61	25.42	27.26	30.14	40.27	56.26	82.20
3.24	2.60	2.88	2.45	3.56	3.76	4.55	6.25
45.21	17.02	22.54	24.81	26.58	36.51	51.71	75.95

?1994-2018 China Academic Journal Electronic Publishing House. All rights reserved. http://www.cnki.net

6-1 续表 3

指 标	单 位	2000年	2005年	2006年	2007年	2008年
固定电话用户	万户	37.74	95.94	98.85	94.70	84.12
移动电话用户	万户	13.87	68.52	81.79	125.78	151.02
互联网宽带用户	万户	1.91	15.38	11.79	15.81	21.41
限额以上企业数	个					
批发业	个					
零售业	个					
住宿业	个					
餐饮业	个					
社会消费品零售总额	亿元	71.89	137.48	155.23	179.81	220.90
进出口总额	万美元		14721	62176	69825	46279
# 出口	万美元		6984	41947	51915	26022
实际外商直接投资额	万美元		60	827	1339	3379
入境旅游人数	万人次	1.83	3.50	4.20	5.40	9.00
# 外国人	万人次	1.09	2.30	3.10	4.10	4.00
国际旅游外汇收入	万美元	525	1113	1474	1975	1909
国内旅游人数	万人次	409	715	820	940	1085
国内旅游收入	亿元	12.40	28.90	40.20	54.50	65.50
星级饭店数	个		33	33	35	35
幼儿园数	所	104	126	161	158	167
在园儿童数	万人	7.28	5.59	5.57	5.46	5.27
普通小学学校数	所	2044	1753	1702	1496	1224
普通小学专任教师数	人	15984	16724	16686	16416	16510
普通小学在校学生数	万人	47.92	32.84	31.32	29.12	26.96
普通中学学校数	所	266	279	275	272	257
普通中学专任教师数	人	14273	17317	17491	17529	17600
普通中学在校学生数	万人	26.14	31.01	30.74	29.65	28.30
卫生机构数	个	581	677	703	626	594
卫生机构床位数	张	10339	11995	13093	13811	14267
卫生技术人员	人	14366	15152	14814	14844	15043
# 执业(助理)医师	人	6764	6589	6043	6102	5455
注册护师、护士	人	3769	4125	4236	4321	4692

?1994-2018 China Academic Journal Electronic Publishing House. All rights reserved. http://www.cnki.net

2009年	2010年	2011年	2012年	2013年	2014年	2015年	2016年
79.66	77.24	74.24	70.96	69.36	64.91	64.76	61.32
178.21	194.61	224.69	242.34	250.24	266.46	295.45	300.61
23.09	28.60	30.61	34.45	40.54	42.31	50.17	67.33
157	179	219	327	538	608	696	800
11	16	18	34	73	85	92	106
58	69	91	162	294	341	395	463
37	39	45	47	69	74	82	87
51	55	65	84	102	108	127	144
260.10	307.52	358.23	412.83	473.39	539.67	612.84	702.16
45724	59925	82268	74527	88740	52.43(亿元)	53.61(亿元)	63.61(亿元)
17443	26444	49899	55235	69833	41.21(亿元)	40.85(亿元)	45.53(亿元)
3610	2127	5020	6015	7006	8008	268	
13.00	16.10	20.30	25.30	30.02	32.00	33.20	33.80
6.67	8.30	10.20	12.57	14.00	14.76	15.30	15.67
3502	4648	5712	7320	8685	9152	9406	9514
1297	1659	2030	2737	3595	4680	5356	6351
78.00	100.21	132.00	171.40	236.40	310.80	363.40	436.38
36	37	37	36	36	34	34	28
174	201	281	337	400	425	469	487
5.38	5.69	7.72	9.22	10.17	10.67	11.19	11.25
1081	918	796	757	713	633	526	505
16220	15876	14954	14216	13644	13364	12761	12248
25.02	23.16	22.67	20.40	19.76	19.36	19.36	19.59
246	236	219	217	216	212	205	202
17460	17423	18289	17576	18237	17683	16504	16944
26.44	24.98	23.59	21.37	20.03	19.10	18.14	17.53
575	571	2788	2816	2917	2916	2999	3024
15872	15913	17211	18498	19731	21478	23091	24506
15516	16159	18031	19583	21137	22784	24460	27307
5596	5766	6630	6911	7255	7554	7924	8858
5006	5248	6151	6796	7654	8388	9324	10612

?1994-2018 China Academic Journal Electronic Publishing House. All rights reserved.   http://www.cnki.net

6-2 渭滨区经济

指 标	单 位	2000年	2005年	2006年	2007年	2008年
年底总人口	万人	31.78	39.52	39.17	40.30	40.45
生产总值	亿元	44.30	102.74	120.68	157.60	189.15
第一产业	亿元	0.70	1.23	1.36	1.54	2.09
第二产业	亿元	25.90	64.62	77.53	107.45	130.18
第三产业	亿元	17.70	36.89	41.79	48.61	56.88
# 工业增加值	亿元	13.06	46.01	57.00	82.98	99.95
人均生产总值	元	13939	26254	30172	38414	46848
生产总值指数	上年=100	112.4	116.9	115.8	117.2	126.5
全社会固定资产投资	万元	19359	86987	115257	195067	315592
地方财政收入	万元	8663	9758	11338	15021	18800
地方财政支出	万元	8831	18028	24124	34734	46945
农村居民人均纯收入	元	2390	2818	3109	3700	4511
城镇居民人均可支配收入	元	5341	8592	9451	10993	13484
常用耕地面积	公顷	6232	6580	6468	6310	6564
粮食产量	吨	22080	31004	32191	23963	27757
农林牧渔业总产值	万元	12363	24353	26260	28117	34138
社会消费品零售总额	万元	114912	230553	270772	333284	409987
普通小学专任教师数	人	1605	1798	1777	1750	1733
普通小学在校学生数	人	27800	29000	29000	29000	29000
普通中学专任教师数	人	1388	1928	1985	2029	2009
普通中学在校学生数	人	20000	30000	30000	30000	29999
卫生机构床位数	张	2232	2863	2921	3190	3248
卫生技术人员	人	2566	2256	2707	3462	3573
# 执业(助理)医师	人	907	832	1007	1368	1373
注册护师、护士	人	941	850	1011	1290	1305

?1994-2018 China Academic Journal Electronic Publishing House. All rights reserved. http://www.cnki.net

社会主要指标

2009年	2010年	2011年	2012年	2013年	2014年	2015年	2016年
44.75	44.85	44.94	45.06	45.15	45.26	45.38	45.54
220.04	253.90	343.81	407.16	427.25	445.70	471.41	500.49
2.20	2.68	3.12	3.56	3.92	4.01	4.04	4.03
158.62	183.29	261.84	315.18	325.48	323.44	342.86	356.85
59.22	67.94	78.84	88.42	97.85	118.25	124.51	139.62
119.56	137.09	204.61	254.23	257.10	242.86	241.40	240.38
54271	57372	78045	90476	94720	93148	103700	110093
118.0	114.5	116.8	115.1	112.1	108.5	115.0	107.3
523431	806004	1172002	2824639	2628639	3343372	4079719	4964300
23309	27628	33207	38960	45216	51757	56738	56847
60113	72052	84366	110783	122768	132242	157452	182538
5679	6928	8785	10208	11596	13069	11587	12514
16612	19203	22736	26465	29323	32519	31598	34031
6567	6626	6523	6504	6389	5969	5934	5565
30459	34097	21460	26527	22000	20000	19896	15866
38291	46992	55170	63308	69557	74374	77360	77834
836062	986397	1189056	1181110	1379329	1749607	2001551	2333330
1779	1799	1720	1345	1225	1309	1276	1712
28421	28471	29096	21542	21385	21235	21259	31082
2045	2109	2431	1842	1530	1548	1521	2244
30454	48234	32133	22415	22579	21773	20627	30174
3898	3797	4444	5016	4832	5383	6622	7126
3787	4444	4500	4984	5157	5341	6676	7307
1441	1413	1633	1860	1867	1906	2372	2559
1436	1446	1863	1965	2249	2325	2960	3292

?1994-2018 China Academic Journal Electronic Publishing House. All rights reserved. http://www.cnki.net

6-3 金台区经济

指标	单位	2000年	2005年	2006年	2007年	2008年
年底总人口	万人	26.20	37.90	37.65	37.87	37.87
生产总值	亿元	35.67	83.54	96.54	110.72	130.72
第一产业	亿元	0.31	0.75	0.81	0.97	1.36
第二产业	亿元	20.16	55.47	64.32	73.86	85.43
第三产业	亿元	15.20	27.32	31.41	35.90	43.93
# 工业增加值	亿元	16.40	48.09	56.55	63.49	72.47
人均生产总值	元	10254	22792	26155	29684	34829
生产总值指数	上年=100	109.9	124.7	115.6	114.7	118.1
全社会固定资产投资	万元	15060	220300	330954	403000	584000
地方财政收入	万元	5542	6936	8642	10759	13331
地方财政支出	万元	6650	15124	20911	31040	43275
农村居民人均纯收入	元	2821	2486	2800	3269	4037
城镇居民人均可支配收入	元	5341	8592	9451	10993	13236
常用耕地面积	公顷	1316	9762	9826	10204	9997
粮食产量	吨	3824	32386	34486	28589	37211
农林牧渔业总产值	万元	5049	12551	13605	15680	20046
社会消费品零售总额	万元	92088	162464	217753	278772	345000
普通小学专任教师数	人	1050	1325	1359	1393	1378
普通小学在校学生数	人	21600	27500	27400	27000	26600
普通中学专任教师数	人	1018	1684	1674	1708	1672
普通中学在校学生数	人	18100	29100	29000	29100	28000
卫生机构床位数	张	1735	2216	2306	2312	2031
卫生技术人员	人	2339	3969	4023	2378	2672
# 执业(助理)医师	人	866	821	830	871	870
注册护师、护士	人	183	301	320	350	356

?1994-2018 China Academic Journal Electronic Publishing House. All rights reserved. http://www.cnki.net

社会主要指标

2009年	2010年	2011年	2012年	2013年	2014年	2015年	2016年
39.39	39.48	39.56	39.66	39.75	39.84	39.94	40.05
151.80	174.16	204.60	241.25	275.73	295.45	304.04	338.50
1.43	1.77	2.14	2.36	2.57	2.56	2.64	2.80
99.05	114.02	134.46	162.41	188.45	196.04	203.62	225.34
51.32	58.36	68.00	76.48	84.70	96.85	97.78	110.37
83.25	95.36	111.31	135.37	157.43	157.25	161.16	173.48
40079	45663	51771	60905	69447	70711	76267	84635
116.1	114.7	115.7	114.6	114.7	112.0	110.6	109.8
472974	629313	1005100	1600925	2232390	2876062	3561032	4445300
17035	22201	29109	35635	41905	47402	51409	47080
60777	70063	82453	103257	116692	126698	140635	168255
5054	6186	7893	9290	10581	11883	10523	11365
16307	18835	22112	25473	28224	31329	30485	32832
10050	10499	10797	10748	10613	10176	10122	10199
42733	46774	31238	36787	34114	33019	36866	35591
22077	28418	34273	37857	41236	43570	45197	47906
532423	629856	736301	1200700	1351437	1373046	1548666	1772906
1370	1355	1409	1328	1402	1391	1412	1390
26300	25154	25293	24188	23736	23722	24068	24580
1708	1719	1350	1667	1729	1742	1792	1766
27300	26114	25138	23503	22938	23045	23381	22535
2734	2563	2940	2846	3110	3474	3300	3412
2917	2937	3174	3408	3663	3872	3704	4184
879	1217	1364	1394	1485	1498	1430	1699
360	478	511	1313	1437	1588	1566	1728

?1994-2018 China Academic Journal Electronic Publishing House. All rights reserved. http://www.cnki.net

6-4 陈仓区经济

指 标	单 位	2000年	2005年	2006年	2007年	2008年
年底总人口	万人	72.40	60.60	60.86	59.97	60.07
生产总值	亿元	24.02	51.77	60.66	73.13	87.48
第一产业	亿元	4.37	6.52	7.72	9.71	13.17
第二产业	亿元	11.66	29.73	34.62	42.05	49.15
第三产业	亿元	7.99	15.52	18.32	21.37	25.16
# 工业增加值	亿元	9.81	24.45	28.79	35.16	39.81
人均生产总值	元	3338	8548	9988	12105	14575
生产总值指数	上年=100	108.2	116.8	115.8	116.8	114.5
全社会固定资产投资	万元	50100	183900	238000	329300	468600
地方财政收入	万元	8572	8786	11104	13925	16769
地方财政支出	万元	11991	20653	31934	51674	100892
农村居民人均纯收入	元	1480	2524	2910	3548	4100
城镇居民人均可支配收入	元	4328	6589	7971	9526	12951
常用耕地面积	公顷	66535	44467	44475	44885	44833
粮食产量	吨	239085	234994	260829	271868	252532
农林牧渔业总产值	万元	73572	119135	141103	174479	234746
社会消费品零售总额	万元	75600	131900	162200	197800	238400
普通小学专任教师数	人	3022	2448	2523	2492	2665
普通小学在校学生数	人	101509	53887	50242	44915	40727
普通中学专任教师数	人	3040	3058	3104	3115	3068
普通中学在校学生数	人	50246	49044	47730	45597	42497
卫生机构床位数	张	1278	1309	1557	1557	1572
卫生技术人员	人	1566	1154	1071	1656	1282
# 执业(助理)医师	人	627	530	510	488	490
注册护师、护士	人	310	324	306	312	320

?1994-2018 China Academic Journal Electronic Publishing House. All rights reserved. http://www.cnki.net

社会主要指标

2009年	2010年	2011年	2012年	2013年	2014年	2015年	2016年
59.41	59.55	59.67	59.83	59.95	60.09	60.25	60.40
101.60	114.83	134.91	151.54	162.91	173.64	147.72	164.78
13.89	15.42	19.30	21.54	23.72	23.92	24.63	25.73
58.06	65.26	75.85	85.34	89.93	94.60	81.43	91.93
29.65	34.15	39.76	44.66	49.26	55.11	41.66	47.12
46.34	51.44	58.76	66.71	68.69	69.25	52.92	60.22
16900	19069	22631	25361	27200	23851	24550	27315
115.5	114.8	114.5	113.3	112.3	108.8	108.5	110.0
660300	898100	1071515	1308218	1672026	2128473	2647010	3308800
17226	23066	29556	28268	29136	36868	37806	27678
146028	122255	141916	166616	195173	183899	219520	239034
4904	5880	7462	8678	9850	11081	9823	10638
16371	19121	22544	25971	28698	31797	28881	31105
45002	44996	45072	45170	45084	44968	44983	44600
271900	291127	210511	240560	231078	224800	233370	226699
239570	270653	340796	369445	403722	419956	431498	449627
250025	295529	334243	351735	431748	491545	555907	626003
2646	2425	2389	1722	1778	1757	1686	2007
37842	34841	33339	22763	21506	20483	19974	24872
2970	2706	2614	1982	1872	1768	1888	2145
40029	36424	33003	22495	21017	20467	19930	23637
1886	1876	1844	1970	2484	2418	2228	2993
1275	1616	1681	1728	1995	2061	2371	2463
495	159	453	650	731	743	813	876
345	348	350	870	572	705	852	921

?1994-2018 China Academic Journal Electronic Publishing House. All rights reserved. http://www.cnki.net

6-5 凤翔县经济

指 标	单 位	2000年	2005年	2006年	2007年	2008年
年底总人口	万人	50.06	51.87	51.84	50.82	50.92
生产总值	亿元	19.55	42.05	50.56	60.92	73.33
第一产业	亿元	5.73	7.80	8.32	9.98	12.67
第二产业	亿元	9.66	25.05	30.64	36.59	42.48
第三产业	亿元	4.16	9.20	11.60	14.35	18.18
# 工业增加值	亿元	8.16	20.55	25.57	30.48	35.42
人均生产总值	元	3833	8183	9750	11754	14415
生产总值指数	上年=100	110.3	117.6	115.0	115.0	114.5
全社会固定资产投资	万元	23242	170240	200210	302457	418752
地方财政收入	万元	5652	6203	8098	11100	14163
地方财政支出	万元	9899	20352	29766	41781	58626
农村居民人均纯收入	元	1462	2352	2705	3609	4311
城镇居民人均可支配收入	元				9380	13214
常用耕地面积	公顷	49565	47105	47003	46909	46958
粮食产量	吨	224559	247883	297334	234143	286704
农林牧渔业总产值	万元	80724	128695	140798	165457	214560
社会消费品零售总额	万元	44800	87694	100934	141294	183984
普通小学专任教师数	人	2161	2193	2145	2051	2002
普通小学在校学生数	人	70466	44835	41543	37567	34067
普通中学专任教师数	人	1855	2214	2221	2241	2292
普通中学在校学生数	人	39464	43738	43726	41841	38393
卫生机构床位数	张	835	1164	1078	1079	1078
卫生技术人员	人	890	1002	1052	1055	1633
# 执业(助理)医师	人		132	474	474	494
注册护师、护士	人		211	251	251	577

?1994-2018 China Academic Journal Electronic Publishing House. All rights reserved. http://www.cnki.net

社会主要指标

2009年	2010年	2011年	2012年	2013年	2014年	2015年	2016年
48.27	48.38	48.48	48.61	48.71	48.82	48.95	49.14
85.20	102.80	120.84	143.01	161.96	181.81	184.93	202.04
14.19	15.40	19.26	20.90	22.84	24.17	24.22	25.05
49.89	63.42	73.75	90.95	104.87	118.49	109.85	121.10
21.12	23.98	27.84	31.16	34.25	39.15	50.86	55.90
41.50	53.31	61.78	77.08	89.47	102.17	91.45	100.41
16726	20173	24951	29459	33284	35615.0	37777.0	41194.0
116.3	120.7	114.5	114.1	114.6	110.5	110.3	108.6
580463	827603	1029871	1381243	1795399	2282157	2812865	3378800
19001	24991	29501	34760	41000	44915	47636	45028
85375	100050	136611	159456	176949	190019	220305	245255
5160	6342	7896	9159	10423	11716	10375	11225
16518	19656	23096	26630	29426	32428	29426	31662
47141	46796	46131	46181	46335	46332	45813	45724
298897	308786	261013	270408	260048	256353	264037	256439
220005	256997	317811	338535	371181	398017	406835	421979
215926	255657	289659	311754	334402	378048	429015	481598
1936	1878	2121	1777	1756	1723	1618	1432
31221	29071	27984	22971	21695	21093	21073	20981
2303	2312	2745	2490	2408	2270	2142	2064
36793	34200	36588	29450	26386	23832	20154	18539
1154	1163	1243	1283	1506	1656	1712	1830
1617	1707	1788	1876	1902	2288	2287	2458
486	143	185	585	571	660	639	726
561	500	564	590	612	667	680	845

?1994-2018 China Academic Journal Electronic Publishing House. All rights reserved.   http://www.cnki.net

6-6 岐山县经济

指　　标	单　位	2000年	2005年	2006年	2007年	2008年
年底总人口	万人	47.51	46.69	46.69	46.53	46.58
生产总值	亿元	19.46	43.89	51.00	58.77	71.93
第一产业	亿元	3.24	5.32	6.14	8.30	11.33
第二产业	亿元	10.30	26.57	31.02	34.55	42.16
第三产业	亿元	5.92	12.00	13.84	15.92	18.44
# 工业增加值	亿元	8.70	22.68	26.57	29.38	35.69
人均生产总值	元	4106	9560	10942	12609	15450
生产总值指数	上年=100	116.5	115.0	115.2	115.6	115.7
全社会固定资产投资	万元	27035	97874	136000	224376	322666
地方财政收入	万元	4030	5705	7428	9248	11311
地方财政支出	万元	7516	19176	28900	41509	52251
农村居民人均纯收入	元	1602	2482	2847	3477	4202
城镇居民人均可支配收入	元			8204	9402	13234
常用耕地面积	公顷	37258	35283	37220	37740	35222
粮食产量	吨	178238	250905	286025	248672	296462
农林牧渔业总产值	万元	54021	101467	114502	149282	190770
社会消费品零售总额	万元	61454	106957	138007	169557	216712
普通小学专任教师数	人	2091	2250	2211	2236	2288
普通小学在校学生数	人	62700	40100	39100	36400	32400
普通中学专任教师数	人	2084	2494	2468	2473	2413
普通中学在校学生数	人	37700	41400	41500	39700	37400
卫生机构床位数	张	1243	1226	1302	1338	1318
卫生技术人员	人	1226	1354	1329	1415	1446
# 执业(助理)医师	人	476	643	562	590	526
注册护师、护士	人	286	385	373	402	443

?1994-2018 China Academic Journal Electronic Publishing House. All rights reserved.　http://www.cnki.net

社会主要指标

2009年	2010年	2011年	2012年	2013年	2014年	2015年	2016年
45.83	45.94	46.04	46.15	46.25	46.36	46.48	46.65
83.57	96.74	113.57	134.01	144.09	149.10	144.43	156.11
12.25	14.37	18.43	20.51	22.41	22.54	22.96	23.74
49.87	58.08	66.89	81.64	86.38	86.45	79.06	84.98
21.45	24.29	28.25	31.86	35.29	40.10	42.41	47.39
42.18	49.00	55.66	67.48	71.23	69.84	61.06	64.44
17936	20756	24696	29075	31187	29286	30995	33525
116.1	116.2	114.5	113.7	112.7	112.1	110.5	110.0
456966	617772	774733	1056357	1315647	1748844	2156505	2748200
13572	16218	20044	23086	27402	32262	38121	36626
69720	83087	100872	141264	164799	172759	211824	243218
5244	6267	7884	9153	10435	11791	10483	11343
16555	19303	22681	26242	29050	32100	29212	31461
35266	35266	35253	35237	35237	35237	35248	35289
291841	290251	263544	275099	265000	265783	270048	262309
199600	240030	293567	323161	351924	375889	384523	396887
234567	277962	315208	342059	359149	406844	460904	523336
2328	2306	2337	1781	1935	1762	1588	1544
29000	25790	24336	21771	20917	20236	19821	20011
2411	2424	2366	2714	2427	2361	2287	2197
33400	31058	28979	25689	23571	21671	19821	19054
1507	1588	1594	1624	1825	1996	2053	2104
1514	1603	1682	1697	1861	2068	2218	2886
508	672	676	573	575	594	630	767
466	539	573	476	685	793	866	1058

?1994-2018 China Academic Journal Electronic Publishing House. All rights reserved. http://www.cnki.net

6-7 扶风县经济

指 标	单 位	2000年	2005年	2006年	2007年	2008年
年底总人口	万人	45.53	45.92	45.89	46.82	46.92
生产总值	亿元	10.76	25.95	29.80	35.96	42.93
第一产业	亿元	3.19	6.47	7.16	8.90	9.82
第二产业	亿元	4.13	10.52	12.40	15.18	19.19
第三产业	亿元	3.44	8.96	10.24	11.88	13.92
# 工业增加值	亿元	2.88	7.91	9.52	11.57	14.75
人均生产总值	元	2346	5663	6509	7682	9159
生产总值指数	上年=100	108.4	113.8	113.3	114.5	114.0
全社会固定资产投资	万元	17500	83842	99125	163000	242000
地方财政收入	万元	3620	2753	3181	3760	4559
地方财政支出	万元	8103	18076	25778	36833	49688
农村居民人均纯收入	元	1396	1949	2217	2750	3447
城镇居民人均可支配收入	元				9058	12578
常用耕地面积	公顷	42569	38150	37957	37174	36716
粮食产量	吨	263346	298900	300098	242458	293472
农林牧渔业总产值	万元	64878	104920	113735	142193	174520
社会消费品零售总额	万元	27237	51600	62100	81300	103400
普通小学专任教师数	人	1933	2109	2047	2073	2041
普通小学在校学生数	人	72348	44007	42421	40160	37223
普通中学专任教师数	人	2064	1764	1715	1508	1537
普通中学在校学生数	人	33533	30088	28507	25894	24380
卫生机构床位数	张	915	1120	1266	1266	1384
卫生技术人员	人	1095	1110	1244	1306	1351
# 执业(助理)医师	人	410	414	468	497	423
注册护师、护士	人	312	343	362	406	411

?1994-2018 China Academic Journal Electronic Publishing House. All rights reserved. http://www.cnki.net

社会主要指标

2009年	2010年	2011年	2012年	2013年	2014年	2015年	2016年
41.57	41.67	41.76	41.86	41.95	42.05	42.16	42.31
49.90	57.35	69.37	81.85	95.13	100.57	97.38	108.16
9.89	11.03	16.21	17.64	19.12	19.72	20.38	21.06
23.75	27.28	31.02	39.24	48.47	49.77	48.68	55.36
16.26	19.04	22.14	24.98	27.54	31.09	28.32	31.74
18.03	20.56	22.81	29.72	37.58	37.51	37.26	40.35
11556	13294	16630	19578	20196	21278	23244	25609
115.4	114.5	114.1	114.5	114.5	112.0	111.2	110.2
342000	464520	606834	849143	1119800	1498481	1853033	2313400
7808	11524	16088	20063	24384	27579	31735	26785
66890	96175	125680	153399	162189	176399	201819	218789
4285	5159	6438	7565	8647	9736	8646	9363
15257	17774	20902	24205	26892	29608	27125	29186
34892	34490	34091	33199	32715	32395	31802	31177
290065	289871	264287	280526	273000	265514	280898	272954
174039	217542	269670	299723	329922	350702	361807	374923
136831	161871	183076	183923	224141	256305	291604	330256
1789	1764	1700	1674	1670	1768	1720	1632
32256	30588	28211	23873	22760	22110	21782	21565
1407	1366	1887	1994	1978	1748	1719	1680
21281	28685	26934	24717	22590	21299	20416	19870
1439	1507	1507	1506	1452	1637	1655	1879
1296	1293	1302	1463	1408	1574	1568	1934
422	387	343	453	457	519	511	616
410	408	413	523	454	510	499	660

?1994-2018 China Academic Journal Electronic Publishing House. All rights reserved. http://www.cnki.net

6-8 眉县经济

指　　标	单　位	2000年	2005年	2006年	2007年	2008年
年底总人口	万人	29.89	30.44	30.44	30.82	30.82
生产总值	亿元	8.72	23.44	26.99	33.46	40.14
第一产业	亿元	2.67	4.31	4.76	6.14	7.25
第二产业	亿元	4.06	12.94	15.21	19.15	23.31
第三产业	亿元	2.00	6.18	7.02	8.18	9.58
# 工业增加值	亿元	3.68	10.48	12.50	15.93	19.11
人均生产总值	元	2902	7704	8883	10925	13024
生产总值指数	上年=100	108.8	114.1	113.8	114.5	114.8
全社会固定资产投资	万元	16084	96503	120542	170893	248532
地方财政收入	万元	3190	2934	3487	4451	5466
地方财政支出	万元	6902	15280	19387	27306	40610
农村居民人均纯收入	元	1378	2052	2349	2892	3619
城镇居民人均可支配收入	元				9136	12924
常用耕地面积	公顷	25287	22489	22634	26628	26601
粮食产量	吨	145682	142732	150394	162538	145914
农林牧渔业总产值	万元	45916	74227	82059	107556	137604
社会消费品零售总额	万元	28301	51888	60335	78094	100230
普通小学专任教师数	人	1389	1446	1432	1353	1318
普通小学在校学生数	人	42217	26734	24324	21855	19569
普通中学专任教师数	人	1210	1471	1510	1510	1547
普通中学在校学生数	人	25117	36383	36371	35573	34925
卫生机构床位数	张	499	729	844	845	1020
卫生技术人员	人	613	777	876	886	819
# 执业(助理)医师	人	195	329	372	374	336
注册护师、护士	人	57	196	193	199	202

?1994-2018 China Academic Journal Electronic Publishing House. All rights reserved.   http://www.cnki.net

社会主要指标

2009年	2010年	2011年	2012年	2013年	2014年	2015年	2016年
29.95	30.02	30.08	30.16	30.22	30.29	30.37	30.45
46.50	53.30	62.35	77.19	90.57	104.63	108.61	115.51
8.78	10.47	13.33	15.18	18.06	20.37	20.44	20.98
25.23	28.45	32.25	43.21	51.75	60.86	57.93	60.62
12.48	14.39	16.78	18.79	20.76	23.40	30.24	33.91
19.85	22.15	24.62	34.34	41.50	48.34	48.48	51.32
15067	17223	20748	25625	31325	34583	32685	37981
115.8	114.6	114.8	120.0	116.2	116.1	114.0	.112.0
355397	479395	617731	1042892	1461278	1990858	2453021	3015800
6453	8006	13143	20022	26049	29963	33869	30188
53438	63504	88370	140463	149695	164704	179783	190612
4535	5528	7021	8313	9527	10755	9590	10386
16349	19325	22765	26817	29874	32981	30127	32417
26426	23985	23950	23157	23188	23161	23236	23175
151402	144034	132125	134532	126000	126173	123284	117587
145458	196060	243345	290770	331773	363300	375668	386713
115317	136420	155928	164607	218455	249803	283249	322356
1265	1235	1197	1151	1213	1208	1167	1019
18294	17673	16865	16179	16545	16094	17010	18031
1547	1492	1540	1504	1488	1413	1365	1243
31113	23547	20496	17865	15218	14184	13504	13497
1100	1172	1215	1330	1366	1574	1669	1880
821	825	1192	1426	1710	1982	2084	2314
337	349	454	471	480	535	533	583
199	200	397	497	595	680	448	845.00

?1994-2018 China Academic Journal Electronic Publishing House. All rights reserved. http://www.cnki.net

6-9 陇县经济

指　　标	单　位	2000年	2005年	2006年	2007年	2008年
年底总人口	万人	25.19	25.31	25.45	25.38	25.43
生产总值	亿元	6.48	13.85	15.76	17.59	22.23
第一产业	亿元	2.79	5.28	6.03	7.05	9.09
第二产业	亿元	1.34	3.95	4.76	4.77	6.30
第三产业	亿元	2.34	4.62	4.97	5.78	6.84
# 工业增加值	亿元	1.04	2.99	3.67	3.45	4.66
人均生产总值	元	2641	5525	6274	6951	8748
生产总值指数	上年=100	111.5	113.6	115.2	113.4	112.8
全社会固定资产投资	万元	6821	38345	49458	75573	119134
地方财政收入	万元	3104	3167	3608	4501	5772
地方财政支出	万元	6357	13861	18202	26613	33698
农村居民人均纯收入	元	1080	1863	2103	2480	3079
城镇居民人均可支配收入	元	3937	5549	6742	7942	9583
常用耕地面积	公顷	41783	36082	36301	36227	35807
粮食产量	吨	87840	110383	117465	93428	105153
农林牧渔业总产值	万元	43601	83047	97050	114458	146171
社会消费品零售总额	万元	20996	42998	52791	68879	88196
普通小学专任教师数	人	1399	1332	1374	1335	1324
普通小学在校学生数	人	36300	26400	24700	22800	21200
普通中学专任教师数	人	905	1042	1109	1171	1242
普通中学在校学生数	人	14500	19000	18900	18800	18900
卫生机构床位数	张	715	715	715	1180	1180
卫生技术人员	人	813	920	994	1032	1048
# 执业(助理)医师	人	236	340	374	386	398
注册护师、护士	人	113	172	175	189	194

?1994-2018 China Academic Journal Electronic Publishing House. All rights reserved.　http://www.cnki.net

社会主要指标

2009年	2010年	2011年	2012年	2013年	2014年	2015年	2016年
24.85	24.91	24.96	25.03	25.08	25.14	25.20	25.25
25.60	29.38	36.71	43.30	49.88	57.16	59.22	67.55
9.71	11.72	14.65	16.25	17.29	16.61	17.13	17.88
7.88	8.43	11.28	14.91	19.19	24.03	23.54	29.15
8.01	9.23	10.77	12.14	13.40	16.52	18.55	20.52
5.94	6.14	8.43	11.60	15.18	19.52	19.80	23.55
10017	11510	14722	17322	20275	22762	23723	26776
115.2	114.3	114.3	115.5	114.6	113.0	112.0	110.3
213665	305711	406831	662503	866188	1160188	1441867	1823600
8400	11014	14485	19163	23157	26805	30375	18913
53666	73333	89032	124368	130086	138954	154625	173820
3799	4570	5717	6740	7644	8568	7581	8226
12675	14995	17739	20630	23002	25464	24179	26089
35554	35577	35292	35436	35479	35442	35413	35368
113662	116542	101013	110804	101012	100101	105230	103314
150171	195405	240760	266633	286761	305299	313671	328986
95364	112911	128266	139101	144955	162712	184208	206686
1335	1194	1134	1081	1065	1013	1218	904
19800	18796	17940	16760	16473	16535	16767	17387
1290	1257	1270	1285	1234	1232	1218	1176
18400	17315	16353	14350	13903	13463	13049	13208
1180	779	1009	1129	1245	1348	1378	1380
1095	863	1006	1082	1164	1209	1289	1376
458	330	360	349	364	377	363	364
217	237	300	348	380	417	448	505

?1994-2018 China Academic Journal Electronic Publishing House. All rights reserved. http://www.cnki.net

6-10 千阳县经济

指　　标	单　位	2000年	2005年	2006年	2007年	2008年
年底总人口	万人	12.77	12.63	12.63	12.96	12.96
生产总值	亿元	2.97	6.51	7.42	8.76	11.41
第一产业	亿元	1.47	2.65	3.00	3.54	4.33
第二产业	亿元	0.62	1.73	1.98	2.40	3.75
第三产业	亿元	0.87	2.13	2.44	2.82	3.33
# 工业增加值	亿元	0.45	1.07	1.22	1.50	2.66
人均生产总值	元	2322	5148	5861	6838	8806
生产总值指数	上年=100	110.7	113.9	113.3	114.8	113.7
全社会固定资产投资	万元	4631	35800	46458	73868	101689
地方财政收入	万元	1167	2074	2103	2430	3008
地方财政支出	万元	4277	10546	12932	17811	24066
农村居民人均纯收入	元	1016	1957	2191	2520	3167
城镇居民人均可支配收入	元				7702	10403
常用耕地面积	公顷	21755	18185	18093	18128	17997
粮食产量	吨	57440	59958	62961	52250	60634
农林牧渔业总产值	万元	24365	39187	44900	54774	75744
社会消费品零售总额	万元	8433	13679	18246	21603	31376
普通小学专任教师数	人	632	619	598	575	560
普通小学在校学生数	人	17400	13000	12600	11800	10800
普通中学专任教师数	人	421	520	533	514	542
普通中学在校学生数	人	7100	9000	9000	8600	8100
卫生机构床位数	张	320	360	361	411	450
卫生技术人员	人	361	363	376	413	453
# 执业(助理)医师	人	110	146	150	151	150
注册护师、护士	人	130	151	153	157	167

?1994-2018 China Academic Journal Electronic Publishing House. All rights reserved.　http://www.cnki.net

社会主要指标

2009年	2010年	2011年	2012年	2013年	2014年	2015年	2016年
12.38	12.40	12.43	12.46	12.49	12.52	12.55	12.57
13.33	15.61	20.80	25.94	31.01	35.06	41.75	48.66
5.03	5.38	7.96	8.84	9.94	9.18	9.57	10.34
4.62	6.03	7.96	11.65	15.03	18.75	21.67	26.79
3.68	4.21	4.88	5.45	6.03	7.13	10.51	11.54
3.34	4.55	6.16	9.68	12.81	16.42	19.17	24.01
10242	11959	16302	20839	24358	28432	33303	38740
115.7	114.7	118.6	118.0	117.2	116.9	112.3	110.7
201272	282028	319018	446789	584783	785393	954461	1150000
3383	4201	5615	7132	8616	10353	11477	7414
35363	41476	50412	77806	84702	97571	104636	121453
3946	4766	5958	7007	7938	8955	7915	8572
14102	16669	19536	22525	25070	27703	26341	28369
18030	17817	17824	18574	18567	18552	18599	18677
64281	69514	53625	61280	55003	56297	58908	56707
79758	101960	124780	140736	154688	160611	168664	181288
40705	48113	54367	45211	67805	76043	86355	99492
550	542	534	489	513	494	465	432
10100	9285	8761	7704	7176	6940	6826	6817
541	542	526	519	507	496	478	475
7600	7155	6585	6153	6111	5899	5641	5561
456	414	446	508	762	774	709	709
503	524	561	656	676	638	650	660
142	154	166	183	185	174	164	167
180	172	167	224	247	226	245	243

?1994-2018 China Academic Journal Electronic Publishing House. All rights reserved.   http://www.cnki.net

6-11　麟游县经济

指　　标	单　位	2000年	2005年	2006年	2007年	2008年
年底总人口	万人	8.71	9.09	8.90	8.81	8.81
生产总值	亿元	2.07	5.08	5.75	6.91	8.31
第一产业	亿元	0.69	2.01	2.13	2.71	3.24
第二产业	亿元	0.50	1.34	1.64	1.88	2.37
第三产业	亿元	0.87	1.73	1.98	2.32	2.70
# 工业增加值	亿元	0.41	1.04	1.32	1.51	1.93
人均生产总值	元	2376	5814	6574	7842	9416
生产总值指数	上年=100	110.1	112.8	112.0	113.5	113.0
全社会固定资产投资	万元	6683	19611	25690	41364	69690
地方财政收入	万元	843	823	1136	1638	2208
地方财政支出	万元	3522	7250	10453	15099	19084
农村居民人均纯收入	元	927	1499	1697	2010	2525
城镇居民人均可支配收入	元	3010	4276	4602	6260	9105
常用耕地面积	公顷	24776	18813	18950	31407	31542
粮食产量	吨	51466	65578	66105	50153	58862
农林牧渔业总产值	万元	15270	31743	33857	41099	54031
社会消费品零售总额	万元	7497	12526	14649	21823	26183
普通小学专任教师数	人	496	507	515	501	518
普通小学在校学生数	人	13250	11171	9670	8724	7378
普通中学专任教师数	人	243	344	358	359	393
普通中学在校学生数	人	4587	6565	7231	6610	6710
卫生机构床位数	张	339	339	339	371	449
卫生技术人员	人	271	308	324	336	347
# 执业(助理)医师	人	132	153	158	164	170
注册护师、护士	人	49	67	72	76	80

?1994-2018 China Academic Journal Electronic Publishing House. All rights reserved.　http://www.cnki.net

社会主要指标

2009年	2010年	2011年	2012年	2013年	2014年	2015年	2016年
9.06	9.08	9.10	9.12	9.14	9.16	9.19	9.22
10.17	13.29	19.25	41.15	50.59	55.75	65.54	71.11
3.49	4.51	5.52	6.24	6.69	6.59	6.70	7.13
3.63	5.31	10.26	31.06	39.66	43.99	51.05	55.44
3.04	3.47	3.47	3.86	4.25	5.17	7.79	8.54
2.62	4.43	9.37	30.21	38.68	42.59	49.47	53.41
11505	14985	21181	45176	55413	62805	70163	77268
115.4	120.4	121.0	122.0	121.2	118.9	114.6	113.0
248293	426723	477964	668961	879892	1159126	1436928	1788200
3086	4160	6890	12960	16881	19188	21137	22430
28633	35759	45305	71581	76031	90789	95341	101202
3697	4529	5684	6696	7580	8512	7518	8157
13192	15619	18384	21215	23676	26304	25046	26974
34537	34631	34652	29530	30252	30045	30057	30057
65903	77951	53998	63284	60236	66326	72780	78558
58074	71016	87669	95471	102609	107561	111414	117637
38096	45144	50967	60733	46577	52609	59711	66914
541	509	529	460	447	416	412	378
6955	6224	5683	4854	4617	4357	4098	3928
413	405	477	387	376	391	401	387
6444	5784	5159	4496	4319	4059	3836	3605
449	419	454	596	460	549	534	535
371	343	436	478	493	618	713	771
175	132	190	158	162	165	195	221
88	73	90	118	147	181	199	230

?1994-2018 China Academic Journal Electronic Publishing House. All rights reserved. http://www.cnki.net

6-12 凤县经济

指　　标	单　位	2000年	2005年	2006年	2007年	2008年
年底总人口	万人	9.84	10.94	10.85	10.24	10.24
生产总值	亿元	5.56	13.64	16.92	24.88	33.51
第一产业	亿元	0.39	0.55	0.60	0.66	1.34
第二产业	亿元	2.87	8.74	11.35	18.59	25.63
第三产业	亿元	2.30	4.35	4.97	5.63	6.54
# 工业增加值	亿元	2.67	8.23	10.78	17.90	24.41
人均生产总值	元	5655	13061	15532	23597	32729
生产总值指数	上年=100	114.8	115.4	115.7	117.5	120.3
全社会固定资产投资	万元	9985	41901	54015	106932	225786
地方财政收入	万元	2627	3770	5082	10084	12613
地方财政支出	万元	4589	12284	12444	24212	32972
农村居民人均纯收入	元	1241	1892	2170	2850	3700
城镇居民人均可支配收入	元			8021	9786	12875
常用耕地面积	公顷	10988	8850	8950	8956	12735
粮食产量	吨	34602	36743	38919	31546	33751
农林牧渔业总产值	万元	14508	8668	9579	10961	19122
社会消费品零售总额	万元	12143	16627	20367	25180	32000
普通小学专任教师数	人	553	569	549	519	480
普通小学在校学生数	人	12300	8000	7600	6700	6300
普通中学专任教师数	人	375	375	385	379	412
普通中学在校学生数	人	5700	7100	6900	6500	6200
卫生机构床位数	张	271	316	337	337	344
卫生技术人员	人	395	396	303	361	369
# 执业(助理)医师	人	93	138	133	137	123
注册护师、护士	人	82	73	78	70	92

?1994-2018 China Academic Journal Electronic Publishing House. All rights reserved. http://www.cnki.net

社会主要指标

2009年	2010年	2011年	2012年	2013年	2014年	2015年	2016年
10.53	10.56	10.58	10.61	10.63	10.65	10.68	10.73
46.65	66.54	91.15	110.80	130.21	145.61	148.86	170.74
2.47	3.84	4.52	5.63	6.43	6.37	6.65	6.88
34.52	48.08	69.37	85.79	102.55	115.19	118.03	139.57
9.66	14.62	17.25	19.37	21.23	24.05	22.18	24.29
33.08	46.38	67.32	83.43	99.86	112.28	117.82	135.96
45538	64913	86235	104582	116254	135479	139167	159479
119.9	123.2	119.1	121.0	114.5	115.7	114.3	110.7
444116	580708	758712	1107720	1448766	1755007	2137092	2595500
18778	25442	33633	38095	41153	37655	39175	29038
40662	53532	81526	91572	80489	82366	119451	117177
4695	5949	7621	9061	10402	11765	10236	11055
16488	19736	23387	27550	30415	33548	30432	32715
8954	9467	9467	9714	9645	9624	9631	9620
37528	35591	26122	28714	22025	22389	23637	23282
41252	66472	83353	98099	111174	118555	123185	128764
80091	95148	111132	123077	142611	162648	184850	210939
463	538	516	480	502	479	471	433
6000	5922	5619	5083	4818	4656	4458	4296
428	375	382	403	394	381	383	358
5900	5246	5015	4883	4774	4407	4245	3830
355	414	463	464	463	463	464	460
466	476	505	575	564	626	714	747
133	140	162	167	171	192	203	206
103	97	114	168	176	203	245	234

?1994-2018 China Academic Journal Electronic Publishing House. All rights reserved. http://www.cnki.net

6-13 太白县经济

指　　标	单　位	2000年	2005年	2006年	2007年	2008年
年底总人口	万人	5.17	5.48	5.49	5.18	5.18
生产总值	亿元	2.08	4.00	4.30	5.13	6.58
第一产业	亿元	0.75	1.37	1.38	1.57	2.38
第二产业	亿元	0.84	1.56	1.69	2.13	2.48
第三产业	亿元	0.49	1.08	1.23	1.44	1.72
# 工业增加值	亿元	0.64	1.19	1.28	1.63	1.88
人均生产总值	元	4024	7271	7488	8940	11801
生产总值指数	上年=100	110.3	112.6	111.0	113.9	113.4
全社会固定资产投资	万元	3954	16960	20204	26350	45523
地方财政收入	万元	1086	809	824	1104	1572
地方财政支出	万元	3239	7335	9722	15711	20893
农村居民人均纯收入	元	1233	1462	1608	1838	2405
城镇居民人均可支配收入	元				6685	9238
常用耕地面积	公顷	5777	6833	6763	6718	6562
粮食产量	吨	17836	12344	12265	13452	10841
农林牧渔业总产值	万元	14138	22850	26549	31687	37502
社会消费品零售总额	万元	5745	10535	10529	16141	19974
普通小学专任教师数	人	470	433	417	434	452
普通小学在校学生数	人	6537	4687	4577	4491	4356
普通中学专任教师数	人	185	200	168	222	220
普通中学在校学生数	人	2667	2901	3152	3702	3576
卫生机构床位数	张	192	192	192	284	284
卫生技术人员	人	213	185	184	184	190
# 执业(助理)医师	人	78	73	74	80	74
注册护师、护士	人	30	37	38	45	41

?1994-2018 China Academic Journal Electronic Publishing House. All rights reserved.　http://www.cnki.net

社会主要指标

2009年	2010年	2011年	2012年	2013年	2014年	2015年	2016年
5.08	5.10	5.11	5.12	5.13	5.14	5.16	5.19
7.65	9.10	11.35	13.79	15.83	17.11	19.92	20.91
2.61	3.31	4.10	4.61	5.17	5.29	5.76	5.85
3.05	3.50	4.55	6.17	7.33	7.85	8.75	9.01
1.99	2.29	2.69	3.01	3.33	3.97	5.43	6.05
2.34	2.66	3.56	5.06	6.07	6.49	7.24	7.31
14766	17558	22243	26974	30883	34753	38054	40423
116.1	113.8	114.0	113.5	113.0	112.1	112.0	110.0
75022	101200	115371	167405	218479	292489	362236	450100
2006	2655	4914	6326	7439	9043	10924	9111
26403	35789	40467	59973	68601	73927	93310	97379
3656	4479	5640	6610	7476	8463	7540	8181
12845	14964	17658	20713	23054	25497	24295	26214
6567	6631	6665	6513	6482	6460	6475	6498
11113	10201	6269	7331	6370	6405	7592	7380
43096	54937	65970	76011	85366	91208	99085	102951
25567	30221	34119	24337	33309	37456	42401	47801
436	388	368	392	386	369	352	319
4091	3794	3537	2781	2622	2509	2416	2393
196	253	322	259	267	266	271	255
3326	3001	3868	2207	2072	1946	1855	1756
284	227	227	226	226	206	196	198
185	190	204	210	235	200	213	207
73	70	72	68	74	63	71	74
37	42	43	48	57	48	49	51

?1994-2018 China Academic Journal Electronic Publishing House. All rights reserved. http://www.cnki.net
