# Supplementary material for: A study on the tourism efficiency of tourism destination based on DEA model: A case of ten cities in Shaanxi province
Source: PLoS One. 2024 Jan 19;19(1):e0296660. doi: 10.1371/journal.pone.0296660 (PMC10798521; doi:10.1371/journal.pone.0296660)
Supplement: S1 File — (ZIP) [file pone.0296660.s001.zip › Supporting information/Statistical yearbook/Hanzhong.caj]

十、汉中市

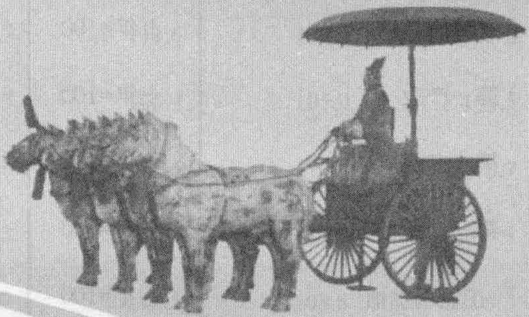

资料整理：左　丹　伍池宏　张　梦

?1994-2018 China Academic Journal Electronic Publishing House. All rights reserved.　http://www.cnki.net

10-1 汉中市经济

指　　标	单 位	2000年	2005年	2006年	2007年	2008年
年底总人口	万人	368.93	348.46	349.62	350.48	351.33
人口自然增长率	‰		2.30	2.45	2.46	2.45
年底总户数	万户	107.29	113.45	113.95	117.88	121.70
生产总值	亿元	119.23	217.72	249.83	299.71	366.19
第一产业	亿元	31.41	48.07	55.64	66.77	87.64
第二产业	亿元	38.41	85.38	99.01	115.39	135.03
第三产业	亿元	49.41	84.27	95.18	117.55	143.52
# 工业增加值	亿元	26.77	64.72	75.67	87.82	99.22
人均生产总值	元	3250	6255	7158	8562	10435
生产总值指数	上年=100	108.2	111.9	112.1	113.9	113.8
第一产业	上年=100	103.8	109.2	108.1	106.0	107.9
第二产业	上年=100	109.3	112.0	115.6	114.9	113.8
第三产业	上年=100	110.2	113.0	110.8	117.3	116.8
# 工业增加值	上年=100	106.0	114.1	118.1	116.3	113.8
人均生产总值指数	上年=100	107.5	111.2	111.8	113.6	113.5
非公有制经济增加值	亿元		91.83	111.62	140.86	175.89
文化产业增加值	亿元					
单位GDP能耗	吨标准煤/万元		1.800	1.739	1.655	1.563
单位GDP能耗比上年增长	%			-3.40	-4.80	-5.60
就业人员	万人	207.51	188.84	212.64	209.45	208.00
城镇单位就业人员	万人	24.96	21.97	22.03	21.90	21.49
# 国有单位	万人	20.38	16.28	15.88	15.60	15.39
集体单位	万人	2.90	1.64	1.27	1.19	1.03
# 在岗职工人数	万人	24.96	21.97	22.03	21.90	21.49
城镇单位就业人员平均工资	元					
城镇单位在岗职工平均工资	元	6437	12248	13957	17968	22333

?1994-2018 China Academic Journal Electronic Publishing House. All rights reserved.   http://www.cnki.net

社会主要指标

2009年	2010年	2011年	2012年	2013年	2014年	2015年	2016年
342.09	341.76	341.51	341.84	342.50	343.15	343.81	344.63
2.38	2.47	2.44	2.55	2.63	2.42	2.42	2.72
124.34	125.97	127.59	128.77	129.93	130.74	131.86	132.97
415.64	509.70	647.48	754.57	890.31	1002.83	1059.61	1156.49
91.71	110.39	142.29	159.47	171.52	183.98	191.53	205.74
152.48	199.50	267.58	320.42	391.01	453.60	459.02	495.03
171.45	199.81	237.61	274.68	327.78	365.25	409.06	455.72
108.58	146.32	203.17	245.70	303.16	353.28	345.46	365.69
11819	14907	18952	22084	26020	29252	30849	33597
114.5	115.1	115.5	115.2	112.7	111.6	109.6	109.0
106.4	106.6	106.6	105.8	105.3	105.4	105.0	104.5
113.6	119.6	122.2	121.8	118.6	115.5	110.4	110.7
119.3	114.3	113.7	112.9	109.3	109.5	110.4	109.1
111.0	120.6	125.5	124.7	120.7	116.5	108.8	109.3
114.3	115.2	115.6	115.1	112.5	111.4	109.3	108.7
203.14	252.35	327.03	387.88	467.60	516.86	545.60	595.87
					24.05	26.36	29.38
1.494	1.099	1.059	1.022	0.986	0.946	0.899	0.755
-4.38	-3.63	-3.61	-3.56	-3.51	-4.05	-4.96	-4.66
207.83	206.37	205.33	199.50	203.50	202.10	203.33	202.46
23.98	25.01	25.66	26.37	30.02	30.87	30.32	29.97
15.34	15.81	15.82	16.21	15.16	15.52	15.62	15.66
1.27	1.36	1.33	1.26	1.26	1.91	1.71	1.86
21.50	22.15	22.85	23.35	27.32	27.67	27.07	26.44
			39106	42160	44417	48893	52736
26629	30148	35119	40739	43710	46348	51411	55739

?1994-2018 China Academic Journal Electronic Publishing House. All rights reserved.   http://www.cnki.net

10-1 续表 1

指 标	单 位	2000年	2005年	2006年	2007年	2008年
全社会固定资产投资	亿元	38.84	75.00	90.88	120.05	163.63
# 房地产开发	亿元	6.15	12.49	14.11	22.85	22.97
商品房销售面积	万平方米	41.42	79.53	101.69	112.59	106.86
# 住宅	万平方米	39.77	76.34	97.34	104.85	101.52
地方财政收入	亿元	5.20	6.40	7.69	10.24	12.33
地方财政支出	亿元	13.00	25.02	32.62	48.41	77.72
金融机构人民币各项存款余额	亿元	154.46	307.97	369.98	404.58	534.07
金融机构人民币各项贷款余额	亿元	150.31	150.12	165.42	179.26	178.28
农村居民人均纯收入	元	1428	1893	2038	2393	2884
城镇居民人均可支配收入	元	4117	6257	6925	8209	10155
城市人均公园绿地面积	平方米			6.3	6.5	14.3
城市人均道路面积	平方米		6.0	6.2	4.2	5.9
城市用水普及率	%		81.9	76.2	78.1	82.0
城市燃气普及率	%		28.2	28.6	37.2	68.3
常用耕地面积	千公顷	231.56	202.15	200.16	201.03	202.35
农林牧渔业总产值	亿元	55.15	83.22	94.79	114.63	150.49
农作物总播种面积	千公顷	459.67	445.75	447.17	408.56	492.99
# 粮食作物	千公顷	346.36	301.73	303.56	264.88	277.21
粮食产量	万吨	124.50	111.69	116.89	93.23	102.12
棉花产量	吨	181	37	36	33	29
油料产量	吨	105420	146944	124889	134942	151344
蔬菜产量	吨	1018968	1024168	1095835	1198475	1385397

?1994-2018 China Academic Journal Electronic Publishing House. All rights reserved. http://www.cnki.net

2009年	2010年	2011年	2012年	2013年	2014年	2015年	2016年
238.43	312.80	411.33	534.86	679.27	845.04	1039.40	1209.89
24.21	38.22	57.21	73.41	69.09	82.10	68.20	85.40
145.17	165.34	216.45	185.02	164.35	127.16	124.69	141.83
140.78	157.78	205.14	176.90	156.36	118.92	116.62	128.96
14.39	18.62	23.55	30.09	35.83	40.89	44.67	45.19
116.63	126.79	159.25	195.84	214.40	234.00	258.65	280.17
676.57	798.11	932.82	1072.86	1246.83	1390.67	1591.92	1752.53
241.57	300.08	347.95	402.44	491.85	554.85	630.54	680.21
3446	4183	5283	6181	7053	7439	8164	8855
12562	14509	17019	19827	22167	21725	23625	25595
14.1	14.1	15.0	15.0	14.8	14.6	13.2	13.4
5.9	6.3	6.9	7.4	7.5	7.9	7.5	9.1
75.8	75.7	81.9	80.5	81.0	79.7	82.5	81.5
61.1	67.6	83.7	90.9	97.7	100.0	91.0	89.9
202.99	203.59	204.62	205.26	205.08	205.15	204.05	203.77
159.10	190.34	245.56	274.66	305.07	325.51	339.66	364.26
443.62	448.03	432.95	438.11	442.29	517.53	518.09	518.97
284.10	285.87	267.00	268.90	269.24	268.25	267.39	266.28
109.87	114.13	95.14	101.36	102.66	101.75	103.24	103.65
10	2	3	3	3	1	53	53
159260	170121	176689	178941	184756	189144	192065	192038
1597018	1744760	1794085	1910851	2024013	2143400	2272298	2427099

?1994-2018 China Academic Journal Electronic Publishing House. All rights reserved. http://www.cnki.net

10-1 续表 2

指 标	单 位	2000年	2005年	2006年	2007年	2008年
水果产量	吨	62087	198409	192948	263457	266842
# 苹果	吨	4677	3909	4473	4720	4814
肉类产量	吨	157802	223341	245898	191607	212744
# 猪牛羊肉	吨	147844	207893	227945	171346	196206
奶类产量	吨	4833	8883	9407	9742	10771
# 牛奶	吨	4636	8268	8710	9139	9495
禽蛋产量	吨	20818	38739	40632	48718	53279
水产品产量	吨	12204	18173	18331	19427	20202
规模以上工业企业单位数	个	251	231	252	250	266
规模以上工业总产值	亿元	68.43	165.41	204.37	241.11	260.60
纱产量	吨	919	2070	2283	3676	6207
布产量	万米	293.91	823.80	1278.00	1157.10	1128.93
原煤产量	万吨	8.62	8.40	14.90	21.93	26.22
发电量	亿千瓦小时	11.29	14.78	14.68	20.89	23.74
粗钢产量	万吨	27.38	98.06	126.75	117.20	94.25
钢材产量	万吨	27.38	21.15	38.48	64.24	54.08
水泥产量	万吨	108.04	203.37	234.54	255.00	264.38
汽车产量	辆	291	1294	4666		
建筑业企业单位数	个	119	85	92	94	97
建筑业企业年末从业人员	万人	3.64	3.32	3.48	3.18	3.07
建筑业总产值	亿元	9.00	16.64	16.65	20.46	25.39
房屋建筑施工面积	万平方米	258.13	336.70	313.18	377.75	417.87
房屋建筑竣工面积	万平方米	138.06	153.35	137.90	168.34	159.93
公路里程	公里	6227	7305	14003	13662	12398
# 等级公路	公里	4667	5608	5695	8385	8930
# 高速公路	公里		106	166	192	210
民用汽车拥有量	辆	24391	43110	56024	68029	78171
# 私人汽车	辆	10129	24733	40180	49155	57610
邮电业务总量	亿元	5.61	19.40	24.56	29.60	37.17
邮政业务总量	亿元	0.48	1.52	2.04	2.50	2.99
电信业务总量	亿元	5.13	17.89	22.52	27.10	34.18

?1994-2018 China Academic Journal Electronic Publishing House. All rights reserved. http://www.cnki.net

2009年	2010年	2011年	2012年	2013年	2014年	2015年	2016年
335157	305389	382869	397746	411483	430669	455763	462576
4875	4954	4806	4559	4602	4593	4711	4863
250761	279910	297264	312077	324562	316589	332560	330139
232094	259511	274839	289308	300905	292967	308697	306626
11679	13097	13783	13756	13820	13967	13688	13297
10552	11693	12171	12101	12144	12213	11905	11568
55237	62068	69111	70095	71569	71434	72899	73842
21118	23498	23594	26240	31248	35100	37671	40805
340	353	254	324	348	392	425	461
310.79	404.41	545.42	718.09	856.10	873.56	922.03	992.02
8184	8172	8862	9740	14477	13614	15816	18393
1341.48	1040.00	1179.00	1112.00	1291.00	714.00	916.00	846.00
61.47	101.78	135.09	169.49	199.87	219.20	236.89	234.96
22.61	32.88	28.56	30.88	35.21	45.01	48.84	62.70
145.59	182.35	201.41	301.14	428.19	434.74	384.32	347.11
125.52	145.89	155.53	233.65	455.98	455.20	332.97	265.20
308.62	508.35	618.80	664.71	702.14	700.09	698.46	663.33
98	98	94	105	102	114	119	129
3.43	4.23	3.42	4.33	4.88	5.14	6.19	6.09
37.72	52.26	70.00	87.68	102.91	120.87	145.19	178.17
511.50	708.71	875.80	991.16	1201.75	1347.01	1333.54	1571.13
219.69	266.97	312.28	337.04	413.84	482.91	460.09	610.21
14298	15051	15974	17935	18462	18828	19312	20062
11458	13785	14021	15598	16216	16791	17329	18262
210	307	431	428	465	465	502	505
86510	101494	113814	131267	153494	174218	198993	231104
66799	79665	91305	107585	128854	150810	176642	209485
35.79	16.77	21.91	23.05	25.96	34.91	47.70	74.79
3.60	3.30	4.04	3.22	3.84	4.45	5.15	6.42
32.19	13.47	17.87	19.83	22.13	30.46	42.56	68.37

?1994-2018 China Academic Journal Electronic Publishing House. All rights reserved. http://www.cnki.net

10-1 续表 3

指　　标	单 位	2000年	2005年	2006年	2007年	2008年
固定电话用户	万户	22.61	66.61	72.20	72.52	65.94
移动电话用户	万户	9.89	60.80	74.31	105.33	120.00
互联网宽带用户	万户	1.29	5.47	6.90	10.18	13.82
限额以上企业数	个					
批发业	个					
零售业	个					
住宿业	个					
餐饮业	个					
社会消费品零售总额	亿元	42.91	65.30	76.10	91.80	116.66
进出口总额	万美元		2267	2130	2944	3720
# 出口	万美元		1913	1869	2205	3111
实际外商直接投资额	万美元		541	251	674	826
入境旅游人数	万人次	0.51	0.69	0.76	1.10	1.25
# 外国人	万人次		0.18	0.19	0.21	0.24
国际旅游外汇收入	万美元	64	98	148	260	330
国内旅游人数	万人次	452	560	620	732	820
国内旅游收入	亿元	4.07	8.10	12.50	17.00	23.00
星级饭店数	个		14	14	28	28
幼儿园数	所	405	141	158	164	171
在园儿童数	万人	8.33	4.78	4.67	3.31	4.59
普通小学学校数	所	3603	2196	1909	1758	1570
普通小学专任教师数	人	18274	16813	16628	16524	16265
普通小学在校学生数	万人	38.63	30.27	29.23	27.93	26.32
普通中学学校数	所	249	254	248	244	241
普通中学专任教师数	人	10822	12463	12844	13191	13373
普通中学在校学生数	万人	18.51	22.13	21.94	21.77	21.63
卫生机构数	个	596	523	468	402	402
卫生机构床位数	张	11197	10194	10586	10598	11447
卫生技术人员	人	13619	12916	13058	12873	13930
# 执业(助理)医师	人	7407	5198	5299	5473	5408
注册护师、护士	人	3157	3108	3217	3392	4015

?1994-2018 China Academic Journal Electronic Publishing House. All rights reserved.　http://www.cnki.net

2009年	2010年	2011年	2012年	2013年	2014年	2015年	2016年
61.80	62.68	59.34	57.08	55.58	53.31	52.58	49.79
138.35	151.10	177.80	199.94	215.21	234.63	251.83	266.71
13.82	21.77	24.42	29.64	32.53	35.49	38.98	50.35
123	204	231	294	332	372	395	423
16	24	25	35	44	52	50	52
58	100	125	162	189	215	231	248
36	51	50	57	56	53	59	63
13	29	31	40	43	52	55	60
133.02	157.50	184.44	216.05	248.15	281.65	319.00	369.17
4071	4422	4595	5426	7315	6.37(亿元)	5.54(亿元)	7.02(亿元)
3268	3032	3948	5256	6670	4.54(亿元)	4.34(亿元)	4.80(亿元)
3597	1595	2428	3014	3100	4005	1054	2013
1.52	1.72	2.01	2.31	2.79	3.40	3.87	4.25
0.29	1.22	1.42	1.51	1.84	2.20	2.62	2.90
490	690	830	998	1269	1561	1836	2120
1030	1210	1504	1903	2247	2625	2911	3256
31.60	48.70	63.00	81.50	105.18	130.42	151.70	172.00
31	30	30	29	29	29	29	30
192	655	731	750	745	777	809	780
4.47	7.37	8.80	9.68	9.94	10.17	10.07	10.07
1325	972	876	769	742	700	559	489
16016	15631	14330	13948	13266	12819	12218	12208
24.99	23.85	22.67	21.22	19.71	19.52	19.54	19.74
232	225	219	207	208	212	212	211
13643	13866	14998	14246	15209	15290	14355	15728
21.75	21.44	21.12	20.17	19.21	18.77	18.31	18.10
401	412	3951	3897	3858	3858	3814	3698
12200	13309	14580	16332	18212	19805	20960	20580
14260	15833	16499	17619	19434	20065	20356	21973
5669	5231	5484	5901	6367	6191	5757	6660
4191	4903	5145	5786	6713	7237	7410	8308

?1994-2018 China Academic Journal Electronic Publishing House. All rights reserved. http://www.cnki.net

10-2 汉台区经济

指 标	单 位	2000年	2005年	2006年	2007年	2008年
年底总人口	万人	50.50	53.94	54.22	54.77	54.81
生产总值	亿元	27.23	49.59	55.89	66.18	80.64
第一产业	亿元	2.81	4.59	5.11	6.18	7.97
第二产业	亿元	12.88	20.49	23.07	25.76	31.00
第三产业	亿元	11.54	24.51	27.71	34.24	41.67
# 工业增加值	亿元	9.81	14.52	16.24	17.71	20.25
人均生产总值	元	5436	9358	10340	12150	14718
生产总值指数	上年=100	109.9	111.5	112.1	115.0	115.1
全社会固定资产投资	万元	118595	204500	257700	320100	388604
地方财政收入	万元	15824	16519	20068	25808	32369
地方财政支出	万元	17314	28459	34110	45615	69458
农村居民人均纯收入	元	1878	2639	2850	3236	3878
城镇居民人均可支配收入	元	4308	6671	7704	8981	10602
常用耕地面积	公顷	16855	15381	15548	15497	15579
粮食产量	吨	131827	114229	122689	96184	103464
农林牧渔业总产值	万元	46954	78562	87369	106057	136899
社会消费品零售总额	万元	168963	237000	282300	341300	443600
普通小学专任教师数	人	2358	2254	2375	2351	2309
普通小学在校学生数	人	44500	39700	39800	38300	36000
普通中学专任教师数	人	1835	2049	2108	2179	2167
普通中学在校学生数	人	27900	30700	31200	30900	31000
卫生机构床位数	张	4396	3866	4022	3951	4305
卫生技术人员	人	3588	3937	4025	4192	4438
# 执业(助理)医师	人	1305	1539	1551	1585	1541
注册护师、护士	人	1064	1193	1309	1485	1685

?1994-2018 China Academic Journal Electronic Publishing House. All rights reserved. http://www.cnki.net

社会主要指标

2009年	2010年	2011年	2012年	2013年	2014年	2015年	2016年
53.26	53.52	53.55	53.62	53.76	53.91	54.01	54.03
91.59	111.01	135.47	156.29	177.14	203.89	230.25	252.27
8.35	10.05	12.95	14.44	16.11	16.51	16.97	18.32
33.21	43.13	54.61	63.75	73.68	88.71	101.71	111.74
50.03	57.83	67.91	78.10	87.36	98.67	111.58	122.22
20.06	27.12	34.61	41.34	48.17	58.24	67.03	71.51
16762	20753	25304	29167	32993	37874	42670	46698
114.5	116.1	115.9	114.5	112.1	112.9	111.7	110.8
539100	728000	920900	1200500	1550500	2076000	2556200	3402800
37366	45069	56689	69571	81545	93427	107394	110065
83245	109315	141726	179216	212643	216471	264497	285947
4603	5522	6984	8171	9331	10497	8308	9014
12922	15109	17768	20966	23450	26030	23832	25810
15539	15383	15056	14923	14815	14773	14618	14064
114289	116797	97100	103517	104845	103886	107582	107944
144554	173000	222837	248738	276641	294765	306742	330189
530300	626236	736933	862409	990676	1124390	1273379	1454853
2263	2255	2063	2141	2067	1883	1803	1799
34400	34039	31481	31195	30058	28897	29248	30135
2199	2151	2150	2194	2260	2186	2230	2413
31900	32014	30906	30884	31684	27706	27460	27183
4409	4666	5084	6499	6823	7389	7179	7307
4694	5095	5223	5889	6265	6603	6732	7668
1783	1759	1763	2079	2146	2157	2136	2381
1782	2132	2160	2550	2799	3112	3281	3542

?1994-2018 China Academic Journal Electronic Publishing House. All rights reserved.   http://www.cnki.net

10-3 南郑县经济

指　标	单　位	2000年	2005年	2006年	2007年	2008年
年底总人口	万人	53.89	49.04	49.21	49.24	49.35
生产总值	亿元	21.14	31.89	35.99	42.15	51.62
第一产业	亿元	5.63	5.92	6.66	8.05	10.17
第二产业	亿元	8.37	16.43	18.71	21.80	26.59
第三产业	亿元	7.14	9.55	10.62	12.30	14.85
# 工业增加值	亿元	6.55	13.89	15.87	18.48	22.43
人均生产总值	元	3970	6504	7325	8563	10471
生产总值指数	上年=100	91.6	113.9	111.1	113.3	113.6
全社会固定资产投资	万元	25245	74300	72871	100200	132500
地方财政收入	万元	4938	10387	12574	15089	18634
地方财政支出	万元	13031	26341	34989	48996	81756
农村居民人均纯收入	元	1517	2042	2216	2694	3360
城镇居民人均可支配收入	元				8451	10167
常用耕地面积	公顷	30612	30390	30252	30203	30202
粮食产量	吨	177861	165769	169791	135507	144523
农林牧渔业总产值	万元	84635	107290	118670	143454	181303
社会消费品零售总额	万元	51383	72134	77381	88298	106873
普通小学专任教师数	人	2538	2576	2470	2423	2372
普通小学在校学生数	人	53300	46300	43300	40800	38200
普通中学专任教师数	人	1527	1814	1847	1887	1956
普通中学在校学生数	人	25200	32500	33000	34200	32600
卫生机构床位数	张	775	775	965	965	965
卫生技术人员	人	1283	1121	1125	1096	1110
# 执业(助理)医师	人	597	571	561	553	567
注册护师、护士	人	461	419	421	423	417

?1994-2018 China Academic Journal Electronic Publishing House. All rights reserved. http://www.cnki.net

社会主要指标

2009年	2010年	2011年	2012年	2013年	2014年	2015年	2016年
47.31	47.19	47.12	47.18	47.31	47.43	47.52	47.73
58.71	73.72	94.63	114.90	134.46	154.04	164.88	174.07
10.67	12.81	16.56	18.59	20.71	21.26	22.30	24.06
28.70	38.26	50.16	64.25	78.12	91.67	96.08	96.81
19.34	22.65	27.91	32.06	35.63	41.12	46.50	53.20
23.58	32.03	42.55	55.43	67.96	80.04	82.79	81.38
11849	15602	20067	24370	28459	32519	34730	36550
114.0	115.4	117.6	115.9	113.5	111.9	111.5	108.3
176768	241000	330500	442500	573000	839100	1033900	1247800
22438	27005	35128	44944	55098	64961	71567	72118
105050	127390	166598	218682	235237	264583	293040	311035
4005	4848	6125	7228	8305	9368	8217	8910
12748	14718	17353	20442	22880	25465	23661	25649
30264	30264	30250	30224	30164	30446	29194	29198
159429	165901	138018	147416	149315	148007	148153	148790
192085	228253	295814	330886	367411	392411	412008	443339
126101	153568	176908	206638	237114	268842	304305	348278
2337	2272	1964	1934	1812	1725	1543	1507
36100	33835	31949	29787	28772	28631	28542	28812
2032	2033	2862	3013	2624	2662	2765	2711
33300	30982	30905	30053	29653	29029	28580	28726
965	965	965	965	1090	1090	1551	1588
1094	1231	1298	1449	1740	1779	2192	2090
589	768	729	452	571	583	677	709
430	354	405	413	508	517	676	696

?1994-2018 China Academic Journal Electronic Publishing House. All rights reserved. http://www.cnki.net

10-4 城固县经济

指　　标	单　位	2000年	2005年	2006年	2007年	2008年
年底总人口	万人	50.06	46.59	46.71	46.82	46.91
生产总值	亿元	20.37	32.23	36.94	43.67	54.79
第一产业	亿元	7.75	10.15	12.48	15.07	19.86
第二产业	亿元	6.98	10.06	10.91	12.51	14.94
第三产业	亿元	5.64	12.02	13.55	16.09	19.99
# 工业增加值	亿元	5.96	8.45	9.10	10.36	12.09
人均生产总值	元	4122	7014	7919	9339	11691
生产总值指数	上年=100	107.2	111.1	111.1	113.0	114.5
全社会固定资产投资	万元	37500	82500	108000	147451	202300
地方财政收入	万元	6594	3668	4099	4721	5707
地方财政支出	万元	10790	22384	31774	39768	66003
农村居民人均纯收入	元	1704	2086	2205	2511	3275
城镇居民人均可支配收入	元	4311	6037	6603	7752	10270
常用耕地面积	公顷	28557	25694	24274	24240	24278
粮食产量	吨	175790	153787	165933	133367	145073
农林牧渔业总产值	万元	132389	180892	211046	255088	336215
社会消费品零售总额	万元	55120	73460	84723	99438	122900
普通小学专任教师数	人	3237	2346	2258	2329	2245
普通小学在校学生数	人	53300	39200	38200	38100	37500
普通中学专任教师数	人	1844	1951	1924	2018	2023
普通中学在校学生数	人	26500	32100	31000	30500	30300
卫生机构床位数	张	1018	1157	1178	1195	1225
卫生技术人员	人	1309	1597	1655	1701	1758
# 执业(助理)医师	人	399	553	601	630	655
注册护师、护士	人	219	369	412	437	468

?1994-2018 China Academic Journal Electronic Publishing House. All rights reserved.　http://www.cnki.net

社会主要指标

2009年	2010年	2011年	2012年	2013年	2014年	2015年	2016年
46.47	46.51	46.45	46.51	46.62	46.74	46.83	47.20
61.75	77.53	101.44	117.30	138.29	168.84	192.52	217.22
20.78	25.10	32.27	36.02	40.10	41.58	42.77	45.75
17.55	25.02	35.56	41.58	54.60	76.35	93.72	107.73
23.42	27.42	33.61	39.70	43.59	50.91	56.03	63.75
14.08	20.83	30.30	35.53	47.68	66.97	83.18	95.92
13152	16677	21824	25237	29699	36171	41150	46203
114.6	115.3	115.7	115.4	113.4	113.2	113.8	112.3
284100	412600	545800	707500	916900	1387600	1722400	2183500
6719	9271	12160	16519	20325	23730	28726	30615
79692	112701	153850	182600	211118	227081	280012	306988
4012	4898	6199	7309	8383	9473	8236	8953
12755	14725	17346	20468	22916	25437	23648	25611
24290	24332	24328	24326	24325	24150	24143	23999
150956	157017	131008	139827	141604	140327	141017	141359
355277	426444	549088	612689	679674	726539	748832	800481
146900	177880	207887	243754	280159	318687	361240	413078
2168	2110	2033	1902	1881	1900	1879	1951
36000	34803	34031	30732	27475	27261	27533	28205
2034	2090	2141	2150	2213	2214	2191	2207
30300	29635	29846	26946	26435	25411	24561	24315
1238	1264	1351	1494	1555	1828	1777	1835
1798	1797	1764	1815	1856	1742	1736	1820
689	639	592	625	654	714	752	970
497	478	479	481	685	989	1256	884

?1994-2018 China Academic Journal Electronic Publishing House. All rights reserved.   http://www.cnki.net

10-5　洋县经济

指　　标	单　位	2000年	2005年	2006年	2007年	2008年
年底总人口	万人	44.08	39.42	39.52	39.57	39.68
生产总值	亿元	11.71	20.49	23.26	28.02	34.98
第一产业	亿元	3.25	5.25	6.51	7.97	10.50
第二产业	亿元	4.97	8.25	8.98	10.67	12.97
第三产业	亿元	3.49	6.99	7.77	9.38	11.52
# 工业增加值	亿元	4.43	5.28	5.66	6.74	7.97
人均生产总值	元	2722	5197	5886	7081	8815
生产总值指数	上年=100	133.1	109.7	110.6	113.8	115.5
全社会固定资产投资	万元	21262	103026	115183	130000	191691
地方财政收入	万元	3612	3167	3632	4228	5633
地方财政支出	万元	8450	21313	28054	35632	55451
农村居民人均纯收入	元	1154	1498	1570	1768	2313
城镇居民人均可支配收入	元					9255
常用耕地面积	公顷	27799	28158	28135	28082	27913
粮食产量	吨	159063	163236	174135	140508	154306
农林牧渔业总产值	万元	56354	92423	108113	133752	176483
社会消费品零售总额	万元	24186	36757	41150	48404	59270
普通小学专任教师数	人	2783	2461	2402	2309	2236
普通小学在校学生数	人	42367	36274	35326	31182	30663
普通中学专任教师数	人	1405	1420	1463	1523	1567
普通中学在校学生数	人	27568	26737	26118	25881	25803
卫生机构床位数	张	703	760	768	943	934
卫生技术人员	人	799	911	958	1221	1388
# 执业(助理)医师	人	562	586	602	537	612
注册护师、护士	人	237	305	320	332	376

?1994-2018 China Academic Journal Electronic Publishing House. All rights reserved.　http://www.cnki.net

社会主要指标

2009年	2010年	2011年	2012年	2013年	2014年	2015年	2016年
38.46	38.42	38.37	38.41	38.47	38.54	38.61	38.70
40.08	49.23	62.36	73.76	85.05	94.40	100.26	106.53
10.99	13.21	17.00	19.13	21.28	22.40	23.35	25.05
15.43	19.68	25.52	31.69	38.09	42.47	43.60	43.03
13.66	16.33	19.84	22.94	25.67	29.53	33.31	38.45
9.32	12.31	16.27	21.99	27.21	29.93	29.65	27.90
10090	12778	16242	19212	22125	24516	25966	27525
115.1	116.0	115.6	115.1	113.0	125.0	110.4	108.0
285900	410200	480200	614700	765900	1033000	1281200	964500
7182	20617	14119	17798	16385	19942	24171	24656
75167	10303	141333	169489	188892	221117	247469	276666
3063	3790	4885	5755	6618	7419	8164	8882
12136	14116	16784	19788	22212	24700	23379	25366
27896	27895	27859	27923	27732	27636	27790	27972
167528	176625	147023	155658	157626	156204	157065	157752
186761	224183	288202	322047	356278	380783	398639	427962
70700	85730	100588	118068	135736	154241	174905	223779
2169	2070	1943	2041	1951	1981	1985	1995
30667	27315	26255	25144	23827	23826	23980	23440
1604	1675	1711	1798	1700	1760	1689	1678
25476	25416	25317	25230	23386	22953	22650	22650
1051	1317	1419	1420	1438	1526	1446	1595
1331	1292	1295	1383	1397	1700	1643	1668
607	612	620	696	708	677	678	761
358	360	362	368	375	525	528	536

?1994-2018 China Academic Journal Electronic Publishing House. All rights reserved. http://www.cnki.net

10-6 西乡县经济

指　　标	单　位	2000年	2005年	2006年	2007年	2008年
年底总人口	万人	39.80	35.04	35.11	35.19	35.34
生产总值	亿元	7.36	14.37	16.69	20.35	25.63
第一产业	亿元	2.59	4.44	5.17	6.25	8.40
第二产业	亿元	1.81	3.60	4.16	4.97	5.96
第三产业	亿元	2.96	6.32	7.36	9.14	11.27
# 工业增加值	亿元	1.54	2.35	2.75	3.30	3.81
人均生产总值	元	1844	4084	4765	5783	7267
生产总值指数	上年=100	106.1	110.1	112.7	113.6	114.7
全社会固定资产投资	万元	14009	51800	60325	79000	137900
地方财政收入	万元	3542	1915	2336	3505	4903
地方财政支出	万元	8668	17460	23873	33061	54560
农村居民人均纯收入	元	1019	1289	1427	1698	2360
城镇居民人均可支配收入	元	4015	6517	6975	7816	9501
常用耕地面积	公顷	29215	20972	21213	21479	21684
粮食产量	吨	132214	113674	112727	90189	99147
农林牧渔业总产值	万元	44683	79045	87995	107675	144632
社会消费品零售总额	万元	25660	41079	46363	59857	74509
普通小学专任教师数	人	1685	1472	1421	1391	1376
普通小学在校学生数	人	41549	26988	26716	25775	24835
普通中学专任教师数	人	1107	1311	1355	1404	1429
普通中学在校学生数	人	23301	26501	26576	24583	24096
卫生机构床位数	张	813	895	895	907	907
卫生技术人员	人	1096	1194	1201	1213	1259
# 执业(助理)医师	人	165	411	448	483	518
注册护师、护士	人	124	207	231	256	282

?1994-2018 China Academic Journal Electronic Publishing House. All rights reserved. http://www.cnki.net

社会主要指标

2009年	2010年	2011年	2012年	2013年	2014年	2015年	2016年
34.27	34.20	34.15	34.20	34.28	34.36	34.43	34.46
29.33	36.08	44.31	54.96	65.20	75.38	81.20	90.69
8.79	10.54	13.62	15.26	17.04	17.42	18.47	19.96
7.17	9.27	10.89	15.99	20.82	25.97	27.05	31.66
13.37	16.27	19.80	23.72	27.34	31.99	35.68	39.07
4.53	6.06	6.90	11.46	15.68	18.89	19.05	22.41
8291	10534	12966	16082	19042	21964	23610	26331
114.9	115.5	115.5	115.3	112.8	112.9	112.0	110.1
214200	335255	440000	553500	696500	844900	990300	995800
6767	10061	12582	16006	18681	22069	25458	25802
70061	91916	136382	164500	179588	199500	222550	239611
3065	3802	4905	5788	6662	7541	8180	8875
12465	14536	17065	20068	22405	24914	23612	25605
21793	21797	21746	21711	21861	21950	21950	21950
105037	110108	92056	98264	99524	98670	102531	102853
153199	182510	236337	264662	294714	313732	330407	356141
89201	83033	97476	114828	132255	151181	171520	221146
1328	1287	1181	1130	1067	1203	1193	1239
24575	24361	23907	23918	24125	24312	24522	24496
1407	1414	1589	1541	1574	1334	1337	1344
22018	21821	21458	21917	21863	21467	21530	20841
1216	1289	1314	1397	1985	1824	2048	2080
1288	1188	1355	1404	1548	1630	1704	1798
561	388	434	414	369	448	491	437
358	359	436	452	478	633	755	806

?1994-2018 China Academic Journal Electronic Publishing House. All rights reserved.   http://www.cnki.net

10-7　勉县经济

指　　标	单　位	2000年	2005年	2006年	2007年	2008年
年底总人口	万人	41.89	40.87	41.03	41.02	41.09
生产总值	亿元	12.12	24.67	30.20	38.27	44.24
第一产业	亿元	3.63	4.98	5.83	6.92	9.25
第二产业	亿元	4.74	12.46	16.22	21.37	21.01
第三产业	亿元	3.75	7.23	8.15	9.98	13.99
# 工业增加值	亿元	3.49	10.10	13.59	18.28	16.81
人均生产总值	元	2893	5809	7235	9328	10777
生产总值指数	上年=100	161.9	119.2	116.3	116.1	114.6
全社会固定资产投资	万元	33286	71500	83200	125000	167200
地方财政收入	万元	5222	5173	6926	10098	12814
地方财政支出	万元	9154	20336	26628	39853	95590
农村居民人均纯收入	元	1182	1651	1860	2136	2989
城镇居民人均可支配收入	元					9912
常用耕地面积	公顷	27207	23727	24104	23923	24303
粮食产量	吨	159570	147576	151982	120598	134060
农林牧渔业总产值	万元	60506	93656	105056	126528	168082
社会消费品零售总额	万元	37790	64926	73500	85800	105657
普通小学专任教师数	人	1873	1817	1781	1752	1726
普通小学在校学生数	人	44800	34200	32600	30200	28000
普通中学专任教师数	人	1199	1386	1417	1463	1472
普通中学在校学生数	人	20400	28100	28500	28300	27800
卫生机构床位数	张	1137	1133	1213	1249	1249
卫生技术人员	人	1821	1672	1841	1934	1963
# 执业(助理)医师	人	499	537	575	616	611
注册护师、护士	人	274	312	395	394	387

?1994-2018 China Academic Journal Electronic Publishing House. All rights reserved.　http://www.cnki.net

社会主要指标

2009年	2010年	2011年	2012年	2013年	2014年	2015年	2016年
38.96	38.83	38.78	38.80	38.87	38.95	39.02	39.37
48.85	59.77	78.66	96.59	105.44	111.41	105.76	101.16
9.67	11.66	15.07	16.95	18.89	19.56	20.50	22.14
23.33	30.35	42.17	54.95	58.85	60.11	49.56	38.00
15.84	17.76	21.41	24.69	27.70	31.74	35.70	41.02
18.23	24.16	34.41	46.16	48.87	49.03	37.11	23.89
11877	15366	20270	24900	27150	28632	27126	25807
113.8	115.7	116.2	115.6	113.3	112.8	106.6	102.0
253700	355900	485000	632400	815900	423000	533000	669500
13090	17667	21825	25574	29690	31020	31243	27939
134676	119508	135055	164777	187699	195829	223185	263309
3571	4440	5624	6642	7612	8556	8189	8910
12613	14715	17378	20228	22610	25188	23630	25662
24790	25089	25591	26231	26284	26321	26480	26605
144774	150511	125106	132994	134696	133513	132014	132523
177695	210786	272296	304966	338204	359608	377187	405494
126200	153500	180447	211579	243200	275434	311823	357194
1718	1657	1525	1481	1335	1288	1245	1199
26100	24451	21959	20068	17853	17257	16744	16794
1495	1514	1659	1665	1635	1676	1638	1686
27200	25971	25205	24610	23224	22515	21178	20562
1287	1324	1456	1603	1759	1963	1824	1931
2146	1927	2043	2101	2047	2171	2388	2584
675	508	574	655	588	628	710	698
400	450	489	547	572	623	676	890

?1994-2018 China Academic Journal Electronic Publishing House. All rights reserved. http://www.cnki.net

10-8 宁强县经济

指 标	单 位	2000年	2005年	2006年	2007年	2008年
年底总人口	万人	33.16	31.80	31.89	31.91	31.98
生产总值	亿元	7.45	12.81	15.33	18.40	23.13
第一产业	亿元	3.37	4.69	5.61	6.60	8.35
第二产业	亿元	2.28	3.12	4.20	5.10	6.01
第三产业	亿元	1.80	5.00	5.52	6.70	8.77
# 工业增加值	亿元	1.97	2.18	3.14	3.83	4.37
人均生产总值	元	2252	3831	4610	5728	6944
生产总值指数	上年=100	110.9	113.4	114.8	115.1	113.4
全社会固定资产投资	万元	30632	33504	48508	82500	137119
地方财政收入	万元	2826	3827	2887	3600	4554
地方财政支出	万元	7151	17566	23620	31083	70680
农村居民人均纯收入	元	992	1410	1519	1823	2552
城镇居民人均可支配收入	元	3434	5698	6321	8177	9771
常用耕地面积	公顷	24734	20699	20311	20527	20758
粮食产量	吨	94324	77624	79315	79449	85404
农林牧渔业总产值	万元	53517	78295	89512	105388	138088
社会消费品零售总额	万元	19615	39388	45061	53114	65298
普通小学专任教师数	人	1381	1395	1436	1478	1525
普通小学在校学生数	人	38607	31850	30756	29845	28398
普通中学专任教师数	人	747	988	1071	1135	1168
普通中学在校学生数	人	13757	19902	20363	20522	20937
卫生机构床位数	张	502	541	504	612	751
卫生技术人员	人	398	500	502	627	1209
# 执业(助理)医师	人	278	380	382	329	441
注册护师、护士	人	83	108	110	123	277

?1994-2018 China Academic Journal Electronic Publishing House. All rights reserved. http://www.cnki.net

社会主要指标

2009年	2010年	2011年	2012年	2013年	2014年	2015年	2016年
30.98	30.89	30.85	30.85	30.86	30.88	30.94	30.88
26.33	32.05	41.65	48.50	55.85	61.94	64.12	71.54
8.72	10.53	13.58	15.20	16.95	17.41	18.19	19.49
6.78	8.48	12.43	14.78	17.64	20.07	18.25	21.15
10.83	13.03	15.64	18.52	21.26	24.46	27.69	30.90
4.77	6.02	9.35	11.31	13.68	14.37	11.78	13.77
8217	10359	13493	15719	18100	20063	20746	23144
114.5	115.6	115.8	114.5	112.7	112.7	112.2	110.1
231400	331000	315000	408200	541600	717400	889700	999600
5782	7339	9121	11701	13808	16072	17418	15544
142687	119583	112026	157816	151795	169701	186927	200081
3146	3899	4914	5754	6548	7373	8068	8746
12468	14562	17183	20018	22489	24985	23602	25599
20960	21136	21136	21256	21259	21263	21267	21249
88548	92005	77003	82208	83255	82549	85233	85656
145949	176280	227324	254727	283862	301567	315377	337556
77400	93914	109121	127535	146315	165795	187705	213193
1556	1540	1478	1354	1359	1243	1100	1090
27007	25095	23412	20370	15954	15373	14910	14830
1257	1283	1266	1356	1236	1405	1464	1569
21679	21407	21287	18642	16398	16437	16095	15658
813	926	1092	1261	1332	1355	1379	1280
1275	1309	1332	1368	1482	1487	1506	1517
470	340	346	428	461	439	431	418
282	287	292	369	420	463	467	493

?1994-2018 China Academic Journal Electronic Publishing House. All rights reserved.   http://www.cnki.net

10-9 略阳县经济

指 标	单 位	2000年	2005年	2006年	2007年	2008年
年底总人口	万人	20.21	20.08	20.23	20.25	20.33
生产总值	亿元	8.99	17.94	20.04	22.61	27.96
第一产业	亿元	1.47	2.39	2.64	3.14	4.09
第二产业	亿元	4.33	8.42	9.41	10.07	12.60
第三产业	亿元	3.19	7.13	7.99	9.40	11.27
# 工业增加值	亿元	3.60	6.65	7.41	7.67	9.73
人均生产总值	元	4467	8909	9933	11171	13778
生产总值指数	上年=100	107.8	110.5	111.0	113.7	113.5
全社会固定资产投资	万元	16270	69787	114957	126170	159111
地方财政收入	万元	4219	5959	6565	7560	9109
地方财政支出	万元	7353	16059	19005	27086	55315
农村居民人均纯收入	元	1197	1668	1756	1942	2550
城镇居民人均可支配收入	元	4326	6322	6825	7961	9978
常用耕地面积	公顷	10043	9957	9945	9986	10091
粮食产量	吨	64853	49279	40842	43507	47970
农林牧渔业总产值	万元	25816	41537	46925	55011	71629
社会消费品零售总额	万元	20275	36305	41478	49753	61531
普通小学专任教师数	人	768	924	1005	995	1009
普通小学在校学生数	人	23500	15800	14900	13400	11800
普通中学专任教师数	人	524	639	617	628	622
普通中学在校学生数	人	7000	8700	11000	10500	9800
卫生机构床位数	张	753	785	785	744	743
卫生技术人员	人	875	623	623	775	779
# 执业(助理)医师	人	507	250	205	342	301
注册护师、护士	人	221	165	149	211	209

?1994-2018 China Academic Journal Electronic Publishing House. All rights reserved. http://www.cnki.net

社会主要指标

2009年	2010年	2011年	2012年	2013年	2014年	2015年	2016年
20.19	20.17	20.19	20.19	20.19	20.18	20.22	20.02
32.05	38.74	48.54	60.23	65.87	63.20	50.46	55.94
4.27	5.18	6.66	7.46	8.31	8.61	8.94	9.55
14.49	18.08	24.61	32.94	35.87	30.29	15.74	18.63
13.30	15.48	17.27	19.82	21.69	24.30	25.78	27.76
11.21	13.77	19.23	26.75	29.11	23.95	8.79	10.84
15743	19195	24051	29831	32621	31308	24983	27805
115.2	114.7	115.2	115.8	110.0	102.0	86.8	107.9
236200	325000	319200	419200	399400	313000	314200	385000
11580	12386	14870	18091	19053	17016	11088	11977
126114	82565	91625	120616	130816	131970	140726	181022
3117	3804	4824	5654	6468	7277	8043	8714
12660	14534	17107	19930	22270	24675	23357	25282
9831	9831	10074	10074	10072	10046	10046	10046
50346	52462	44004	46939	47532	47119	48168	48421
75617	91202	117544	131357	145976	155719	162582	173098
73601	89927	104460	122063	139827	157943	178908	203193
1016	965	915	912	823	858	877	864
10600	10150	9488	8952	8220	7895	7851	7805
636	650	508	665	703	695	637	606
9700	6289	5863	7459	6884	6854	6470	6161
750	896	997	749	702	874	1201	1206
752	720	775	781	624	710	897	990
315	283	293	330	300	202	195	180
195	192	233	166	166	192	301	269

?1994-2018 China Academic Journal Electronic Publishing House. All rights reserved.   http://www.cnki.net

10-10 镇巴县经济

指　　标	单　位	2000年	2005年	2006年	2007年	2008年
年底总人口	万人	27.37	24.17	24.11	24.03	24.11
生产总值	亿元	5.36	9.42	10.95	13.03	16.71
第一产业	亿元	2.61	3.62	4.36	5.34	7.26
第二产业	亿元	1.13	1.54	1.64	1.95	2.44
第三产业	亿元	1.62	4.26	4.95	5.74	7.02
# 工业增加值	亿元	0.80	0.85	0.86	1.03	1.24
人均生产总值	元	1972	3426	4567	5415	6939
生产总值指数	上年=100	111.7	109.5	110.3	111.4	115.1
全社会固定资产投资	万元	18001	36010	41051	51200	69581
地方财政收入	万元	2647	1311	1440	1700	2218
地方财政支出	万元	6965	15192	18677	28724	39081
农村居民人均纯收入	元	1030	1306	1432	1686	2358
城镇居民人均可支配收入	元	3643	5930	5648	7320	9007
常用耕地面积	公顷	22281	22573	22481	22501	22879
粮食产量	吨	103979	95291	99699	99585	88944
农林牧渔业总产值	万元	33691	62772	72776	88540	117931
社会消费品零售总额	万元	10742	32574	37486	44927	55560
普通小学专任教师数	人	1203	1141	1223	1206	1193
普通小学在校学生数	人	32500	25500	24600	23500	22300
普通中学专任教师数	人	559	701	831	836	862
普通中学在校学生数	人	11400	14200	14400	14900	15000
卫生机构床位数	张	631	631	631	679	679
卫生技术人员	人	849	868	875	893	915
# 执业(助理)医师	人	202	213	218	231	249
注册护师、护士	人	137	146	149	152	167

?1994-2018 China Academic Journal Electronic Publishing House. All rights reserved.   http://www.cnki.net

社会主要指标

2009年	2010年	2011年	2012年	2013年	2014年	2015年	2016年
24.77	24.69	24.71	24.73	24.78	24.80	24.85	24.87
19.46	24.87	33.66	40.50	48.14	56.42	58.30	65.17
7.49	8.91	11.49	12.95	14.18	15.17	15.82	16.93
2.97	4.98	8.63	11.38	15.40	19.87	17.49	19.72
9.00	10.98	13.54	16.17	18.56	21.38	24.99	28.51
1.50	3.22	6.43	8.84	12.52	16.56	13.75	15.50
8057	10260	13626	16383	19447	22761	23487	26214
114.2	115.1	115.2	114.4	113.2	113.0	112.2	108.9
93600	131742	193443	259400	340400	467200	610200	750100
3034	4500	6128	8085	9698	11270	12594	10609
56162	92928	99108	129086	148470	157028	178736	190789
3013	3715	4687	5470	6232	7042	8002	8698
11986	13850	16252	19096	21495	23924	23348	25216
22880	22946	23693	23674	23650	23631	23620	23699
96245	97583	81011	86517	87623	86865	89515	89981
124614	148550	191925	215886	240308	257282	268989	287859
66401	80779	94917	111814	128378	145780	165005	187537
1213	1169	1061	1084	1171	996	995	988
21100	19979	18837	17781	17542	17597	18032	18229
880	889	867	860	870	866	874	820
14900	15162	15063	14560	13633	13124	12807	12572
679	631	676	766	841	841	891	1333
935	772	785	943	965	985	1013	930
265	229	349	641	281	308	320	308
202	202	207	287	355	420	453	458

?1994-2018 China Academic Journal Electronic Publishing House. All rights reserved. http://www.cnki.net

10-11 留坝县经济

指标	单位	2000年	2005年	2006年	2007年	2008年
年底总人口	万人	4.59	4.31	4.35	4.39	4.41
生产总值	亿元	1.50	2.45	2.80	3.26	4.15
第一产业	亿元	0.53	0.72	0.84	1.01	1.36
第二产业	亿元	0.41	0.57	0.64	0.72	0.89
第三产业	亿元	0.56	1.17	1.31	1.52	1.89
# 工业增加值	亿元	0.30	0.27	0.31	0.33	0.39
人均生产总值	元	3268	5693	6433	7419	9401
生产总值指数	上年=100	115.3	112.4	110.8	112.3	114.4
全社会固定资产投资	万元	13812	24296	12275	21300	28200
地方财政收入	万元	912	570	361	461	600
地方财政支出	万元	2472	5461	7432	10450	15101
农村居民人均纯收入	元	1180	1636	1716	1891	2451
城镇居民人均可支配收入	元					8543
常用耕地面积	公顷	2897	2974	2964	2961	2955
粮食产量	吨	13882	11268	10917	9421	10669
农林牧渔业总产值	万元	7690	12240	14198	17206	23211
社会消费品零售总额	万元	11626	7651	8799	10312	12800
普通小学专任教师数	人	242	222	250	263	251
普通小学在校学生数	人	5148	3789	3706	3541	3180
普通中学专任教师数	人	148	161	171	183	185
普通中学在校学生数	人	2872	2747	2734	2683	2486
卫生机构床位数	张	182	182	202	202	222
卫生技术人员	人	194	190	185	187	183
# 执业(助理)医师	人	98	101	104	110	116
注册护师、护士	人	52	49	50	52	55

?1994-2018 China Academic Journal Electronic Publishing House. All rights reserved. http://www.cnki.net

社会主要指标

2009年	2010年	2011年	2012年	2013年	2014年	2015年	2016年
4.37	4.34	4.34	4.34	4.34	4.34	4.35	4.34
4.69	5.78	7.21	8.78	10.07	11.42	12.77	14.11
1.43	1.72	2.21	2.48	2.77	2.88	2.99	3.17
1.01	1.29	1.63	2.26	2.57	2.99	3.21	3.73
2.26	2.77	3.37	4.04	4.73	5.55	6.57	7.21
0.41	0.58	0.76	1.15	1.31	1.51	1.51	1.78
10603	12970	16613	20238	23209	26323	29392	32510
114.8	115.0	112.9	115.2	112.0	112.1	113.5	109.3
41300	46500	35500	48300	73400	157700	213200	265700
938	1539	2036	2886	3533	4346	6877	7833
19603	32800	42789	51533	53291	61465	70330	75984
3028	3728	4700	5471	6231	7047	8015	8712
11832	13696	16044	18948	21325	23649	23242	25125
2963	2958	3080	3086	3086	3066	3061	3131
13005	13439	11013	11748	11897	11791	12072	12113
24558	29443	37885	42369	47257	50856	52745	56014
15360	18519	21711	25418	29098	33019	37306	42326
229	231	199	193	165	194	181	153
2895	2889	2437	2027	1731	1893	1929	1724
188	172	167	200	171	175	198	178
2586	2669	1689	2027	2047	2562	1855	1768
222	204	230	202	204	225	204	174
179	213	211	284	281	176	278	280
122	99	99	93	89	90	85	94
59	56	58	81	66	60	58	95

?1994-2018 China Academic Journal Electronic Publishing House. All rights reserved. http://www.cnki.net

10-12 佛坪县经济

指　　标	单　位	2000年	2005年	2006年	2007年	2008年
年底总人口	万人	3.44	3.20	3.23	3.29	3.32
生产总值	亿元	1.05	1.30	1.59	1.83	2.36
第一产业	亿元	0.28	0.27	0.33	0.40	0.53
第二产业	亿元	0.23	0.37	0.42	0.49	0.64
第三产业	亿元	0.54	0.66	0.83	0.93	1.19
# 工业增加值	亿元	0.15	0.09	0.11	0.12	0.15
人均生产总值	元	3109	3881	4916	5565	7141
生产总值指数	上年=100	109.4	127.5	113.0	120.2	119.5
全社会固定资产投资	万元	2655	9200	13300	17950	22100
地方财政收入	万元	704	469	593	877	571
地方财政支出	万元	2453	4913	6640	10071	13962
农村居民人均纯收入	元	1150	1227	1450	1675	2012
城镇居民人均可支配收入	元				6850	8340
常用耕地面积	公顷	2247	1622	1624	1629	1706
粮食产量	吨	12032	8126	7506	6940	7597
农林牧渔业总产值	万元	5237	5490	6225	7598	10089
社会消费品零售总额	万元	3815	4806	5695	6720	8198
普通小学专任教师数	人	188	207	207	196	184
普通小学在校学生数	人	3932	3176	2918	2607	2344
普通中学专任教师数	人	102	111	124	123	130
普通中学在校学生数	人	1872	2565	2476	2320	2183
卫生机构床位数	张	135	185	185	175	150
卫生技术人员	人	188	195	178	186	148
# 执业(助理)医师	人				65	47
注册护师、护士	人				23	23

?1994-2018 China Academic Journal Electronic Publishing House. All rights reserved. http://www.cnki.net

社会主要指标

2009年	2010年	2011年	2012年	2013年	2014年	2015年	2016年
3.05	3.01	3.00	3.01	3.01	3.02	3.03	3.02
2.67	3.23	4.09	4.83	5.79	6.65	7.32	8.56
0.56	0.68	0.88	0.99	1.11	1.18	1.25	1.32
0.78	0.96	1.29	1.54	2.00	2.34	2.45	2.84
1.34	1.59	1.92	2.30	2.69	3.14	3.63	4.40
0.18	0.23	0.36	0.44	0.69	0.79	0.66	0.79
8039	10673	13621	16063	19251	22050	24216	28311
115.1	114.9	115.0	114.0	114.0	113.1	113.6	111.5
28100	38000	47700	62400	92200	191600	249700	234600
739	1001	1352	1759	2246	3117	3897	4418
16795	27207	35815	46501	53051	55700	66278	76669
3001	3716	4698	5473	6272	7050	8030	8745
11633	13691	16040	18911	21309	23674	23257	25094
1788	1801	1800	1833	1833	1871	1883	1854
8532	9019	8016	8550	8658	8581	9063	9099
10677	12781	16335	18252	20379	21795	23135	24469
9777	11942	13958	16346	18724	21170	23919	27112
174	169	175	179	158	155	157	155
2092	1861	1795	1707	1571	1573	1533	1524
125	127	117	119	124	126	122	123
1995	1811	1640	1520	1414	1289	1192	1164
166	175	175	175	175	175	222	222
192	152	168	180	199	210	223	245
62	37	47	64	65	55	43	46
28	29	31	38	53	73	78	86

?1994-2018 China Academic Journal Electronic Publishing House. All rights reserved. http://www.cnki.net
