# Supplementary material for: A study on the tourism efficiency of tourism destination based on DEA model: A case of ten cities in Shaanxi province
Source: PLoS One. 2024 Jan 19;19(1):e0296660. doi: 10.1371/journal.pone.0296660 (PMC10798521; doi:10.1371/journal.pone.0296660)
Supplement: S1 File — (ZIP) [file pone.0296660.s001.zip › Supporting information/Statistical yearbook/Shangluo.caj]

十三、商洛市

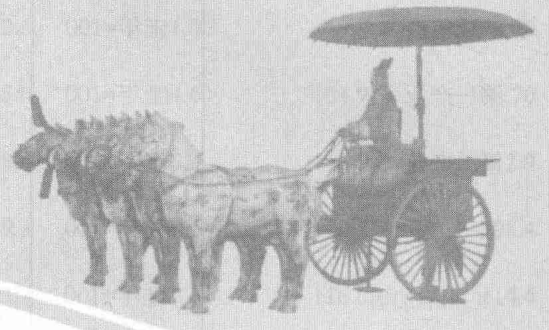

资料整理：王　丹　金文娟

?1994-2018 China Academic Journal Electronic Publishing House. All rights reserved.　http://www.cnki.net

13-1　商洛市经济

指　　标	单 位	2000年	2005年	2006年	2007年	2008年
年底总人口	万人	236.72	237.91	237.73	238.45	238.96
人口自然增长率	‰		3.06	3.07	3.04	3.01
年底总户数	万户	62.20	68.78	69.66	69.30	70.74
生产总值	亿元	56.35	114.43	137.77	160.40	197.45
第一产业	亿元	16.66	25.43	28.31	33.87	44.57
第二产业	亿元	20.08	37.25	46.64	55.19	71.18
第三产业	亿元	19.61	51.75	62.82	71.34	81.70
# 工业增加值	亿元	8.22	17.87	20.98	26.69	35.58
人均生产总值	元	2382	4800	5787	6737	8272
生产总值指数	上年=100	111.2	109.9	110.8	112.8	115.8
第一产业	上年=100	104.5	107.7	106.5	106.3	107.2
第二产业	上年=100	118.5	110.4	111.2	114.6	119.9
第三产业	上年=100	110.8	110.6	112.6	114.5	116.6
# 工业增加值	上年=100	115.0	110.3	112.7	116.3	118.7
人均生产总值指数	上年=100	110.9	109.6	110.5	112.5	115.5
非公有制经济增加值	亿元		44.28	62.00	75.15	93.05
文化产业增加值	亿元					
单位GDP能耗	吨标准煤/万元		1.080	1.066	1.024	0.975
单位GDP能耗比上年增长	%			-1.30	-3.96	-4.78
就业人员	万人	103.20	107.56	107.80	108.69	110.96
城镇单位就业人员	万人	10.37	10.83	10.75	11.99	11.89
# 国有单位	万人	8.45	8.52	8.70	9.14	8.95
集体单位	万人	0.68	0.60	0.64	0.75	0.78
# 在岗职工人数	万人	9.97	10.83	10.75	11.41	11.25
城镇单位就业人员平均工资	元					
城镇单位在岗职工平均工资	元	6185	10426	11619	14431	18675

?1994-2018 China Academic Journal Electronic Publishing House. All rights reserved.　http://www.cnki.net

社会主要指标

2009年	2010年	2011年	2012年	2013年	2014年	2015年	2016年
234.61	234.29	233.62	234.19	234.61	235.08	235.74	237.17
2.99	3.18	3.15	3.35	3.43	3.28	3.27	3.77
72.07	73.03	74.77	76.06	78.24	81.10	81.03	82.33
224.47	285.90	362.95	423.31	510.88	574.99	618.52	692.13
46.65	58.05	70.61	79.43	85.20	90.82	91.75	96.65
83.75	117.82	163.03	195.14	258.97	298.39	318.60	364.88
94.07	110.03	129.31	148.74	166.71	185.78	208.17	230.60
40.09	64.49	96.38	118.96	169.86	197.97	210.65	247.75
9383	12194	15513	18097	21795	24484	26274	29271
114.1	114.9	115.1	114.8	112.6	111.0	111.2	110.00
106.4	106.5	106.5	105.9	105.0	104.6	104.9	103.70
115.8	119.5	121.2	120.7	118.0	115.2	113.5	112.9
115.8	114.7	113.1	112.5	109.3	108.1	110.7	108.4
107.6	122.6	124.4	128.2	123.2	117.8	116.0	114.6
113.8	115.3	115.3	114.9	112.3	110.7	111.0	109.5
107.56	138.93	180.09	211.78	260.15	296.60	328.45	375.4
					10.81	11.91	13.34
0.926	0.596	0.575	0.555	0.537	0.520	0.505	0.413
-5.01	-3.20	-3.51	-3.38	-3.32	-3.08	-2.97	-3.64
111.44	110.75	111.33	109.24	110.00	114.53	111.75	129.73
12.48	12.60	13.88	14.70	18.85	19.17	19.71	19.82
9.14	9.15	9.88	10.60	11.74	11.60	11.90	12.02
0.94	0.95	0.98	1.03	1.06	0.92	1.00	0.96
11.74	11.96	12.89	13.37	16.59	16.80	17.49	17.62
			33088	34832	37870	42821	45324
22203	26171	30287	34769	37124	39946	44741	47292

?1994-2018 China Academic Journal Electronic Publishing House. All rights reserved. http://www.cnki.net

13-1 续表 1

指 标	单 位	2000年	2005年	2006年	2007年	2008年
全社会固定资产投资	亿元	23.27	40.32	99.97	133.19	164.65
# 房地产开发	亿元	0.21	1.77	1.92	2.32	5.45
商品房销售面积	万平方米	2.55	11.82	13.08	13.66	21.49
# 住宅	万平方米	2.55	10.63	12.87	13.56	21.49
地方财政收入	亿元	2.78	2.46	3.37	4.94	6.82
地方财政支出	亿元	7.73	15.40	20.30	32.16	47.59
金融机构人民币各项存款余额	亿元	67.35	132.03	166.95	205.16	256.32
金融机构人民币各项贷款余额	亿元	54.07	66.54	72.52	86.01	97.41
农村居民人均纯收入	元	1128	1513	1609	1850	2491
城镇居民人均可支配收入	元	4551	7038	7770	8870	10444
城市人均公园绿地面积	平方米			7.8	8.5	8.9
城市人均道路面积	平方米		9.4	9.5	11.3	11.9
城市用水普及率	%		85.7	89.2	90.1	85.7
城市用气普及率	%		67.2	91.7	93.3	85.0
常用耕地面积	千公顷	146.46	129.26	128.01	129.85	131.05
农林牧渔业总产值	亿元	29.49	43.33	47.99	57.80	76.09
农作物总播种面积	千公顷	307.93	302.04	303.95	258.31	271.02
# 粮食作物	千公顷	269.44	238.59	242.31	201.48	209.89
粮食产量	万吨	74.72	70.62	74.94	50.13	59.46
棉花产量	吨	48	25	26	17	20
油料产量	吨	12555	11545	13036	12125	16084
蔬菜产量	吨	171000	312773	328720	298579	311969

?1994-2018 China Academic Journal Electronic Publishing House. All rights reserved. http://www.cnki.net

2009年	2010年	2011年	2012年	2013年	2014年	2015年	2016年
220.34	290.07	308.36	391.60	496.16	625.16	767.69	935.55
5.94	8.22	10.98	14.20	17.16	18.35	19.35	20.06
27.41	34.56	43.60	52.95	66.07	75.61	76.72	72.69
27.26	32.02	41.97	45.67	62.15	73.01	71.20	64.82
8.96	12.01	16.49	21.50	25.68	29.04	31.79	26.77
65.07	90.20	106.78	134.03	146.34	163.14	181.52	192.98
319.96	382.91	446.66	518.65	589.36	671.44	801.50	872.46
131.91	155.47	187.49	222.12	248.08	285.12	337.70	391.54
3002	3605	4586	5425	6223	7011	7706	8358
12857	14811	17344	19998	22257	21613	23509	25468
10.2	10.0	11.4	9.9	9.4	9.9	10.4	7.06
13.7	13.9	14.1	12.0	11.4	11.2	11.8	8.21
96.6	84.7	76.0	68.0	90.5	92.1	100.0	99.75
75.2	59.6	61.4	90.5	93.0	48.5	63.8	50.10
131.58	132.27	133.23	133.60	133.43	133.39	133.59	133.61
81.56	100.35	124.76	139.55	156.02	165.92	168.24	178.07
282.49	286.20	275.25	279.22	283.81	282.88	285.95	278.79
216.66	221.86	208.44	209.81	209.92	209.53	206.93	203.03
67.65	71.05	59.80	64.30	65.07	52.20	61.44	61.88
17	12	12	2	2	2	2	2
18482	18482	21603	23427	23716	21609	24033	25678
369858	440049	410457	425026	454755	471300	499048	512294

?1994-2018 China Academic Journal Electronic Publishing House. All rights reserved.   http://www.cnki.net

13-1 续表 2

指 标	单 位	2000年	2005年	2006年	2007年	2008年
水果产量	吨	41791	53995	56088	56381	61865
# 苹果	吨	5463	8778	9137	9235	10081
肉类产量	吨	88259	128651	136566	81273	93051
# 猪牛羊肉	吨	84957	122478	130189	74071	87235
奶类产量	吨	1036	978	967	1056	1270
# 牛奶	吨	524	812	772	768	852
禽蛋产量	吨	29504	37315	37861	33329	37802
水产品产量	吨	1200	1444	1363	1328	1385
规模以上工业企业单位数	个	71	100	80	87	113
规模以上工业总产值	亿元	11.47	32.76	41.39	60.08	74.43
原煤产量	万吨	26.20	15.00	15.94	13.24	10.55
发电量	亿千瓦小时	0.69	0.78	1.36	1.30	1.31
水泥产量	万吨	52.25	68.50	111.00	161.00	202.90
建筑业企业单位数	个	68	51	52	52	52
建筑业企业年末从业人员	万人	1.14	1.76	1.66	2.18	2.43
建筑业总产值	亿元	3.76	11.30	13.26	19.30	28.67
房屋建筑施工面积	万平方米	84.46	184.67	187.84	216.30	240.61
房屋建筑竣工面积	万平方米	43.83	98.00	99.25	122.42	136.65
公路里程	公里	4899	6167	10873	9268	9943
# 等级公路	公里	4153	5546	4863	7395	8634
# 高速公路	公里				13	247
民用汽车拥有量	辆	6511	11022	24982	33428	36537
# 私人汽车	辆	3420	4715	21609	30629	28309
邮电业务总量	亿元	1.90	7.47	10.99	14.65	17.96
邮政业务总量	亿元	0.27	0.90	1.17	1.35	1.52
电信业务总量	亿元	1.64	6.57	9.83	13.30	16.43

?1994-2018 China Academic Journal Electronic Publishing House. All rights reserved. http://www.cnki.net

2009年	2010年	2011年	2012年	2013年	2014年	2015年	2016年
65339	64174	69847	69781	69633	67201	66796	64231
10652	10741	9701	9652	9622	9249	8419	7169
110474	124291	134281	141201	148727	152654	151939	149166
102379	115005	123428	129286	135201	139882	137691	136910
1509	1647	1660	1765	1571	1395	1645	1463
1127	1530	1598	1615	1353	1162	952	962
45125	51488	61961	70055	74114	72852	75109	76804
1562	1896	1900	2136	2371	2880	3566	3960
127	136	120	150	173	207	221	227
104.56	176.99	265.07	365.04	479.93	634.11	793.37	935.31
11.71	11.62	6.37	11.37				
2.40	2.93	4.03	3.08	0.70	3.29	1.06	1.83
229.23	215.60	210.19	320.33	379.14	334.83	342.57	402.00
54	54	53	54	66	73	73	71
2.43	3.10	4.00	3.60	5.08	5.17	5.09	4.65
39.01	59.39	82.20	112.55	112.26	114.69	126.67	142.56
294.77	320.97	412.31	588.42	644.24	580.54	567.17	649.13
151.20	190.52	245.32	315.77	321.24	303.49	350.95	351.77
11759	11871	12107	12988	13237	13482	13595	13776
10852	11198	11520	11944	12142	12373	12496	12667
341	347	315	364	364	364	409	410
43136	49643	55004	60107	66059	66634	69030	77467
34463	40301	45059	49911	55648	56253	59172	68014
17.99	7.77	10.89	10.94	12.10	16.28	22.10	35.61
1.67	1.32	1.49	1.18	1.32	1.50	1.75	2.31
16.32	6.45	9.40	9.77	10.78	14.77	20.35	33.30

?1994-2018 China Academic Journal Electronic Publishing House. All rights reserved. http://www.cnki.net

13-1 续表 3

指 标	单 位	2000年	2005年	2006年	2007年	2008年
固定电话用户	万户	11.64	30.29	36.98	39.93	40.18
移动电话用户	万户	2.38	23.42	32.84	46.31	59.88
互联网宽带用户	万户	0.25	4.42	2.95	3.91	7.76
限额以上企业数	个					
批发业	个					
零售业	个					
住宿业	个					
餐饮业	个					
社会消费品零售总额	亿元	19.91	35.97	40.01	46.22	56.70
进出口总额	万美元		122	317	882	2674
# 出口	万美元		122	317	315	1077
实际外商直接投资额	万美元			278	310	2633
入境旅游人数	万人次	1.21	0.23	0.25	0.25	0.60
# 外国人	万人次	1.00	0.10	0.10	0.11	0.60
国际旅游外汇收入	万美元	273	6	9	9	23
国内旅游人数	万人次	50	73	117	260	453
国内旅游收入	亿元	0.75	1.46	2.40	5.33	10.46
星级饭店数	个		10	10	10	10
幼儿园数	所	70	88	95	116	106
在园儿童数	万人	2.50	2.26	2.23	1.91	2.50
普通小学学校数	所	4237	2479	2228	2112	1928
普通小学专任教师数	人	12853	11447	11339	11263	11068
普通小学在校学生数	万人	36.51	24.80	23.06	21.65	20.02
普通中学学校数	所	178	187	187	187	187
普通中学专任教师数	人	7074	9905	10204	10590	10771
普通中学在校学生数	万人	12.24	19.43	19.57	19.05	18.15
卫生机构数	个	360	324	321	309	277
卫生机构床位数	张	4009	4432	4504	5055	5786
卫生技术人员	人	5898	6236	6286	6682	6905
# 执业(助理)医师	人	3615	3007	3083	3208	2840
注册护师、护士	人	1082	1226	1259	1434	1841

?1994-2018 China Academic Journal Electronic Publishing House. All rights reserved. http://www.cnki.net

2009年	2010年	2011年	2012年	2013年	2014年	2015年	2016年
38.19	35.67	33.84	31.08	29.23	26.96	24.74	22.79
76.76	85.32	99.55	106.41	113.12	120.22	128.86	144.68
7.25	9.46	12.33	13.55	16.14	17.36	19.58	26.36
44	65	73	97	115	132	158	178
5	10	12	15	18	22	26	26
16	24	29	45	59	68	82	100
19	26	24	26	27	27	30	30
4	5	8	11	11	15	20	22
66.79	79.19	92.18	107.28	121.82	136.92	154.66	174.93
10680	30754	33666	7728	6245	22.47(亿元)	18.72(亿元)	14.42(亿元)
431	5186	395	646	4758	18.32(亿元)	15.28(亿元)	9.85(亿元)
4014	5867	6600	8461	7010	473	152	
1.73	1.20	1.23	1.41	1.60	1.65	1.72	1.82
1.73	0.58	0.66	0.69	0.80	0.87	0.90	0.94
78	76	98	145	172	183	194	209.60
734	1270	1703	2288	2765	3006	3363	3734.77
23.10	48.26	68.12	102.50	135.43	153.29	174.87	199.81
10	12	12	19	19	19	20	18
129	181	296	332	374	409	432	442
2.76	3.02	5.46	6.91	7.84	7.74	8.34	8.82
1614	1306	1130	843	681	513	428	415
10880	10823	9894	9568	9233	9168	8734	8429
18.73	17.75	17.15	16.36	15.63	13.14	13.41	14.23
186	183	180	172	171	160	163	162
10931	11003	11644	11002	11692	11504	10527	11288
17.29	16.44	15.65	14.91	14.44	11.53	10.84	10.85
258	261	2616	2662	2980	3007	3119	3028
6071	6701	7998	9060	10270	10991	11765	12282
7104	7933	9401	9879	11095	11771	11892	13031
2856	2909	3217	3278	3546	3757	3627	3829
1969	2177	2713	2972	3565	3865	3996	4499

?1994-2018 China Academic Journal Electronic Publishing House. All rights reserved. http://www.cnki.net

13-2 商州区经济

指 标	单 位	2000年	2005年	2006年	2007年	2008年
年底总人口	万人	54.19	54.56	54.52	54.69	54.78
生产总值	亿元	10.67	26.37	30.63	35.55	46.38
第一产业	亿元	2.60	3.84	4.62	5.64	7.33
第二产业	亿元	4.10	8.97	11.49	12.93	17.56
第三产业	亿元	3.96	13.56	14.52	16.98	21.49
# 工业增加值	亿元	2.04	4.06	5.01	5.59	7.51
人均生产总值	元	1977	4804	5546	6472	8474
生产总值指数	上年=100	114.8	110.2	114.3	113.4	116.0
全社会固定资产投资	万元	56957	93329	105395	149400	223800
地方财政收入	万元	4845	4258	5807	8236	11282
地方财政支出	万元	11992	22003	30106	44798	68566
农村居民人均纯收入	元	1097	1451	1570	1806	2384
城镇居民人均可支配收入	元	4437	6916	7875	9104	11007
常用耕地面积	公顷	25647	23059	22629	22583	22263
粮食产量	吨	124732	126257	140818	94821	115984
农林牧渔业总产值	万元	44325	66277	77878	95096	122946
社会消费品零售总额	万元	51691	90028	100045	114218	139697
普通小学专任教师数	人	3058	2907	2861	2812	2682
普通小学在校学生数	人	78528	53418	50933	49609	46696
普通中学专任教师数	人	1676	2103	2212	2270	2288
普通中学在校学生数	人	30407	39366	41573	40111	37802
卫生机构床位数	张	1456	1371	1419	1553	1724
卫生技术人员	人	1680	1750	1735	1916	2277
# 执业(助理)医师	人	744	818	798	863	787
注册护师、护士	人		496	545	578	734

?1994-2018 China Academic Journal Electronic Publishing House. All rights reserved. http://www.cnki.net

社会主要指标

2009年	2010年	2011年	2012年	2013年	2014年	2015年	2016年
53.92	53.19	53.04	53.17	53.29	53.37	53.52	53.86
52.53	67.59	83.03	98.61	108.35	117.65	121.90	136.09
7.69	9.37	11.15	12.02	13.50	13.62	13.20	13.95
19.93	29.07	37.54	44.19	48.09	51.60	53.24	60.67
24.92	29.15	34.34	42.40	46.77	52.43	55.46	61.47
8.01	15.08	20.36	24.16	28.60	30.70	31.10	36.68
9567	12280	15632	18568	20356	22029	22808	25347
114.3	115.1	115.7	114.3	112	110.0	110.9	109.6
418700	513300	696695	814400	1031900	1304060	1509300	1801600
15603	20271	26407	33502	40155	46010	50326	42900
91649	108157	141135	198269	210506	242143	271961	310358
2993	3594	4584	5414	6204	7066	7614	8230
13281	15539	18194	21100	23558	26196	24010	26016
21913	21714	21609	21578	21378	21230	20972	20967
121716	118523	99556	107590	108103	81183	106420	105291
131770	156501	190741	205123	230111	243724	241200	257463
164632	195321	227298	264163	300428	338138	381485	429545
2646	2565	2359	2318	2250	2142	1927	1890
43856	41076	40048	37560	35300	26063	27413	29431
2301	2316	2545	2568	2677	2750	2625	2623
37439	34971	34325	32020	31600	26820	24282	24044
1925	2060	2481	2938	3284	3463	3073	3798
2156	2447	2647	3311	3279	3616	3713	3741
851	969	999	1052	1130	1199	1289	1471
763	875	1013	1095	1332	1541	1556	1604

?1994-2018 China Academic Journal Electronic Publishing House. All rights reserved. http://www.cnki.net

13-3 洛南县经济

指　　标	单 位	2000年	2005年	2006年	2007年	2008年
年底总人口	万人	45.00	45.28	45.25	45.48	45.57
生产总值	亿元	10.47	19.22	22.18	25.99	33.41
第一产业	亿元	4.00	5.03	5.66	6.86	9.90
第二产业	亿元	3.75	7.32	8.71	9.89	12.09
第三产业	亿元	2.72	6.87	7.81	9.24	11.42
# 工业增加值	亿元	2.22	4.69	5.84	6.45	7.27
人均生产总值	元	2351	4259	4900	5729	7339
生产总值指数	上年=100	116.4	110.2	111.0	113.5	116.2
全社会固定资产投资	万元	40162	46743	53200	73484	137347
地方财政收入	万元	6103	3926	5282	7542	11827
地方财政支出	万元	12938	24565	29403	44916	80427
农村居民人均纯收入	元	1219	1482	1605	1844	2499
城镇居民人均可支配收入	元	4669	7222	7631	9005	10894
常用耕地面积	公顷	32064	31330	31242	31263	31286
粮食产量	吨	178527	170121	172720	117141	126733
农林牧渔业总产值	万元	65061	93411	102576	124751	175263
社会消费品零售总额	万元	40570	67688	76582	89831	102636
普通小学专任教师数	人	2649	2350	2269	2315	2181
普通小学在校学生数	人	71400	45000	40800	41300	33500
普通中学专任教师数	人	1480	1805	1870	1878	4323
普通中学在校学生数	人	23500	36100	36200	36500	33900
卫生机构床位数	张	690	714	728	1112	949
卫生技术人员	人	982	897	912	1342	1232
# 执业(助理)医师	人	482	459	472	685	582
注册护师、护士	人	173	181	184	230	249

?1994-2018 China Academic Journal Electronic Publishing House. All rights reserved.　http://www.cnki.net

社会主要指标

2009年	2010年	2011年	2012年	2013年	2014年	2015年	2016年
44.69	44.18	44.06	44.16	44.24	44.32	44.45	44.78
38.15	47.61	58.59	68.07	82.84	92.06	98.17	112.64
10.35	12.57	15.25	17.22	19.12	19.80	20.61	21.70
14.62	19.50	24.87	30.05	40.72	45.99	48.91	59.59
13.18	15.54	18.47	20.81	23.00	26.27	28.65	31.36
8.60	12.40	16.05	19.92	28.77	31.09	33.17	42.58
8366	10716	13279	15432	18741	20757	22118	25248
114.0	114.7	114.8	115.9	113.0	112.3	111.5	109.0
258600	386300	418900	602022	771800	978000	1052700	1245900
15165	19342	27219	35388	43827	50240	54859	42996
89504	109833	157805	206015	220265	243928	266941	268070
3041	3710	4719	5592	6414	7301	7705	8375
13089	15065	17707	20407	22754	25302	23476	25421
31286	31202	31296	31457	31671	31794	31892	31974
160038	178364	150530	163415	168079	142081	162334	164437
188057	221357	271748	310000	344618	364527	368882	389686
120700	143106	166401	193224	219222	246696	278777	315229
2157	2080	1861	1688	1595	1550	1470	1578
29600	28726	28355	27822	26379	21642	21803	22972
2050	2098	2469	2295	2299	2269	2265	2082
31200	30229	27783	25506	24810	20070	19608	19340
980	877	1607	1657	1820	1806	1919	1918
1104	1334	1457	1551	1761	1849	1852	2324
569	404	411	498	552	642	568	556
265	231	428	427	541	484	490	609

?1994-2018 China Academic Journal Electronic Publishing House. All rights reserved. http://www.cnki.net

13-4 丹凤县经济

指　标	单 位	2000年	2005年	2006年	2007年	2008年
年底总人口	万人	29.27	30.13	30.11	30.16	30.23
生产总值	亿元	5.83	13.58	15.20	18.03	24.33
第一产业	亿元	2.56	3.51	3.71	4.36	6.14
第二产业	亿元	1.28	3.75	4.26	5.42	8.34
第三产业	亿元	1.99	6.32	7.23	8.25	9.85
# 工业增加值	亿元	0.68	1.48	1.65	2.28	3.88
人均生产总值	元	1992	4454	5046	5890	8059
生产总值指数	上年=100	113.5	112.6	111.2	113.4	115.9
全社会固定资产投资	万元	15456	45197	40186	63900	121500
地方财政收入	万元	3003	2391	3350	4724	6545
地方财政支出	万元	8119	17317	24519	34131	49345
农村居民人均纯收入	元	998	1540	1661	1906	2617
城镇居民人均可支配收入	元	4041	6279	6844	8872	10678
常用耕地面积	公顷	14680	12404	12442	12312	12319
粮食产量	吨	81289	84581	84791	51605	57008
农林牧渔业总产值	万元	36939	57983	62757	75029	106659
社会消费品零售总额	万元	25521	46824	53023	62118	77500
普通小学专任教师数	人	1526	1548	1445	1899	1651
普通小学在校学生数	人	45200	37400	35000	32400	28300
普通中学专任教师数	人	810	1332	1307	1562	1326
普通中学在校学生数	人	14200	27200	24600	24700	23200
卫生机构床位数	张	450	483	503	613	668
卫生技术人员	人	439	462	576	612	630
# 执业(助理)医师	人	177	256	267	274	289
注册护师、护士	人	56	91	100	110	135

?1994-2018 China Academic Journal Electronic Publishing House. All rights reserved. http://www.cnki.net

社会主要指标

2009年	2010年	2011年	2012年	2013年	2014年	2015年	2016年
29.72	29.55	29.47	29.53	29.58	29.64	29.71	29.85
27.24	33.33	43.18	51.46	61.63	73.06	78.62	86.67
6.43	7.40	9.09	10.34	10.61	11.06	10.92	11.04
9.55	12.55	17.67	21.95	28.25	34.89	38.80	44.00
11.26	13.38	16.42	19.17	22.77	27.11	28.90	31.63
4.31	5.70	8.99	13.35	17.02	21.72	22.94	26.40
9062	11245	16434	17909	21056	24723	26602	29102
114.1	114.9	116.0	114.8	114.0	112.1	111.7	108.8
227400	246600	329300	489300	625300	775700	1024000	1233400
7553	11457	16101	22376	26400	29822	32609	22000
62623	101099	119188	155601	173955	195950	208279	205532
3051	3661	4664	5536	6333	7149	7701	8340
12863	15204	17788	20527	22905	25447	23571	25569
12307	12307	12307	12307	12185	12163	12163	12090
67889	72820	61370	65152	65304	51142	60140	61124
114338	130436	165999	186654	199680	207058	209831	218054
92655	107577	125382	146310	166546	186966	211292	239637
1347	1281	1248	1027	1038	1156	1230	1071
25700	22970	20589	18864	17411	16727	16967	18042
1346	1499	1470	1435	1353	1315	1404	1465
22400	26512	26174	25976	24656	21355	14255	13444
722	804	871	881	1200	1200	1300	1480
668	1136	1180	1199	1394	1403	1417	1447.0
324	531	541	618	496	506	517	483
142	219	233	235	346	360	403	506

?1994-2018 China Academic Journal Electronic Publishing House. All rights reserved.   http://www.cnki.net

13-5 商南县经济

指　　标	单 位	2000年	2005年	2006年	2007年	2008年
年底总人口	万人	22.85	21.93	21.91	21.97	22.04
生产总值	亿元	4.52	11.02	13.54	16.35	20.03
第一产业	亿元	1.73	2.99	3.39	4.18	5.79
第二产业	亿元	1.16	2.57	3.86	4.73	5.29
第三产业	亿元	1.63	5.46	6.29	7.44	8.95
# 工业增加值	亿元	0.59	1.32	2.17	2.55	2.96
人均生产总值	元	1973	4697	5762	6899	8416
生产总值指数	上年=100	118.1	111.5	110.6	113.4	115.9
全社会固定资产投资	万元	12960	42826	60504	92788	116552
地方财政收入	万元	3001	2115	2732	3990	6369
地方财政支出	万元	7761	16347	21748	31072	44679
农村居民人均纯收入	元	1115	1485	1580	1822	2408
城镇居民人均可支配收入	元		6872	7602	8780	10587
常用耕地面积	公顷	13329	13057	12791	12742	13442
粮食产量	吨	70590	71683	76655	40203	54711
农林牧渔业总产值	万元	28671	51893	56659	70590	96715
社会消费品零售总额	万元	12591	32882	37221	43857	54809
普通小学专任教师数	人	1187	1034	968	921	892
普通小学在校学生数	人	36900	20800	19400	18000	17100
普通中学专任教师数	人	934	1188	1211	1063	1070
普通中学在校学生数	人	14700	21800	21900	20400	18800
卫生机构床位数	张	363	422	450	450	504
卫生技术人员	人	546	417	456	463	693
# 执业(助理)医师	人	230	130	134	193	199
注册护师、护士	人	86	44	46	102	173

?1994-2018 China Academic Journal Electronic Publishing House. All rights reserved.　http://www.cnki.net

社会主要指标

2009年	2010年	2011年	2012年	2013年	2014年	2015年	2016年
21.65	22.17	22.10	22.18	22.21	22.28	22.34	22.46
23.07	29.61	38.82	47.39	53.92	63.39	68.92	76.32
6.05	8.17	9.78	11.12	11.97	11.86	11.72	12.32
6.73	9.72	15.28	20.30	24.66	31.34	35.00	39.68
10.29	11.72	13.76	15.97	17.29	20.19	22.20	24.32
3.83	6.09	10.68	14.93	18.44	23.69	26.50	30.55
9669	12379	16126	21367	24296	28498	30893	34067
114.3	114.7	114.9	114.8	112.8	111.5	111.6	109.3
222300	337600	309900	455400	579300	720620	938100	1220700
8988	14058	19647	26371	29113	31983	34497	28526
62036	85410	120861	148342	159817	180405	204575	203095
2998	3681	4678	5529	6400	7219	7739	8420
12743	14653	17327	20058	22307	24783	23250	25215
14048	14084	14224	14201	14153	14138	14126	14038
63153	69399	57476	61733	62560	49332	56538	57209
103581	148408	182457	201808	217129	227986	226022	238841
65723	70815	82533	96067	109329	122459	138389	157004
870	922	967	776	878	899	902	819
16500	16403	16006	15681	15597	13267	14223	15166
1182	1248	1104	1052	1005	1115	958	933
17400	16534	15152	14879	14688	11004	10867	11204
504	576	599	743	874	914	1258	1202
693	706	897	877	839	904	1189	1206
199	231	294	366	335	301	349	352
173	150	238	204	205	210	224	218

?1994-2018 China Academic Journal Electronic Publishing House. All rights reserved. http://www.cnki.net

13-6 山阳县经济

指　　标	单 位	2000年	2005年	2006年	2007年	2008年
年底总人口	万人	41.87	42.93	42.90	42.99	43.08
生产总值	亿元	7.49	16.03	18.64	22.37	27.64
第一产业	亿元	2.85	3.65	4.05	4.83	6.11
第二产业	亿元	2.04	4.94	5.81	7.13	9.37
第三产业	亿元	2.60	7.44	8.78	10.41	12.16
# 工业增加值	亿元	0.79	2.36	3.05	3.78	5.14
人均生产总值	元	1787	3718	4343	5208	6423
生产总值指数	上年=100	111.3	110.1	110.7	113.1	116.0
全社会固定资产投资	万元	24359	40347	45578	69941	110400
地方财政收入	万元	3401	2482	3450	4728	6320
地方财政支出	万元	10642	21687	28134	42408	61114
农村居民人均纯收入	元	1110	1503	1620	1858	2391
城镇居民人均可支配收入	元	4186	6613	7639	8777	10568
常用耕地面积	公顷	26104	23525	23519	23519	23605
粮食产量	吨	124916	98917	117035	73619	101087
农林牧渔业总产值	万元	50628	60486	67813	80562	101214
社会消费品零售总额	万元	28220	55657	61747	72577	89592
普通小学专任教师数	人	2170	1832	1789	1824	1798
普通小学在校学生数	人	72100	49500	44400	41200	39300
普通中学专任教师数	人	1008	1659	1687	1869	1917
普通中学在校学生数	人	20100	37100	37100	36500	35100
卫生机构床位数	张	425	522	613	677	840
卫生技术人员	人	656	793	798	928	896
# 执业(助理)医师	人	257	277	262	369	316
注册护师、护士	人	85	110	110	105	208

?1994-2018 China Academic Journal Electronic Publishing House. All rights reserved.　http://www.cnki.net

社会主要指标

2009年	2010年	2011年	2012年	2013年	2014年	2015年	2016年
42.19	42.25	42.13	42.22	42.28	42.35	42.47	42.66
31.80	40.38	52.49	66.07	80.39	95.47	103.05	121.30
6.39	8.43	10.76	12.89	15.08	16.52	17.46	19.24
11.29	15.48	22.29	30.13	40.09	49.17	53.24	66.19
14.12	16.47	19.44	23.05	25.22	29.78	32.35	35.87
6.03	8.83	13.93	20.35	28.25	35.61	39.74	50.34
7382	9566	12442	15664	19120	22571	24535	28498
114.4	114.8	115.8	116.8	114.8	112.5	113.3	111.0
223000	261500	350742	501230	635600	821300	1060500	1307200
9760	13200	17807	25116	31530	38003	42113	40366
86179	139200	151231	197743	221761	252670	282617	317339
2956	3693	4723	5606	6474	7380	7850	8501
12696	14624	17098	19750	21922	24443	23245	25135
23688	23943	24005	24006	24001	24003	23992	23975
117808	121300	101795	110726	111477	86812	101365	102559
108400	138200	176457	210779	250496	282881	300646	330490
105423	124900	145438	169619	192135	216392	244453	277726
1738	1879	1727	1882	1836	1816	1714	1606
38100	36178	34795	32539	30978	26139	25816	27649
1947	1908	2145	2014	2010	1856	1901	1910
33300	31227	28869	26440	24607	21048	19703	20001
892	1002	1169	1477	1487	1625	1771	1865
873	953	1362	1592	1675	1713	1786	1758
280	294	339	444	452	460	479	541
211	283	335	384	435	469	482	517

?1994-2018 China Academic Journal Electronic Publishing House. All rights reserved. http://www.cnki.net

13-7 镇安县经济

指　　标	单 位	2000年	2005年	2006年	2007年	2008年
年底总人口	万人	28.67	27.57	27.54	27.62	27.69
生产总值	亿元	8.47	16.01	18.12	21.79	25.61
第一产业	亿元	3.11	4.54	4.74	5.76	6.12
第二产业	亿元	2.35	5.17	6.26	7.71	10.22
第三产业	亿元	3.01	6.30	7.12	8.32	9.27
# 工业增加值	亿元	0.86	1.23	1.61	2.07	2.80
人均生产总值	元	2961	5794	6575	7899	9260
生产总值指数	上年=100	112.6	109.9	111.0	112.4	114.6
全社会固定资产投资	万元	43384	70301	79300	98400	132200
地方财政收入	万元	3710	2526	3208	4918	6452
地方财政支出	万元	8802	17350	23312	38639	56596
农村居民人均纯收入	元	1369	1576	1654	1852	2384
城镇居民人均可支配收入	元	4740	6500	7000	8761	10543
常用耕地面积	公顷	14438	17933	17493	19684	20232
粮食产量	吨	117126	114144	113777	90203	96809
农林牧渔业总产值	万元	52100	72200	79101	91887	106031
社会消费品零售总额	万元	25065	45812	51120	57747	70324
普通小学专任教师数	人	1346	1011	1027	1164	1229
普通小学在校学生数	人	37652	25330	23277	21292	19757
普通中学专任教师数	人	773	1174	1201	1242	1281
普通中学在校学生数	人	12698	22774	22153	21535	21255
卫生机构床位数	张	437	528	540	608	693
卫生技术人员	人	623	691	704	734	847
# 执业(助理)医师	人	268	378	405	425	440
注册护师、护士	人	75	170	185	215	249

?1994-2018 China Academic Journal Electronic Publishing House. All rights reserved. http://www.cnki.net

社会主要指标

2009年	2010年	2011年	2012年	2013年	2014年	2015年	2016年
27.26	27.60	27.52	27.59	27.64	27.70	27.78	27.90
29.62	37.37	47.03	55.85	65.44	77.30	83.41	89.79
6.41	7.88	9.67	10.57	11.71	11.89	11.55	11.78
12.46	16.94	22.59	28.11	35.68	44.57	48.88	52.47
10.75	12.55	14.77	17.17	18.05	20.84	22.98	25.54
3.22	5.39	8.06	12.89	16.25	21.98	24.81	25.80
10671	13433	17066	20820	23970	28008	30249	32251
114.0	115.4	114.8	114.5	112.5	111.5	110.8	110.0
248800	244200	326100	481900	616700	756000	970200	1248300
8250	10388	14189	18936	24150	27689	30238	24505
73878	101415	131414	165662	179351	200995	214935	234665
3003	3718	4721	5585	6394	7276	7750	8370
12668	14831	17452	20201	22475	25037	23292	25289
20278	20723	21324	21569	21559	21586	21961	21899
100000	104223	87688	92502	92941	75641	86613	86690
113665	137811	172292	187819	208394	216494	209866	214784
83052	98534	114747	133653	151574	170091	192193	217218
1229	1213	1232	1173	1014	1020	968	1079
18610	17775	17821	17736	17990	18128	18067	18869
1317	1308	1195	1438	1545	1552	1534	1434
20140	18414	16865	16500	15913	14997	13794	13808
693	693	923	1000	1075	1075	1206	1075
856	1016	1072	1141	1184	1220	1315	1553
470	486	511	495	578	574	574	591
266	295	342	397	474	409	509	538

?1994-2018 China Academic Journal Electronic Publishing House. All rights reserved. http://www.cnki.net

13-8 柞水县经济

指　　标	单 位	2000年	2005年	2006年	2007年	2008年
年底总人口	万人	16.15	15.51	15.50	15.54	15.57
生产总值	亿元	4.13	10.73	14.34	16.00	20.05
第一产业	亿元	1.11	1.78	1.98	2.39	3.18
第二产业	亿元	1.10	3.25	4.19	5.59	8.30
第三产业	亿元	1.92	5.70	8.17	8.02	8.57
# 工业增加值	亿元	0.82	2.13	2.82	3.47	5.90
人均生产总值	元	2565	6916	9246	10307	12890
生产总值指数	上年=100	119.2	110.8	112.4	113.9	116.9
全社会固定资产投资	万元	13400	31408	47555	66100	123500
地方财政收入	万元	2010	2218	3069	4068	5829
地方财政支出	万元	6238	13188	17938	28156	39902
农村居民人均纯收入	元	919	1373	1506	1734	2380
城镇居民人均可支配收入	元	4180	6860	7613	8755	10555
常用耕地面积	公顷	8110	7956	7895	7746	7901
粮食产量	吨	50050	43470	43612	45007	41627
农林牧渔业总产值	万元	16876	29465	33126	40015	52026
社会消费品零售总额	万元	12080	23770	26100	30300	37000
普通小学专任教师数	人	862	872	708	887	913
普通小学在校学生数	人	23000	16000	12000	16000	15600
普通中学专任教师数	人	375	501	533	527	529
普通中学在校学生数	人	5000	9000	9000	9000	8000
卫生机构床位数	张	255	265	274	460	394
卫生技术人员	人	376	367	375	385	412
# 执业(助理)医师	人	232	199	202	267	218
注册护师、护士	人	73	75	76	116	85

?1994-2018 China Academic Journal Electronic Publishing House. All rights reserved.　http://www.cnki.net

社会主要指标

2009年	2010年	2011年	2012年	2013年	2014年	2015年	2016年
15.18	15.35	15.30	15.34	15.37	15.43	15.47	15.66
22.63	29.97	40.48	51.10	60.34	63.73	66.17	74.79
3.33	4.22	4.91	5.28	6.20	6.05	6.30	6.63
9.53	14.56	22.72	31.59	39.18	40.39	41.53	48.29
9.77	11.19	12.85	14.23	14.96	17.28	18.34	19.87
7.47	9.01	18.24	26.43	34.11	34.67	35.21	41.41
14609	19351	26412	33351	39693	41675	42831	48052
114.6	115.5	116.3	116.0	113.0	110.9	111.6	110.5
228800	330100	296020	437700	543400	700400	903500	1152900
7506	10689	14868	21616	26385	27519	30698	24003
51792	66230	89255	120330	130799	134277	146775	175435
2995	3597	4580	5446	6306	7168	7622	8277
12708	14898	17459	20128	22362	24822	23236	25141
8059	8301	8467	8481	8481	8481	8481	8667
45849	46551	39615	41839	42253	35792	40946	41450
55772	70782	87920	93320	109728	116521	126000	131341
43500	51654	60039	69784	78957	88503	100012	112920
893	883	866	879	794	847	803	765
15000	14389	13907	13374	12641	9406	9765	10177
560	760	806	800	819	785	787	763
8000	11076	11295	11581	12108	7343	6862	6635
422	567	471	479	501	545	545	617
425	441	491	504	537	608	747	749
224	194	181	191	179	187	175	385
100	156	148	172	191	221	225	237

?1994-2018 China Academic Journal Electronic Publishing House. All rights reserved. http://www.cnki.net
