# Supplementary material for: A study on the tourism efficiency of tourism destination based on DEA model: A case of ten cities in Shaanxi province
Source: PLoS One. 2024 Jan 19;19(1):e0296660. doi: 10.1371/journal.pone.0296660 (PMC10798521; doi:10.1371/journal.pone.0296660)
Supplement: S1 File — (ZIP) [file pone.0296660.s001.zip › Supporting information/Statistical yearbook/Tongchuan.caj]

五、铜川市

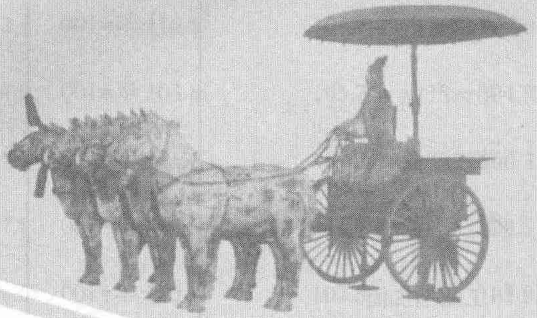

资料整理：王　珍　李雯佳

?1994-2018 China Academic Journal Electronic Publishing House. All rights reserved.　http://www.cnki.net

5-1 铜川市经济

指　标	单 位	2000年	2005年	2006年	2007年	2008年
年底总人口	万人	83.22	82.99	83.19	83.56	83.93
人口自然增长率	‰			4.42	4.44	4.42
年底总户数	万户	21.31	25.30	25.76	26.11	26.70
生产总值	亿元	34.55	71.84	86.41	102.81	129.87
第一产业	亿元	4.02	5.87	6.33	7.84	9.68
第二产业	亿元	15.53	38.67	49.08	58.81	77.94
第三产业	亿元	15.00	27.30	31.00	36.16	42.25
# 工业增加值	亿元	12.96	33.66	43.29	51.99	68.91
人均生产总值	元	4171	8582	10993	12331	15508
生产总值指数	上年=100	108.3	112.7	115.0	115.3	117.1
第一产业	上年=100	108.1	106.9	108.0	104.9	107.8
第二产业	上年=100	108.5	113.2	119.5	118.5	119.9
第三产业	上年=100	108.1	113.2	110.2	112.7	114.3
# 工业增加值	上年=100	108.8	112.5	120.4	119.9	121.1
人均生产总值指数	上年=100	107.7	113.3	115.8	115.0	116.6
非公有制经济增加值	亿元		30.27	33.77	39.40	48.60
文化产业增加值	亿元					
单位GDP能耗	吨标准煤/万元		2.160	2.123	2.017	1.917
单位GDP能耗比上年增长	%			-1.70	-5.01	-4.95
就业人员	万人	41.07	40.33	39.32	40.07	39.80
城镇单位就业人员	万人	12.24	9.56	9.31	9.06	9.32
# 国有单位	万人	9.62	7.84	7.64	7.51	7.80
集体单位	万人	1.34	0.48	0.46	0.35	0.30
# 在岗职工人数	万人	12.13	9.17	8.92	8.79	8.77
城镇单位就业人员平均工资	元					
城镇单位在岗职工平均工资	元	6484	12668	15221	18963	23988

?1994-2018 China Academic Journal Electronic Publishing House. All rights reserved. http://www.cnki.net

社会主要指标

2009年	2010年	2011年	2012年	2013年	2014年	2015年	2016年
83.31	83.50	83.82	84.08	84.28	84.51	84.62	84.72
4.34	3.51	3.36	3.62	3.69	3.79	3.53	3.83
27.51	27.33	27.55	27.93	28.16	27.90	28.03	28.18
154.40	187.73	232.63	273.31	323.27	325.36	307.16	311.61
10.81	14.18	17.41	19.47	20.96	22.61	22.76	23.91
93.73	116.50	147.41	176.82	210.85	204.88	170.31	159.76
49.86	57.05	67.81	77.02	91.46	97.87	114.09	127.94
82.94	103.99	131.91	159.29	190.60	182.48	146.83	134.36
18375	22509	27806	32556	38402	38550	36322	36803
115.2	115.6	116.0	115.8	113.8	110.5	108.5	107.0
106.3	107.7	107.3	106.3	104.9	104.8	105.2	104.3
114.0	118.1	118.0	119.4	117.2	111.0	107.7	106.3
118.9	112.8	114.2	110.5	107.8	110.6	111.1	108.5
113.1	119.0	118.2	120.6	118.0	110.8	107.8	105.9
114.9	115.3	115.7	115.4	113.4	110.2	108.3	106.8
63.40	82.13	107.48	129.00	154.62	158.20	154.25	160.73
					10.33	11.49	13.01
1.798	1.666	1.606	1.548	1.466	1.369	1.278	1.380
-6.21	-4.31	-3.62	-3.62	-5.31	-6.62	-6.60	-3.92
40.34	41.08	42.44	43.24	44.26	45.40	46.69	47.25
9.67	9.83	10.16	10.03	12.00	12.13	12.02	11.50
7.63	7.85	8.13	7.89	5.43	5.47	4.93	4.60
0.29	0.24	0.24	0.20	0.32	0.30	0.27	0.27
9.23	9.43	9.67	9.31	11.05	11.01	10.96	10.34
			36884	43897	45665	47506	50005
28220	31046	33963	38722	45980	47539	49562	52459

?1994-2018 China Academic Journal Electronic Publishing House. All rights reserved. http://www.cnki.net

5-1　续表 1

指　　标	单 位	2000年	2005年	2006年	2007年	2008年
全社会固定资产投资	亿元	14.01	34.49	43.73	57.03	63.40
# 房地产开发	亿元	2.90	3.75	4.58	5.25	5.92
商品房销售面积	万平方米	22.39	45.92	46.43	44.65	30.16
# 住宅	万平方米	18.40	44.22	44.72	40.71	28.54
地方财政收入	亿元	1.49	3.02	4.07	6.00	7.50
地方财政支出	亿元	3.69	7.90	12.20	16.70	25.42
金融机构人民币各项存款余额	亿元	40.51	98.55	115.88	126.38	165.86
金融机构人民币各项贷款余额	亿元	46.31	48.58	53.41	66.82	70.27
农村居民人均纯收入	元	1459	2010	2215	2620	3291
城镇居民人均可支配收入	元	3837	5871	6820	8163	11008
城市人均公园绿地面积	平方米			7.3	7.6	7.6
城市人均道路面积	平方米		8.3	8.3	8.3	9.6
城市用水普及率	%		97.1	97.1	96.9	92.1
城市燃气普及率	%		76.9	76.3	76.2	72.4
常用耕地面积	千公顷	70.06	64.26	64.50	63.86	62.00
农林牧渔业总产值	亿元	7.26	10.62	11.72	14.63	18.14
农作物总播种面积	千公顷	88.57	90.48	90.21	73.86	77.33
# 粮食作物	千公顷	75.06	72.30	71.00	55.35	58.90
粮食产量	万吨	23.05	23.70	24.81	18.24	22.99
棉花产量	吨	23	12	11		
油料产量	吨	7587	9162	8083	7901	10471
蔬菜产量	吨	107200	80053	84819	46769	98201

?1994-2018 China Academic Journal Electronic Publishing House. All rights reserved.　http://www.cnki.net

2009年	2010年	2011年	2012年	2013年	2014年	2015年	2016年
88.12	117.23	145.96	201.81	260.02	327.63	383.98	423.23
10.94	14.68	20.36	22.52	22.49	31.66	28.30	42.74
36.36	44.02	51.76	26.68	28.28	32.06	30.47	34.77
35.89	43.97	51.71	25.94	26.15	31.43	29.45	32.32
10.08	13.75	18.89	21.00	24.10	22.06	23.11	21.51
33.71	53.96	64.34	71.94	81.15	83.01	89.86	96.87
206.62	250.92	296.45	350.87	392.82	411.85	451.78	463.99
80.83	79.40	91.20	99.73	114.54	124.18	145.85	173.49
3969	4789	6077	7134	8140	7966	8739	9478
13717	15884	18775	21929	24495	23550	25559	27594
9.5	9.7	10.0	10.6	11.4	11.5	11.7	11.8
10.2	11.0	11.2	11.1	11.4	11.5	11.5	10.0
95.2	95.7	93.8	94.6	94.8	92.9	93.4	92.9
73.7	73.4	71.5	74.0	75.0	91.2	80.0	90.5
62.60	62.71	63.32	64.57	64.67	64.64	64.70	67.16
19.15	25.15	30.90	34.59	38.74	41.88	42.22	44.32
79.53	82.95	79.33	78.83	79.34	78.73	80.54	80.19
60.79	63.09	58.72	59.28	59.76	60.06	61.67	62.67
23.32	26.63	21.44	24.41	23.98	23.39	24.11	24.18
9706	9706	9590	10046	8174	8049	9027	8478
105826	117911	133601	143951	155000	164500	173250	182817

?1994-2018 China Academic Journal Electronic Publishing House. All rights reserved.   http://www.cnki.net

5-1 续表 2

指 标	单 位	2000年	2005年	2006年	2007年	2008年
水果产量	吨	148539	264762	305714	332987	373893
# 苹果	吨	145131	255253	298540	322834	355693
肉类产量	吨	9011	13051	14099	7749	8770
# 猪牛羊肉	吨	8330	12172	13145	6758	7803
奶类产量	吨	351	13186	19463	23897	17793
# 牛奶	吨	224	11622	17786	22080	14943
禽蛋产量	吨	4470	5723	6387	6159	6728
水产品产量	吨	604	881	873	204	242
规模以上工业企业单位数	个	82	117	136	121	124
规模以上工业总产值	亿元	30.84	70.49	99.53	124.34	166.84
纱产量	吨	5161	6005	7029	7002	8514
布产量	万米	704	425	318	55	
原煤产量	万吨	973.02	1336.33	1676.68	1749.43	1745.97
发电量	万千瓦小时	571	37556	39388		568631
水泥产量	万吨	346.93	495.95	565.89	659.39	724.68
建筑业企业单位数	个	34	24	23	23	25
建筑业企业年末从业人员	万人	1.70	0.91	1.00	0.96	1.06
建筑业总产值	亿元	4.29	6.35	8.92	11.42	19.63
房屋建筑施工面积	万平方米	90.85	96.85	110.98	137.51	147.75
房屋建筑竣工面积	万平方米	27.41	36.69	35.56	41.84	61.28
公路里程	公里	1260	2051	3271	3243	3185
# 等级公路	公里	1253	2033	2357	2814	2765
# 高速公路	公里		96	96	96	96
民用汽车拥有量	辆	11379	20628	23748	27562	33850
# 私人汽车	辆	5396	10666	15804	21557	23797
邮电业务总量	亿元	1.52	6.42	7.98	9.63	10.83
邮政业务总量	亿元	0.12	0.45	0.51	0.56	0.61
电信业务总量	亿元	1.40	5.97	7.47	9.07	10.23

?1994-2018 China Academic Journal Electronic Publishing House. All rights reserved. http://www.cnki.net

2009年	2010年	2011年	2012年	2013年	2014年	2015年	2016年
471274	557781	629490	670865	673462	690593	730204	756706
447769	532015	605229	647008	647689	663312	691986	715419
12273	14641	15453	16125	16879	17278	16887	16915
11054	12443	13207	13991	14492	14904	14532	14601
22466	26001	27650	28077	29907	30292	29181	26862
20221	23633	25522	26000	27807	28400	26141	25473
11118	13796	15796	16118	16991	17039	16504	16330
475	1135	1210	1207	1223	1327	1383	1348
136	130	121	144	146	153	172	203
195.02	249.05	335.76	461.80	549.66	565.91	565.23	559.83
10495	10987	12259	16465	23600	31806	38534	11266
1914.64	2140.75	2363.06	3029.74	2921.02	2775.50	2594.85	1916.21
522701	550506	631780	650089	678900	601475	598950	601706
748.05	997.39	1429.23	1948.66	2613.64	2998.51	3070.78	2380.79
27	28	28	29	28	31	32	36
0.93	1.22	1.40	0.67	0.98	0.66	1.12	1.19
24.32	26.10	34.12	29.74	31.44	31.96	28.58	31.39
177.97	223.16	304.47	326.58	375.26	391.22	357.39	422.65
75.31	38.84	54.22	63.36	49.34	94.21	69.28	113.88
3427	3521	3560	3707	3782	3767	3901	3980
3037	3140	3179	3248	3296	3307	3443	3531
97	99	104	104	152	152	236	236
40834	49915	37487	56114	61596	69349	76573	80904
29582	36492	42384	41521	47033	55854	64226	70047
10.93	4.69	6.64	7.23	7.76	9.95	13.74	52.77
0.64	0.58	0.63	0.76	0.64	0.66	0.76	0.96
10.29	4.10	6.01	6.47	7.12	9.29	12.98	51.81

?1994-2018 China Academic Journal Electronic Publishing House. All rights reserved. http://www.cnki.net

5-1 续表 3

指　标	单 位	2000年	2005年	2006年	2007年	2008年
固定电话用户	万户	8.28	17.99	20.35	21.49	28.10
移动电话用户	万户	2.61	19.03	22.28	28.89	33.84
互联网宽带用户	万户	0.35	1.85	2.46	3.40	4.06
限额以上企业数	个					
批发业	个					
零售业	个					
住宿业	个					
餐饮业	个					
社会消费品零售总额	亿元	13.79	21.76	24.12	27.58	33.54
进出口总额	万美元		3443	1700	2696	2364
# 出口	万美元		2129	224	565	677
实际外商直接投资额	万美元			365	750	536
入境旅游人数	万人次	0.46	0.91	1.01	1.40	1.60
# 外国人	万人次	0.34	0.71	0.73	0.79	1.01
国际旅游外汇收入	万美元	69	136	151	210	240
国内旅游人数	万人次	164	291	320	346	367
国内旅游收入	亿元	1.32	3.15	3.46	4.20	5.20
星级饭店数	个		11	11	12	12
幼儿园数	所	31	30	25	33	28
在园儿童数	万人	0.72	0.57	0.57	0.58	0.60
普通小学学校数	所	935	608	528	461	375
普通小学专任教师数	人	5177	4885	4895	4801	4638
普通小学在校学生数	万人	10.71	6.82	6.43	5.88	5.49
普通中学学校数	所	76	81	77	75	73
普通中学专任教师数	人	3039	3741	3875	4015	4056
普通中学在校学生数	万人	5.29	6.81	6.99	6.75	6.51
卫生机构数	个	163	152	152	135	126
卫生机构床位数	张	3412	3698	4035	4042	4109
卫生技术人员	人	4563	3927	4070	3765	3776
# 执业(助理)医师	人	2003	1656	1760	1591	1463
注册护师、护士	人	1499	1266	1354	1372	1370

?1994-2018 China Academic Journal Electronic Publishing House. All rights reserved.　http://www.cnki.net

2009年	2010年	2011年	2012年	2013年	2014年	2015年	2016年
24.88	15.21	14.49	13.62	13.75	13.43	13.22	12.73
44.75	48.06	52.95	56.01	62.77	66.71	65.96	67.38
4.41	5.44	6.69	7.28	8.57	9.42	10.34	15.27
35	64	83	107	129	143	160	185
4	5	7	8	10	10	16	17
14	31	46	61	75	87	99	118
12	14	14	18	24	28	24	26
5	14	16	20	20	18	21	24
40.56	49.15	59.15	71.12	84.70	96.64	110.05	134.97
721	490	1017	1242	1623	1.37(亿元)	1.48(亿元)	2.14(亿元)
360	391	836	985	1518	1.36(亿元)	1.47(亿元)	2.14(亿元)
500	520	2098	3000	3003	2100		
1.92	2.24	2.45	2.60	2.60	2.60	2.60	2.67
1.17	1.29	1.52	1.63	1.57	1.60	1.58	1.64
288	336	268	390	391	424	413	419
430	495	560	677	886	993	1211	1392
6.50	10.50	19.11	30.60	43.75	53.60	65.34	76.29
12	12	13	13	13	13	13	9
29	51	53	67	83	98	94	101
0.64	0.94	1.31	1.53	1.61	1.70	1.81	1.84
290	220	194	139	130	100	85	87
4619	4536	3896	3775	3459	3284	3177	2804
5.34	5.12	4.80	4.05	3.63	3.59	3.57	3.63
70	58	58	55	53	49	49	44
4056	3925	4539	3914	4262	4040	3416	3396
6.21	5.84	5.39	4.65	4.22	4.00	3.85	3.63
138	146	972	1001	942	947	946	950
4536	4658	4629	4621	4855	5179	5219	5939
4389	5161	6074	6401	6764	7048	7310	8159
1508	1747	2156	2155	2179	2156	2158	2321
1625	1971	2317	2515	2764	2895	3001	3428

?1994-2018 China Academic Journal Electronic Publishing House. All rights reserved. http://www.cnki.net

5-2 王益区经济

指　　标	单　位	2000年	2005年	2006年	2007年	2008年
年底总人口	万人	21.43	21.02	21.08	21.17	21.37
生产总值	亿元	3.03	20.92	23.51	26.35	30.77
第一产业	亿元	0.24	0.32	0.37	0.44	0.53
第二产业	亿元	1.70	8.71	9.96	10.52	13.12
第三产业	亿元	1.10	11.89	13.19	15.39	17.12
# 工业增加值	亿元	1.45	6.96	7.95	8.39	10.17
人均生产总值	元	3986	9784	11121	11932	14466
生产总值指数	上年=100	109.2	114.1	114.3	115.8	116.4
全社会固定资产投资	万元	608	21750	29100	39240	174259
地方财政收入	万元	1409	2903	3742	5008	6518
地方财政支出	万元	2574	7596	10614	19001	30960
农村居民人均纯收入	元	1720	2246	2472	2980	3734
城镇居民人均可支配收入	元	3576	5685	6741	8141	11003
常用耕地面积	公顷	4514	3788	3723	3727	3737
粮食产量	吨	12050	12115	11852	8273	9475
农林牧渔业总产值	万元	3888	5924	6615	7934	9540
社会消费品零售总额	万元	38537	98000	109300	124800	151000
普通小学专任教师数	人	992	673	965	934	908
普通小学在校学生数	人	18200	14000	12600	11600	10900
普通中学专任教师数	人	532	542	588	571	342
普通中学在校学生数	人	11500	9800	9600	9300	8500
卫生机构床位数	张	320	320	320	320	320
卫生技术人员	人	239	239	239	239	239
# 执业(助理)医师	人	137	137	137	137	137
注册护师、护士	人	102	102	102	102	102

?1994-2018 China Academic Journal Electronic Publishing House. All rights reserved.   http://www.cnki.net

社会主要指标

2009年	2010年	2011年	2012年	2013年	2014年	2015年	2016年
20.00	20.04	20.10	20.15	20.21	20.26	20.24	20.15
36.70	44.21	53.69	64.12	75.89	80.20	82.16	87.74
0.57	0.75	0.91	1.02	1.09	1.18	1.20	1.25
15.94	19.90	25.01	31.79	41.18	39.52	34.40	35.33
20.19	23.56	27.77	31.30	33.62	39.50	46.56	51.15
15.86	15.60	19.85	25.33	30.86	31.92	26.38	26.76
17179	20868	26749	31858	37607	39634	40573	43446
116.8	115.7	116.4	117.3	114.6	113.8	110.0	108.6
213752	280033	354404	489078	626187	788701	1027071	1161958
8018	10585	14333	18998	21986	21568	22528	21002
38078	53152	67297	86992	93785	96420	97423	119617
4540	5453	6878	8068	9189	10398	8499	9201
13738	15809	18845	22218	24995	27992	25013	27053
3977	3887	3895	3891	3938	3977	3988	5333
10282	11175	9516	11256	10182	9967	10559	10235
10112	13149	16060	17870	19950	21575	22263	23179
175554	211849	253333	300934	353287	400996	457369	549369
990	1058	736	599	555	579	506	479
10200	10461	8920	8178	7681	7242	6961	6918
508	698	843	930	965	876	881	848
7800	7980	14200	11609	10415	10275	9989	9109
320	2058	2029	2079	2169	2278	2238	2092
243	2036	2237	2247	2495	2552	2581	2769
139	621	628	703	730	723	736	747
104	1415	1609	1002	1182	1212	1225	1344

?1994-2018 China Academic Journal Electronic Publishing House. All rights reserved. http://www.cnki.net

5-3 印台区经济

指　　标	单　位	2000年	2005年	2006年	2007年	2008年
年底总人口	万人	23.09	23.14	23.16	23.27	23.32
生产总值	亿元	2.96	20.36	24.82	28.31	32.89
第一产业	亿元	0.58	1.15	1.27	1.68	2.08
第二产业	亿元	0.93	12.30	16.21	18.65	21.48
第三产业	亿元	1.45	6.91	7.33	7.98	9.33
# 工业增加值	亿元	0.76	11.27	15.10	17.51	20.12
人均生产总值	元	1281	8614	10054	11949	13842
生产总值指数	上年=100	108.3	112.5	114.4	116.2	116.6
全社会固定资产投资	万元	3511	19236	25162	37231	59333
地方财政收入	万元	1449	2009	2817	3875	5152
地方财政支出	万元	4445	10315	15477	22904	38594
农村居民人均纯收入	元	1396	1802	2010	2480	3120
城镇居民人均可支配收入	元	3837	5748	6679	8042	10637
常用耕地面积	公顷	12066	11350	11202	10508	8738
粮食产量	吨	38947	42113	43006	30353	34811
农林牧渔业总产值	万元	12365	20891	23540	31031	38486
社会消费品零售总额	万元	19385	46200	50900	57800	70900
普通小学专任教师数	人	1329	837	1114	1139	1109
普通小学在校学生数	人	17800	10800	13200	13600	13000
普通中学专任教师数	人	336	375	269	738	1071
普通中学在校学生数	人	5800	6900	9600	10500	10021
卫生机构床位数	张	1080	1226	1207	1165	1162
卫生技术人员	人	393	469	475	805	827
# 执业(助理)医师	人	62	102	108	330	294
注册护师、护士	人	117	130	129	272	299

?1994-2018 China Academic Journal Electronic Publishing House. All rights reserved.　http://www.cnki.net

社会主要指标

2009年	2010年	2011年	2012年	2013年	2014年	2015年	2016年
21.72	21.77	21.84	21.85	21.88	21.94	21.91	21.65
39.21	47.24	58.34	70.02	77.21	74.32	62.72	63.26
2.31	3.06	3.80	4.26	4.60	4.98	5.06	5.33
25.71	31.76	40.02	49.50	57.20	48.83	33.86	31.77
11.19	12.41	14.52	16.26	15.41	20.51	23.80	26.16
24.11	29.96	37.86	47.15	54.76	45.03	29.95	27.57
16825	20282	26756	32089	35312	33921	28607	29045
116.6	116.3	116.2	115.2	115.0	109.0	105.9	105.6
105880	138065	169846	232179	297505	368063	428075	517971
6001	7225	10693	14666	17397	15176	16168	17508
44010	60497	77776	96232	96498	101861	113027	142041
3806	4597	5843	6863	7879	8934	8175	8919
13339	15473	18305	21325	23778	26348	23654	25464
8510	8604	8846	8924	9269	9208	9223	9333
35657	48191	34462	38450	37361	36091	35691	36198
40467	53595	66520	74923	84041	90846	92086	96681
82026	98080	115390	135464	158907	180058	204353	244207
1058	1047	835	771	634	600	584	329
12800	12235	11200	7892	7172	7078	6830	6655
1040	956	831	846	779	768	668	376
8861	7592	6813	5785	4672	3836	3117	2910
1196	1084	1084	1058	1020	1020	1020	881
1186	1194	1565	1510	1457	1565	1612	1712
366	379	513	539	523	533	521	551
416	432	461	456	446	455	476	509

?1994-2018 China Academic Journal Electronic Publishing House. All rights reserved. http://www.cnki.net

5-4 耀州区经济

指　　标	单　位	2000年	2005年	2006年	2007年	2008年
年底总人口	万人	29.39	29.63	29.73	29.87	29.97
生产总值	亿元	7.87	26.39	32.49	40.56	57.96
第一产业	亿元	1.68	2.89	3.08	3.69	4.52
第二产业	亿元	3.48	16.01	20.50	26.45	40.46
第三产业	亿元	2.71	7.50	8.91	10.42	12.99
# 工业增加值	亿元	2.98	13.68	17.88	23.87	36.13
人均生产总值	元	3060	8908	9215	13611	18957
生产总值指数	上年=100	109.8	111.5	113.6	118.5	118.9
全社会固定资产投资	万元	12010	91502	81728	120530	233961
地方财政收入	万元	4150	6940	9687	12943	16965
地方财政支出	万元	7696	17768	28045	35560	60122
农村居民人均纯收入	元	1438	2047	2248	2695	3404
城镇居民人均可支配收入	元	3219	6684	7660	9123	11812
常用耕地面积	公顷	36272	34015	34447	34363	34052
粮食产量	吨	118321	111822	114730	83269	111683
农林牧渔业总产值	万元	37802	51785	56859	68504	85690
社会消费品零售总额	万元	33516	60500	66100	76900	93600
普通小学专任教师数	人	1623	1879	1977	1973	1960
普通小学在校学生数	人	34900	27000	28400	27500	25400
普通中学专任教师数	人	874	1252	1316	1662	1689
普通中学在校学生数	人	14800	21900	24100	27500	26100
卫生机构床位数	张	439	562	687	837	947
卫生技术人员	人	700	599	701	701	845
# 执业(助理)医师	人	318	322	340	338	478
注册护师、护士	人	209	210	221	219	327

?1994-2018 China Academic Journal Electronic Publishing House. All rights reserved. http://www.cnki.net

社会主要指标

2009年	2010年	2011年	2012年	2013年	2014年	2015年	2016年
32.49	32.58	32.73	32.86	32.94	33.03	33.18	33.61
69.86	82.72	105.06	127.56	145.71	142.67	131.92	127.50
5.06	6.55	7.98	8.90	9.61	10.32	10.47	11.00
48.80	57.74	75.73	94.07	99.71	100.68	84.75	76.02
16.00	18.43	21.36	24.59	36.39	31.67	36.70	40.48
44.23	52.45	69.31	87.27	93.89	91.79	75.45	65.49
23221	27391	32186	38897	44289	43253	39849	38181
116.1	116.6	116.5	116.2	115.6	113.6	109.5	106.5
485900	618407	817811	1136199	1451073	1832736	1993751	2112138
21327	29859	44948	59676	69148	71726	74621	64011
91162	120347	145866	186149	209960	234651	273227	274821
4101	5358	6799	7975	9099	10227	9386	10181
14840	17847	21399	25358	28528	31894	28150	30436
33982	475221	32732	32624	32335	32154	32040	31815
108851	102214	86461	96919	92626	90347	94029	90989
90607	102325	143828	160530	179850	194054	196590	206613
109736	134506	163757	219588	281070	324645	369852	450814
1976	2012	1802	1880	1806	1643	1637	1584
24400	24120	22572	19953	17557	17642	17977	18626
1715	1670	2213	2141	2128	2004	1934	1919
25500	23627	28188	25661	24266	23336	23072	22182
1032	1084	1207	1175	1347	1566	1646	2196
1161	1423	1916	2165	2221	2364	2414	2952
475	529	751	762	764	766	761	761
394	557	769	913	953	1045	1060	1060

?1994-2018 China Academic Journal Electronic Publishing House. All rights reserved. http://www.cnki.net

5-5 宜君县经济

指　　标	单　位	2000年	2005年	2006年	2007年	2008年
年底总人口	万人	9.30	9.20	9.22	9.25	9.27
生产总值	亿元	1.68	4.18	4.86	6.01	8.21
第一产业	亿元	1.01	1.49	1.64	2.10	2.56
第二产业	亿元	0.39	1.54	1.92	2.38	3.38
第三产业	亿元	0.27	1.14	1.30	1.52	2.28
# 工业增加值	亿元	0.35	3.82	3.21	4.21	2.50
人均生产总值	元	1806	4500	5279	7028	8432
生产总值指数	上年=100	110.2	114.0	114.0	115.0	115.0
全社会固定资产投资	万元	5696	16032	20640	26848	43997
地方财政收入	万元	1040	1543	2188	3020	4018
地方财政支出	万元	4379	11132	16408	21492	38475
农村居民人均纯收入	元	1268	1683	1884	2333	2941
城镇居民人均可支配收入	元	2617	5520	6128	7375	10371
常用耕地面积	公顷	17208	15108	15131	15262	15477
粮食产量	吨	61177	70905	78477	60458	73916
农林牧渔业总产值	万元	18544	27588	30235	38796	47636
社会消费品零售总额	万元	7955	12900	14300	16400	19952
普通小学专任教师数	人	671	723	690	700	661
普通小学在校学生数	人	14700	7500	7100	6400	5900
普通中学专任教师数	人	303	432	441	445	429
普通中学在校学生数	人	4900	6900	6500	6000	5400
卫生机构床位数	张	167	212	274	270	273
卫生技术人员	人	274	215	264	225	268
# 执业(助理)医师	人	140	148	149	157	171
注册护师、护士	人	71	77	102	101	108

?1994-2018 China Academic Journal Electronic Publishing House. All rights reserved.   http://www.cnki.net

社会主要指标

2009年	2010年	2011年	2012年	2013年	2014年	2015年	2016年
9.10	9.12	9.16	9.22	9.25	9.28	9.29	9.31
10.48	13.58	17.42	21.21	24.46	28.17	30.36	33.11
2.86	3.83	4.71	5.28	5.66	6.13	6.02	6.33
4.76	6.41	8.44	11.02	12.76	15.85	17.30	18.95
2.86	3.34	4.27	4.91	6.04	6.19	7.04	7.83
3.76	5.17	6.79	9.14	11.09	13.74	15.05	16.50
11304	14886	19017	23076	26486	30405	32698	35602
115.0	115.5	116.0	116.5	113.5	114.0	110.6	109.5
73163	95303	117530	160664	225435	286800	390889	440252
5133	6830	10056	14418	19354	21149	22309	21002
40343	54346	70288	82938	96939	96980	106145	116769
3541	4290	5466	6477	7423	8417	7888	8547
12950	15140	17866	20922	23516	26246	22949	24751
16134	17105	17846	19134	19132	19302	19451	20681
78382	96960	84010	97446	99645	97536	100782	104358
50299	67088	82587	92575	103544	112333	111304	116691
24423	29933	35997	44950	53719	60708	68961	105358
667	600	620	525	464	462	450	412
5600	5342	4514	4450	3927	3965	3970	4132
423	431	492	417	390	392	390	405
5100	4892	3302	3429	2895	2584	2317	2332
273	273	309	309	319	315	315	315
294	307	320	479	591	567	703	726
171	166	185	151	162	134	134	158
108	118	120	144	183	183	183	223

?1994-2018 China Academic Journal Electronic Publishing House. All rights reserved. http://www.cnki.net
