# Supplementary material for: A study on the tourism efficiency of tourism destination based on DEA model: A case of ten cities in Shaanxi province
Source: PLoS One. 2024 Jan 19;19(1):e0296660. doi: 10.1371/journal.pone.0296660 (PMC10798521; doi:10.1371/journal.pone.0296660)
Supplement: S1 File — (ZIP) [file pone.0296660.s001.zip › Supporting information/Statistical yearbook/Weinan.caj]

八、渭南市

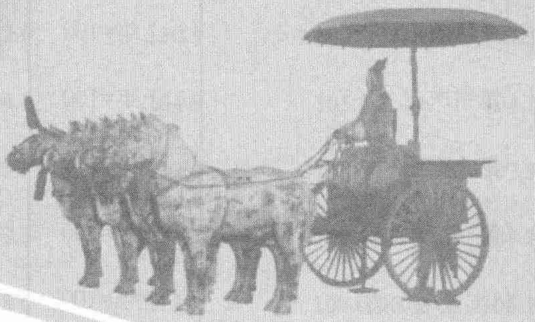

资料整理：同小庆　马　靖

?1994-2018 China Academic Journal Electronic Publishing House. All rights reserved.　http://www.cnki.net

8-1 渭南市经济

指 标	单 位	2000年	2005年	2006年	2007年	2008年
年底总人口	万人	529.02	547.11	545.65	542.04	543.07
人口自然增长率	‰		4.46	4.44	4.43	4.48
年底总户数	万户	136.22	153.46	159.23	161.56	165.36
生产总值	亿元	165.47	330.17	377.40	456.95	563.09
第一产业	亿元	37.43	58.66	63.13	80.19	96.26
第二产业	亿元	60.42	148.71	171.83	206.06	256.22
第三产业	亿元	67.62	122.80	142.44	170.70	210.61
# 工业增加值	亿元	51.82	131.28	151.31	181.10	222.49
人均生产总值	元	3149	6052	6907	8402	10378
生产总值指数	上年=100	108.2	112.4	112.9	114.2	116.3
第一产业	上年=100	104.3	105.5	107.3	104.9	107.6
第二产业	上年=100	107.5	117.7	114.8	115.5	117.3
第三产业	上年=100	112.0	110.6	113.3	116.8	118.7
# 工业增加值	上年=100	107.6	119.1	115.0	115.6	117.1
人均生产总值指数	上年=100	107.2	111.2	112.7	114.7	116.6
非公有制经济增加值	亿元		127.68	150.27	182.89	235.24
文化产业增加值	亿元					
单位GDP能耗	吨标准煤/万元		3.510	3.429	3.263	3.085
单位GDP能耗比上年增长	%			-2.30	-4.85	-5.44
就业人员	万人	272.10	297.70	299.74	301.35	303.16
城镇单位就业人员	万人	35.04	33.75	33.91	34.51	34.73
# 国有单位	万人	29.80	27.41	27.11	27.12	27.68
集体单位	万人	2.30	1.93	1.77	1.62	1.53
# 在岗职工人数	万人	33.90	32.65	32.69	33.39	33.48
城镇单位就业人员平均工资	元					
城镇单位在岗职工平均工资	元	6192	11822	13577	16792	21092

?1994-2018 China Academic Journal Electronic Publishing House. All rights reserved. http://www.cnki.net

社会主要指标

2009年	2010年	2011年	2012年	2013年	2014年	2015年	2016年
529.03	528.99	530.49	532.10	533.17	534.30	535.99	537.16
4.39	3.26	3.29	3.45	3.61	3.46	3.43	3.66
169.48	171.35	176.56	178.35	180.31	176.56	173.52	173.48
636.96	801.42	1028.97	1157.32	1321.81	1423.75	1430.41	1488.62
100.55	128.94	160.47	171.54	193.09	207.16	213.92	224.81
294.44	394.55	545.19	610.67	710.74	751.34	697.70	685.20
241.97	277.93	323.31	375.11	417.98	465.25	518.79	578.61
248.78	339.71	476.64	533.55	623.00	652.18	587.63	560.82
11728	15149	19424	21783	24816	26675	26729	27743
114.3	115.0	115.0	114.5	112.0	110.5	108.5	107.5
106.5	107.3	107.0	106.1	104.6	104.9	105.5	104.1
116.3	120.7	120.6	119.6	115.3	112.1	108.6	107.5
114.7	110.6	110.9	110.3	109.5	109.7	109.5	108.7
113.7	121.8	121.3	121.2	115.6	112.2	108.0	106.3
114.2	115.1	114.9	114.1	111.7	110.2	108.2	107.2
279.58	350.19	459.62	526.82	610.86	676.86	695.19	736.92
					34.89	38.22	42.98
2.946	1.593	1.535	1.480	1.424	1.362	1.318	1.259
-4.51	-4.72	-3.60	-3.61	-3.81	-4.33	-3.21	-3.73
311.97	334.47	351.21	336.87	340.06	365.10	383.20	398.60
34.87	37.35	38.99	39.24	47.31	46.75	45.74	45.04
27.72	27.16	27.45	27.96	24.99	24.93	24.11	23.95
1.52	1.88	1.33	1.11	1.17	1.17	1.18	1.07
34.47	35.47	36.69	36.83	44.02	43.14	42.02	40.32
			38368	40337	42508	45775	49376
24589	28628	33703	39510	41294	43542	46842	51057

?1994-2018 China Academic Journal Electronic Publishing House. All rights reserved. http://www.cnki.net

8-1 续表 1

指 标	单 位	2000年	2005年	2006年	2007年	2008年
全社会固定资产投资	亿元	49.96	107.76	159.69	215.99	333.59
# 房地产开发	亿元	2.10	8.19	10.93	16.11	22.17
商品房销售面积	万平方米	14.82	40.23	73.95	85.53	100.29
# 住宅	万平方米	12.44	36.89	68.04	80.14	94.11
地方财政收入	亿元	8.91	9.01	12.02	16.06	21.36
地方财政支出	亿元	13.76	29.40	40.62	57.88	82.97
金融机构人民币各项存款余额	亿元	188.73	411.23	471.52	519.80	651.75
金融机构人民币各项贷款余额	亿元	169.02	236.60	265.33	291.22	319.85
农村居民人均纯收入	元	1465	1882	2043	2410	2972
城镇居民人均可支配收入	元	4117	6764	7451	8827	11001
城市人均公园绿地面积	平方米			3.9	7.6	7.6
城市人均道路面积	平方米		10.3	11.6	17.4	19.8
城市用水普及率	%	93.3	99.5	99.4	99.2	99.2
城市燃气普及率	%	63.3	93.1	98.2	94.5	94.8
常用耕地面积	千公顷	534.61	514.80	513.09	517.91	519.19
农林牧渔业总产值	亿元	68.91	96.32	107.31	136.00	167.34
农作物总播种面积	千公顷	701.36	700.11	701.41	652.26	704.83
# 粮食作物	千公顷	584.29	541.82	531.60	481.05	532.80
粮食产量	万吨	184.17	190.28	201.19	166.91	228.56
棉花产量	吨	23455	67884	72441	80389	91711
油料产量	吨	88919	68411	71707	55732	67168
蔬菜产量	吨	563022	633346	680677	693757	960089

?1994-2018 China Academic Journal Electronic Publishing House. All rights reserved. http://www.cnki.net

2009年	2010年	2011年	2012年	2013年	2014年	2015年	2016年
509.39	742.25	912.94	1172.21	1467.61	1765.63	2085.21	2289.51
28.20	42.08	68.21	85.11	101.33	81.35	97.33	96.95
100.38	139.72	243.49	257.17	320.96	325.26	220.99	174.48
94.40	127.87	211.93	233.48	301.60	282.85	188.77	159.61
28.25	34.00	44.30	55.06	65.06	67.46	72.06	65.69
116.54	154.30	197.32	242.41	275.87	293.41	336.09	352.51
847.23	998.47	1131.64	1347.73	1522.40	1664.58	1837.37	2061.59
386.38	471.52	544.34	622.52	726.02	811.40	920.59	1035.35
3584	4372	5571	6602	7565	7935	8705	9415
13652	15918	18768	21808	24164	23470	25472	27485
9.8	11.9	12.1	12.2	12.2	12.2	12.3	12.8
17.0	16.8	18.8	18.7	8.5	9.5	10.2	10.3
93.7	99.4	99.5	99.8	99.3	99.3	98.8	98.6
85.9	86.7	81.3	83.4	84.7	88.1	92.5	93.2
520.13	521.04	521.65	521.45	519.41	511.14	505.34	493.48
174.67	227.61	283.71	318.00	357.54	384.46	397.54	418.69
754.28	758.77	721.79	703.67	696.67	690.47	687.25	683.44
572.23	583.84	538.00	521.64	520.13	512.98	510.77	508.14
245.13	264.19	210.47	224.34	211.17	206.01	217.27	211.53
75932	61501	60070	59365	53612	40219	36399	32229
65647	65647	66322	69754	64097	73583	72976	72924
1726268	1918245	2013540	2142860	2250100	2388100	2532330	2701945

?1994-2018 China Academic Journal Electronic Publishing House. All rights reserved. http://www.cnki.net

8-1　续表 2

指　　标	单 位	2000年	2005年	2006年	2007年	2008年
水果产量	吨	1319485	1283589	1578790	1573896	1799235
# 苹果	吨	1107810	961794	1200284	1210942	1348448
肉类产量	吨	86651	122491	129062	78877	105348
# 猪牛羊肉	吨	72786	108438	115479	67440	93551
奶类产量	吨	87825	124251	130950	165509	221754
# 牛奶	吨	14910	56694	62355	92229	137557
禽蛋产量	吨	65429	66358	67933	56948	59407
水产品产量	吨	13660	15300	15022	7277	7508
规模以上工业企业单位数	个	278	265	267	265	283
规模以上工业总产值	亿元	100.27	337.71	377.63	474.27	638.00
纱产量	万吨	1.78	2.31	0.01	2.68	2.67
布产量	万米	10644	9013	283	7087	3544
原煤产量	万吨	704.65	1191.24	1122.86	1561.10	1521.79
发电量	亿千瓦小时	103.59	167.67	218.81	240.61	232.21
粗钢产量	万吨	19.78	204.42	254.50	263.51	204.20
钢材产量	万吨	17.54	152.74	224.02	271.11	244.03
水泥产量	万吨	243.61	275.72	338.80	440.30	482.96
建筑业企业单位数	个	90	93	93	90	93
建筑业企业年末从业人员	万人	3.39	5.11	4.26	4.44	4.73
建筑业总产值	亿元	16.92	62.36	73.03	89.04	98.23
房屋建筑施工面积	万平方米	107.77	229.69	282.73	330.95	381.83
房屋建筑竣工面积	万平方米	48.75	125.23	132.87	177.88	225.87
公路里程	公里	5198	5945	11941	14940	17072
# 等级公路	公里	5056	5773	7671	11861	13204
# 高速公路	公里	96	270	270	270	270
民用汽车拥有量	辆	32318	66357	97672	113515	116766
# 私人汽车	辆	16351	36219	68370	71219	69307
邮电业务总量	亿元	8.25	29.83	38.35	47.70	56.64
邮政业务总量	亿元	0.41	1.41	1.75	2.12	2.46
电信业务总量	亿元	7.84	28.41	36.60	45.58	54.18

?1994-2018 China Academic Journal Electronic Publishing House. All rights reserved.　http://www.cnki.net

2009年	2010年	2011年	2012年	2013年	2014年	2015年	2016年
2376744	2542490	2738925	2820252	2830857	2920632	3105551	3272056
1751151	1826027	1929095	1949679	1929175	1937295	1991745	2049356
158135	178342	196762	206230	215707	222017	220137	219035
145831	164682	180683	189877	197534	204697	201226	201230
296480	335594	363871	378295	392582	402279	403968	398424
180886	221499	230575	232135	246145	251677	250884	247946
77839	85662	97722	100370	105196	105138	108352	110172
8205	17375	17115	20004	26151	29823	44818	47581
422	527	342	411	415	450	490	512
767.22	1039.83	1360.03	1634.36	1728.47	1959.32	2053.87	2141.37
3.74	3.73	3.94	3.34	2.06	2.53	3.01	2.16
3834	1448	1223	190				
1614.64	1884.41	2487.57	3045.32	2494.41	2292.21	2341.05	2340.58
259.20	282.89	275.04	321.69	339.95	334.66	293.96	302.18
373.09	408.07	559.18	525.12	545.70	589.21	622.26	567.31
394.64	459.91	562.58	581.09	558.48	612.74	626.74	683.32
436.49	523.21	575.78	810.36	1014.60	946.50	763.94	736.49
93	90	89	88	104	116	125	130
5.27	7.90	5.65	5.16	12.71	7.12	7.05	7.39
144.05	221.13	314.48	328.60	215.64	239.21	261.28	276.87
579.29	909.23	1104.86	1396.69	1358.83	1388.51	1612.00	1224.51
223.01	323.05	919.81	472.54	532.49	546.06	495.68	484.68
17388	17716	17764	18073	18182	18402	18644	19029
14206	14627	14715	15066	15200	15389	15710	16076
270	324	324	324	324	324	356	367
150868	266699	309902	361235	396764	394396	434758	478700
106794	205237	238791	277933	312502	316876	361220	411327
57.28	24.46	32.16	35.05	38.56	49.95	64.96	103.12
2.99	2.60	2.94	2.56	2.98	3.36	4.24	5.65
54.29	21.86	29.22	32.49	35.58	46.59	60.72	97.47

?1994-2018 China Academic Journal Electronic Publishing House. All rights reserved. http://www.cnki.net

8-1 续表 3

指 标	单 位	2000年	2005年	2006年	2007年	2008年
固定电话用户	万户	38.01	100.05	105.41	105.25	98.53
移动电话用户	万户	15.84	103.54	117.86	166.31	190.77
互联网宽带用户	万户	1.68	9.25	10.30	13.92	19.25
限额以上企业数	个					
批发业	个					
零售业	个					
住宿业	个					
餐饮业	个					
社会消费品零售总额	亿元	58.53	93.02	108.65	131.96	175.03
进出口总额	万美元		9768	11646	14903	11994
# 出口额	万美元		6195	9321	12291	9314
实际外商直接投资额	万美元		1725	2041	3448	5499
入境旅游人数	万人次	1.30	3.00	3.67	4.56	6.41
# 外国人	万人次	0.86	2.14	2.38	3.75	4.13
国际旅游外汇收入	万美元	222.89	526.52	641.70	821.30	1153.80
国内旅游人数	万人次	308.70	600.00	688.83	798.84	898.69
国内旅游收入	亿元	5.43	18.08	20.95	26.20	36.33
星级饭店数	个		16	16	23	24
幼儿园数	所	1305	654	545	532	499
在园儿童数	万人	7.94	6.13	6.09	3.93	6.45
普通小学学校数	所	3886	2600	2392	2146	1854
普通小学专任教师数	人	23937	27199	27001	26163	25391
普通小学在校学生数	万人	71.31	45.77	43.79	41.03	38.23
普通中学学校数	所	388	429	422	405	408
普通中学专任教师数	人	18760	25542	26560	26926	27163
普通中学在校学生数	万人	36.23	48.66	49.67	47.87	45.69
卫生机构数	个	482	433	424	399	351
卫生机构床位数	张	9331	10680	11020	11550	12845
卫生技术人员	人	12449	13730	14048	14589	14177
# 执业(助理)医师	人	5769	6136	6137	5900	5808
注册护师、护士	人	3421	3626	3867	4208	3947

?1994-2018 China Academic Journal Electronic Publishing House. All rights reserved. http://www.cnki.net

2009年	2010年	2011年	2012年	2013年	2014年	2015年	2016年
94.86	90.08	87.50	85.83	84.39	82.46	79.97	74.44
221.43	246.08	282.51	314.95	337.60	362.98	407.79	413.84
21.91	31.47	39.86	50.58	60.75	65.84	72.99	93.01
203	260	366	433	504	510	567	625
18	18	21	31	35	45	50	58
100	136	217	256	303	310	346	386
27	34	41	47	54	54	51	55
58	72	87	99	112	101	120	126
202.80	240.77	284.08	335.00	387.70	441.98	503.25	574.01
11505	16927	17390	23329	24335	14.17(亿元)	10.55(亿元)	12.13(亿元)
8454	10110	13420	14434	20201	11.91(亿元)	8.66(亿元)	10.65(亿元)
4470	3051	5019	6183	5008	1216	289	3
9.05	15.04	18.87	19.62	20.13	28.90	30.02	34.30
5.37	6.02	7.13	4.72	5.12	14.40	19.00	17.80
1628.32	2707.70	3545.00	5604.36	5757.00	7093.00	7410.00	7523.00
1103.48	1313.50	2039.00	2492.46	2900.00	3173.10	4200.00	4780.00
50.32	65.75	116.30	152.03	212.00	247.68	323.80	361.00
27	24	33	33	33	29	34	31
483	575	1002	1037	1086	1102	1194	1188
6.73	8.46	14.75	16.16	17.19	18.13	19.35	19.67
1609	1362	1266	1175	1038	937	787	753
24548	23624	21287	20544	19487	18861	17845	17480
35.89	34.25	32.60	27.65	26.97	27.21	27.56	28.26
397	390	385	368	346	329	328	314
27435	27479	29117	27084	27912	26700	23891	25084
42.36	39.02	36.11	32.05	29.31	26.74	24.79	23.47
345	358	3893	3882	4137	4203	4246	4291
13276	13950	15913	18147	19724	20832	22183	26972
14643	15943	19126	21270	24504	25780	26869	30575
5687	5955	5775	5980	6878	7266	7710	8651
4051	4690	5935	6792	8184	8891	9616	11593

?1994-2018 China Academic Journal Electronic Publishing House. All rights reserved. http://www.cnki.net

8-2 临渭区经济

指 标	单 位	2000年	2005年	2006年	2007年	2008年
年底总人口	万人	88.44	91.93	91.87	91.69	91.85
生产总值	亿元	32.92	68.27	80.78	96.38	121.88
第一产业	亿元	6.17	9.10	11.24	14.09	16.19
第二产业	亿元	12.55	31.40	38.01	43.71	55.51
第三产业	亿元	14.20	27.77	31.53	38.58	50.18
# 工业增加值	亿元	8.27	22.59	27.58	32.45	40.96
人均生产总值	元	3744	7423	8790	10501	13281
生产总值指数	上年=100	109.4	115.7	112.2	119.4	120.1
全社会固定资产投资	万元	111528	313769	385353	530267	909865
地方财政收入	万元	9739	6267	7558	11530	16321
地方财政支出	万元	13532	30299	40015	62413	90949
农村居民人均纯收入	元	1609	2160	2322	2638	3150
城镇居民人均可支配收入	元	4275	7172	7684	9025	11282
常用耕地面积	公顷	76522	67050	67691	69026	68898
粮食产量	吨	346528	361597	365206	291701	387019
农林牧渔业总产值	万元	115753	168098	184307	238723	291390
社会消费品零售总额	万元	105365	198885	258417	338217	455266
普通小学专任教师数	人	4041	3854	3762	3565	3488
普通小学在校学生数	人	113200	75800	71900	65400	62400
普通中学专任教师数	人	3319	3943	4055	4155	4080
普通中学在校学生数	人	62500	80500	79500	77800	71800
卫生机构床位数	张	2135	2158	2179	2426	2763
卫生技术人员	人	1418	1514	1493	2425	2842
# 执业(助理)医师	人	1039	1102	1078	1590	1914
注册护师、护士	人	398	431	434	854	976

?1994-2018 China Academic Journal Electronic Publishing House. All rights reserved. http://www.cnki.net

社会主要指标

2009年	2010	2011年	2012年	2013年	2014年	2015年	2016年
87.78	87.78	88.19	88.78	88.88	89.07	89.45	89.82
141.83	173.53	220.26	262.52	253.16	291.18	306.81	332.67
16.98	21.61	26.50	29.33	31.76	33.90	35.08	36.77
65.25	81.08	108.20	134.91	115.80	138.77	141.42	151.99
59.61	70.84	85.57	98.28	105.58	118.52	130.31	143.91
45.75	57.34	77.82	101.21	75.60	89.55	113.19	86.53
15439	18886	25034	29669	30817	34143	34300	37037
118.1	118.4	117.6	117.4	112.1	111.5	111.7	110.0
1356388	1614135	1919684	2668700	3206501	3708456	4899200	5650000
21164	27666	38611	47578	58439	64761	67403	60259
129574	176929	226591	274551	365762	380995	432053	472469
3736	4565	5820	6887	7870	8940	9045	9805
14008	16495	19679	23280	26143	29149	26897	29092
68415	69277	68702	69495	69522	65963	65683	63547
396689	429995	350510	367542	337679	333084	349473	342034
302580	383463	476007	527055	590453	630349	653461	687069
525235	607186	716307	889531	1037532	1196605	1361381	1560768
3603	3856	3952	3882	2534	2679	2558	2542
28700	30245	35264	40563	42491	42580	42919	44604
4390	4693	4986	4659	3419	4090	3623	3450
70600	72654	75285	72364	50770	44572	41026	37128
2961	4035	3987	4121	3998	2695	3534	5678
3471	4530	6097	5698	9031	5820	6260	5277
2077	2578	3012	2896	3120	3240	3512	2960
1357	1475	1892	1762	1889	1920	2038	2317

?1994-2018 China Academic Journal Electronic Publishing House. All rights reserved.   http://www.cnki.net

8-3 华州区经济

指 标	单 位	2000年	2005年	2006年	2007年	2008年
年底总人口	万人	34.38	36.40	36.18	35.18	35.29
生产总值	亿元	9.60	36.08	37.82	45.16	54.47
第一产业	亿元	1.56	2.27	2.51	3.11	4.29
第二产业	亿元	5.43	28.13	28.58	33.58	39.99
第三产业	亿元	2.61	5.68	6.74	8.47	10.19
# 工业增加值	亿元	4.92	27.75	27.85	32.69	38.80
人均生产总值	元	2800	10515	10422	12657	15460
生产总值指数	上年=100	108.8	120.4	107.2	114.2	116.4
全社会固定资产投资	万元	23463	52893	96453	144769	260615
地方财政收入	万元	5800	16929	26706	30349	40099
地方财政支出	万元	7712	28663	43825	53939	67593
农村居民人均纯收入	元	1350	1425	1645	2371	2948
城镇居民人均可支配收入	元		4618	7343	9165	11420
常用耕地面积	公顷	24648	23888	23903	23964	24061
粮食产量	吨	121266	121441	136142	130644	146650
农林牧渔业总产值	万元	30798	39861	44759	56021	70344
社会消费品零售总额	万元	22672	30818	34000	40363	71944
普通小学专任教师数	人	1588	1931	1836	1820	1726
普通小学在校学生数	人	44144	24692	22513	21088	20429
普通中学专任教师数	人	1309	1826	1802	1763	1749
普通中学在校学生数	人	23987	28622	28195	25778	23619
卫生机构床位数	张	1074	912	880	980	1083
卫生技术人员	人	1045	839	793	750	988
# 执业(助理)医师	人	335	337	293	320	386
注册护师、护士	人	287	189	208	218	264

?1994-2018 China Academic Journal Electronic Publishing House. All rights reserved. http://www.cnki.net

社会主要指标

2009年	2010年	2011年	2012年	2013年	2014年	2015年	2016年
32.24	32.24	32.31	32.43	32.53	32.60	32.66	32.73
64.76	77.01	92.13	105.01	105.50	107.94	100.70	88.20
4.51	5.84	7.00	7.94	8.74	9.40	9.64	10.05
48.56	58.30	70.20	80.65	78.43	78.44	68.86	53.61
11.69	12.87	14.93	16.42	18.33	20.10	22.20	24.54
46.92	56.34	67.80	78.11	76.66	77.20	67.29	52.28
18342	23887	28547	32441	32482	33146	30862	26976
114.2	115.7	114.3	125.8	108.0	109.2	109.5	103.7
440127	659621	820000	1043243	1307663	1527463	1742333	1880227
48482	40000	45000	39306	34300	34300	30000	21600
84112	115223	150001	195794	144750	151161	183378	171039
3600	4288	5480	6505	7487	8438	8430	9088
14218	16364	19293	22438	24749	27348	25140	26963
24032	24039	24043	23958	23922	23922	23952	23746
152411	147912	108749	119562	112300	110102	117639	114575
76316	101497	121850	138149	155283	166851	171806	179231
83624	95375	112154	130094	155118	175058	198929	225232
1626	1577	1551	1485	1426	1424	1327	1215
20310	20387	19532	14330	13767	13650	14155	14738
1681	1657	1624	1494	1389	1255	1171	1010
20729	18525	16210	12896	12181	11588	10765	10169
1127	1030	1197	1351	1379	912	1373	1747
1006	1036	944	1157	1181	1191	1219	1431
450	470	492	528	532	520	505	64
286	290	297	423	425	426	447	602

?1994-2018 China Academic Journal Electronic Publishing House. All rights reserved. http://www.cnki.net

8-4 潼关县经济

指　　标	单 位	2000年	2005年	2006年	2007年	2008年
年底总人口	万人	14.46	15.78	15.57	15.27	15.38
生产总值	亿元	5.48	7.12	9.19	11.38	14.27
第一产业	亿元	0.74	0.85	0.73	1.03	1.43
第二产业	亿元	2.07	2.01	3.42	4.44	5.76
第三产业	亿元	2.66	4.26	5.04	5.90	7.08
# 工业增加值	亿元	1.73	1.75	2.98	3.99	5.29
人均生产总值	元	3803	4464	5862	7378	9308
生产总值指数	上年=100	102.8	107.4	113.1	113.9	116.0
全社会固定资产投资	万元	11515	14887	18477	34929	63028
地方财政收入	万元	6120	3650	3690	4103	5338
地方财政支出	万元	6117	9035	13232	19508	31133
农村居民人均纯收入	元	1533	1853	1950	2341	2910
城镇居民人均可支配收入	元				7461	10763
常用耕地面积	公顷	11568	9971	9997	10089	10214
粮食产量	吨	36680	43175	36996	48051	48139
农林牧渔业总产值	万元	11343	12354	14543	20330	26499
社会消费品零售总额	万元	17984	25229	28652	32364	43648
普通小学专任教师数	人	892	1258	1198	1126	1080
普通小学在校学生数	人	23117	15929	15271	13769	12030
普通中学专任教师数	人	437	739	775	798	848
普通中学在校学生数	人	9046	15088	16175	16012	16781
卫生机构床位数	张	296	363	363	451	464
卫生技术人员	人	584	594	640	676	681
# 执业(助理)医师	人	116	224	237	258	263
注册护师、护士	人	55	186	184	174	174

?1994-2018 China Academic Journal Electronic Publishing House. All rights reserved.　http://www.cnki.net

社会主要指标

2009年	2010年	2011年	2012年	2013年	2014年	2015年	2016年
15.56	15.56	15.59	15.66	15.76	15.79	15.84	15.91
17.00	20.30	26.80	31.66	34.66	37.16	35.76	37.03
1.51	1.92	2.61	2.99	3.24	3.42	3.52	3.66
7.45	9.61	13.97	17.02	19.11	19.93	16.42	15.77
8.04	8.78	10.22	11.65	12.31	13.81	15.82	17.60
6.80	8.83	13.03	15.90	17.84	18.58	14.78	13.47
11059	13217	17207	20262	22064	23558	22611	23326
115.1	115.1	116.2	115.5	112.5	110.4	106.3	106.4
130638	232321	256600	333952	436600	538087	577135	63984
7268	10018	14680	19230	24530	28010	31742	22306
44510	60068	80018	100518	106600	118600	142800	136600
3524	4302	5382	6388	7391	8381	8368	9063
13317	15394	18196	21089	23346	26101	23911	25693
10379	10417	11449	10654	10720	10661	10593	10660
52855	52858	43106	46481	43100	42009	45339	44039
29620	35871	45705	52526	60204	64299	66261	69086
50855	61104	71912	88602	105972	121674	138248	156679
1025	978	946	1035	860	835	709	681
10571	9578	9004	8856	6572	7475	7656	8847
802	831	975	915	814	754	613	574
15789	14271	11042	10485	7918	7886	7411	6667
463	449	448	545	468	525	536	674
655	650	792	768	816	820	858	890
245	244	231	318	249	259	263	278
179	185	240	230	328	242	258	292

?1994-2018 China Academic Journal Electronic Publishing House. All rights reserved. http://www.cnki.net

8-5 大荔县经济

指 标	单 位	2000年	2005年	2006年	2007年	2008年
年底总人口	万人	68.93	72.30	72.19	71.99	72.06
生产总值	亿元	17.26	26.08	30.02	37.54	45.89
第一产业	亿元	6.73	7.67	7.90	10.84	13.42
第二产业	亿元	2.72	4.67	5.73	7.19	8.29
第三产业	亿元	7.81	13.74	16.39	19.51	24.19
# 工业增加值	亿元	2.33	3.82	4.53	5.59	6.15
人均生产总值	元	2509	3608	4156	5207	6372
生产总值指数	上年=100	106.4	112.4	113.9	114.4	116.2
全社会固定资产投资	万元	34007	45226	66985	133254	252825
地方财政收入	万元	8552	2427	2670	3671	4100
地方财政支出	万元	11509	29740	39206	51552	74874
农村居民人均纯收入	元	1626	2156	2354	2713	3250
城镇居民人均可支配收入	元	3406	5158	5544	8455	10776
常用耕地面积	公顷	73044	72125	71411	71638	72057
粮食产量	吨	255228	195279	193210	214184	308209
农林牧渔业总产值	万元	111191	164666	171458	212373	265320
社会消费品零售总额	万元	68669	120282	139122	165406	219472
普通小学专任教师数	人	2896	3267	3319	3253	3212
普通小学在校学生数	人	91600	50300	56900	53500	51300
普通中学专任教师数	人	2525	3296	3297	3321	3294
普通中学在校学生数	人	52600	61400	68600	63900	62400
卫生机构床位数	张	1055	1073	1073	1072	1072
卫生技术人员	人	808	979	1097	908	1098
# 执业(助理)医师	人	331	459	534	405	438
注册护师、护士	人	277	216	259	237	350

?1994-2018 China Academic Journal Electronic Publishing House. All rights reserved. http://www.cnki.net

社会主要指标

2009年	2010年	2011年	2012年	2013年	2014年	2015年	2016年
69.39	69.39	69.56	69.65	69.75	69.90	70.11	70.29
52.13	63.80	81.23	93.17	96.96	102.83	104.54	115.98
14.11	18.27	23.68	26.63	28.78	31.13	31.96	33.75
9.87	12.91	19.44	21.94	25.67	25.09	21.15	25.23
28.15	32.61	38.11	44.60	42.51	46.61	51.43	57.00
7.00	9.45	15.21	16.94	20.36	22.26	17.92	20.14
7233	8851	11692	13386	13911	14727	14933	16521
114.5	114.5	114.5	113.8	111.9	109.6	108.6	108.3
310705	436649	549320	711220	935331	1175836	1281863	1468776
5540	7239	10510	13311	16610	19174	21111	19603
109720	131032	165158	213522	250532	262388	300869	323746
3851	4660	5969	7051	8070	9184	9306	10106
13315	15431	18147	21200	23596	26168	24264	26193
73303	74173	75493	76399	75705	74380	74031	70692
315671	343211	353901	290095	278100	270535	286422	275547
274530	350657	435449	483972	543715	584115	597803	630040
253931	302531	356178	412646	475266	537858	612249	694628
3226	3102	3020	3075	2893	2861	2785	2495
48400	45477	44385	34874	35235	36839	36705	37366
3314	3261	3315	3201	2997	2950	2888	2674
21400	47203	45326	34119	32522	29103	27303	26317
1111	1230	1883	1866	2247	2475	2475	3225
1424	1559	1634	2310	4304	3511	3763	4042
442	542	453	644	798	913	991	1098
370	516	528	746	948	1105	1228	1426

?1994-2018 China Academic Journal Electronic Publishing House. All rights reserved. http://www.cnki.net

8-6 合阳县经济

指　　标	单 位	2000年	2005年	2006年	2007年	2008年
年底总人口	万人	43.46	44.82	44.73	44.33	44.42
生产总值	亿元	7.96	16.74	20.46	23.86	27.51
第一产业	亿元	2.72	4.29	4.91	5.80	6.83
第二产业	亿元	1.95	2.57	4.47	5.20	5.43
第三产业	亿元	3.28	9.87	11.08	12.87	15.25
# 工业增加值	亿元	1.20	1.97	3.68	4.19	4.16
人均生产总值	元	1866	3265	3782	4543	5356
生产总值指数	上年=100	108.4	112.8	113.3	113.4	114.8
全社会固定资产投资	万元	22538	32424	57227	75729	145160
地方财政收入	万元	6650	2155	2335	3272	4412
地方财政支出	万元	8742	20317	27830	39558	58368
农村居民人均纯收入	元	1380	1580	1682	1858	2360
城镇居民人均可支配收入	元				6480	10410
常用耕地面积	公顷	58054	58384	57603	58058	58815
粮食产量	吨	168013	161041	180547	203879	205766
农林牧渔业总产值	万元	50056	75133	84481	104572	131251
社会消费品零售总额	万元	21998	66289	72848	83293	115664
普通小学专任教师数	人	1787	2260	2237	2066	1981
普通小学在校学生数	人	54075	40446	38649	36416	34694
普通中学专任教师数	人	1292	1742	1883	1890	1981
普通中学在校学生数	人	25688	38375	39633	40309	38312
卫生机构床位数	张	463	474	495	625	781
卫生技术人员	人	796	820	796	760	804
# 执业(助理)医师	人	225	331	317	286	304
注册护师、护士	人	182	187	183	198	214

?1994-2018 China Academic Journal Electronic Publishing House. All rights reserved.　http://www.cnki.net

社会主要指标

2009年	2010年	2011年	2012年	2013年	2014年	2015年	2016年
43.68	43.68	43.77	43.83	43.93	44.02	44.12	44.32
32.87	40.27	50.69	56.96	66.98	71.54	72.64	78.50
7.18	9.45	13.15	15.13	16.07	17.49	18.20	19.18
8.00	11.00	14.55	15.35	21.02	20.21	17.82	17.97
17.69	19.82	22.99	26.49	29.89	33.84	36.62	41.35
6.27	8.93	12.03	12.56	17.93	16.76	16.70	12.63
7402	9070	11593	13005	15265	16268	16485	17752
114.1	114.3	114.2	114.0	110.1	108.9	106.9	106.3
205297	320479	550737	699193	779109	967484	1162898	1356451
6267	10009	14021	17908	21906	24568	26008	18006
78998	109880	125800	169800	192000	211500	233900	253021
3007	3761	4867	5912	6785	7620	7646	8311
13034	15380	17902	20784	23133	25563	23749	25495
57973	58311	58342	58383	58628	58395	58535	58324
247514	274941	201833	220180	207000	197372	207610	203091
138019	176347	240399	274949	310376	332370	344712	363275
134808	159208	188554	218052	250669	286438	327164	373720
1977	1992	1732	1640	1605	1646	1345	1278
32591	30778	27712	22974	21117	20728	20663	21072
1987	2057	2079	2053	1870	2233	2549	1846
37316	34296	31888	28176	25533	23785	20991	19527
806	745	765	696	939	1393	1559	1816
893	1002	1090	1234	1984	2197	2250	2415
312	340	471	368	656	741	726	720
233	269	215	423	686	817	844	933

?1994-2018 China Academic Journal Electronic Publishing House. All rights reserved. http://www.cnki.net

8-7 澄城县经济

指　　标	单 位	2000年	2005年	2006年	2007年	2008年
年底总人口	万人	38.51	38.92	38.76	38.46	38.53
生产总值	亿元	11.00	18.97	21.58	26.20	32.24
第一产业	亿元	3.63	4.60	5.24	7.52	9.08
第二产业	亿元	3.89	8.14	9.44	10.65	12.83
第三产业	亿元	3.48	6.22	6.90	8.03	10.32
# 工业增加值	亿元	3.17	6.82	7.79	8.64	10.13
人均生产总值	元	2857	4887	5556	6786	8374
生产总值指数	上年=100	109.1	112.1	113.8	116.7	118.1
全社会固定资产投资	万元	24042	58373	87147	140052	258639
地方财政收入	万元	6300	4348	4783	6738	9218
地方财政支出	万元	10905	21581	28182	37889	56898
农村居民人均纯收入	元	1270	1653	1819	2165	2673
城镇居民人均可支配收入	元			6684	8467	10785
常用耕地面积	公顷	49100	41160	41046	45343	45653
粮食产量	吨	126508	117621	127288	102846	137101
农林牧渔业总产值	万元	57706	78823	86789	112378	150426
社会消费品零售总额	万元	43522	60234	67573	82056	109132
普通小学专任教师数	人	2016	1676	1790	1777	1739
普通小学在校学生数	人	50200	33700	32500	31800	31206
普通中学专任教师数	人	1436	2006	2105	2127	2095
普通中学在校学生数	人	32700	38000	37500	36600	36100
卫生机构床位数	张	930	949	955	967	990
卫生技术人员	人	1000	927	917	910	954
# 执业(助理)医师	人	482	439	434	429	449
注册护师、护士	人	197	270	292	320	352

?1994-2018 China Academic Journal Electronic Publishing House. All rights reserved. http://www.cnki.net

社会主要指标

2009年	2010年	2011年	2012年	2013年	2014年	2015年	2016年
38.65	38.64	38.73	38.85	38.95	39.03	39.15	39.24
36.81	50.16	65.49	69.60	82.89	85.12	78.30	79.18
9.59	12.03	15.28	17.53	18.87	19.64	20.21	21.30
14.94	23.82	33.26	32.02	39.38	38.15	26.29	22.04
12.29	14.31	16.95	20.05	24.64	27.33	31.80	35.85
11.30	19.48	27.91	25.87	37.98	37.14	25.10	20.38
9552	12453	16144	17943	21309	21831	20030	20201
115.0	116.8	115.8	114.6	113.7	110.2	106.0	101.4
430446	695548	806800	1013932	1203338	1412965	1593556	1027127
12550	16320	22899	28869	35836	40180	40300	28526
79989	102863	135383	166968	183596	200369	236656	239836
3272	3936	5007	5938	6800	7691	7687	8302
13428	15751	18759	22079	24750	27374	25219	27124
45298	45548	45494	45504	46307	46925	46655	46884
182454	212046	164266	177655	175100	167303	174436	175766
156443	206979	271788	311150	343357	368722	380550	402459
126851	149648	177017	203271	234267	265571	302866	345757
1640	1781	1591	1335	1276	1304	1190	1151
24187	20898	21325	18406	17533	17139	16654	16897
2097	2132	2314	2156	2171	2022	1792	1819
33900	32837	30247	26790	24494	22007	19816	18179
998	1180	1120	1270	1420	1496	1796	1755
975	1029	1089	1249	1608	1638	1905	2003
461	487	514	641	683	683	693	594
381	410	435	468	655	701	800	1046

?1994-2018 China Academic Journal Electronic Publishing House. All rights reserved. http://www.cnki.net

8-8 蒲城县经济

指　标	单 位	2000年	2005年	2006年	2007年	2008年
年底总人口	万人	73.37	76.10	75.98	75.82	75.93
生产总值	亿元	21.71	37.57	42.67	49.05	61.56
第一产业	亿元	6.34	8.17	8.92	9.66	13.08
第二产业	亿元	7.66	15.65	18.23	20.95	24.68
第三产业	亿元	7.71	13.75	15.52	18.44	23.80
# 工业增加值	亿元	6.61	14.04	16.17	18.36	21.92
人均生产总值	元	2982	5036	5696	6462	8113
生产总值指数	上年=100	107.9	113.1	115.5	113.5	116.2
全社会固定资产投资	万元	56599	102398	184658	255448	460964
地方财政收入	万元	11252	5340	7200	8822	12201
地方财政支出	万元	14023	26623	37043	49637	81324
农村居民人均纯收入	元	1402	1810	1983	2355	2985
城镇居民人均可支配收入	元	4084	6628	7296	8754	10797
常用耕地面积	公顷	103630	103433	103406	103342	101020
粮食产量	吨	250270	311425	292927	281705	352696
农林牧渔业总产值	万元	102439	105486	144391	182564	225119
社会消费品零售总额	万元	75160	119519	130395	151063	216153
普通小学专任教师数	人	3415	3681	3579	3390	3255
普通小学在校学生数	人	108500	63500	60500	57200	53300
普通中学专任教师数	人	2593	3640	3862	3975	4049
普通中学在校学生数	人	53000	73100	75000	71900	66900
卫生机构床位数	张	796	995	995	995	1471
卫生技术人员	人	1129	1151	1200	1210	1481
# 执业(助理)医师	人	497	521	554	561	908
注册护师、护士	人	202	215	225	231	369

?1994-2018 China Academic Journal Electronic Publishing House. All rights reserved. http://www.cnki.net

社会主要指标

2009年	2010年	2011年	2012年	2013年	2014年	2015年	2016年
74.36	74.35	74.54	74.49	74.59	74.75	74.98	75.04
69.49	83.86	111.08	125.47	139.19	148.42	151.66	163.14
13.60	16.74	19.68	21.34	22.22	23.62	24.37	25.46
28.71	36.07	54.23	63.10	70.70	72.76	68.65	72.07
27.19	31.05	37.17	41.03	46.27	52.05	58.64	65.61
24.98	31.59	48.72	56.97	63.59	65.63	64.80	62.60
9152	11043	14921	16838	18673	19876	20258	21749
114.8	116.5	116.2	114.4	111.8	109.7	110.2	108.8
748061	1008311	1087565	1404123	1652167	2143040	2573714	3030153
19600	25601	36000	45500	54624	61600	65534	55300
115513	151581	196775	237162	265041	304836	348416	372132
3585	4388	5555	6603	7615	8590	8557	9250
13462	16033	19031	22342	24800	27677	25476	27476
101397	100731	99261	97855	94933	93352	91033	88093
392646	405500	336481	360256	330097	329654	352984	342191
231769	284873	335754	363911	411106	440782	455728	479333
250311	294484	347603	403465	466659	529634	603488	688507
3094	2839	2759	2649	2039	2073	2057	2159
49170	46438	41518	39161	33762	32593	43219	44884
4047	4341	4403	4278	4425	4022	3952	3591
60405	54407	47756	44463	31484	27006	34283	31307
1546	1695	1695	1700	2452	2643	3474	3649
1663	1745	1945	2287	2364	3537	3896	3399
1059	1140	1228	1244	1286	1323	1356	1459
428	529	608	652	705	811	1550	1660

?1994-2018 China Academic Journal Electronic Publishing House. All rights reserved. http://www.cnki.net

8-9 白水县经济

指　　标	单 位	2000年	2005年	2006年	2007年	2008年
年底总人口	万人	27.74	28.13	27.91	27.61	27.59
生产总值	亿元	7.95	13.61	16.98	19.72	24.69
第一产业	亿元	2.95	4.50	5.49	6.89	8.12
第二产业	亿元	2.20	3.27	4.75	4.76	6.30
第三产业	亿元	2.79	5.84	6.74	8.07	10.27
# 工业增加值	亿元	1.62	2.69	4.07	4.10	5.40
人均生产总值	元	2882	4904	6060	7104	8946
生产总值指数	上年=100	103.3	110.1	113.5	113.1	115.3
全社会固定资产投资	万元	15492	31000	36034	49102	89474
地方财政收入	万元	5306	2425	3130	4100	5500
地方财政支出	万元	7417	16845	23048	31521	45613
农村居民人均纯收入	元	1162	1448	1616	1957	2467
城镇居民人均可支配收入	元				7243	10464
常用耕地面积	公顷	27917	29251	28687	28340	28255
粮食产量	吨	80691	79712	98478	112701	116302
农林牧渔业总产值	万元	46749	69273	91426	118886	137484
社会消费品零售总额	万元	36052	44995	51004	58595	77336
普通小学专任教师数	人	1427	1717	1744	1776	1773
普通小学在校学生数	人	41200	26800	24199	21602	20078
普通中学专任教师数	人	885	1431	1589	1611	1634
普通中学在校学生数	人	21100	27849	27934	26710	25977
卫生机构床位数	张	248	408	350	478	499
卫生技术人员	人	310	452	651	546	575
# 执业(助理)医师	人	101	201	223	275	291
注册护师、护士	人	97	137	152	161	173

?1994-2018 China Academic Journal Electronic Publishing House. All rights reserved.　http://www.cnki.net

社会主要指标

2009年	2010年	2011年	2012年	2013年	2014年	2015年	2016年
27.99	27.99	28.05	28.17	28.27	28.33	28.39	28.34
28.36	37.85	47.69	54.65	53.09	60.96	60.05	63.12
8.58	13.19	16.02	18.10	20.27	22.79	23.72	24.98
7.56	10.40	15.13	17.50	14.86	17.95	13.44	12.47
12.21	14.26	16.54	19.05	17.96	20.21	22.88	25.67
6.32	8.90	13.03	16.26	14.36	17.45	13.33	12.36
10279	13723	17020	19441	18814	21540	21700	22252
114.5	115.0	114.0	114.2	112.1	108.6	106.8	106.0
162173	264156	377599	441076	611437	772468	891993	926857
7500	10006	14105	17700	21510	23700	24200	11300
61899	90582	114208	140294	152612	167935	188745	206769
3210	4070	5250	6242	7215	8218	8314	9046
13086	15389	17959	20922	23202	25824	23923	25754
28214	28355	28533	28577	28704	28718	28735	26165
115652	137284	111129	117880	110759	105208	111623	109079
146905	227528	273846	313727	359886	398236	414710	437159
90247	110574	131007	150523	177284	202726	231956	263575
1609	1560	1513	1423	1500	1183	1127	1086
18530	17682	17322	14193	13913	13279	13679	14138
1629	1562	1706	1601	1552	1519	1432	1395
15010	13107	21105	19142	17058	15431	13592	12183
435	480	556	522	526	592	914	1083
733	1011	1123	1207	1129	1292	1360	1425
291	189	257	208	228	291	315	306
173	193	174	182	223	296	486	447

?1994-2018 China Academic Journal Electronic Publishing House. All rights reserved.   http://www.cnki.net

8-10 富平县经济

指　　标	单 位	2000年	2005年	2006年	2007年	2008年
年底总人口	万人	75.53	76.96	76.81	76.51	76.62
生产总值	亿元	13.50	25.87	30.84	36.57	44.55
第一产业	亿元	4.24	7.75	8.09	10.32	13.28
第二产业	亿元	3.14	7.94	10.00	11.20	13.43
第三产业	亿元	6.11	10.18	12.75	15.05	17.84
# 工业增加值	亿元	2.63	7.15	8.69	10.26	12.17
人均生产总值	元	1792	3412	4048	4770	5818
生产总值指数	上年=100	109.2	111.0	112.8	113.9	115.5
全社会固定资产投资	万元	17859	63362	89147	124257	196551
地方财政收入	万元	8703	3594	3829	5117	7003
地方财政支出	万元	11862	27953	38382	53031	90489
农村居民人均纯收入	元	1454	1922	1924	2366	2945
城镇居民人均可支配收入	元	3200	5547	6290	7830	10784
常用耕地面积	公顷	74871	71272	71245	71179	71122
粮食产量	吨	280985	343325	390618	404812	405495
农林牧渔业总产值	万元	90214	127893	138639	167294	205764
社会消费品零售总额	万元	58785	121540	130496	151161	203067
普通小学专任教师数	人	3146	3690	3651	3487	3407
普通小学在校学生数	人	103900	70860	64071	59906	54510
普通中学专任教师数	人	2673	3481	3677	3868	4030
普通中学在校学生数	人	55600	71216	71817	72394	69326
卫生机构床位数	张	919	1173	1355	1355	2120
卫生技术人员	人	1602	2690	1549	1457	1486
# 执业(助理)医师	人	405	480	500	515	525
注册护师、护士	人	416	490	498	511	521

?1994-2018 China Academic Journal Electronic Publishing House. All rights reserved.　http://www.cnki.net

社会主要指标

2009年	2010年	2011年	2012年	2013年	2014年	2015年	2016年
74.40	74.39	74.61	74.68	74.78	74.94	75.17	75.21
50.01	60.43	80.02	94.00	117.91	126.60	128.98	137.39
13.70	16.96	20.61	23.02	25.69	26.33	27.10	28.44
15.48	19.68	32.18	39.35	57.62	60.49	57.12	58.55
20.84	23.79	27.23	31.63	34.60	39.80	44.76	50.40
13.75	17.62	29.67	36.46	54.50	65.71	55.12	53.53
6526	7886	10740	12593	15779	16914	17184	18273
114.0	114.4	115.1	115.4	112.7	111.2	110.1	109.2
329498	465245	632245	815418	1030000	1299700	1573000	1858000
10792	14491	22211	28693	36276	40365	44634	45000
120641	159496	203315	251983	281364	308006	350247	372205
3581	4329	5524	6551	7514	8476	8515	9171
13412	15718	18673	22015	24591	27321	25417	27468
71193	71554	71132	70987	71002	70814	70171	68101
413882	441645	363948	379999	360006	355004	372374	360201
214605	285970	355447	397056	445089	477479	494383	520442
234965	286719	338064	390653	451395	511263	581531	661500
3369	3246	2678	2960	2748	2882	2476	2824
51104	47793	45340	39187	34386	36974	37128	37601
4238	4269	4224	4048	3793	2440	3555	3113
65144	59584	53561	48827	38142	21390	33126	29821
1560	1655	1561	2220	2357	2221	2270	2700
1520	1845	2089	2140	2314	2429	2448	3321
535	1040	1082	1090	1135	1140	1250	1534
531	720	765	810	1027	1045	1145	1746

?1994-2018 China Academic Journal Electronic Publishing House. All rights reserved. http://www.cnki.net

8-11 韩城市经济

指　　标	单 位	2000年	2005年	2006年	2007年	2008年
年底总人口	万人	38.25	39.53	39.49	39.32	39.43
生产总值	亿元	17.57	56.90	70.30	80.86	101.10
第一产业	亿元	3.26	4.54	5.66	6.62	7.56
第二产业	亿元	9.63	36.94	46.70	52.48	67.93
第三产业	亿元	4.68	15.42	17.94	21.76	25.61
# 工业增加值	亿元	8.99	33.98	43.65	49.17	63.48
人均生产总值	元	4638	15531	18069	21561	25676
生产总值指数	上年=100	108.1	115.8	117.2	115.2	116.6
全社会固定资产投资	万元	39806	279913	227819	318020	467396
地方财政收入	万元	9205	16273	21125	31459	43008
地方财政支出	万元	11666	28851	38325	57623	80155
农村居民人均纯收入	元	1708	2179	2496	3213	3972
城镇居民人均可支配收入	元	3655	7046	8019	9726	12386
常用耕地面积	公顷	28678	26107	26252	26192	26047
粮食产量	吨	97416	72597	93675	68132	86138
农林牧渔业总产值	万元	53595	66737	80186	98260	119615
社会消费品零售总额	万元	47788	84068	97905	116845	151513
普通小学专任教师数	人	1737	2337	2317	2203	2142
普通小学在校学生数	人	49604	35185	33189	31159	29026
普通中学专任教师数	人	1453	2241	2306	2222	2228
普通中学在校学生数	人	28643	36500	35700	33500	31400
卫生机构床位数	张	635	1216	1434	1203	1200
卫生技术人员	人	824	1792	1771	1794	1804
# 执业(助理)医师	人	346	986	912	817	820
注册护师、护士	人	478	703	756	871	861

?1994-2018 China Academic Journal Electronic Publishing House. All rights reserved. http://www.cnki.net

社会主要指标

2009年	2010年	2011年	2012年	2013年	2014年	2015年	2016年
39.15	39.15	39.25	39.53	39.63	39.71	39.86	39.98
120.71	145.22	197.28	221.95	287.20	299.78	308.64	319.70
7.66	9.37	11.54	12.94	13.24	13.82	14.33	15.19
84.18	103.24	147.71	166.91	219.00	225.82	228.06	230.88
28.87	32.61	38.03	42.10	54.96	60.14	66.25	73.63
78.19	96.10	138.90	157.24	207.67	210.95	210.76	205.92
30091	36791	50325	56347	72561	75568	77577	80086
116.8	118.2	120.6	113.6	113.9	111.2	110.4	111.7
693954	947184	1204021	1565642	2007864	2523942	3112607	4067211
55208	69822	87065	153156	190263	170031	187655	216239
107521	151609	189025	211518	280945	307921	344624	386603
4846	5854	7499	8852	10179	11400	11429	12400
15501	18042	21308	24717	27312	30071	27504	29784
26070	26107	26406	26882	27240	25305	23370	24709
79493	87302	77541	83268	77600	72790	72576	68721
123472	158898	205574	231794	258311	276872	287983	305760
176252	215139	253057	289210	326808	369903	421444	482436
2020	1905	1593	1446	1692	1569	1533	1172
26819	25443	24243	22365	22001	21526	21047	20982
2164	2172	2385	2379	2034	1849	1798	1906
29700	27469	25686	23105	21281	20594	19848	20041
1200	1200	1558	1558	1995	2068	1968	2073
1810	1814	2366	2780	3090	3158	3125	3509
864	868	938	1075	1305	1097	1287	1164
459	459	753	1080	1121	1068	1325	1497

?1994-2018 China Academic Journal Electronic Publishing House. All rights reserved.   http://www.cnki.net

8-12 华阴市经济

指　　标	单 位	2000年	2005年	2006年	2007年	2008年
年底总人口	万人	25.95	26.24	26.16	25.86	25.97
生产总值	亿元	10.19	19.67	20.83	26.95	30.75
第一产业	亿元	1.45	1.95	1.96	2.36	2.99
第二产业	亿元	5.21	9.54	9.72	12.67	14.06
第三产业	亿元	3.53	8.18	9.15	11.92	13.70
# 工业增加值	亿元	4.79	8.28	8.67	11.36	12.01
人均生产总值	元	4016	7679	8033	10383	11629
生产总值指数	上年=100	121.0	112.3	112.0	114.2	115.4
全社会固定资产投资	万元	37519	52903	54533	74186	156492
地方财政收入	万元	4710	3550	4844	6101	8608
地方财政支出	万元	7138	13119	19254	25096	38238
农村居民人均纯收入	元	1345	1703	1891	2409	2968
城镇居民人均可支配收入	元	5477	6674	7350	8819	10968
常用耕地面积	公顷	14049	13022	13043	13051	13052
粮食产量	吨	67980	83760	80452	63006	83636
农林牧渔业总产值	万元	19215	28476	30388	37407	46053
社会消费品零售总额	万元	29946	48764	55788	66784	87185
普通小学专任教师数	人	946	1528	1568	3965	3988
普通小学在校学生数	人	32900	20300	18300	16600	15000
普通中学专任教师数	人	848	1220	1230	1217	1196
普通中学在校学生数	人	14000	18200	17800	16700	15400
卫生机构床位数	张	886	1172	1121	1170	1143
卫生技术人员	人	963	1135	1135	1125	1067
# 执业(助理)医师	人	297	368	369	366	390
注册护师、护士	人	256	270	265	263	265

?1994-2018 China Academic Journal Electronic Publishing House. All rights reserved.   http://www.cnki.net

社会主要指标

2009年	2010年	2011年	2012年	2013年	2014年	2015年	2016年
25.83	25.83	25.89	26.03	26.13	26.19	26.26	26.28
40.49	48.75	58.31	65.41	73.73	78.77	78.12	70.02
3.15	3.57	4.41	5.04	5.25	5.63	5.79	6.05
21.46	26.94	32.70	36.72	38.60	39.95	34.81	22.37
15.89	18.24	21.20	23.65	29.88	33.19	37.52	41.61
18.70	23.62	28.60	31.87	34.18	38.27	33.82	20.89
15588	18765	22548	25196	28272	30111	29788	26655
115.3	115.2	115.0	115.0	113.5	111.6	106.9	100.1
286569	489608	585214	735121	962916	1216500	1443800	990559
12133	15835	22674	28844	35233	38701	39315	27518
53267	74055	94208	123223	126280	127778	145994	152133
3621	4287	5389	6402	7325	8241	8236	8862
13612	15871	18712	21668	24246	26719	24611	26567
12996	12728	12798	12758	12727	12703	12580	12557
95504	97623	73768	80480	78100	77000	82176	80052
48894	60345	75313	85727	97577	104549	107973	113091
100971	125691	148939	173492	196026	223045	253254	287296
1399	1088	1459	1186	1111	1082	924	979
14500	14122	13623	12497	12415	13160	12677	14300
1135	1059	1230	968	927	777	1137	787
14000	11162	12490	9421	8776	7981	8737	8050
1366	1063	1105	1143	1141	1241	1261	1296
1225	1497	1097	1192	1137	1159	1199	1187
395	297	291	478	502	503	300	316
270	284	299	271	402	415	433	417

?1994-2018 China Academic Journal Electronic Publishing House. All rights reserved. http://www.cnki.net
