# Supplementary material for: A study on the tourism efficiency of tourism destination based on DEA model: A case of ten cities in Shaanxi province
Source: PLoS One. 2024 Jan 19;19(1):e0296660. doi: 10.1371/journal.pone.0296660 (PMC10798521; doi:10.1371/journal.pone.0296660)
Supplement: S1 File — (ZIP) [file pone.0296660.s001.zip › Supporting information/Statistical yearbook/Xi'an.caj]

四、西安市

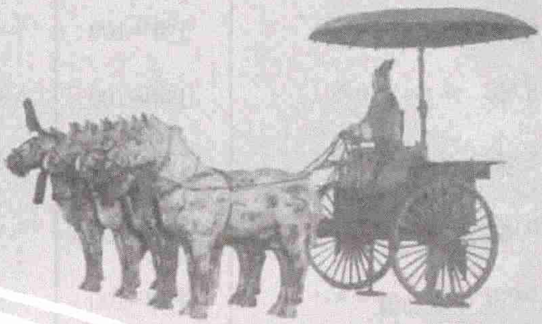

资料整理：赵　博　金文娟

?1994-2018 China Academic Journal Electronic Publishing House. All rights reserved.　http://www.cnki.net

4-1 西安市经济

指　标	单 位	2000年	2005年	2006年	2007年	2008年
年底总人口	万人	688.01	806.81	822.52	830.54	837.52
人口自然增长率	‰		4.42	4.52	4.52	4.58
年底总户数	万户	187.08	203.05	207.04	211.12	216.52
生产总值	亿元	646.13	1313.93	1538.94	1856.63	2318.14
第一产业	亿元	44.65	66.01	70.44	82.51	103.45
第二产业	亿元	277.13	540.50	645.65	781.94	981.58
第三产业	亿元	324.35	707.42	822.85	992.18	1233.11
# 工业增加值	亿元	218.44	420.00	494.22	594.95	721.40
人均生产总值	元	9484	16406	18890	22463	27794
生产总值指数	上年=100	113.0	114.0	114.0	115.6	116.3
第一产业	上年=100	103.5	107.5	107.1	104.5	107.6
第二产业	上年=100	115.1	112.3	113.7	115.7	116.4
第三产业	上年=100	111.5	116.3	114.9	116.4	116.9
# 工业增加值	上年=100	113.8	110.3	112.3	114.9	115.7
人均生产总值指数	上年=100	111.4	112.2	112.9	113.9	115.3
非公有制经济增加值	亿元		568.45	684.66	854.26	1103.95
文化产业增加值	亿元					
单位GDP能耗	吨标准煤/万元		1.030	0.987	0.930	0.869
单位GDP能耗比上年增长	%			-4.15	-5.75	-6.65
就业人员	万人	389.10	415.83	422.15	436.36	448.05
城镇单位就业人员	万人	112.45	123.66	125.10	129.40	130.85
# 国有单位	万人	84.21	81.98	82.84	83.22	83.38
集体单位	万人	13.35	10.00	9.47	9.23	8.73
# 在岗职工人数	万人	105.56	119.73	121.06	125.56	126.89
城镇单位就业人员平均工资	元					
城镇单位在岗职工平均工资	元	9197	17728	20475	25012	29749

?1994-2018 China Academic Journal Electronic Publishing House. All rights reserved.　http://www.cnki.net

社会主要指标

2009年	2010年	2011年	2012年	2013年	2014年	2015年	2016年
843.46	847.41	851.34	855.29	858.81	862.75	870.56	883.21
4.45	4.39	4.33	4.56	4.20	4.64	4.64	6.14
221.51	226.71	234.34	239.54	245.53	250.26	253.13	256.65
2724.88	3242.86	3869.84	4394.47	4924.97	5492.64	5801.20	6282.65
110.38	140.06	173.14	195.59	200.45	214.55	220.20	232.01
1144.75	1357.53	1583.21	1781.09	1998.82	2194.78	2126.29	2200.36
1469.75	1745.27	2113.49	2417.79	2725.70	3083.31	3454.71	3850.28
816.92	954.38	1098.51	1228.05	1376.74	1488.02	1376.72	1397.25
32420	38357	45561	51499	57464	63794	66938	71647
114.5	114.5	113.5	112.2	111.1	109.9	108.2	108.6
106.3	106.9	106.7	106.0	104.7	105.1	105.0	103.8
112.8	115.2	112.5	112.0	113.6	109.3	105.6	108.5
116.3	114.5	114.9	112.9	109.7	110.7	110.4	109.0
110.1	114.3	112.8	112.7	114.0	108.4	104.8	109.2
113.7	113.8	113.0	111.7	110.6	109.4	107.5	107.4
1327.87	1611.71	1956.41	2258.95	2569.20	2892.90	3060.38	3314.20
					410.04	451.62	501.54
0.820	0.575	0.555	0.535	0.516	0.486	0.470	0.394
-5.56	-2.06	-3.56	-3.51	-3.57	-5.89	-3.20	-3.83
462.52	477.58	495.99	514.57	530.71	532.92	528.06	539.18
135.64	140.38	154.30	179.76	198.42	193.82	188.97	187.89
85.42	88.84	91.60	108.31	84.98	79.24	75.04	75.84
6.41	4.75	5.33	5.10	7.30	6.67	5.29	4.85
129.61	130.70	142.71	169.45	183.41	177.62	181.86	173.70
			46248	50988	53974	60213	67428
34032	37870	41083	47566	52619	55897	63193	70011

?1994-2018 China Academic Journal Electronic Publishing House. All rights reserved. http://www.cnki.net

4-1 续表 1

指　　标	单 位	2000年	2005年	2006年	2007年	2008年
全社会固定资产投资	亿元	233.15	835.10	1066.62	1435.33	1906.36
# 房地产开发	亿元	52.48	212.29	285.76	387.33	545.71
商品房销售面积	万平方米	212.92	497.34	621.50	833.92	760.72
# 住宅	万平方米	200.77	476.39	584.06	782.91	715.75
地方财政收入	亿元	41.40	73.05	85.89	112.92	145.61
地方财政支出	亿元	46.50	97.82	119.22	161.25	226.99
金融机构人民币各项存款余额	亿元	1335.63	3599.70	4066.16	4582.71	5749.35
金融机构人民币各项贷款余额	亿元	972.51	2158.10	2344.77	2683.77	3275.12
农村居民人均纯收入	元	2344	3460	3808	4399	5212
城镇居民人均可支配收入	元	6364	9628	10905	12662	15207
城市人均公园绿地面积	平方米			7.6	7.6	7.8
城市人均道路面积	平方米	5.1	8.1	9.9	12.7	14.0
城市用水普及率	%	99.0	99.0	99.1	100.0	100.0
城市燃气普及率	%	81.5	91.3	92.6	98.6	97.7
常用耕地面积	千公顷	295.58	266.78	263.86	261.18	260.51
农林牧渔业总产值	亿元	74.37	106.54	114.15	134.14	168.27
农作物总播种面积	千公顷	523.29	505.27	512.99	508.25	504.04
# 粮食作物	千公顷	465.03	428.50	432.00	424.70	420.21
粮食产量	万吨	201.92	205.53	212.85	189.06	214.40
棉花产量	吨	1367	4515	4812	5903	6173
油料产量	吨	13692	11554	10800	9555	11544
蔬菜产量	吨	1621400	1956992	1893000	2044395	2215303

?1994-2018 China Academic Journal Electronic Publishing House. All rights reserved. http://www.cnki.net

2009年	2010年	2011年	2012年	2013年	2014年	2015年	2016年
2500.13	3250.56	3352.12	4243.43	5134.56	5903.98	5165.98	5191.36
696.34	842.34	996.81	1281.90	1595.64	1761.88	1831.67	1955.82
1256.02	1587.81	1778.02	1538.91	1662.75	1707.71	1763.68	2047.67
1202.12	1523.24	1674.85	1383.87	1522.50	1525.95	1584.08	1877.78
181.40	241.86	318.55	396.96	501.98	583.79	650.99	641.07
276.85	371.62	494.58	597.49	729.81	819.54	917.24	942.52
7522.08	8933.23	10430.27	12125.53	13763.19	15166.78	17796.38	19073.96
4482.63	6482.28	7564.93	8635.22	10023.63	11668.14	13714.02	15282.65
6275	7750	9788	11442	12930	12898	14072	15191
18963	22244	25981	29982	33100	30715	33188	35630
7.9	9.5	10.4	10.8	11.2	11.6	11.7	11.9
14.8	15.6	16.0	17.9	18.0	18.1	18.3	18.3
100.0	100.0	100.0	100.0	100.0	100.0	100.0	100.0
98.2	99.3	99.9	100.0	100.0	100.0	100.0	100.0
258.59	255.54	251.40	246.61	244.15	240.49	237.93	231.20
178.70	227.10	272.66	308.36	342.89	367.21	380.76	405.63
504.74	501.16	469.42	467.71	466.32	457.43	450.33	439.43
419.13	414.47	382.08	381.67	378.58	367.64	358.46	351.89
218.20	221.65	182.04	192.54	183.12	175.61	180.86	175.33
6251	5861	5649	4721	2467	328	294	237
11217	12000	11722	10176	9994	9785	9478	8649
2424110	2531000	2615200	2778013	2981225	3162800	3327900	3367449

?1994-2018 China Academic Journal Electronic Publishing House. All rights reserved. http://www.cnki.net

4-1 续表 2

指 标	单 位	2000年	2005年	2006年	2007年	2008年
水果产量	吨	343551	512869	553433	603193	716902
# 苹果	吨	89416	53387	51809	52180	53194
肉类产量	吨	147571	182046	108634	102191	115352
# 猪牛羊肉	吨	122969	156640	170099	84954	97829
奶类产量	吨	245913	422229	471438	528037	589697
# 牛奶	吨	176155	327961	374813	428462	475681
禽蛋产量	吨	138305	118115	97816	98140	108515
水产品产量	吨	11384	9370	11937	12402	12487
规模以上工业企业单位数	个	816	901	904	937	1032
规模以上工业总产值	亿元	433.43	952.18	1194.60	1623.85	2030.76
纱产量	万吨	6.56	6.72	6.47	6.99	5.88
布产量	万米	27819	27012	26508	27995	22721
发电量	亿千瓦小时	19.24	48.04	66.80	71.03	71.69
粗钢产量	万吨	3.06	1.29	1.39	1.10	1.40
钢材产量	万吨	1.04	24.02	71.53	78.73	47.88
水泥产量	万吨	107.21	121.01	156.57	263.13	404.76
汽车产量	辆	8832	41325	103469	170596	268191
建筑业企业单位数	个	200	223	212	274	329
建筑业企业年末从业人员	万人	13.67	15.94	17.31	25.49	34.89
建筑业总产值	亿元	105.93	326.65	416.48	604.65	915.12
房屋建筑施工面积	万平方米	793.32	1773.81	2140.12	2940.92	3236.53
房屋建筑竣工面积	万平方米	336.80	566.19	594.20	835.12	1076.14
公路里程	公里	3010	3901	9672	11063	11895
# 等级公路	公里	2908	6542	7139	7281	10891
# 高速公路	公里	100	240	286	324	374
民用汽车拥有量	辆	179014	330480	393778	473216	595735
# 私人汽车	辆	63146	205442	258081	330507	452347
邮电业务总量	亿元	39.89	149.06	186.76	226.76	264.86
邮政业务总量	亿元	1.70	5.94	7.10	7.64	8.41
电信业务总量	亿元	38.19	126.10	179.66	219.13	256.45

?1994-2018 China Academic Journal Electronic Publishing House. All rights reserved. http://www.cnki.net

2009年	2010年	2011年	2012年	2013年	2014年	2015年	2016年
789587	847822	911361	932054	951851	996570	1052068	1077442
53023	39130	34313	28675	25212	26047	32038	30522
126182	136501	144631	151711	158449	144209	161254	156829
108309	117025	120321	126282	130467	114481	132601	129510
618186	633663	647978	666439	657748	658016	637340	562038
498394	509178	509777	513337	512796	514587	485640	420765
116685	123793	125639	129970	135382	135191	140175	140357
13044	11850	13350	14010	14200	14168	14120	14106
1134	1126	890	966	1002	1113	1117	1189
2490.50	3130.15	3552.21	4066.31	4497.62	4420.06	4346.16	4669.32
4.68	4.32	3.93	3.40	2.70	2.24	3.52	4.21
22202	23811	16344	14000	14000	11219	10400	12200
83.21	96.94	95.01	99.50	184.50	179.58	158.68	
0.92	2.37	2.14	0.90				
110.51	110.77	18.48	31.30	54.30	42.87	37.03	43.92
487.72	236.50	409.99	534.20	391.10	412.69	330.73	217.71
506758	652050	556664	542000	422000	374714	341400	382500
326	322	334	390	415	531	697	882
44.31	53.90	49.21	39.12	57.79	39.41	59.53	68.59
1296.58	1820.34	1900.72	1874.23	2228.41	2586.33	2648.19	2877.55
3947.01	4592.57	6183.02	7531.07	9753.45	11182.18	11974.53	11945.73
1209.88	1391.91	2610.85	1985.31	2229.97	2536.71	2709.14	2233.20
12378	12575	12599	13127	13135	13251	13328	13336
11839	12118	12142	12587	12598	12733	12805	12834
377	371	382	465	471	471	532	534
754803	957162	1168238	1380125	1634709	1924402	2189116	2442084
596835	778902	970440	1174425	1412211	1703443	1972289	2219569
275.65	102.51	143.46	167.35	176.21	247.13	328.05	538.14
9.07	6.88	8.69	14.40	16.58	22.67	33.23	53.13
266.58	95.63	134.77	152.95	159.63	224.46	294.8	485.0

?1994-2018 China Academic Journal Electronic Publishing House. All rights reserved. http://www.cnki.net

4-1 续表 3

指　标	单 位	2000年	2005年	2006年	2007年	2008年
固定电话用户	万户	141.28	324.04	330.07	334.99	332.48
移动电话用户	万户	69.01	372.61	480.21	605.70	693.53
互联网宽带用户	万户	20.71	33.93	50.88	58.62	81.40
限额以上企业数	个					
批发业	个					
零售业	个					
住宿业	个					
餐饮业	个					
社会消费品零售总额	亿元	360.42	670.56	784.95	936.21	1176.58
进出口总额	万美元	173696	390146	415393	536162	701366
# 出口	万美元	106062	263441	272852	347133	444508
实际外商直接投资额	万美元	15633	57112	82463	105317	114738
入境旅游人数	万人次	65.03	77.56	86.73	100.01	63.20
# 外国人	万人次	54.65	65.86	73.40	85.09	53.58
国内旅游人数	万人次	1502	2423	2738	3118	3232
国际旅游外汇收入	万美元	27000	40900	46700	54323	35900
国内旅游收入	亿元	82.59	144.96	166.47	194.77	214.80
星级饭店数	个		63	63	92	93
幼儿园数	所	367	737	863	830	905
在园儿童数	万人	12.85	12.75	13.38	14.00	15.46
普通小学学校数	所	2323	1980	1929	1872	1781
普通小学专任教师数	人	30215	29674	30018	30533	30382
普通小学在校学生数	万人	77.81	60.47	59.33	56.83	54.66
普通中学学校数	所	466	460	457	453	442
普通中学专任教师数	人	26230	31094	31203	31373	31425
普通中学在校学生数	万人	48.31	55.74	56.11	54.68	52.83
卫生机构数	个	1453	1478	1478	1214	1071
卫生机构床位数	张	31690	32323	32137	33598	34618
卫生技术人员	人	42243	42241	43862	43707	47431
# 执业(助理)医师	人	18473	17721	18007	17266	18066
注册护师、护士	人	13543	14000	15538	15337	17186

?1994-2018 China Academic Journal Electronic Publishing House. All rights reserved.　http://www.cnki.net

2009年	2010年	2011年	2012年	2013年	2014年	2015年	2016年
305.44	297.99	305.16	313.82	321.32	322.91	314.39	298.61
940.45	986.56	1141.70	1322.50	1511.07	1462.32	1370.63	1445.23
116.79	146.18	172.44	189.39	219.95	246.12	265.29	339.86
844	894	980	1080	1219	1341	1367	1582
162	178	200	240	282	342	359	423
260	275	299	335	401	461	496	591
156	177	189	203	217	223	226	246
266	264	292	302	319	315	286	322
1398.37	1678.01	2039.24	2400.67	2742.89	3093.89	3405.38	3767.20
725234	1038293	1257852	1301433	1798534	1532.15(亿元)	1761.75(亿元)	1829.95(亿元)
333271	531713	580369	729865	847819	734.68(亿元)	819.87(亿元)	947.31(亿元)
121873	156665	200522	247856	312994	370318	400833	450461
67.29	84.18	100.23	115.35	121.11			
59.09	73.21	88.63	101.40	106.89	12000	13601	15012
3929	5201	6553	7863	10009			
39000	53000	64100	74862	80200	950(亿元)	1073.69(亿元)	1213.81(亿元)
266.35	362.78	478.87	594.50	747.28			
104	116	116	116	116	109	111	102
896	1004	1122	1239	1295	1343	1417	1475
16.30	18.40	24.03	27.08	28.56	28.95	30.90	31.80
1666	1531	1424	1322	1291	1257	1234	1190
30334	29944	28453	28146	27954	28395	28748	30941
52.52	51.60	51.39	50.85	51.95	53.79	56.62	59.79
439	436	423	419	418	421	422	422
31415	31506	33122	31526	32886	32615	31183	33962
50.63	48.90	47.20	45.33	43.73	42.57	41.37	40.70
1017	1117	5554	5578	5692	5742	5802	5869
36849	39407	41010	44239	47867	51065	54708	56332
51641	56579	61281	66899	71553	76375	81462	86258
19284	20913	21551	23051	24015	24931	26626	27864
20167	22640	25043	27837	30062	32210	34819	37518

?1994-2018 China Academic Journal Electronic Publishing House. All rights reserved. http://www.cnki.net

4-2 新城区经济

指 标	单 位	2000年	2005年	2006年	2007年	2008年
年底总人口	万人	46.95	62.24	63.92	64.32	64.43
生产总值	亿元	77.84	144.81	167.94	198.47	244.22
第一产业	亿元	0.02	0.01	0.01		
第二产业	亿元	37.84	67.85	79.19	84.74	97.39
第三产业	亿元	39.98	76.95	88.74	113.73	146.83
# 工业增加值	亿元	6.97	55.99	63.56	64.43	66.81
人均生产总值	元	16587	23475	26623	31312	38969
生产总值指数	上年=100	117.9	108.4	112.2	114.0	114.1
全社会固定资产投资	万元	27100	625600	820000	1120141	1513400
地方财政收入	万元	24900	61072	69097	93033	108054
地方财政支出	万元	24712	40892	52588	64032	79495
农村居民人均纯收入	元	5571				
城镇居民人均可支配收入	元					15213
常用耕地面积	公顷	127	1	11		
粮食产量	吨	247	2	4	4	
农林牧渔业总产值	万元	388	111	113	40	
社会消费品零售总额	万元	706800	1290046	1466969	1699511	2070063
普通小学专任教师数	人	1947	1711	1815	1816	1773
普通小学在校学生数	人	44312	43858	44710	44096	42271
普通中学专任教师数	人	1933	2295	2144	2092	2106
普通中学在校学生数	人	35576	44093	40551	40226	39146
卫生机构床位数	张	530	5527	5503	5597	5620
卫生技术人员	人		6428	7138	7710	8831
# 执业(助理)医师	人		2707	2843	3112	3326
注册护师、护士	人		2361	2862	3089	4162

?1994-2018 China Academic Journal Electronic Publishing House. All rights reserved. http://www.cnki.net

社会主要指标

2009年	2010年	2011年	2012年	2013年	2014年	2015年	2016年
62.80	59.01	59.23	59.44	59.64	59.86	60.33	60.91
278.99	314.61	363.56	394.87	429.07	467.08	494.00	540.66
111.55	132.51	150.45	171.74	177.99	186.67	178.21	192.05
167.44	182.10	213.11	223.13	251.08	280.41	315.79	348.61
64.52	81.77	90.35	103.68	110.38	95.84		108.52
45217	52470	61495	66544	72064	78172	82203	89188
113.6	113.7	113.3	111.0	109.2	109.0	106.4	108.2
2036600	2637266	2884809	3535000	3786700	4002742	2892500	2537100
130002	163588	205654	269944	320736	355811	370669	364816
107883	126076	170555	212440	282246	311201	341540	377029
19049	22554	26498	30658	33816	37029	34622	37212
2415613	2854070	3424804	3970835	4460631	4997780	5575912	6079842
1773	1784	1757	1739	1718	1522	1537	1911
40955	39653	38594	37322	37121	36804	36920	36618
1996	1982	1980	1980	1974	2051	2035	2270
37941	37658	37211	36771	36019	35501	34188	33940
6123	6422	6236	6637	6988	7424	6964	7065
9461	10393	9930	10725	11248	11774	10893	11343
3561	3749	3606	3736	3750	3870	3740	3172
4579	4630	4549	5009	5422	5677	5178	5051

?1994-2018 China Academic Journal Electronic Publishing House. All rights reserved.   http://www.cnki.net

4-3 碑林区经济

指　　标	单　位	2000年	2005年	2006年	2007年	2008年
年底总人口	万人	61.04	83.29	84.90	83.36	82.75
生产总值	亿元	76.12	145.99	169.00	201.04	250.17
第一产业	亿元					
第二产业	亿元	12.19	27.01	33.03	39.29	50.32
第三产业	亿元	63.93	118.98	135.97	161.75	199.85
# 工业增加值	亿元		9.47	11.59	13.33	15.39
人均生产总值	元	12700	17856	20095	25919	36003
生产总值指数	上年=100	124.5	111.4	112.9	115.0	115.6
全社会固定资产投资	万元	37700	673960	911314	1257158	1674500
地方财政收入	万元	24066	76246	87859	109759	146616
地方财政支出	万元	22100	45937	51501	64902	78572
农村居民人均纯收入	元	5097				
城镇居民人均可支配收入	元					15508
常用耕地面积	公顷	44				
粮食产量	吨	14				
农林牧渔业总产值	万元	29				
社会消费品零售总额	万元	699600	1310860	1490689	1725458	2067959
普通小学专任教师数	人	1821	1776	1762	1732	1723
普通小学在校学生数	人	41289	40173	39959	39906	39067
普通中学专任教师数	人	1876	2527	2506	2604	2713
普通中学在校学生数	人	35792	45411	45986	47458	48357
卫生机构床位数	张	4767	5121	5013	4790	4937
卫生技术人员	人	6123	5970	5983	6538	6827
# 执业(助理)医师	人	2057	2311	2333	2613	2541
注册护师、护士	人	2091	2201	2295	2531	2683

?1994-2018 China Academic Journal Electronic Publishing House. All rights reserved.　http://www.cnki.net

社会主要指标

2009年	2010年	2011年	2012年	2013年	2014年	2015年	2016年
78.52	61.62	61.87	62.08	62.23	62.40	62.89	63.87
308.44	369.07	443.80	513.25	544.66	617.90	677.74	741.68
61.14	85.98	109.77	129.50	105.91	120.03	134.23	151.36
247.30	283.09	334.03	383.75	438.75	497.87	543.51	590.32
19.04	33.42	43.47	12.48	14.16	13.03		12.44
46146	58323	71876	82816	87629	99158	108188	117021
115.2	114.1	114.6	113.8	110.8	110.8	109.9	108.3
2153800	2790607	2911287	3764500	4642600	5434300	1999800	2075700
179401	216601	267590	330285	392335	432276	453335	450089
100956	133034	144602	179421	240507	282868	321992	358550
19424	22998	27025	31268	34520	37765	34924	37539
2414856	2854530	3425613	3950674	4451118	5058665	5614587	6105271
1764	1771	1872	1861	1895	1938	1992	2070
38526	38469	38958	39027	40391	41443	42754	43978
2739	2916	3013	3025	2898	2908	2956	3035
49724	50226	50391	50065	47283	48308	47955	49269
5374	5846	6250	6325	7202	7495	7486	8119
7674	8575	9883	10580	11105	12011	11946	12261
2863	3199	3340	3564	3691	3825	3814	2798
3223	3576	4159	4423	4671	4943	4921	4507

?1994-2018 China Academic Journal Electronic Publishing House. All rights reserved. http://www.cnki.net

4-4 莲湖区经济

指　　标	单　位	2000年	2005年	2006年	2007年	2008年
年底总人口	万人	57.48	73.08	73.97	74.36	74.56
生产总值	亿元	77.61	164.50	191.20	230.76	293.13
第一产业	亿元	0.18	0.19	0.18	0.19	
第二产业	亿元	37.30	79.68	91.35	109.90	140.60
第三产业	亿元	40.13	84.63	99.67	120.67	152.53
# 工业增加值	亿元	13.56	64.77	73.12	87.83	108.14
人均生产总值	元	13551	22789	26004	31115	39367
生产总值指数	上年=100	130.0	111.1	112.4	115.4	116.9
全社会固定资产投资	万元	20292	879653	1120045	1545102	2089500
地方财政收入	万元	21624	72619	83877	115475	154672
地方财政支出	万元	21580	46287	50866	79982	93628
农村居民人均纯收入	元	5027				
城镇居民人均可支配收入	元					15471
常用耕地面积	公顷	459				
粮食产量	吨	251	176	146	35	25
农林牧渔业总产值	万元	2534	2644	1900	2598	40
社会消费品零售总额	万元	644076	1163430	1287064	1445661	1696195
普通小学专任教师数	人	2209	2170	2135	2118	2103
普通小学在校学生数	人	45159	45066	45980	45685	45197
普通中学专任教师数	人	1897	2076	2076	2091	2088
普通中学在校学生数	人	27336	26693	27224	36787	36668
卫生机构床位数	张	3782	4094	3958	3602	3974
卫生技术人员	人	4668	4691	5888	4346	5493
# 执业(助理)医师	人		1947	2489	1615	2147
注册护师、护士	人		1623	2116	1541	2023

?1994-2018 China Academic Journal Electronic Publishing House. All rights reserved. http://www.cnki.net

社会主要指标

2009年	2010年	2011年	2012年	2013年	2014年	2015年	2016年
73.82	69.86	70.13	70.25	70.43	70.68	71.23	72.23
339.20	367.87	421.50	451.09	501.94	539.53	573.60	621.91
158.43	164.60	182.01	180.23	194.35	193.90	183.47	194.55
180.77	203.27	239.49	270.86	307.59	345.63	390.13	427.36
111.66	126.29	137.62	140.47	143.83	106.52		102.67
45218	51207	62459	68300	74876	76468	80840	86702
113.8	114	112.3	111.7	111.1	109.0	107.7	108.0
2680534	3479335	3764003	4734900	5838600	6088200	2831000	2251400
182161	223533	281296	347437	415439	471313	491623	481780
110360	152009	194774	235296	290525	339115	410484	437088
19375	22940	26962	31195	34450	37757	34820	37425
1981035	2348412	2820804	3259272	3686210	4130029	4518207	4917248
2149	2152	2136	2201	2196	2196	2228	2281
44349	43743	44424	45315	47213	49313	51522	53059
2130	2133	2095	2117	2118	2205	2233	2277
36635	35703	35064	35213	34602	34260	33074	32207
4610	4740	4994	5444	5972	6326	7350	7213
6745	7192	7883	8854	9313	9810	10856	11071
2711	2815	2813	3053	3321	3485	3763	2593
2782	3074	3479	3992	4176	4465	5108	4293

?1994-2018 China Academic Journal Electronic Publishing House. All rights reserved. http://www.cnki.net

4-5 灞桥区经济

指　　标	单　位	2000年	2005年	2006年	2007年	2008年
年底总人口	万人	43.11	53.20	54.23	54.78	55.26
生产总值	亿元	27.55	60.10	72.87	88.77	112.61
第一产业	亿元	2.73	4.61	5.28	6.29	7.91
第二产业	亿元	13.52	32.52	39.52	47.79	59.38
第三产业	亿元	11.30	22.97	28.07	34.69	45.32
# 工业增加值	亿元	12.63	28.10	34.23	41.78	52.00
人均生产总值	元	6415	11357	13509	16291	20367
生产总值指数	上年=100	109.8	113.2	115.1	116.0	117.6
全社会固定资产投资	万元	27995	280125	436214	602863	861335
地方财政收入	万元	9367	20309	24216	32921	50132
地方财政支出	万元	10463	23639	32042	44198	58402
农村居民人均纯收入	元	2841	3815	4250	4862	5844
城镇居民人均可支配收入	元					14195
常用耕地面积	公顷	15518	13400	12950	12468	12220
粮食产量	吨	75001	74557	73676	50954	72307
农林牧渔业总产值	万元	44607	72204	82963	99530	125150
社会消费品零售总额	万元	105500	178857	200641	227928	270076
普通小学专任教师数	人	2123	2104	2072	1954	2308
普通小学在校学生数	人	46900	34300	33600	32300	31700
普通中学专任教师数	人	1862	1953	1962	1804	1746
普通中学在校学生数	人	33200	39300	37300	28800	26700
卫生机构床位数	张	368	353	1431	1450	1593
卫生技术人员	人	429	390	1813	2082	1858
# 执业(助理)医师	人	188	175	856	1005	869
注册护师、护士	人	75	97	620	702	580

?1994-2018 China Academic Journal Electronic Publishing House. All rights reserved.　http://www.cnki.net

社会主要指标

2009年	2010年	2011年	2012年	2013年	2014年	2015年	2016年
57.25	59.56	59.87	60.16	60.50	60.82	61.39	62.73
144.95	168.37	204.58	238.80	288.05	309.73	312.87	329.78
8.79	11.50	14.41	16.08	16.86	18.14	18.74	19.73
80.52	89.53	106.11	118.25	140.10	141.61	124.25	117.98
55.64	67.34	84.06	104.47	131.09	149.98	169.88	192.07
64.22	83.91	102.89	113.79	123.82	101.24		57.41
25213	28269	34171	39694	47612	50926	51203	53139
115.0	115.4	116.1	114	114.9	111.1	108.8	109.6
1198689	1629018	1865078	2363600	3156900	4136848	5059200	5810600
70281	100245	131500	168539	208989	236711	254221	219910
73026	104972	144121	169283	190742	220305	295407	272233
7136	8849	11291	13278	15203	16982	18891	20431
17843	21162	24817	28688	31952	35147	34236	36784
11824	11374	10700	10511	9840	9241		5242
72016	72827	57059	61082	56370	52853		52567
136095	178275	219746	245553	279503	300728		327061
362680	501825	680230	954303	1322583	1542793	1732664	2008157
1813	1732	1677	1665	1631	1604	1702	1923
30700	32268	33768	34553	36658	39474	43391	47558
1718	1691	1619	1658	1701	1803	1864	2075
25300	24139	22891	22266	22781	23571	23355	23304
1658	2373	2157	2094	2621	2944	3059	2825
1969	3301	3773	3962	4129	4130	4212	4056
835	1485	1472	1485	1447	1426	1430	799
765	1182	1403	1571	1676	1689	1721	1232

?1994-2018 China Academic Journal Electronic Publishing House. All rights reserved.   http://www.cnki.net

4-6 未央区经济

指　　标	单　位	2000年	2005年	2006年	2007年	2008年
年底总人口	万人	37.97	54.54	56.29	59.00	61.30
生产总值	亿元	62.54	146.90	176.28	217.19	281.85
第一产业	亿元	2.20	1.82	1.89	1.74	2.16
第二产业	亿元	35.05	76.99	91.48	112.88	148.18
第三产业	亿元	25.29	68.09	82.91	102.57	131.51
# 工业增加值	亿元		65.97	76.87	89.75	107.39
人均生产总值	元	16635	21737	25228	30015	37547
生产总值指数	上年=100	114.8	118.3	115.3	118.0	118.9
全社会固定资产投资	万元	101700	1030300	1330700	1751600	2404200
地方财政收入	万元	13919	30831	37358	62928	84491
地方财政支出	万元	14614	36525	49178	60393	86329
农村居民人均纯收入	元	3332	4389	4800	5421	6441
城镇居民人均可支配收入	元					14883
常用耕地面积	公顷	10156	5379	5089	5304	4310
粮食产量	吨	55826	27040	25637	27367	28364
农林牧渔业总产值	万元	31403	23293	21737	31118	30611
社会消费品零售总额	万元	256000	643788	852064	1145296	1641442
普通小学专任教师数	人	2274	2276	2353	2257	2512
普通小学在校学生数	人	40500	41100	44200	47400	49400
普通中学专任教师数	人	1792	1970	1983	2459	2276
普通中学在校学生数	人	28600	28000	32000	33300	37700
卫生机构床位数	张	1403	1955	2171	2030	2243
卫生技术人员	人	2425	1874	2100	2632	2958
# 执业(助理)医师	人	771	770	880	958	1148
注册护师、护士	人	738	748	842	1042	1129

?1994-2018 China Academic Journal Electronic Publishing House. All rights reserved. http://www.cnki.net

社会主要指标

2009年	2010年	2011年	2012年	2013年	2014年	2015年	2016年
62.96	80.72	81.14	81.46	81.84	82.28	83.05	85.08
332.93	397.43	459.54	509.58	607.40	703.14	720.22	772.88
2.42	2.48	2.31	2.24	1.41	1.06	1.34	1.06
172.39	211.37	232.12	251.68	310.80	371.00	357.72	379.68
158.12	183.58	225.11	255.66	295.19	331.08	361.16	392.14
130.20	156.63	174.79	189.88	232.36	266.42		239.61
42956	49894	56782	62679	73619	85686	87126	91938
115.5	115.1	113.0	109.6	111.9	110.1	107.0	108.7
3233600	4385921	4498254	5689900	7234200	9155090	9270500	9836400
108301	144600	174578	250009	313703	355092	378000	326330
98167	128260	143641	184981	207918	239836	282397	318025
7826	9712	12383	14562	16455	18364	19824	21294
18721	22184	26041	30103	33268	36462	34525	37085
4149	3473	3180	1468	1386	1047	833	404
28523	26776	20508	15695	8012	3997	1968	800
39860	40781	37534	36525	25320	19090	25786	20405
2005426	2454065	3010965	3617547	4237165	4807887	5152112	5861678
2537	2533	1963	1918	2027	1714	2231	2731
51100	53362	43524	46106	51232	58329	65326	74218
2255	2265	1933	1926	2050	2575	2933	3132
33100	33518	28763	28799	30423	30727	31440	32396
2335	2585	2718	3148	3330	3573	4873	4948
3153	3938	4117	4435	4984	5701	7728	8267
1151	1480	1470	1525	1709	1811	2414	1609
1324	1592	1640	1771	2045	2193	3125	2626

?1994-2018 China Academic Journal Electronic Publishing House. All rights reserved. http://www.cnki.net

4-7 雁塔区经济

指 标	单 位	2000年	2005年	2006年	2007年	2008年
年底总人口	万人	55.78	107.74	110.33	111.63	112.27
生产总值	亿元	109.82	231.02	278.52	339.96	432.87
第一产业	亿元	1.46	1.57	1.68	1.75	2.12
第二产业	亿元	45.95	92.69	116.06	140.03	174.13
第三产业	亿元	62.41	136.76	160.78	198.18	256.62
# 工业增加值	亿元	36.08	60.18	75.71	91.17	108.42
人均生产总值	元	20535	22310	25543	30633	38666
生产总值指数	上年=100	113.0	116.1	117.0	118.2	118.1
全社会固定资产投资	万元	23820	2452340	3295000	4219382	5155100
地方财政收入	万元	16908	53818	68018	85606	118696
地方财政支出	万元	16019	43631	49185	69138	84115
农村居民人均纯收入	元	3365	4658	5020	5604	6585
城镇居民人均可支配收入	元					15695
常用耕地面积	公顷	6296	2775	2665	2454	1953
粮食产量	吨	29301	14506	13181	8616	5930
农林牧渔业总产值	万元	25916	23204	23946	25959	31584
社会消费品零售总额	万元	524500	1048738	1315291	1671787	2259537
普通小学专任教师数	人	2391	2482	2573	2672	2844
普通小学在校学生数	人	49200	55300	59700	61700	64600
普通中学专任教师数	人	2393	2717	2817	2827	2869
普通中学在校学生数	人	35000	43500	45400	45300	45500
卫生机构床位数	张	1605	6103	6274	6166	7128
卫生技术人员	人	2220	5706	7355	6963	9037
# 执业(助理)医师	人		2243	2930	2741	3298
注册护师、护士	人					

?1994-2018 China Academic Journal Electronic Publishing House. All rights reserved. http://www.cnki.net

社会主要指标

2009年	2010年	2011年	2012年	2013年	2014年	2015年	2016年
113.67	117.98	118.48	118.89	119.29	119.74	120.96	123.11
498.58	636.46	765.99	848.00	988.20	1114.46	1165.11	1235.43
2.19	2.13	2.61	2.34	1.93	1.62	0.14	
180.68	211.23	256.40	282.69	346.45	389.82	365.07	338.05
315.71	423.10	506.98	562.97	639.82	723.02	799.90	897.38
93.99	105.00	137.95	183.97	212.06	245.07		183.33
44134	54948	64788	71447	82979	93245	96810	101236
114.4	113.5	113.7	111.5	110.1	110.7	107.8	108.0
6518640	8101966	7677355	9560600	10292400	9696465	8121500	8927900
155260	203716	264486	344958	414186	470617	501690	465070
101301	130818	164006	208047	273763	326731	347450	351053
7948	9863	12585	14800	16724			
19762	23517	27601	31934	35341	38345	35071	37631
1692	1347	1056	564	434	434		
1762	565	337					
31094	30143	36907	32760	31763	26798	3205	
2723539	3279400	4016162	4728522	5343029	5964957	6564690	7143663
2926	2954	3204	3287	3436	3408	3680	4079
65700	66433	68354	70405	73857	78732	86177	92831
2915	3017	3092	3193	3174	3954	4124	4248
46900	48097	48630	48623	47934	48056	48347	48881
7397	7490	7542	8171	8508	9245	8726	8885
10002	10271	11076	12123	12347	13560	14353	15300
3551	3606	3488	3934	3947	4268	4647	3655
	4433	4921	5436	5628	6299	6509	5953

?1994-2018 China Academic Journal Electronic Publishing House. All rights reserved.   http://www.cnki.net

4-8 阎良区经济

指　　标	单　位	2000年	2005年	2006年	2007年	2008年
年底总人口	万人	23.74	24.49	24.91	25.28	25.76
生产总值	亿元	22.01	41.64	49.30	58.51	70.90
第一产业	亿元	3.68	6.31	7.03	8.31	10.34
第二产业	亿元	11.49	21.87	26.26	31.34	37.94
第三产业	亿元	6.84	13.46	16.01	18.86	22.62
# 工业增加值	亿元	9.09	19.21	23.01	27.55	32.84
人均生产总值	元	9358	17150	19960	23311	27782
生产总值指数	上年=100	109.0	112.6	113.2	113.0	112.9
全社会固定资产投资	万元	42249	222143	314672	443678	652800
地方财政收入	万元	8875	14943	18533	23558	31042
地方财政支出	万元	9565	20942	29721	38190	49003
农村居民人均纯收入	元	2471	3806	4251	4928	5939
城镇居民人均可支配收入	元					15534
常用耕地面积	公顷	17094	16387	16348	16159	16087
粮食产量	吨	116359	103724	101912	115624	96400
农林牧渔业总产值	万元	53373	95360	106590	125740	156360
社会消费品零售总额	万元	70126	82536	95850	112937	138550
普通小学专任教师数	人	934	1012	1056	1041	1042
普通小学在校学生数	人	28860	18524	17436	16032	14995
普通中学专任教师数	人	1021	1148	1153	1123	1069
普通中学在校学生数	人	17351	22569	21813	19447	17691
卫生机构床位数	张	1143	1165	1268	1190	1278
卫生技术人员	人	1062	1176	1348	1397	1356
# 执业(助理)医师	人	354	372	389	397	456
注册护师、护士	人	417	408	435	435	517

?1994-2018 China Academic Journal Electronic Publishing House. All rights reserved.　http://www.cnki.net

社会主要指标

2009年	2010年	2011年	2012年	2013年	2014年	2015年	2016年
26.27	27.87	28.01	28.23	28.40	28.53	28.84	29.08
82.32	99.38	116.37	137.64	164.88	184.25	188.72	193.94
11.29	14.26	17.61	19.70	20.37	22.21	22.37	22.56
43.50	51.16	58.94	74.31	94.18	105.61	102.72	102.39
27.53	33.96	39.82	43.63	50.33	56.43	63.63	68.99
32.79	41.56	48.82	62.42	80.62	89.19		91.33
31637	36712	41650	48947	58220	64717	65791	66968
112.9	115.3	113.5	112.3	111.5	109.9	105.7	102.3
885243	1178258	1209231	1545200	2011600	2612321	2872100	1583500
39024	49530	65023	80905	102426	120355	128342	127552
66758	90044	109707	139962	166351	186987	220632	230682
7233	8969	11426	13403	15212	17007	18850	20262
19512	22927	26839	31026	34375	37503	34382	36931
16009	15843	15795	15792	15696	15632	15478	15457
95975	96027	81075	81318	85369	81003	88211	86790
169394	214232	259395	290460	324413	354330	368854	381618
164091	196666	239115	278683	313725	349333	379026	412174
1047	1026	1042	822	908	916	888	894
14285	13774	13552	13442	13439	13780	14401	14972
1075	1005	1011	921	951	974	945	971
15921	14713	13765	12824	12191	11507	10830	10286
1221	1323	1265	1402	1401	1418	1422	1438
1377	1520	1559	1864	1960	1931	2007	2133
451	534	516	603	631	643	683	490
531	611	659	805	847	824	855	729

?1994-2018 China Academic Journal Electronic Publishing House. All rights reserved. http://www.cnki.net

4-9 临潼区经济

指　　标	单　位	2000年	2005年	2006年	2007年	2008年
年底总人口	万人	67.39	65.82	66.60	67.29	67.90
生产总值	亿元	25.00	60.72	70.35	84.25	104.92
第一产业	亿元	8.34	12.52	12.64	15.18	19.74
第二产业	亿元	7.08	28.97	35.25	42.60	52.36
第三产业	亿元	9.58	19.23	22.46	26.47	32.82
# 工业增加值	亿元	8.47	26.32	32.01	39.08	48.05
人均生产总值	元	3710	9223	10625	12585	15522
生产总值指数	上年=100	109.6	112.7	113.0	112.7	114.6
全社会固定资产投资	万元	37516	241037	317477	440753	612234
地方财政收入	万元	12918	16613	20328	25627	30761
地方财政支出	万元	17414	34555	48264	61673	78968
农村居民人均纯收入	元	2428	3172	3534	4020	4828
城镇居民人均可支配收入	元					12489
常用耕地面积	公顷	52598	50522	50144	49911	49802
粮食产量	吨	353592	340412	370835	351489	375202
农林牧渔业总产值	万元	130362	191633	201723	240467	313700
社会消费品零售总额	万元	119500	203743	224520	250354	290155
普通小学专任教师数	人	3080	3111	3253	3394	3418
普通小学在校学生数	人	91200	63500	60300	56100	51900
普通中学专任教师数	人	2572	3109	2940	3015	3010
普通中学在校学生数	人	47700	57800	54400	52200	50000
卫生机构床位数	张	1070	1130	1130	1315	1345
卫生技术人员	人	1390	1590	1625	1688	1718
# 执业(助理)医师	人	396	517	552	613	665
注册护师、护士	人	321	382	413	479	503

?1994-2018 China Academic Journal Electronic Publishing House. All rights reserved. http://www.cnki.net

社会主要指标

2009年	2010年	2011年	2012年	2013年	2014年	2015年	2016年
68.62	65.60	65.98	66.45	66.81	67.16	67.62	68.18
113.01	149.08	181.54	208.07	221.58	221.78	193.10	183.11
20.72	25.58	28.27	29.63	29.89	31.16	30.27	30.59
54.88	79.79	102.03	120.48	124.06	116.26	81.20	59.25
37.41	43.71	51.24	57.96	67.63	74.36	81.63	93.27
58.06	72.12	93.33	109.91	114.58	101.65		46.53
16556	22214	27594	31426	33255	33109	28653	26968
113.5	113.6	112.9	111.8	111.5	105.0	105.6	104.2
822243	1084539	1178946	1522000	1969400	2315638	1863100	1417500
35086	45908	62604	80065	102977	121441	137407	149128
108894	154060	194348	242058	272237	293966	392195	384236
5794	7156	9109	10685	12160	13595	15164	16389
15527	18213	21271	24568	27318	29804	28628	30753
49586	49330	47732	46994	46873	46492	46246	45926
385592	400971	320266	351676	326809	317870	328636	316894
333229	411121	447588	468170	515993	538221	551556	569391
342913	409523	497232	577527	640802	726349	779895	846604
3350	3212	3111	3013	2966	2773	2531	2578
46500	43329	40634	37899	36867	36776	36807	37673
3018	2903	2908	2899	2899	2830	2769	2764
46400	43878	40950	37962	35356	33021	31225	29645
1445	1439	1756	1911	2221	2309	2331	2524
1749	2023	2336	2287	2454	2578	2648	2816
720	836	866	834	869	876	898	441
540	621	817	810	905	954	988	655

?1994-2018 China Academic Journal Electronic Publishing House. All rights reserved. http://www.cnki.net

4-10 长安区经济

指　　标	单　位	2000年	2005年	2006年	2007年	2008年
年底总人口	万人	89.64	99.16	100.73	101.36	102.00
生产总值	亿元	31.06	73.16	86.45	112.91	148.69
第一产业	亿元	6.75	12.21	13.13	14.28	17.59
第二产业	亿元	15.28	33.74	41.51	60.61	81.55
第三产业	亿元	9.03	27.21	31.81	38.02	49.55
# 工业增加值	亿元	13.73	25.07	33.74	45.00	60.74
人均生产总值	元	3487	7513	8650	11174	14623
生产总值指数	上年=100	114.8	113.8	115.0	114.4	117.5
全社会固定资产投资	万元	46300	628257	868400	1254002	1699800
地方财政收入	万元	10055	27875	33550	44828	67684
地方财政支出	万元	17034	50045	61291	84980	113192
农村居民人均纯收入	元	2315	3331	3592	4143	4926
城镇居民人均可支配收入	元					13108
常用耕地面积	公顷	53114	47083	46874	46780	46723
粮食产量	吨	361843	387323	398964	347450	400973
农林牧渔业总产值	万元	119642	183638	199588	230454	283643
社会消费品零售总额	万元	182000	373627	441272	524437	646673
普通小学专任教师数	人	3368	4012	4207	3809	3693
普通小学在校学生数	人	109500	69700	65000	60700	56700
普通中学专任教师数	人	3259	4741	4639	3889	3849
普通中学在校学生数	人	64700	66600	68000	65500	61300
卫生机构床位数	张	2565	2084	2169	2013	2002
卫生技术人员	人	2315	2000	2157	2660	2874
# 执业(助理)医师	人	1916	734	785	1001	1122
注册护师、护士	人	644	533	660	773	865

?1994-2018 China Academic Journal Electronic Publishing House. All rights reserved.　http://www.cnki.net

社会主要指标

2009年	2010年	2011年	2012年	2013年	2014年	2015年	2016年
103.37	108.48	109.01	109.54	110.03	110.59	111.83	114.11
225.70	261.53	299.20	328.79	366.82	445.43	513.88	608.02
19.20	24.50	28.97	30.06	31.76	33.67	36.53	38.47
110.60	130.69	141.93	152.36	157.81	217.23	261.90	332.60
95.90	106.34	128.30	146.37	177.25	194.53	215.45	236.95
88.49	110.92	128.47	122.60	128.70	174.18		284.55
21980	24708	27533	30088	33413	40380	46208	53821
114.8	114.7	113.2	114.3	111.2	111.3	114.3	112.0
2264100	3022567	3143523	4069900	5415300	6782191	7265300	7380200
95124	132207	180176	247228	311607	354267	377767	354999
157795	244637	304227	365362	447642	494189	534331	612604
5965	7389	9421	11107	12695	14206	15486	16741
16490	19557	22918	26493	29460	32377	32204	34627
46502	46265	45757	44219	43579	41980	41428	39355
415742	414922	346214	368814	357678	338400	345885	332415
304864	382196	438545	453711	509105	538210	584120	621269
768058	918642	1114116	1300948	1449431	1640031	1833669	2024940
3658	3623	3178	3119	3122	3072	3093	3263
53300	52926	45552	46924	49233	52230	56023	59971
3852	4125	3680	3575	3535	3478	3493	3637
54800	56307	47739	44936	43142	41733	40725	40702
2335	2413	2772	3226	3349	3575	4953	5592
3395	3768	3690	4038	4526	4775	6083	7195
1223	1546	1537	1632	1690	1722	2032	1199
1007	1148	1247	1427	1650	1756	2470	2061

?1994-2018 China Academic Journal Electronic Publishing House. All rights reserved. http://www.cnki.net

4-11 高陵区经济

指　　标	单　位	2000年	2005年	2006年	2007年	2008年
年底总人口	万人	23.30	24.49	26.35	27.00	28.63
生产总值	亿元	10.38	31.42	41.17	60.28	88.53
第一产业	亿元	3.35	5.65	5.13	6.85	8.87
第二产业	亿元	4.69	19.49	28.90	44.54	67.12
第三产业	亿元	2.34	6.28	7.14	8.89	12.54
# 工业增加值	亿元	3.47	15.55	25.48	40.39	60.79
人均生产总值	元	4477	13174	18021	25012	31828
生产总值指数	上年=100	114.1	123.6	127.6	135.1	135.6
全社会固定资产投资	万元	37791	348900	510200	690269	1002000
地方财政收入	万元	3547	10443	14604	21177	30874
地方财政支出	万元	7291	22265	30379	41964	54690
农村居民人均纯收入	元	2219	3120	3436	3914	4708
城镇居民人均可支配收入	元				9990	11890
常用耕地面积	公顷	16431	15156	15211	15247	15876
粮食产量	吨	211113	187298	188352	187068	207578
农林牧渔业总产值	万元	64165	96876	98162	114968	148577
社会消费品零售总额	万元	25200	45600	53600	64300	94776
普通小学专任教师数	人	1114	1195	1064	1218	1251
普通小学在校学生数	人	31213	19004	18828	16204	15014
普通中学专任教师数	人	1128	1423	1190	1149	1138
普通中学在校学生数	人	20469	21816	20566	19459	18696
卫生机构床位数	张	329	492	495	457	770
卫生技术人员	人	580	649	725	720	896
# 执业(助理)医师	人	281	217	267	247	293
注册护师、护士	人	124	179	211	230	220

?1994-2018 China Academic Journal Electronic Publishing House. All rights reserved.　http://www.cnki.net

社会主要指标

2009年	2010年	2011年	2012年	2013年	2014年	2015年	2016年
30.80	33.35	33.53	33.81	34.01	34.22	34.77	35.11
107.76	138.46	184.25	238.69	283.23	298.27	297.19	300.24
9.20	12.12	16.93	20.33	22.12	25.96	27.70	30.23
82.45	106.03	140.71	184.37	213.60	219.67	215.02	205.14
16.11	20.31	26.61	33.99	47.51	52.64	54.47	64.87
86.85	110.79	138.20	167.50	209.38	199.92		184.98
36265	43168	55099	70891	83524	87431	86154	85930
126.8	124.2	119.4	118.3	117.8	112.0	102.7	107.3
1343700	1744059	1776472	2358700	3147100	4158300	4320800	4595300
40138	52011	70031	95341	115071	132510	141334	120185
80088	100412	123775	162754	184052	202716	255372	232310
5735	7106	9053	10673	12167	13615	15191	16431
14827	17377	20313	23462	26030	28581	27423	29464
15919	15406	15254	15244	15197	15126	15076	37467
210622	208999	200313	200246	198221	190373	192757	297777
155044	204388	279451	334939	399495	468141	495452	555928
115084	140900	175338	224251	250091	283978	317265	445415
1325	1361	1359	1352	1324	1308	1311	1409
14099	13743	13767	13958	14644	15992	17429	18973
1140	1104	1058	1026	1008	982	984	904
17601	14228	13535	12439	11457	10890	10636	10412
822	856	1034	1227	1260	1436	1632	1702
976	1132	1231	1613	1765	2114	2322	2676
320	394	392	475	531	588	720	525
343	388	423	547	636	798	921	749

?1994-2018 China Academic Journal Electronic Publishing House. All rights reserved. http://www.cnki.net

4-12 蓝田县经济

指　　标	单　位	2000年	2005年	2006年	2007年	2008年
年底总人口	万人	62.93	50.84	51.40	52.00	52.00
生产总值	亿元	16.61	29.54	33.44	39.59	49.68
第一产业	亿元	4.63	7.59	8.21	9.71	12.17
第二产业	亿元	4.95	9.07	11.23	13.34	16.83
第三产业	亿元	7.03	12.88	14.00	16.54	20.68
# 工业增加值	亿元	3.56	6.38	7.93	9.34	11.35
人均生产总值	元	2639	5829	6559	7678	9554
生产总值指数	上年=100	110.5	110.1	119.5	112.1	113.1
全社会固定资产投资	万元	19800	144344	189471	265320	377293
地方财政收入	万元	5003	5168	5368	6234	7964
地方财政支出	万元	11583	22351	28467	45315	76145
农村居民人均纯收入	元	1699	2430	2663	3121	3611
城镇居民人均可支配收入	元					10202
常用耕地面积	公顷	45523	41069	41061	41059	41059
粮食产量	吨	250618	291305	312477	250267	324830
农林牧渔业总产值	万元	88560	132851	144070	170251	213481
社会消费品零售总额	万元	76600	135283	154121	178254	214450
普通小学专任教师数	人	2909	3136	3128	3185	3042
普通小学在校学生数	人	83448	62866	61600	57502	52325
普通中学专任教师数	人	1799	2460	2397	2431	2447
普通中学在校学生数	人	37432	44827	46212	45682	44660
卫生机构床位数	张	733	852	855	716	779
卫生技术人员	人	938	1024	1036	1056	1216
# 执业(助理)医师	人		486	508	450	524
注册护师、护士	人		243	264	278	328

?1994-2018 China Academic Journal Electronic Publishing House. All rights reserved. http://www.cnki.net

社会主要指标

2009年	2010年	2011年	2012年	2013年	2014年	2015年	2016年
52.89	51.42	51.65	51.88	52.07	52.30	52.53	52.86
57.32	67.53	78.97	92.83	107.81	109.57	112.10	122.44
13.03	16.64	20.49	24.45	24.45	25.90	26.00	27.59
20.50	23.84	26.39	31.81	39.99	35.33	32.89	34.87
23.79	27.05	32.09	36.57	43.37	48.34	53.21	59.98
12.39	17.44	18.35	21.73	26.56	18.67		18.71
10931	12768	15325	17931	20566	20996	21388	23236
112.1	112.5	111.9	111.6	112.0	108.8	106.3	108.2
515382	682881	770932	995400	1273800	1642785	1966800	2643700
10587	15401	21224	26721	32724	38387	42564	42872
96725	132634	155355	192513	234919	251369	301002	356183
4315	5316	6704	7824	8833	9911	11084	12082
12713	14874	17309	19957	21953	23907	24509	26321
40982	40667	40533	40400	39879	39733	39680	14993
331133	335325	265829	289759	264609	250600	260422	187779
223567	286829	337450	401514	436071	462420	465601	492519
250060	297044	356378	411754	457356	515669	563996	620818
2923	2958	2843	2836	2737	2594	2477	2472
46758	43697	41044	35010	32257	30842	29041	28411
2499	2525	2628	2626	2640	2612	2612	2546
43770	42650	40860	37088	35122	32195	30258	28451
842	1179	1225	1005	1127	1142	1256	1525
1231	1377	1356	1494	1565	1550	1900	2291
503	566	542	574	585	559	654	281
315	340	351	424	432	447	598	444

?1994-2018 China Academic Journal Electronic Publishing House. All rights reserved. http://www.cnki.net

4-13 周至县经济

指标	单位	2000年	2005年	2006年	2007年	2008年
年底总人口	万人	62.38	53.56	53.97	54.60	54.78
生产总值	亿元	16.00	24.25	27.56	32.51	40.07
第一产业	亿元	5.11	6.52	7.16	8.50	10.65
第二产业	亿元	3.56	6.97	8.42	9.59	11.22
第三产业	亿元	7.33	10.76	11.98	14.42	18.20
# 工业增加值	亿元	2.69	5.52	6.59	7.58	8.71
人均生产总值	元	2516	4146	5126	5989	7327
生产总值指数	上年=100	109.7	110.9	111.3	111.5	112.3
全社会固定资产投资	万元	47800	139980	198247	271122	377718
地方财政收入	万元	5685	3457	3967	4186	4938
地方财政支出	万元	12309	23170	31638	49277	69157
农村居民人均纯收入	元	1753	2330	2601	3059	3537
城镇居民人均可支配收入	元					10229
常用耕地面积	公顷	40536	34938	33582	33182	33536
粮食产量	吨	262898	290799	288934	251091	281556
农林牧渔业总产值	万元	91468	105979	116742	139686	175186
社会消费品零售总额	万元	53906	85411	99362	116852	143770
普通小学专任教师数	人	2980	2752	2677	2672	2649
普通小学在校学生数	人	91300	64000	57700	50400	45200
普通中学专任教师数	人	2340	2849	2923	2985	2994
普通中学在校学生数	人	52100	45200	63500	61500	57500
卫生机构床位数	张	548	651	797	678	678
卫生技术人员	人	1009	1070	1367	1486	1474
# 执业(助理)医师	人	330	356	437	504	544
注册护师、护士	人	180	200	218	295	285

?1994-2018 China Academic Journal Electronic Publishing House. All rights reserved. http://www.cnki.net

社会主要指标

2009年	2010年	2011年	2012年	2013年	2014年	2015年	2016年
55.72	56.29	56.59	57.00	57.24	57.57	58.09	58.50
44.90	54.37	66.14	78.34	89.98	98.84	104.33	114.99
11.11	15.27	20.30	25.39	26.34	27.62	29.54	33.17
12.15	14.30	15.91	19.40	23.44	26.09	25.01	26.08
21.64	24.80	29.93	33.55	40.20	45.13	49.78	55.74
9.09	10.62	13.42	16.04	18.30	17.98		17.01
8127	9758	11719	13795	15347	16932	18041	19726
111.4	109.6	112	111.7	109.2	108.8	109.3	108.2
506898	672797	684630	891400	1153000	1488047	1689500	1599900
6038	10169	15510	21001	28567	35218	39260	36522
98075	142652	181790	252265	248567	284284	341695	380428
4248	5328	6615	7733	8870	9961	11148	12207
12715	14877	17353	20025	22243	24445	25070	26899
33518	33526	33195	33257	33263	33269	32957	39473
282258	288582	226032	243158	230702	224955	231716	255720
185796	262734	329966	412820	460093	482477	508904	572627
167496	198592	238445	276827	307116	346273	384668	506357
2600	2509	2517	2661	2477	2305	2078	2138
41500	39446	38047	36191	35063	32235	32768	33169
2973	2926	2911	2915	2761	2658	2514	2541
52900	48078	44269	40449	37154	34076	31761	29859
668	779	1026	1235	1276	1398	1517	1470
1547	1778	1837	2130	2543	2617	2637	2672
509	657	542	637	657	681	658	412
336	407	488	602	692	826	849	676

?1994-2018 China Academic Journal Electronic Publishing House. All rights reserved. http://www.cnki.net

4-14 户县经济

指　　标	单　位	2000年	2005年	2006年	2007年	2008年
年底总人口	万人	56.30	54.36	54.91	55.56	55.88
生产总值	亿元	26.89	51.03	59.56	70.19	83.77
第一产业	亿元	5.25	8.03	8.40	9.72	11.90
第二产业	亿元	14.10	28.73	34.67	40.90	46.88
第三产业	亿元	7.54	14.27	16.49	19.57	24.99
# 工业增加值	亿元	12.85	25.53	30.59	35.99	40.27
人均生产总值	元	4789	9099	10901	12708	15034
生产总值指数	上年=100	106.9	111.8	112.6	112.1	110.8
全社会固定资产投资	万元	26920	261077	354483	491985	642068
地方财政收入	万元	9630	10341	13923	17417	21370
地方财政支出	万元	13124	27053	37755	47987	72067
农村居民人均纯收入	元	2009	3018	3325	3833	4408
城镇居民人均可支配收入	元	5338	7915	8685	9917	11496
常用耕地面积	公顷	36680	38702	38647	38620	38609
粮食产量	吨	300973	337469	353805	300442	350349
农林牧渔业总产值	万元	85502	130717	137060	160639	196606
社会消费品零售总额	万元	80892	141828	163696	191186	232147
普通小学专任教师数	人	2658	2638	2568	2537	2453
普通小学在校学生数	人	68300	46200	43400	40200	38100
普通中学专任教师数	人	2377	2959	3000	2969	2958
普通中学在校学生数	人	47200	52500	51600	63400	45800
卫生机构床位数	张	1275	1526	1523	1640	1712
卫生技术人员	人	1563	1662	1684	1619	2119
# 执业(助理)医师	人	547	760	812	827	839
注册护师、护士	人	224	489	468	486	660

?1994-2018 China Academic Journal Electronic Publishing House. All rights reserved.   http://www.cnki.net

社会主要指标

2009年	2010年	2011年	2012年	2013年	2014年	2015年	2016年
56.77	55.65	55.85	56.10	56.32	56.60	57.03	57.44
93.62	104.44	120.00	132.67	146.32	155.92	155.41	162.81
12.44	15.59	21.24	25.37	25.32	27.20	27.57	28.59
52.32	56.50	60.43	64.26	70.15	71.56	64.60	63.80
28.86	32.35	38.33	43.04	50.85	57.16	63.24	70.42
42.02	50.61	61.81	65.20	69.90	58.31		49.62
16623	18580	21525	23702	26031	27616	27353	28446
111.9	112	110.6	109.5	108.2	107.8	107.3	108.5
841986	1096427	1098101	1403300	1424100	1526925	1507700	1254400
26083	33948	44754	55773	70107	82748	91442	94138
92851	132347	177764	213234	236117	269178	338906	402850
5307	6549	8265	9654	10899	12218	13616	14638
14313	16761	19532	22520	24817	27026	26043	27970
38410	38313	38196	38160	38003	37535	37521	32881
357851	371523	302724	313700	303386	296007	304943	222594
208089	260295	340026	407108	447149	481686	496155	515503
273150	326333	393798	454857	509672	575164	637125	699849
2413	2329	2246	2145	2033	2010	1921	1966
36400	34785	33953	32326	30762	29944	29871	30582
2925	2915	2871	2805	2818	2724	2649	2668
42700	39730	37939	35574	33781	31532	29830	27509
1849	1962	2035	2414	2612	2780	3139	3026
2320	2488	2610	2794	3195	3454	3877	4177
880	1011	967	999	1057	1066	1173	788
820	850	907	1020	1187	1265	1576	1317

?1994-2018 China Academic Journal Electronic Publishing House. All rights reserved.   http://www.cnki.net
