# Supplementary material for: A study on the tourism efficiency of tourism destination based on DEA model: A case of ten cities in Shaanxi province
Source: PLoS One. 2024 Jan 19;19(1):e0296660. doi: 10.1371/journal.pone.0296660 (PMC10798521; doi:10.1371/journal.pone.0296660)
Supplement: S1 File — (ZIP) [file pone.0296660.s001.zip › Supporting information/Statistical yearbook/Xianyang.caj]

七、咸阳市

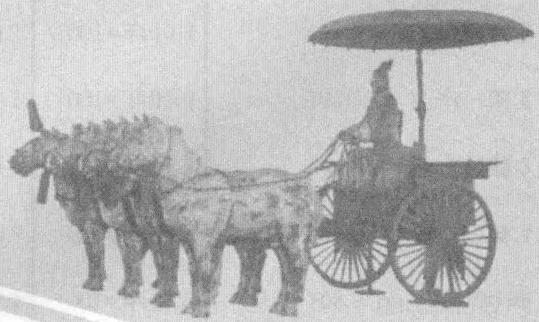

资料整理：向　静　马　艳

?1994-2018 China Academic Journal Electronic Publishing House. All rights reserved.　http://www.cnki.net

7-1 咸阳市经济

指　　标	单 位	2000年	2005年	2006年	2007年	2008年
年底总人口	万人	474.51	498.07	497.44	499.67	500.69
人口自然增长率	‰		4.49	4.47	4.46	4.50
年底总户数	万户	121.99	134.19	137.37	141.50	145.42
生产总值	亿元	234.46	432.49	483.87	588.48	764.55
第一产业	亿元	52.45	89.10	98.34	120.39	148.97
第二产业	亿元	102.33	191.99	220.41	271.39	382.65
第三产业	亿元	79.68	151.40	165.12	196.70	232.93
# 工业增加值	亿元	86.10	157.31	179.68	223.25	321.50
人均生产总值	元	4980	8683	9721	11804	15285
生产总值指数	上年=100	111.7	112.6	111.5	112.3	116.0
第一产业	上年=100	106.5	108.1	107.1	104.5	107.5
第二产业	上年=100	111.3	114.1	114.7	115.5	119.3
第三产业	上年=100	115.4	113.2	110.1	112.4	116.1
# 工业增加值	上年=100	110.6	114.3	114.6	115.6	121.3
人均生产总值指数	上年=100	107.8	110.1	111.6	112.1	115.6
非公有制经济增加值	亿元		191.52	219.17	268.14	360.98
文化产业增加值	亿元					
单位GDP能耗	吨标准煤/万元		1.380	1.330	1.264	1.201
单位GDP能耗比上年增长	%			-3.63	-4.97	-4.97
就业人员	万人	242.76	251.65	253.20	250.19	248.70
城镇单位就业人员	万人	40.33	36.55	36.59	36.76	37.32
# 国有单位	万人	32.05	27.64	26.76	26.86	26.76
集体单位	万人	2.40	2.17	2.15	2.04	1.97
# 在岗职工人数	万人	39.33	36.02	36.03	36.08	36.63
城镇单位就业人员平均工资	元					
城镇单位在岗职工平均工资	元	6893	11388	12897	17156	20383

?1994-2018 China Academic Journal Electronic Publishing House. All rights reserved.   http://www.cnki.net

社会主要指标

2009年	2010年	2011年	2012年	2013年	2014年	2015年	2016年
488.11	489.84	491.23	492.86	494.22	495.68	497.24	498.66
4.40	3.94	3.90	4.08	4.02	3.98	3.95	4.31
150.00	152.04	153.77	157.03	158.54	156.60	154.53	157.47
873.20	1098.68	1361.32	1573.68	1860.39	2085.15	2152.92	2390.97
157.41	203.29	252.46	283.10	299.56	321.72	328.78	345.32
434.02	573.27	740.40	876.78	1073.73	1227.70	1230.41	1385.00
281.77	322.12	368.46	413.80	487.10	535.73	593.73	660.65
356.42	480.70	624.28	743.94	912.14	1043.39	1027.67	1161.99
17434	22469	27751	31982	37695	42128	43365	48016
114.2	114.5	114.2	114.5	113.1	110.9	108.7	107.7
106.3	107.8	107.2	106.1	104.3	105.0	105.3	103.8
115.1	118.7	119.6	119.8	116.5	113.5	109.3	108.3
117.0	111.7	109.1	109.3	110.7	108.2	109.0	108.4
112.1	119.9	120.2	121.5	117.5	113.8	108.9	107.8
114.1	114.3	113.9	114.1	112.6	110.5	108.3	107.3
419.87	532.92	668.87	786.81	944.95	1072.42	1121.60	1267.00
					55.04	61.17	68.54
1.143	0.740	0.714	0.689	0.665	0.642	0.621	0.545
-4.81	-3.59	-3.61	-3.50	-3.50	-3.35	-3.39	-3.40
248.28	241.73	239.67	257.57	263.50	256.57	267.34	265.24
37.18	37.10	39.86	40.15	54.37	54.53	54.86	55.81
24.64	24.89	26.16	26.26	25.47	25.53	25.09	25.42
1.42	1.37	1.55	1.87	3.13	3.52	2.81	3.05
36.17	36.30	38.73	38.03	52.22	52.31	52.67	53.67
			37670	40753	42434	45309	48542
23935	27786	32763	38202	41215	42889	45850	49112

?1994-2018 China Academic Journal Electronic Publishing House. All rights reserved. http://www.cnki.net

7-1　续表 1

指　　标	单 位	2000年	2005年	2006年	2007年	2008年
全社会固定资产投资	亿元	73.29	202.19	245.20	394.17	569.75
# 房地产开发	亿元	3.36	23.27	27.04	35.13	49.41
商品房销售面积	万平方米	19.88	67.41	66.26	83.32	113.82
# 住宅	万平方米	19.47	63.08	66.04	81.30	112.89
地方财政收入	亿元	10.44	10.78	12.63	19.46	24.07
地方财政支出	亿元	16.37	30.38	41.37	60.64	85.01
金融机构人民币各项存款余额	亿元	243.80	471.29	544.25	590.46	733.65
金融机构人民币各项贷款余额	亿元	199.15	215.89	240.76	265.59	270.34
农村居民人均纯收入	元	1625	2268	2472	2914	3511
城镇居民人均可支配收入	元	5812	8780	9432	10970	13208
城市人均公园绿地面积	平方米			7.3	9.6	9.5
城市人均道路面积	平方米		8.5	9.4	10.6	10.0
城市用水普及率	%		96.6	96.1	97.2	99.0
城市燃气普及率	%		73.0	47.7	78.6	94.2
常用耕地面积	千公顷	407.87	369.00	358.52	360.00	357.90
农林牧渔业总产值	亿元	84.20	145.34	165.18	203.51	248.29
农作物总播种面积	千公顷	577.57	561.80	566.28	543.52	543.10
# 粮食作物	千公顷	478.17	448.87	450.31	428.41	425.95
粮食产量	万吨	183.90	199.14	209.18	180.71	204.94
棉花产量	吨	935	617	733	708	682
油料产量	吨	47552	44582	34699	27382	44188
蔬菜产量	吨	1416900	2424834	2595687	2485438	2865035

?1994-2018 China Academic Journal Electronic Publishing House. All rights reserved.　http://www.cnki.net

2009年	2010年	2011年	2012年	2013年	2014年	2015年	2016年
801.53	1050.54	1263.10	1616.47	2054.53	2492.43	3063.20	3643.74
73.40	93.84	118.48	151.76	174.83	195.95	181.82	178.86
129.40	164.51	215.73	139.83	203.89	218.58	225.46	204.75
125.62	159.34	213.38	136.78	201.77	216.16	215.50	189.88
31.67	43.48	58.56	69.17	79.00	85.46	85.44	81.50
107.17	151.40	190.19	234.94	265.19	272.44	300.19	338.32
936.00	1142.46	1334.61	1570.48	1811.67	1965.31	2251.78	2506.81
366.79	454.66	541.01	646.04	762.35	901.82	1028.65	1140.96
4206	5056	6401	7464	8538	8846	9690	10481
16404	18914	22224	25758	28488	27138	29425	31662
9.8	13.4	14.8	14.7	15.0	15.2	15.3	15.4
9.6	10.1	10.1	11.8	12.4	12.6	11.9	13.2
97.6	96.0	92.8	96.4	92.4	91.9	92.0	92.7
95.2	96.5	91.6	96.1	92.7	96.9	90.2	98.1
358.58	359.25	359.27	359.57	356.85	353.96	351.24	349.75
263.40	336.11	418.02	465.37	517.83	559.99	577.52	605.37
539.93	552.41	519.27	522.27	524.24	518.45	521.97	521.41
420.27	430.94	398.88	400.25	398.82	390.99	388.45	386.49
208.07	222.52	186.15	200.23	189.44	184.14	192.38	187.09
579	340	317	192	130	130	123	110
51166	51166	61836	46722	41580	47684	51821	50368
3155219	3384620	3467918	3683250	3872900	4118500	4346017	4430744

?1994-2018 China Academic Journal Electronic Publishing House. All rights reserved.   http://www.cnki.net

7-1　续表 2

指　　标	单 位	2000年	2005年	2006年	2007年	2008年
水果产量	吨	2041070	2653657	3126770	3130853	3883960
# 苹果	吨	1760700	2172571	2568877	2591959	3184291
肉类产量	吨	98363	152571	166864	119584	139010
# 猪牛羊肉	吨	86555	135313	150463	105689	121749
奶类产量	吨	156478	404128	429875	497376	531812
# 牛奶	吨	92552	333693	366060	426484	456776
禽蛋产量	吨	66764	81507	87931	67228	74600
水产品产量	吨	6416	8077	8418	2227	2360
规模以上工业企业单位数	个	336	361	406	432	441
规模以上工业总产值	亿元	208.70	359.16	458.52	565.61	860.24
纱产量	万吨	4.85	4.68	4.63	5.07	5.01
布产量	万米	23694	26525	26927	26689	27222
原煤产量	万吨	168.70	848.12	1188.45	1744.87	2212.38
发电量	亿千瓦小时	66.78	77.61	73.84	72.28	65.75
水泥产量	万吨	76.67	140.65	177.87	289.74	555.93
建筑业企业单位数	个	102	70	69	70	63
建筑业企业年末从业人员	万人	6.90	5.96	6.19	9.32	9.31
建筑业总产值	亿元	58.18	120.56	139.67	203.36	252.59
房屋建筑施工面积	万平方米	332.74	633.72	731.62	895.78	1153.31
房屋建筑竣工面积	万平方米	161.94	255.15	302.98	324.74	421.41
公路里程	公里	3722	4745	11345	13556	14294
# 等级公路	公里	3451	4681	8061	10819	12058
# 高速公路	公里	71	125	125	185	284
民用汽车拥有量	辆	28754	52609	131320	145044	140191
# 私人汽车	辆	9613	25603	100327	113324	101525
邮电业务总量	亿元	8.80	30.12	37.91	46.41	55.76
邮政业务总量	亿元	0.56	1.73	2.18	2.41	2.60
电信业务总量	亿元	8.25	28.39	35.73	44.00	53.16

?1994-2018 China Academic Journal Electronic Publishing House. All rights reserved.　http://www.cnki.net

2009年	2010年	2011年	2012年	2013年	2014年	2015年	2016年
4582138	4965124	5328370	5463202	5458140	5617342	5769646	5833716
3781018	4015329	4400670	4549358	4538669	4615995	4707111	4723312
156002	174616	193468	204921	213821	218664	213408	212491
139709	156521	168589	176812	183567	190901	185691	184105
606198	687387	736835	745987	790603	809917	703354	701866
536338	608599	644572	649080	685758	700515	597792	581151
86230	95839	104327	107266	112232	112063	115598	117753
2664	8633	8764	8001	8899	9905	9919	9902
628	671	553	639	681	774	862	952
1038.83	1401.92	1854.54	2293.52	2633.00	3002.05	3164.89	3541.50
7.77	10.10	11.00	10.42	15.00	16.70	20.66	14.90
29077	29267	26671	27116	24827	24826	23228	24197
2656.26	3310.38	4331.50	5085.14	5917.20	5496.15	5636.97	5910.90
64.13	121.08	170.80	174.71	189.37	186.75	180.66	177.51
759.51	901.76	1125.70	1132.03	1144.62	1303.97	1025.49	850.96
62	61	75	84	86	106	111	123
12.27	11.98	10.47	9.50	12.11	10.71	10.95	13.26
348.88	389.88	556.84	457.38	518.49	547.51	590.46	656.12
1258.32	1442.79	1880.69	2310.56	2724.54	3176.40	3262.60	3338.87
369.93	429.16	616.81	838.91	1109.41	1141.69	1284.83	1301.27
14976	15201	15327	15403	15407	15511	15732	15832
13253	13602	13761	13547	13549	13686	13999	14098
301	301	351	355	355	449	527	527
125420	156209	185481	217558	247124	270589	310627	358545
91066	116103	142466	173279	204096	229999	271133	321627
56.94	22.62	30.49	33.07	36.77	49.33	66.57	114.50
2.89	2.45	2.95	2.78	3.35	4.00	5.11	8.64
54.05	20.17	27.54	30.28	33.41	45.33	61.46	105.85

?1994-2018 China Academic Journal Electronic Publishing House. All rights reserved. http://www.cnki.net

7-1 续表 3

指 标	单 位	2000年	2005年	2006年	2007年	2008年
固定电话用户	万户	38.88	84.85	83.76	81.70	71.60
移动电话用户	万户	15.54	93.88	122.29	162.20	195.16
互联网宽带用户	万户	1.23	15.50	11.04	14.58	21.14
限额以上企业数	个					
批发业	个					
零售业	个					
住宿业	个					
餐饮业	个					
社会消费品零售总额	亿元	70.35	133.03	148.88	173.11	212.67
进出口总额	万美元		26183	29804	38257	37269
# 出口	万美元		18095	23938	30746	29458
实际外商直接投资额	万美元	1487	1954	4811	3476	3777
入境旅游人数	万人次	29.00	3.98	4.50	5.64	7.56
# 外国人	万人次	22.13	2.69	2.93	3.40	4.56
国际旅游外汇收入	万美元	1781	1632	1558	2800	3220
国内旅游人数	万人次	916	913	971	1026	1103
国内旅游收入	亿元	13.25	30.69	34.84	43.84	50.50
星级饭店数	个		26	26	27	27
幼儿园数	所	391	237	251	238	280
在园儿童数	万人	7.44	5.26	5.47	4.14	5.59
普通小学学校数	所	3300	2310	2104	1937	1787
普通小学专任教师数	人	26308	27899	27051	26974	26988
普通小学在校学生数	万人	66.89	50.70	49.10	45.31	42.12
普通中学学校数	所	324	354	343	334	334
普通中学专任教师数	人	17653	22109	22602	23143	24344
普通中学在校学生数	万人	33.63	44.91	46.24	45.80	44.60
卫生机构数	个	542	437	471	445	492
卫生机构床位数	张	10749	13393	14523	15790	17045
卫生技术人员	人	16888	16381	16461	17311	18624
# 执业(助理)医师	人	7548	7281	7253	7116	7125
注册护师、护士	人	4191	4816	4894	5287	6099

?1994-2018 China Academic Journal Electronic Publishing House. All rights reserved. http://www.cnki.net

2009年	2010年	2011年	2012年	2013年	2014年	2015年	2016年
66.08	61.93	62.75	63.73	62.63	61.67	54.01	46.37
219.71	252.54	299.15	334.48	337.72	367.88	373.66	398.34
23.30	29.50	36.97	42.41	46.56	50.57	56.50	80.50
240	303	353	434	544	596	669	780
17	24	30	37	41	50	49	56
118	153	177	220	300	337	398	490
33	38	40	59	63	67	61	62
72	88	106	118	140	142	161	172
250.59	296.35	345.10	401.08	462.68	528.84	601.59	688.55
26252	34045	46641	43299	60516	35.39(亿元)	28.64(亿元)	30.59(亿元)
20260	23849	36969	37359	47834	28.28(亿元)	15.22(亿元)	16.10(亿元)
4059	5188	6269	7080	7561	10356	13443	1995
10.53	14.00	24.06	32.70	11.80	12.90	13.60	14.10
6.35	11.00	15.96	22.42	8.10	5.03	9.10	5.80
1671	3342	722	2370	7363	9756	10361	4061
1246	1846	2480	3167	3988	4292	4787	5328
59.08	80.00	109.30	155.74	195.60	224.00	259.67	316.80
31	28	30	25	25	23	23	15
287	329	603	682	800	893	931	813
5.42	6.96	15.08	15.90	16.89	17.99	18.39	18.67
1634	1445	1372	1314	1238	1026	896	807
27323	27249	26980	25137	24069	22479	21303	20582
40.35	39.47	38.65	35.57	32.12	30.67	30.76	30.73
322	314	311	308	301	297	296	294
24760	24997	25384	25172	26652	26505	24401	25569
43.21	41.64	40.50	36.07	31.37	28.13	25.66	24.62
510	571	4649	4656	4683	4683	4715	4744
18272	18082	19764	21517	24232	27148	28458	29653
24041	26271	28963	31914	35729	37596	39044	42323
8274	7478	8114	8566	9290	9690	10219	10767
7885	8853	9741	11202	12567	14141	14713	16817

?1994-2018 China Academic Journal Electronic Publishing House. All rights reserved. http://www.cnki.net

7-2 秦都区经济

指 标	单 位	2000年	2005年	2006年	2007年	2008年
年底总人口	万人	41.45	49.42	48.89	49.08	49.38
生产总值	亿元		94.81	102.29	123.09	152.79
第一产业	亿元		6.40	6.77	7.98	9.40
第二产业	亿元		47.59	51.53	60.62	79.03
第三产业	亿元		40.82	43.99	54.49	64.36
# 工业增加值	亿元		41.19	42.68	50.16	65.90
人均生产总值	元		20067	20810	25128	31036
生产总值指数	上年=100		107.9	108.9	113.3	114.3
全社会固定资产投资	万元	258889	585932	740559	1059811	1538282
地方财政收入	万元	10997	14455	15427	20137	24279
地方财政支出	万元	11527	18876	20661	30404	46166
农村居民人均纯收入	元	2325	3135	3386	3738	4315
城镇居民人均可支配收入	元	5812	8780	9432	10970	14136
常用耕地面积	公顷	15902	12770	12736	12652	12641
粮食产量	吨	89776	83162	85488	73714	81543
农林牧渔业总产值	万元	75961	108060	112679	138302	159783
社会消费品零售总额	万元	98790	261719	295000	344000	414739
普通小学专任教师数	人	1786	1621	1670	1646	1638
普通小学在校学生数	人	42847	35025	34749	33200	31706
普通中学专任教师数	人	1571	1749	1742	1817	1897
普通中学在校学生数	人	28492	34945	35702	36400	35113
卫生机构床位数	张	2525	3984	3939	3953	4330
卫生技术人员	人	3658	3820	3893	3912	4089
# 执业(助理)医师	人	1671	1565	1599	1536	1435
注册护师、护士	人	1191	1471	1472	1556	1702

?1994-2018 China Academic Journal Electronic Publishing House. All rights reserved. http://www.cnki.net

社会主要指标

2009年	2010年	2011年	2012年	2013年	2014年	2015年	2016年
50.59	50.74	50.85	51.03	51.17	51.31	51.46	51.60
172.34	207.81	279.17	330.04	375.29	425.01	466.54	519.60
9.54	11.18	12.78	14.31	14.53	15.34	15.59	16.42
93.61	118.24	175.54	218.55	247.88	283.49	316.15	351.84
69.19	78.40	90.85	97.18	112.87	126.18	134.80	151.34
76.94	98.25	151.44	192.00	218.39	250.83	280.84	310.22
34898	41508	54959	64790	73441	82944	90793	100833
115.6	114.3	114.8	113.7	114.5	112.7	111.8	108.4
2070518	2701864	3179800	4060420	5168857	6225891	7626716	8819488
31578	40613	53846	71605	86112	100079	95394	97022
58151	79347	115940	148444	176133	178070	179622	226998
5195	6234	7948	9355	10787	12211	11398	12321
17492	20648	24530	28798	32081	35738	32498	34983
11633	11513	11233	10335	9962	8891	8524	8524
83976	82125	60046	70388	62770	58922	60011	56118
164325	190045	216778	238008	270448	285600	299386	316937
475488	568671	669184	805056	938524	1091781	1235389	1435060
1680	1899	1460	1428	1320	1284	1269	1226
31797	36336	36398	35503	36143	37221	39544	41585
1895	2207	2242	2275	2784	3058	3208	3351
35092	39757	38719	39335	38907	38603	38198	38540
4631	4632	5104	5344	5950	7035	7335	7464
5569	6019	6317	6643	7520	8438	8904	9947
2049	1802	2065	2185	2387	2481	2747	3042
2046	2426	2577	2971	3387	3976	4114	4731

?1994-2018 China Academic Journal Electronic Publishing House. All rights reserved. http://www.cnki.net

7-3　渭城区经济

指　标	单 位	2000年	2005年	2006年	2007年	2008年
年底总人口	万人	37.17	42.29	42.56	42.73	42.99
生产总值	亿元		87.33	93.60	109.40	139.38
第一产业	亿元		4.14	3.99	4.63	5.70
第二产业	亿元		59.18	65.45	76.20	100.38
第三产业	亿元		24.01	24.16	28.57	33.30
# 工业增加值	亿元		40.70	44.45	52.20	71.81
人均生产总值	元		21307	22086	25654	32522
生产总值指数	上年=100		117.1	113.0	109.7	113.2
全社会固定资产投资	万元	129112	518198	653124	847622	1244340
地方财政收入	万元	9666	11200	12467	14268	20034
地方财政支出	万元	9865	17106	21097	30942	44161
农村居民人均纯收入	元	2348	3069	3233	3550	4255
城镇居民人均可支配收入	元	5812	8780	9432	10970	14118
常用耕地面积	公顷	15975	15468	15366	15289	15271
粮食产量	吨	110321	114786	118055	107240	122012
农林牧渔业总产值	万元	32355	62220	66132	80776	95830
社会消费品零售总额	万元	34006	230802	253864	291876	352689
普通小学专任教师数	人	1615	1737	1766	1809	1697
普通小学在校学生数	人	38875	27918	26645	25899	25437
普通中学专任教师数	人	1531	1907	1829	1927	1971
普通中学在校学生数	人	30786	35777	34347	34322	32475
卫生机构床位数	张	1252	1802	1945	2338	2686
卫生技术人员	人	1867	1680	1714	2387	2868
# 执业(助理)医师	人	862	741	742	891	990
注册护师、护士	人	605	652	658	902	1159

?1994-2018 China Academic Journal Electronic Publishing House. All rights reserved.　http://www.cnki.net

社会主要指标

2009年	2010年	2011年	2012年	2013年	2014年	2015年	2016年
43.73	43.86	43.95	44.06	44.14	44.23	44.39	44.52
149.67	195.14	228.01	266.44	305.22	316.43	313.09	344.90
6.27	8.57	10.74	11.93	12.43	13.28	13.40	13.68
102.93	140.65	164.47	194.55	222.99	226.04	216.49	239.56
40.47	45.92	52.80	59.97	69.81	77.11	83.20	91.66
61.52	93.96	105.63	127.50	149.09	128.19	109.34	113.83
34818	44937	51932	60545	69206	71613	70661	77585
112.2	114.3	114.7	114.7	112.3	110.8	108.5	107.4
1790842	2336054	2780400	3558950	4522758	5470881	6721033	7725134
23382	32047	45067	60551	74116	85564	73342	81082
55638	72377	99786	118807	134161	149388	158298	192938
5127	6152	7831	9218	10582	11979	11183	12053
17448	20608	24462	28694	31936	35545	32316	34621
15194	14328	14048	13124	12660	12421	12100	11964
121887	126080	90058	107594	97237	92962	94916	88941
104627	131730	173773	200596	223316	232705	240934	249961
410267	482884	563381	660602	759967	862483	975536	1083460
1737	1713	1367	1354	1346	1290	1237	1362
24710	24327	23884	22248	21916	22135	22999	24189
2000	1957	1970	1869	2200	2171	2251	2612
30454	26842	26126	24325	23495	22808	22625	21296
3413	3157	3716	3734	3924	4335	4719	5325
3797	3947	4213	4945	5350	5557	5811	6528
1226	1435	1467	1593	1745	1894	1956	2169
1619	1702	1825	2204	2399	2587	2686	3132

?1994-2018 China Academic Journal Electronic Publishing House. All rights reserved.   http://www.cnki.net

7-4　三原县经济

指　　标	单 位	2000年	2005年	2006年	2007年	2008年
年底总人口	万人	39.63	39.88	39.84	39.99	40.04
生产总值	亿元	17.62	36.31	41.51	52.07	65.85
第一产业	亿元	5.19	10.62	12.08	15.02	16.70
第二产业	亿元	5.95	13.69	16.78	22.25	31.80
第三产业	亿元	6.48	12.00	12.65	14.80	17.35
# 工业增加值	亿元	5.67	12.76	15.76	20.93	30.30
人均生产总值	元	4501	9047	10414	13045	16456
生产总值指数	上年=100	112.7	113.3	112.0	113.3	114.6
全社会固定资产投资	万元	27597	118453	150359	212174	322965
地方财政收入	万元	7417	4660	5827	7418	9643
地方财政支出	万元	11979	23655	30544	42666	57196
农村居民人均纯收入	元	1683	2357	2607	3111	3832
城镇居民人均可支配收入	元	4005	6836	7508	8815	13537
常用耕地面积	公顷	33961	35746	35635	35577	35573
粮食产量	吨	182412	230661	239526	206393	227799
农林牧渔业总产值	万元	101905	178835	205735	254542	303094
社会消费品零售总额	万元	40525	95846	106439	122505	150309
普通小学专任教师数	人	2071	2387	2399	2422	2304
普通小学在校学生数	人	51684	30426	30338	27551	25811
普通中学专任教师数	人	2071	2124	2204	2314	2399
普通中学在校学生数	人	33894	38748	39632	39086	35985
卫生机构床位数	张	769	919	931	1027	1058
卫生技术人员	人	1414	1126	1174	1321	1333
# 执业(助理)医师	人	566	544	566	625	558
注册护师、护士	人	319	237	245	338	369

?1994-2018 China Academic Journal Electronic Publishing House. All rights reserved.　http://www.cnki.net

社会主要指标

2009年	2010年	2011年	2012年	2013年	2014年	2015年	2016年
40.25	40.38	40.51	40.66	40.76	40.89	41.01	41.13
76.62	90.28	103.40	120.48	140.81	160.13	177.63	193.51
17.50	21.20	23.50	26.12	27.41	29.17	29.87	30.64
37.77	44.49	51.16	62.57	74.97	87.32	100.69	110.71
21.35	24.59	28.74	31.79	38.44	43.64	47.07	52.15
36.17	41.79	48.15	59.13	71.03	82.63	95.55	96.74
19134	22448	25564	29687	34591	39224	43377	47117
114.2	115.1	114.5	113.6	114.7	112.3	111.5	108.3
441335	575116	695401	903000	1142357	1361547	1665944	2058476
12580	16896	22840	30201	36723	43389	48543	30303
70818	99777	126058	158501	184626	188235	212525	219181
4690	5670	7190	8383	9607	10846	10135	10961
16943	19501	22908	26619	29387	32561	29570	31851
35630	35614	33168	33208	33129	33055	32980	32902
217418	235988	193134	206465	200001	195125	201111	196009
306385	371211	404796	450714	488895	509048	514073	530811
183667	215240	251479	284712	327427	362789	412871	460223
2247	2224	2233	2105	1956	1830	1759	1762
23917	23092	22637	21605	19401	18622	19408	20926
2424	2432	2522	2487	2495	2522	2489	2483
34435	31985	29640	29653	25466	22034	18227	18239
1162	1168	1194	1342	1592	1770	1791	1780
2114	2492	2617	2739	2832	2876	3017	3291
578	485	517	548	576	615	632	624
464	583	617	644	676	782	850	1016

?1994-2018 China Academic Journal Electronic Publishing House. All rights reserved.   http://www.cnki.net

7-5 泾阳县经济

指 标	单 位	2000年	2005年	2006年	2007年	2008年
年底总人口	万人	48.34	50.04	50.01	50.21	50.27
生产总值	亿元	18.89	37.75	43.89	52.84	65.10
第一产业	亿元	7.85	15.71	18.70	20.68	26.60
第二产业	亿元	5.71	10.63	12.12	16.28	20.01
第三产业	亿元	5.33	11.41	13.07	15.88	18.49
# 工业增加值	亿元	5.04	9.28	10.81	14.68	18.11
人均生产总值	元	3915	7558	8774	10545	12958
生产总值指数	上年=100	109.5	113.7	110.8	114.3	116.3
全社会固定资产投资	万元	26725	137336	172830	217323	317425
地方财政收入	万元	7200	4505	5355	6364	8538
地方财政支出	万元	11905	20862	26678	40819	56989
农村居民人均纯收入	元	1780	2495	2750	3120	3840
城镇居民人均可支配收入	元				9787	13556
常用耕地面积	公顷	44388	45482	45299	45320	45337
粮食产量	吨	222529	249264	261954	258177	284086
农林牧渔业总产值	万元	124130	265732	320050	384040	437295
社会消费品零售总额	万元	29616	101141	113627	132654	165171
普通小学专任教师数	人	2188	2250	2126	2268	2618
普通小学在校学生数	人	66766	45011	40417	35296	30712
普通中学专任教师数	人	1898	2384	2359	2384	3075
普通中学在校学生数	人	37588	41731	42607	42378	42346
卫生机构床位数	张	490	862	912	1182	1479
卫生技术人员	人	999	984	1002	945	1016
# 执业(助理)医师	人	460	370	362	409	423
注册护师、护士	人	236	208	209	260	292

?1994-2018 China Academic Journal Electronic Publishing House. All rights reserved. http://www.cnki.net

社会主要指标

2009年	2010年	2011年	2012年	2013年	2014年	2015年	2016年
48.63	48.81	49.00	49.16	49.33	49.50	49.70	49.84
74.04	87.40	101.52	118.02	135.01	152.03	160.23	179.04
27.69	30.26	35.23	38.64	40.87	43.86	45.00	46.42
23.27	30.52	35.16	44.02	52.98	61.85	64.38	75.24
23.07	26.62	31.13	35.37	41.15	46.32	50.84	57.38
21.28	27.92	32.16	40.89	49.45	57.71	59.72	66.14
14725	17638	20758	24046	27414	30766	32302	35972
114.4	114.4	112.5	113.5	113.1	111.0	111.5	108.0
441529	575620	716576	917000	1165063	1794316	2323692	3318503
11385	17360	22223	25399	32586	40056	28371	26315
78065	110998	127369	159800	209128	218719	219136	247594
4715	5691	7205	8379	9561	10794	10106	10940
16952	19529	22915	26513	29270	32431	29458	31730
45355	45413	44427	44397	43757	43100	42459	41995
277391	287937	248685	257271	242447	239839	247212	240797
453673	522869	576531	632757	701427	737796	756662	791330
188271	217433	250432	285777	328745	378386	433399	530396
2827	2965	2920	2936	2754	2641	2353	2198
28945	28509	28848	26763	23785	24776	25452	26855
3204	3255	3382	3272	3325	3166	3001	2476
41747	40654	36687	32154	25105	23567	21684	20144
1235	1306	1446	1574	1882	2364	2302	2229
1224	1529	2002	2141	2528	2855	3101	3438
787	501	678	687	748	776	802	755
430	459	789	956	1164	1424	1588	1857

?1994-2018 China Academic Journal Electronic Publishing House. All rights reserved. http://www.cnki.net

7-6 乾县经济

指　标	单 位	2000年	2005年	2006年	2007年	2008年
年底总人口	万人	54.18	56.12	56.06	56.34	56.38
生产总值	亿元	14.76	30.45	35.05	42.28	52.52
第一产业	亿元	5.65	8.04	8.72	10.49	12.40
第二产业	亿元	4.12	10.59	13.41	16.17	20.75
第三产业	亿元	4.99	11.82	12.92	15.62	19.37
# 工业增加值	亿元	3.34	9.53	12.26	14.84	18.65
人均生产总值	元	2735	5439	6249	7523	9319
生产总值指数	上年=100	116.8	112.8	112.2	116.3	115.9
全社会固定资产投资	万元	35720	102457	139027	215236	294852
地方财政收入	万元	8034	4102	4574	5530	7391
地方财政支出	万元	10438	21357	30309	41997	60863
农村居民人均纯收入	元	1620	2266	2509	3069	3788
城镇居民人均可支配收入	元	3337	5180	5728	7813	12598
常用耕地面积	公顷	50111	50458	50581	50569	50570
粮食产量	吨	270276	268541	299681	269366	297584
农林牧渔业总产值	万元	86680	130577	145860	179712	206689
社会消费品零售总额	万元	30039	129599	148611	176576	223029
普通小学专任教师数	人	2858	3229	3116	3115	3078
普通小学在校学生数	人	76732	54306	61778	59935	52946
普通中学专任教师数	人	1668	2319	2465	2531	2627
普通中学在校学生数	人	34432	57161	61161	60907	57891
卫生机构床位数	张	621	626	1009	1095	1031
卫生技术人员	人	1115	965	965	986	1110
# 执业(助理)医师	人	605	517	517	517	543
注册护师、护士	人	214	180	180	210	265

?1994-2018 China Academic Journal Electronic Publishing House. All rights reserved.　http://www.cnki.net

社会主要指标

2009年	2010年	2011年	2012年	2013年	2014年	2015年	2016年
52.56	52.75	52.85	52.98	53.08	53.20	53.40	53.52
59.72	73.47	92.98	108.09	127.21	145.04	148.74	162.38
13.05	17.05	22.33	25.48	27.86	29.62	30.46	31.67
22.24	29.13	37.58	46.54	57.82	67.42	68.28	74.25
24.43	27.29	33.07	36.08	41.53	48.00	50.00	56.46
19.78	25.68	33.79	42.12	52.13	60.59	60.63	63.10
10588	13459	17610	20427	23986	27293	27904	30373
115.0	115.9	114.5	113.5	113.2	111.6	111.6	108.4
452379	600152	722132	924000	1171750	1382234	1687017	2036744
8713	10587	14510	18622	22520	23960	26880	16357
75100	99716	130190	172992	196555	223096	228156	239018
4678	5665	7155	8314	9495	10700	10007	10773
15726	18274	21516	24915	27481	30421	27620	29673
50722	51128	51128	51258	49915	48443	46630	46642
293684	305733	276961	268199	253101	250097	262896	255915
217548	279697	366942	426941	465953	513509	535346	556117
261084	305810	352532	404720	465009	526390	593759	661175
3031	2994	2938	2964	2956	2525	2352	2146
47811	53088	53858	49775	47378	42874	39447	33623
2639	2630	2646	2701	2608	2676	2650	2466
53899	53580	55897	51069	44977	38904	33016	28814
999	1040	1030	1368	1467	1579	1715	1779
1107	1785	2192	2514	2578	2881	2731	2887
436	458	458	481	511	614	594	619
263	654	695	826	833	954	900	960

?1994-2018 China Academic Journal Electronic Publishing House. All rights reserved.   http://www.cnki.net

7-7 礼泉县经济

指 标	单 位	2000年	2005年	2006年	2007年	2008年
年底总人口	万人	45.56	46.28	46.23	46.45	46.52
生产总值	亿元	15.63	27.58	32.05	37.94	48.90
第一产业	亿元	6.29	9.67	10.69	12.24	16.00
第二产业	亿元	3.98	6.31	8.38	10.40	14.66
第三产业	亿元	5.36	11.60	12.98	15.30	18.24
# 工业增加值	亿元	3.37	5.43	7.18	8.91	12.99
人均生产总值	元	3449	5990	6929	8187	10519
生产总值指数	上年=100	102.8	112.7	112.0	111.4	115.8
全社会固定资产投资	万元	15912	116595	156880	226562	334400
地方财政收入	万元	8167	3110	3705	4164	6083
地方财政支出	万元	12276	20626	29336	40757	55886
农村居民人均纯收入	元	2119	2560	2850	3238	3886
城镇居民人均可支配收入	元				7300	12611
常用耕地面积	公顷	38222	36352	36237	33525	30761
粮食产量	吨	183791	149288	138959	109427	122475
农林牧渔业总产值	万元	107956	144767	163334	200390	253977
社会消费品零售总额	万元	35227	102132	116663	138540	175302
普通小学专任教师数	人	3829	3274	3116	2995	3080
普通小学在校学生数	人	75311	59187	53847	48677	47223
普通中学专任教师数	人	1991	2293	2310	2336	2406
普通中学在校学生数	人	37826	48216	48915	46520	47572
卫生机构床位数	张	819	915	924	932	956
卫生技术人员	人	1629	1489	1335	1475	1464
# 执业(助理)医师	人	630	559	468	472	472
注册护师、护士	人	465	441	412	434	444

?1994-2018 China Academic Journal Electronic Publishing House. All rights reserved. http://www.cnki.net

社会主要指标

2009年	2010年	2011年	2012年	2013年	2014年	2015年	2016年
44.64	44.81	44.92	45.07	45.21	45.34	45.48	45.61
54.85	72.57	93.24	108.62	127.86	145.00	151.83	164.15
17.13	26.57	37.36	42.68	46.49	50.18	50.80	54.04
14.58	20.90	25.85	36.10	46.41	56.05	58.23	61.54
23.14	25.10	30.03	29.83	34.96	38.77	42.81	48.57
12.82	19.07	23.75	33.73	42.90	51.60	55.26	57.19
11785	15882	20781	24140	28326	32026	33437	36043
115.1	115.0	114.5	113.8	113.2	110.8	110.8	108.5
492140	649600	777800	995000	1259574	1487784	1820318	2320070
9009	12170	16893	21565	26025	21728	23962	19005
71257	99261	124209	168364	200351	195649	215694	227528
4687	5695	7181	8381	9571	10796	10089	10922
15726	18258	21501	25048	27628	30584	27755	29818
30890	31137	30818	30850	30033	28865	28876	28874
128861	153013	115603	130453	123113	120567	126619	122883
286423	408686	601820	665289	729211	781606	796999	851118
203456	237349	277258	312932	361727	410561	462883	539774
3040	2996	2954	2944	2802	2580	2376	2271
45894	43318	42638	39484	31579	26784	26539	25743
2402	2417	2495	2495	2653	2644	2603	2552
46019	44204	44763	40733	31074	26492	21505	21075
914	1024	1031	1115	1193	1593	1761	1834
1322	1892	1899	2101	2146	1858	1990	2297
515	569	555	553	573	537	546	578
424	517	514	545	557	660	679	749

?1994-2018 China Academic Journal Electronic Publishing House. All rights reserved. http://www.cnki.net

7-8　永寿县经济

指　　标	单 位	2000年	2005年	2006年	2007年	2008年
年底总人口	万人	18.28	19.58	19.51	19.60	19.62
生产总值	亿元	3.32	7.22	8.16	10.63	14.72
第一产业	亿元	1.29	2.98	3.65	4.36	6.20
第二产业	亿元	0.67	1.56	1.97	2.82	3.61
第三产业	亿元	1.36	2.68	2.54	3.45	4.91
# 工业增加值	亿元	0.58	1.26	1.66	2.33	2.91
人均生产总值	元	1828	3772	4184	5436	7506
生产总值指数	上年=100	104.2	114.5	115.1	114.3	115.9
全社会固定资产投资	万元	14398	68098	76447	97469	140811
地方财政收入	万元	2674	1580	1869	2318	3468
地方财政支出	万元	6090	10953	17221	26857	36559
农村居民人均纯收入	元	993	1420	1700	1975	2900
城镇居民人均可支配收入	元				5806	10914
常用耕地面积	公顷	29369	28169	28133	19346	19346
粮食产量	吨	65896	78981	91700	71875	92714
农林牧渔业总产值	万元	24314	53960	60353	74459	99766
社会消费品零售总额	万元	5587	33413	37107	42961	53022
普通小学专任教师数	人	1194	1255	1267	1272	1284
普通小学在校学生数	人	29602	24369	23480	21815	20539
普通中学专任教师数	人	530	726	755	789	833
普通中学在校学生数	人	10063	16067	16284	16332	16180
卫生机构床位数	张	383	410	411	425	452
卫生技术人员	人	558	488	490	463	525
# 执业(助理)医师	人	294	267	266	194	222
注册护师、护士	人	117	100	100	101	123

?1994-2018 China Academic Journal Electronic Publishing House. All rights reserved.　http://www.cnki.net

社会主要指标

2009年	2010年	2011年	2012年	2013年	2014年	2015年	2016年
18.41	18.48	18.53	18.58	18.62	18.69	18.75	18.81
17.52	23.07	28.02	34.87	41.33	44.83	50.00	58.61
6.97	9.75	10.82	12.38	13.31	14.30	15.01	15.98
4.84	5.96	8.52	12.76	16.49	17.65	21.16	26.83
5.71	7.36	8.68	9.73	11.54	12.88	13.84	15.80
3.89	5.01	7.44	11.38	14.73	15.40	19.09	23.70
8925	12102	15141	18795	22220	24031	26712	31206
114.3	117.4	113.0	113.6	113.2	110.3	111.7	110.9
190045	250031	301778	386360	488240	573486	697719	854396
4271	5738	8048	10538	13079	14096	14800	8276
45768	65576	76796	101489	115710	117655	123377	137251
3585	4352	5457	6391	7317	8232	7720	8419
13533	15671	18355	21256	23530	26024	23577	25490
19346	19403	19445	19256	19553	20402	20405	20472
96583	96932	75780	84735	82000	81018	89068	87584
111188	148026	171254	195466	250900	273123	292511	312678
60882	80737	94170	107899	124291	143928	180459	208526
1287	1215	1115	1016	942	915	905	906
19499	17734	15176	13650	12912	12725	12669	12609
832	834	834	862	892	881	860	849
16093	14627	13438	12808	10773	9601	9027	8780
493	509	493	515	697	814	870	953
567	661	694	771	1258	1161	1119	1105
256	218	215	226	256	262	251	254
150	184	181	207	329	325	324	344

?1994-2018 China Academic Journal Electronic Publishing House. All rights reserved. http://www.cnki.net

7-9 彬县经济

指　　标	单 位	2000年	2005年	2006年	2007年	2008年
年底总人口	万人	31.63	32.28	32.39	32.54	32.64
生产总值	亿元	6.41	14.84	18.26	24.51	43.86
第一产业	亿元	2.33	3.83	4.05	4.80	6.60
第二产业	亿元	1.97	5.66	8.14	12.72	28.87
第三产业	亿元	2.11	5.35	6.07	6.99	8.39
# 工业增加值	亿元	1.83	5.33	7.79	12.07	27.75
人均生产总值	元	2036	4558	5619	7550	13458
生产总值指数	上年=100	104.6	124.6	122.2	124.4	129.6
全社会固定资产投资	万元	18752	125956	161591	211964	335327
地方财政收入	万元	5103	4526	7864	19340	26031
地方财政支出	万元	8428	20958	31797	49722	71289
农村居民人均纯收入	元	1249	1837	2004	2292	3078
城镇居民人均可支配收入	元	2600	4825	5050	6305	12187
常用耕地面积	公顷	35614	28979	28899	28951	29077
粮食产量	吨	79482	108007	107517	79804	106804
农林牧渔业总产值	万元	39487	64115	72001	90236	110059
社会消费品零售总额	万元	25273	57253	62560	70262	84247
普通小学专任教师数	人	1684	1731	1789	1754	1711
普通小学在校学生数	人	50607	41023	36976	32488	28887
普通中学专任教师数	人	764	1147	1312	1362	1421
普通中学在校学生数	人	18208	29751	32163	31904	29989
卫生机构床位数	张	622	619	669	980	998
卫生技术人员	人	858	837	806	779	876
# 执业(助理)医师	人	390	420	395	371	340
注册护师、护士	人	252	244	238	204	321

?1994-2018 China Academic Journal Electronic Publishing House. All rights reserved.　http://www.cnki.net

社会主要指标

2009年	2010年	2011年	2012年	2013年	2014年	2015年	2016年
32.23	32.35	32.47	32.56	32.66	32.79	32.85	32.95
60.06	80.17	106.10	136.18	165.43	186.10	170.01	188.82
7.15	9.78	12.60	14.45	15.67	16.82	17.24	17.88
41.45	56.57	77.24	103.35	129.26	146.45	128.50	142.71
11.47	13.81	16.27	18.38	20.50	22.83	24.28	28.23
39.62	53.44	73.91	99.34	124.72	141.00	122.43	134.68
18393	24659	32737	41884	50726	56866	51803	57395
122.6	121.3	123.3	120.6	117.8	112.6	109.7	108.0
508990	666046	863711	1105000	1409690	1659969	2025405	2172789
38397	50871	89354	86362	100118	108287	100210	75102
89357	138993	189528	209539	231363	210943	224096	244665
4103	5121	6601	7835	9058	10271	9640	10453
15356	18151	21516	25217	28041	31126	28277	30515
29407	29791	29884	30551	31169	31283	31430	31583
120883	139739	119392	129983	118286	115331	125622	123049
124200	170612	219965	252341	277029	297830	307949	318129
112381	136397	161143	187883	217726	252562	285386	336827
1725	1669	1607	1443	1407	1410	1328	1261
27225	26366	26596	26246	25991	25417	25550	26716
1403	1409	1338	1329	1429	1377	1315	1286
28824	27124	24974	22376	19057	16296	14372	14434
904	1012	1053	1512	1562	1602	1681	1856
1647	1777	1851	1929	1933	2009	2019	2167
542	385	388	400	398	389	383	396
556	702	697	740	725	771	796	977

?1994-2018 China Academic Journal Electronic Publishing House. All rights reserved. http://www.cnki.net

7-10 长武县经济

指 标	单 位	2000年	2005年	2006年	2007年	2008年
年底总人口	万人	17.11	17.04	16.95	17.03	17.06
生产总值	亿元	2.79	6.79	8.05	10.15	15.81
第一产业	亿元	1.14	1.87	2.03	2.53	3.30
第二产业	亿元	0.66	1.83	2.43	3.86	8.18
第三产业	亿元	0.99	3.09	3.59	3.76	4.33
# 工业增加值	亿元	0.38	1.39	1.90	3.07	7.15
人均生产总值	元	1631	3957	4737	5974	9277
生产总值指数(上年=100)	上年=100	111.6	115.0	111.2	115.9	122.9
全社会固定资产投资	万元	17197	53237	69190	120671	274492
地方财政收入	万元	2621	2029	2275	16809	6227
地方财政支出	万元	5602	11263	15133	34017	31552
农村居民人均纯收入	元	1009	1550	1700	1922	2895
城镇居民人均可支配收入	元				5909	11069
常用耕地面积	公顷	19346	10609	11427	11464	11464
粮食产量	吨	50191	54961	53593	42285	56225
农林牧渔业总产值	万元	21510	34465	37681	44772	54563
社会消费品零售总额	万元	7366	30196	34590	41285	52865
普通小学专任教师数	人	911	1170	1135	1174	1089
普通小学在校学生数	人	24476	19453	18247	16167	13898
普通中学专任教师数	人	609	794	820	895	914
普通中学在校学生数	人	10285	12231	12272	12124	11587
卫生机构床位数	张	309	347	353	449	492
卫生技术人员	人	444	432	468	471	523
# 执业(助理)医师	人	248	205	207	186	178
注册护师、护士	人	104	129	129	131	121

?1994-2018 China Academic Journal Electronic Publishing House. All rights reserved. http://www.cnki.net

社会主要指标

2009年	2010年	2011年	2012年	2013年	2014年	2015年	2016年
16.70	16.77	16.83	16.90	16.95	17.01	17.06	17.12
17.80	24.95	34.98	44.62	52.58	60.61	68.83	73.00
3.70	5.49	10.90	12.60	13.66	14.74	15.28	15.87
8.76	14.04	17.93	24.96	30.73	36.59	43.09	45.42
5.34	5.42	6.15	7.06	8.18	9.28	10.47	11.71
7.38	11.62	14.50	20.94	26.11	31.33	37.30	43.93
10433	14745	20822	26457	31066	35687	40408	42720
112.1	123.5	119.1	116.1	116.8	112.4	109.6	107.5
372598	480377	565661	723000	917761	1079825	1323357	1559353
22381	20003	27761	23554	28060	32013	35075	31960
53286	80024	83851	90846	110669	110419	124968	147557
3583	4407	5645	6622	7556	8508	7987	8702
13714	15991	18869	22021	24399	26936	24420	26401
11452	11475	10914	11058	11223	11666	11708	11775
61527	63718	49743	62665	58921	55175	58666	57703
61684	90094	179082	208938	233416	292219	307462	319740
62561	73855	85114	99383	114505	132024	151067	172136
1082	1044	968	921	888	881	844	903
12164	10893	9995	9389	9334	9484	9715	9916
896	913	913	907	851	849	783	765
11001	10907	10532	9756	8716	7919	7210	7051
486	510	570	650	647	686	791	802
584	707	883	976	1080	1166	1225	1293
271	155	181	202	211	207	207	208
156	180	195	234	255	277	285	285

?1994-2018 China Academic Journal Electronic Publishing House. All rights reserved.   http://www.cnki.net

7-11 旬邑县经济

指　　标	单 位	2000年	2005年	2006年	2007年	2008年
年底总人口	万人	26.76	27.04	26.98	27.11	27.14
生产总值	亿元	5.04	11.43	13.61	19.14	28.75
第一产业	亿元	2.78	5.04	5.66	6.74	8.80
第二产业	亿元	0.90	3.04	3.77	7.66	14.34
第三产业	亿元	1.35	3.35	4.18	4.74	5.61
# 工业增加值	亿元	0.79	2.61	3.35	7.11	13.64
人均生产总值	元	1904	4227	5039	7077	10599
生产总值指数	上年=100	110.3	133.4	109.4	123.3	119.1
全社会固定资产投资	万元	8711	60451	88643	117602	180142
地方财政收入	万元	3670	2386	2699	3741	6741
地方财政支出	万元	7159	16118	23320	30920	45000
农村居民人均纯收入	元	1324	1737	1883	2200	2890
城镇居民人均可支配收入	元	3100	5070	5580	5900	10724
常用耕地面积	公顷	28291	27173	27293	27386	27444
粮食产量	吨	86795	107939	107789	90793	101859
农林牧渔业总产值	万元	39996	80040	89944	114712	145078
社会消费品零售总额	万元	9212	40403	45123	52613	65351
普通小学专任教师数	人	1574	1674	1576	1645	1638
普通小学在校学生数	人	41391	33538	31386	28133	25176
普通中学专任教师数	人	767	1103	1100	1118	1149
普通中学在校学生数	人	15838	22897	23895	24663	25286
卫生机构床位数	张	373	450	476	526	546
卫生技术人员	人	657	572	574	671	917
# 执业(助理)医师	人	323	235	229	263	411
注册护师、护士	人	165	111	112	149	254

?1994-2018 China Academic Journal Electronic Publishing House. All rights reserved.　http://www.cnki.net

社会主要指标

2009年	2010年	2011年	2012年	2013年	2014年	2015年	2016年
26.08	26.18	26.28	26.38	26.45	26.54	26.58	26.66
34.70	49.32	68.36	86.84	102.28	116.02	104.92	106.63
9.43	14.21	21.18	23.26	24.42	26.82	27.06	29.29
19.26	28.07	39.16	54.36	65.72	75.26	61.94	60.21
6.01	7.04	8.03	9.23	12.14	13.94	15.92	17.13
18.51	27.31	38.34	53.52	64.75	74.10	60.65	54.71
12779	18492	26065	32983	38723	43789	44772	40055
115.9	121.0	118.4	117.9	116.2	110.2	108.4	107.8
250145	330030	397844	509000	646140	771425	939301	1124337
10616	15011	24070	27596	30167	20261	22443	18527
60600	90062	113002	132368	142504	145102	153941	188720
3580	4368	5617	6662	7701	8741	8208	8951
13469	15516	18324	21403	23715	26229	23789	25719
27479	27646	27766	27766	27767	27678	27752	27816
104656	116742	98749	126735	118923	108028	112523	110004
157125	232592	352962	386944	434653	486744	512243	526502
82744	96253	110746	127688	147196	169129	193398	222120
1615	1605	1476	1462	1468	1421	1422	1413
22187	20073	18811	16084	15445	15867	16570	17551
1196	1213	1227	1290	1365	1323	1250	1215
26105	24531	23762	17907	14385	12781	12079	11539
576	700	770	771	832	862	911	929
669	1001	1272	1568	1646	1685	1785	1810
397	325	293	349	341	327	376	373
199	280	377	459	486	482	512	525

?1994-2018 China Academic Journal Electronic Publishing House. All rights reserved.   http://www.cnki.net

7-12 淳化县经济

指　　标	单 位	2000年	2005年	2006年	2007年	2008年
年底总人口	万人	18.60	19.90	19.84	19.93	19.91
生产总值	亿元	4.99	9.75	11.64	15.44	20.54
第一产业	亿元	3.13	5.48	6.21	9.17	12.70
第二产业	亿元	0.69	1.58	2.34	3.13	4.15
第三产业	亿元	1.17	2.69	3.09	3.14	3.69
# 工业增加值	亿元	0.62	1.37	2.00	2.51	3.41
人均生产总值	元	2702	4920	5858	7765	10310
生产总值指数	上年=100	131.5	115.4	109.6	109.9	115.4
全社会固定资产投资	万元	17961	39484	50400	71979	96493
地方财政收入	万元	3791	1326	1496	1823	2408
地方财政支出	万元	6302	12452	17935	23458	38041
农村居民人均纯收入	元	1205	1704	1883	2192	2946
城镇居民人均可支配收入	元				6005	10200
常用耕地面积	公顷	34060	25621	25655	26179	26561
粮食产量	吨	95154	98076	101662	82586	97938
农林牧渔业总产值	万元	57832	104103	118391	154948	215764
社会消费品零售总额	万元	13690	34669	38013	43469	52574
普通小学专任教师数	人	1219	1371	1373	1334	1300
普通小学在校学生数	人	31756	30911	30545	30531	30392
普通中学专任教师数	人	685	844	863	861	890
普通中学在校学生数	人	11844	16751	17467	17531	16790
卫生机构床位数	张	245	322	453	459	506
卫生技术人员	人	455	438	408	452	520
# 执业(助理)医师	人	190	133	124	129	134
注册护师、护士	人	75	55	53	66	100

?1994-2018 China Academic Journal Electronic Publishing House. All rights reserved.　http://www.cnki.net

社会主要指标

2009年	2010年	2011年	2012年	2013年	2014年	2015年	2016年
19.29	19.35	19.39	19.45	19.50	19.57	19.61	19.66
23.48	32.22	36.80	42.67	50.25	53.54	57.38	64.91
12.16	17.65	19.33	21.74	21.39	22.87	23.17	25.02
5.72	8.35	10.44	12.82	16.85	17.23	19.39	24.24
5.60	6.22	7.04	8.11	12.01	13.44	14.82	15.65
4.77	6.05	8.70	10.39	14.04	13.87	15.59	19.39
11786	16405	18998	21973	25799	27406	29289	33061
114.2	114.0	112.2	113.0	113.0	110.0	109.8	108.0
132293	175421	205100	260000	332899	390043	469909	587976
2853	4285	6043	8001	9500	6066	7037	6241
43718	76851	84086	107593	112882	115757	136172	161053
3645	4476	5622	6572	7518	8451	7929	8639
12821	14911	17450	20173	22311	24654	22346	25240
26684	26817	24221	25432	26034	27330	28754	28756
107151	121995	95825	124294	115544	110000	118043	115709
223917	296990	332235	376957	417469	456662	460098	480087
61771	75552	87260	100652	115805	133871	153041	175833
1236	1240	1165	1114	1028	956	924	881
29832	28931	27732	21658	13223	9695	8200	7925
929	924	970	949	1004	992	981	910
16208	15682	14436	12074	10354	8822	7398	6826
504	498	508	557	602	629	619	599
679	745	783	895	1040	1028	1049	1160
231	149	177	184	193	174	179	190
121	137	151	167	182	182	187	212

?1994-2018 China Academic Journal Electronic Publishing House. All rights reserved. http://www.cnki.net

7-13　武功县经济

指　　标	单 位	2000年	2005年	2006年	2007年	2008年
年底总人口	万人	40.30	42.03	42.01	42.22	42.27
生产总值	亿元	11.84	21.65	25.68	32.57	39.30
第一产业	亿元	3.19	5.65	7.06	11.60	11.70
第二产业	亿元	4.28	8.25	9.30	10.51	15.92
第三产业	亿元	4.37	7.75	9.32	10.46	11.68
# 工业增加值	亿元	3.80	7.18	7.92	8.84	14.00
人均生产总值	元	2953	5185	6111	7734	9303
生产总值指数	上年=100	112.8	108.0	112.7	116.4	115.1
全社会固定资产投资	万元	10119	54713	71497	95208	127523
地方财政收入	万元	3499	2263	2438	3011	4030
地方财政支出	万元	8259	19366	27629	34721	48254
农村居民人均纯收入	元	1603	2304	2576	2950	3798
城镇居民人均可支配收入	元				7492	12329
常用耕地面积	公顷	28064	27512	27541	27572	27565
粮食产量	吨	189332	218592	245906	206687	224598
农林牧渔业总产值	万元	55705	101333	117548	146163	186759
社会消费品零售总额	万元	30987	86066	96140	112243	139000
普通小学专任教师数	人	2203	2758	2413	2299	2262
普通小学在校学生数	人	63951	48472	44200	40386	38284
普通中学专任教师数	人	1643	2141	2222	2246	2214
普通中学在校学生数	人	33305	43267	43464	42780	40207
卫生机构床位数	张	704	875	885	1003	1011
卫生技术人员	人	1120	844	845	1193	1129
# 执业(助理)医师	人	427	336	337	415	381
注册护师、护士	人	341	314	316	350	353

?1994-2018 China Academic Journal Electronic Publishing House. All rights reserved.　http://www.cnki.net

社会主要指标

2009年	2010年	2011年	2012年	2013年	2014年	2015年	2016年
41.01	41.16	41.30	41.47	41.61	41.74	41.87	42.00
44.72	55.52	63.60	74.14	95.94	110.03	112.45	123.25
12.27	15.10	16.73	18.52	18.79	20.26	20.99	22.37
16.83	22.06	25.50	31.71	48.47	56.94	56.76	61.30
15.62	18.36	21.38	23.91	28.69	32.83	34.70	39.58
14.37	19.06	21.04	25.94	41.12	48.81	47.77	41.60
10575	13302	15425	17913	23095	26401	26898	29391
113.7	113.8	113.0	113.5	113.3	110.5	111.1	108.1
182489	248226	309552	396000	499265	586460	716964	845582
5211	6724	8663	10927	13245	15274	16529	10972
59721	87894	110030	141676	165429	172433	195187	209098
4656	5592	7035	8167	9335	10521	9857	10660
15362	17666	20758	24142	26677	29585	26865	28808
27549	27542	26501	26528	26767	26906	26937	26955
229829	242925	206590	210562	200004	197222	203144	198003
202288	249821	276303	293249	323023	348078	362655	385201
166690	194730	224978	259003	292646	327179	374897	425042
2421	2518	2544	2462	2378	2152	2055	1949
40969	40881	39652	36203	29735	26163	25399	24967
2263	2241	2240	2092	1958	1979	1867	1804
38444	39063	37168	31269	25798	22903	23075	23233
1010	1028	1211	1478	1541	1633	1689	1746
1075	1138	1221	1319	2093	2122	2197	2196
431	370	399	436	541	573	601	596
372	413	436	482	695	711	750	795

?1994-2018 China Academic Journal Electronic Publishing House. All rights reserved. http://www.cnki.net

7-14 兴平市经济

指　　标	单 位	2000年	2005年	2006年	2007年	2008年
年底总人口	万人	55.50	56.17	56.17	56.45	56.48
生产总值	亿元	20.77	45.87	50.03	58.47	77.07
第一产业	亿元	4.38	8.26	8.72	10.12	12.80
第二产业	亿元	10.09	23.48	24.71	28.63	40.97
第三产业	亿元	6.30	14.13	16.60	19.72	23.30
# 工业增加值	亿元	8.28	20.49	21.85	25.50	34.91
人均生产总值	元	3794	8167	8907	10384	13649
生产总值指数	上年=100	111.6	116.5	110.9	113.3	115.3
全社会固定资产投资	万元	44947	140463	218428	284760	475181
地方财政收入	万元	6869	7007	7923	9508	12858
地方财政支出	万元	12365	21965	34940	45989	65297
农村居民人均纯收入	元	1609	2359	2597	2960	3908
城镇居民人均可支配收入	元				8858	13600
常用耕地面积	公顷	34562	34942	34990	35004	35146
粮食产量	吨	213083	229172	239948	208773	233780
农林牧渔业总产值	万元	74203	125154	142139	172079	214246
社会消费品零售总额	万元	36300	126282	141043	161907	198094
普通小学专任教师数	人	3076	3234	3105	3039	3086
普通小学在校学生数	人	74895	52492	53951	48510	45066
普通中学专任教师数	人	1925	2369	2406	2356	2343
普通中学在校学生数	人	33732	48106	50637	48947	49895
卫生机构床位数	张	1446	1262	1416	1421	1500
卫生技术人员	人	1695	1410	1404	1325	1447
# 执业(助理)医师	人	725	627	618	592	564
注册护师、护士	人	526	422	420	381	413

?1994-2018 China Academic Journal Electronic Publishing House. All rights reserved.　http://www.cnki.net

社会主要指标

2009年	2010年	2011年	2012年	2013年	2014年	2015年	2016年
54.00	54.20	54.35	54.56	54.72	54.87	55.08	55.23
88.34	107.15	125.00	145.20	175.22	182.77	191.70	217.67
13.35	16.48	18.99	21.00	22.74	24.38	24.93	26.04
42.77	53.56	63.99	79.08	100.77	101.49	105.30	120.58
32.21	37.12	42.02	45.12	51.71	56.90	61.48	71.05
39.38	50.09	57.58	71.75	92.46	91.70	94.41	89.35
15633	19354	23032	26664	32069	33355	36962	39465
113.6	113.3	114.6	113.5	114.5	111.1	110.0	108.4
701246	916900	1115300	1427000	1820881	2140478	2614660	3014536
16209	22008	28585	36600	45000	51300	52000	40000
81539	114986	141510	177113	197989	210795	246536	262501
4787	5768	7273	8437	9677	10936	10232	11067
16992	19838	23488	27246	30216	33600	30542	32782
35423	35616	35715	35810	34875	33924	32687	31490
236882	252245	230911	222957	222003	217156	224011	218148
220664	268760	307761	325470	362534	384997	388869	415054
236630	278635	323331	374490	433232	497350	563791	634908
3159	3167	3083	2988	2824	2594	2479	2304
42166	41178	40261	37131	34330	34964	36081	34727
2469	2565	2605	2644	2788	2867	2831	2800
48664	47401	48899	37687	35577	30520	28150	26220
1345	1498	1638	1557	2343	2246	2274	2357
2025	2578	3019	3373	3725	3960	4096	4204
607	626	721	722	810	841	945	963
426	616	687	767	879	1010	1042	1234

?1994-2018 China Academic Journal Electronic Publishing House. All rights reserved. http://www.cnki.net
