# Supplementary material for: A study on the tourism efficiency of tourism destination based on DEA model: A case of ten cities in Shaanxi province
Source: PLoS One. 2024 Jan 19;19(1):e0296660. doi: 10.1371/journal.pone.0296660 (PMC10798521; doi:10.1371/journal.pone.0296660)
Supplement: S1 File — (ZIP) [file pone.0296660.s001.zip › Supporting information/Statistical yearbook/Yan'an.caj]

九、延安市

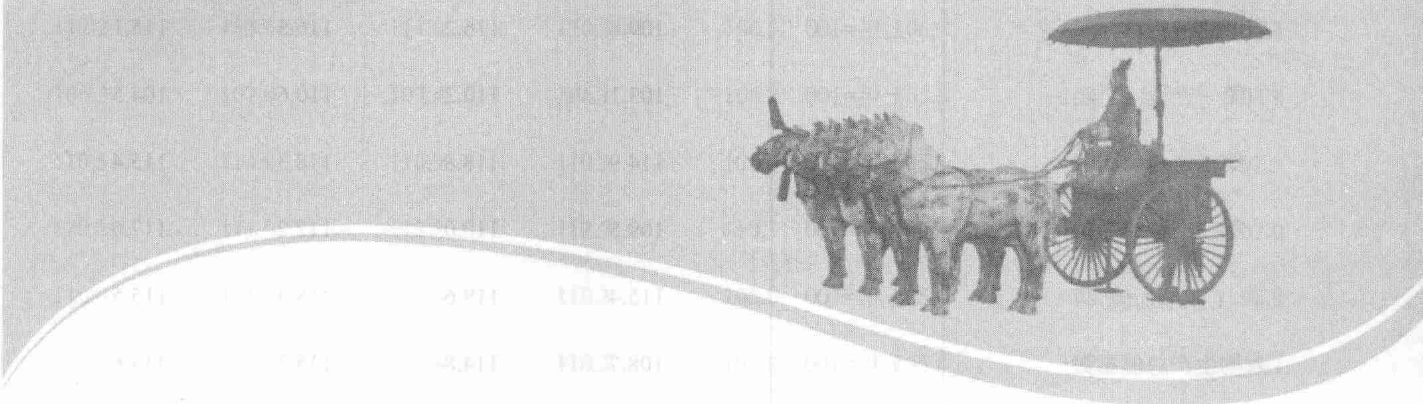

资料整理：胡　狄　张伟琴

?1994-2018 China Academic Journal Electronic Publishing House. All rights reserved.　http://www.cnki.net

9-1 延安市经济

指 标	单 位	2000年	2005年	2006年	2007年	2008年
年底总人口	万人	196.64	211.85	212.02	213.49	214.49
人口自然增长率	‰			4.61	4.62	4.70
年底总户数	万户	50.00	65.28	68.67	71.42	73.76
生产总值	亿元	130.63	394.65	541.86	647.46	760.84
第一产业	亿元	19.13	29.47	34.52	41.32	52.15
第二产业	亿元	78.69	286.90	415.77	498.40	578.20
第三产业	亿元	32.81	78.28	91.57	107.74	130.49
# 工业增加值	亿元	74.65	277.60	404.15	484.02	562.11
人均生产总值	元	6690	18815	25567	30432	35555
生产总值指数	上年=100	109.8	116.2	116.5	115.1	116.3
第一产业	上年=100	103.1	110.2	110.6	104.5	107.1
第二产业	上年=100	114.9	118.8	118.3	115.4	117.0
第三产业	上年=100	109.5	110.0	112.2	117.6	116.6
# 工业增加值	上年=100	115.4	119.6	118.4	115.5	117.2
人均生产总值指数	上年=100	108.7	114.8	115.3	114.6	115.6
非公有制经济增加值	亿元		55.91	65.28	74.56	104.61
文化产业增加值	亿元					
单位GDP能耗	吨标准煤/万元		0.980	0.952	0.907	0.865
单位GDP能耗比上年增长	%			-2.85	-4.78	-4.56
就业人员	万人	91.03	96.88	100.09	101.61	105.93
城镇单位就业人员	万人	18.07	19.66	20.27	21.43	22.31
# 国有单位	万人	16.84	17.89	18.58	19.80	20.52
集体单位	万人	1.04	1.04	1.01	0.95	1.05
# 在岗职工人数	万人	17.30	18.99	19.60	20.73	21.60
城镇单位就业人员平均工资	元					
城镇单位在岗职工平均工资	元	6970	15864	18404	23140	29530

?1994-2018 China Academic Journal Electronic Publishing House. All rights reserved. http://www.cnki.net

社会主要指标

2009年	2010年	2011年	2012年	2013年	2014年	2015年	2016年
217.07	218.87	219.40	219.81	220.61	221.43	223.13	225.28
4.64	4.27	4.27	4.39	4.46	4.34	4.32	4.62
77.55	80.80	81.85	83.59	86.46	85.81	87.27	87.85
728.26	885.42	1113.35	1271.02	1354.14	1386.09	1198.27	1082.91
55.07	71.19	86.69	97.06	105.00	113.70	110.88	117.62
515.89	635.49	815.45	934.85	974.39	968.89	724.79	574.20
157.30	178.74	211.21	239.11	274.75	303.50	362.60	391.09
497.21	614.45	789.45	904.64	941.33	930.77	699.36	539.38
33898	40621	50807	57876	61493	62714	53908	48300
112.2	113.6	111.0	110.5	106.5	106.2	101.7	101.3
106.3	107.0	107.4	106.1	104.3	105.5	104.7	104.8
110.5	114.8	110.9	110.3	105.6	105.3	99.4	97.9
120.2	111.0	112.8	112.7	110.1	109.3	107.5	107.0
110.4	115.0	110.8	110.3	105.5	105.0	98.9	96.9
111.7	112.7	110.4	110.3	106.2	105.8	101.1	100.4
118.71	151.47	201.55	235.25	266.65	292.62	280.77	282.99
					15.35	16.58	18.45
0.826	0.691	0.667	0.644	0.621	0.600	0.577	0.581
-4.54	-5.38	-3.50	-3.50	-3.50	-3.40	-3.80	-3.50
107.79	111.18	115.56	117.71	128.30	134.00	135.28	136.96
22.15	23.11	24.05	24.62	31.79	34.83	33.62	33.65
20.30	21.01	21.95	22.36	15.92	18.51	16.82	16.79
0.95	1.12	1.00	1.02	1.05	0.97	1.09	1.08
20.66	21.48	23.06	23.17	29.50	32.65	30.99	30.92
			46183	51459	53651	56459	58460
34347	38313	42676	47867	52900	55520	58727	60226

?1994-2018 China Academic Journal Electronic Publishing House. All rights reserved. http://www.cnki.net

9-1 续表 1

指 标	单 位	2000年	2005年	2006年	2007年	2008年
全社会固定资产投资	亿元	48.10	160.19	231.67	315.33	406.35
# 房地产开发	亿元	1.28	2.74	5.45	5.22	10.38
商品房销售面积	万平方米	9.39	24.34	11.72	45.13	64.52
# 住宅	万平方米	9.04	22.21	11.27	44.55	57.17
地方财政收入	亿元	8.86	45.08	60.37	72.69	80.05
地方财政支出	亿元	16.47	56.36	83.09	101.07	130.52
金融机构人民币各项存款余额	亿元	97.61	301.99	395.15	442.05	545.75
金融机构人民币各项贷款余额	亿元	84.91	177.46	201.03	219.82	245.23
农村居民人均纯收入	元	1444	2195	2461	2845	3551
城镇居民人均可支配收入	元	5083	7471	8500	9801	12232
城市人均公园绿地面积	平方米		4.4	4.7	8.0	8.5
城市人均道路面积	平方米		4.2	4.4	4.7	4.8
城市用水普及率	%		39.7	66.0	66.6	85.0
城市燃气普及率	%		90.1	92.1	89.5	92.4
常用耕地面积	千公顷	256.45	233.64	231.86	231.36	231.16
农林牧渔业总产值	亿元	35.23	51.99	57.44	70.41	90.86
农作物总播种面积	千公顷	322.25	270.16	211.31	208.17	251.81
# 粮食作物	千公顷	278.30	217.14	216.26	161.28	203.94
粮食产量	万吨	66.60	74.49	75.93	50.46	70.86
棉花产量	吨	110	191	245	489	411
油料产量	吨	16123	19376	17909	16700	16965
蔬菜产量	吨	121194	500989	519766	551495	611565

?1994-2018 China Academic Journal Electronic Publishing House. All rights reserved. http://www.cnki.net

	2009年	2010年	2011年	2012年	2013年	2014年	2015年	2016年
	557.48	724.53	815.21	1032.06	1321.04	1541.07	1637.17	1359.33
	8.18	8.13	8.08	20.31	18.40	26.66	50.98	77.84
	38.71	20.28	23.98	41.51	52.16	58.43	55.58	66.57
	38.71	20.19	21.37	39.68	51.35	55.28	52.51	64.73
	90.47	105.19	120.73	139.26	155.38	168.10	161.17	130.55
	156.93	192.71	223.82	264.24	294.68	310.41	315.60	327.34
	645.36	728.54	826.26	987.21	1103.83	1231.77	1360.00	1451.53
	302.05	360.97	410.49	476.84	566.84	679.61	828.86	925.39
	4258	5173	6565	7655	8681	8955	9789	10568
	15217	17880	21188	24748	27643	26399	28590	30693
	9.6	9.6	9.6	9.6	9.8	12.0	10.4	10.7
	4.9	5.1	5.1	4.8	5.4	7.0	7.2	7.8
	85.3	86.0	86.4	86.9	87.9	81.2	89.7	84.9
	94.0	94.0	94.0	94.0	95.0	99.6	95.5	98.8
	233.51	234.57	235.29	240.42	240.55	244.87	247.15	245.93
	96.41	125.16	152.29	169.69	187.81	203.20	197.63	209.77
	257.00	252.57	239.42	241.65	245.52	244.58	247.93	250.32
	208.98	209.56	196.25	197.84	199.39	200.40	201.85	202.52
	77.10	82.04	68.47	76.58	74.35	78.68	72.21	78.01
	483	484	514	919	875	931	839	834
	23043	23042	23000	21844	21149	22701	21386	23610
	710075	881386	930065	1000274	1060400	1130000	1210374	1299300

?1994-2018 China Academic Journal Electronic Publishing House. All rights reserved. http://www.cnki.net

9-1　续表 2

指　　标	单 位	2000年	2005年	2006年	2007年	2008年
水果产量	吨	467899	1014649	1225550	1433105	1724977
# 苹果	吨	436758	952877	1178175	1380908	1642816
肉类产量	吨	46384	62793	31043	29315	36114
# 猪牛羊肉	吨	41975	56514	27259	25602	31096
奶类产量	吨	1909	5491	5850	6290	6535
# 牛奶	吨	771	3848	3881	4242	4600
禽蛋产量	吨	13683	16884	15934	17256	19514
水产品产量	吨	1693	2119	2197	2439	1810
规模以上工业企业单位数	个	122	92	94	67	95
规模以上工业总产值	亿元	131.00	537.11	765.24	905.20	1085.33
天然原油产量	万吨	213.56	1058.57	1180.32	1333.37	1410.85
发电量	亿千瓦小时	3.39	3.08	3.12	2.94	5.37
钢材产量	万吨	0.56	1.26	1.26		
水泥产量	万吨	6.78	3.96		12.03	15.67
建筑业企业单位数	个	34	44	44	46	63
建筑业企业年末从业人员	万人	0.78	1.45	1.34	1.17	1.96
建筑业总产值	亿元	5.13	16.35	19.80	28.30	41.10
房屋建筑施工面积	万平方米	118.35	204.95	268.87	255.55	378.17
房屋建筑竣工面积	万平方米	36.20	115.18	100.10	123.92	219.33
公路里程	公里	4499	5465	13842	12602	13311
# 等级公路	公里	3049	5061	8625	10604	11969
# 高速公路	公里		58	235	235	235
民用汽车拥有量	辆	22851	49599	100969	115258	137479
# 私人汽车	辆	9581	22104	80898	98079	101208
邮电业务总量	亿元	3.93	21.38	27.74	36.86	44.49
邮政业务总量	亿元	0.35	0.88	1.06	1.20	1.25
电信业务总量	亿元	3.59	20.50	26.68	35.66	43.23

?1994-2018 China Academic Journal Electronic Publishing House. All rights reserved. http://www.cnki.net

2009年	2010年	2011年	2012年	2013年	2014年	2015年	2016年
2069785	2332987	2565509	2711394	2540347	2702089	2861916	3191242
1969101	2215184	2438139	2600206	2440059	2614373	2734600	3031843
54419	61937	68355	71154	74182	76254	76135	75793
48198	55760	60930	64154	66736	68829	67998	67692
6553	7098	7563	7335	7583	7379	7408	7213
4873	5277	5427	5492	5733	5471	5451	5339
21961	23969	25933	26693	27932	27782	28737	29379
1978	2595	2705	2908	2928	3030	3035	3052
134	122	91	113	115	129	140	144
969.50	1227.31	1504.86	1646.76	1612.32	1708.57	1417.24	1090.42
1514.06	1602.12	1623.14	1685.70	1691.02	1716.44	1675.15	1514.27
5.82	4.83	5.00	7.54	7.32	7.15	5.60	38.46
12.05	9.40	69.87	75.98	90.12	122.17	124.36	142.53
63	63	97	111	116	131	147	154
2.16	2.01	2.26	2.37	2.48	4.57	4.70	5.59
44.00	53.95	68.44	84.10	84.88	101.16	104.87	116.79
324.05	342.88	410.53	503.40	506.44	659.02	583.35	686.18
158.91	102.55	184.67	118.47	132.49	176.20	191.56	202.17
14337	14926	15727	16756	17057	17110	17621	17805
13610	14343	15129	15950	16247	16277	16572	16823
235	427	427	427	537	537	716	802
128002	179370	197714	215297	231446	246119	264708	284879
70009	144707	160665	176661	193852	208855	228274	250444
44.12	17.26	22.34	23.12	25.27	34.52	45.94	64.18
1.42	1.22	1.66	1.03	1.26	1.57	1.77	2.47
42.70	16.04	20.68	22.09	24.01	32.95	44.17	61.71

?1994-2018 China Academic Journal Electronic Publishing House. All rights reserved. http://www.cnki.net

9-1 续表 3

指　标	单 位	2000年	2005年	2006年	2007年	2008年
固定电话用户	万户	15.38	30.16	34.02	35.17	33.69
移动电话用户	万户	8.01	84.59	116.86	150.96	203.15
互联网宽带用户	万户	0.59	7.44	10.65	14.64	20.54
限额以上企业数	个					
批发业	个					
零售业	个					
住宿业	个					
餐饮业	个					
社会消费品零售总额	亿元	22.63	47.53	54.70	65.85	84.80
进出口总额	万美元		1295	1309	2115	2532
# 出口	万美元		1236	1273	2093	2531
实际外商直接投资额	万美元			43	20	272
入境旅游人数	万人次	0.40	1.53	1.67	2.23	3.60
# 外国人	万人次	0.13	0.76	0.81	0.61	1.30
国际旅游外汇收入	万美元	52	151	162	220	279
国内旅游人数	万人次	184	508	563	650	742
国内旅游收入	亿元	4.12	20.20	25.49	35.01	42.84
星级饭店数	个		18	18	26	26
幼儿园数	所	155	198	216	233	269
在园儿童数	万人	3.57	4.02	4.03	4.31	4.74
普通小学学校数	所	4707	2400	2192	1614	1302
普通小学专任教师数	人	14189	14931	14594	14065	14018
普通小学在校学生数	万人	36.16	23.52	22.36	21.33	20.02
普通中学学校数	所	180	180	183	173	160
普通中学专任教师数	人	6805	10709	11154	11504	11546
普通中学在校学生数	万人	12.75	21.03	21.39	20.78	19.52
卫生机构数	个	367	397	407	363	341
卫生机构床位数	张	5249	6385	6855	7190	7121
卫生技术人员	人	7206	8368	8395	8722	9192
# 执业(助理)医师	人	3543	3738	3528	3646	3638
注册护师、护士	人	1718	2173	2175	2277	2552

?1994-2018 China Academic Journal Electronic Publishing House. All rights reserved. http://www.cnki.net

2009年	2010年	2011年	2012年	2013年	2014年	2015年	2016年
30.13	47.81	38.69	38.59	36.81	34.70	33.84	33.10
206.20	212.54	201.78	216.46	201.96	213.75	219.27	228.15
16.70	16.59	20.10	20.87	21.36	23.36	25.17	37.27
101	140	169	194	233	329	370	428
12	15	15	22	28	70	77	86
33	60	84	95	119	155	174	212
36	39	35	42	48	59	71	78
20	26	35	35	38	45	48	52
96.86	117.06	140.48	168.30	192.28	218.24	241.09	257.95
2241	2247	3684	6623	9598	7.14(亿元)	6.84(亿元)	1.46(亿元)
2224	2241	3684	6623	9598	7.14(亿元)	6.84(亿元)	1.17(亿元)
174	1027	1566	2000	802	1063		840
5.05	8.54	12.01	12.71	6.90	4.92	3.52	3.46
1.47	6.06	7.75	4.48	0.48	1.83	1.50	2.02
364	659	909	975	671	555	406	375
1019	1442	2038	2177	2841	3146	3501	4025
53.63	76.08	109.43	117.38	151.49	171.75	192.23	227.74
33	34	34	42	42	48	51	49
304	349	428	467	523	559	583	572
5.52	6.36	8.52	10.45	11.43	12.35	12.86	12.85
591	386	346	325	319	307	301	299
13510	13537	12511	12210	12034	11975	11504	11713
18.94	18.90	18.00	17.30	17.20	17.55	18.63	19.65
152	139	130	129	123	121	120	120
11143	10736	10806	10279	10443	10216	9446	10066
18.03	16.71	15.91	14.41	13.59	13.12	12.73	12.75
424	463	3419	3447	3541	3571	3244	3151
7790	8519	8962	10119	11091	11971	12865	13384
9766	10252	11286	12171	13391	13835	15214	16030
3852	4017	3648	3989	4402	4269	4466	4803
2891	3175	3759	4384	4958	5351	6203	6894

?1994-2018 China Academic Journal Electronic Publishing House. All rights reserved. http://www.cnki.net

9-2 宝塔区经济

指　　标	单　位	2000年	2005年	2006年	2007年	2008年
年底总人口	万人	34.04	40.55	40.59	40.98	41.21
生产总值	亿元	21.45	78.54	108.69	129.31	135.07
第一产业	亿元	2.20	3.34	3.81	4.54	5.86
第二产业	亿元	11.92	46.97	71.45	83.53	79.97
第三产业	亿元	7.34	28.24	33.43	41.25	49.24
# 工业增加值	亿元	9.92	43.66	66.83	77.95	73.40
人均生产总值	元	6356	19568	26791	31702	32863
生产总值指数	上年=100	116.8	115.9	119.2	112.1	113.6
全社会固定资产投资	万元	130412	305353	441357	500212	956043
地方财政收入	万元	10455	38990	48801	65595	70977
地方财政支出	万元	14626	44277	55828	72612	101824
农村居民人均纯收入	元	1487	2177	2381	2622	3458
城镇居民人均可支配收入	元	5083	7471	9109	10580	12748
常用耕地面积	公顷	32042	34078	33034	32547	32280
粮食产量	吨	86061	94336	60984	57539	88115
农林牧渔业总产值	万元	36098	54575	59268	72896	96301
社会消费品零售总额	万元	78021	202186	239517	293540	384611
普通小学专任教师数	人	2502	2980	3064	2921	2894
普通小学在校学生数	人	68558	51767	49762	46625	44639
普通中学专任教师数	人	1359	2448	2740	2850	2808
普通中学在校学生数	人	25776	50397	52488	52674	50293
卫生机构床位数	张	1631	2425	2689	2910	2789
卫生技术人员	人	1901	2975	2594	3216	2966
# 执业(助理)医师	人	899	1247	1098	1327	1453
注册护师、护士	人					

?1994-2018 China Academic Journal Electronic Publishing House. All rights reserved.　http://www.cnki.net

社会主要指标

2009年	2010年	2011年	2012年	2013年	2014年	2015年	2016年
46.44	47.57	47.65	47.69	47.85	48.15	48.17	48.93
125.18	146.38	172.83	203.31	214.91	241.33	273.78	255.14
5.91	8.08	10.05	10.70	11.20	12.21	12.28	12.84
60.28	66.33	76.54	94.81	98.05	109.54	108.60	82.58
58.99	71.97	86.24	97.80	105.65	119.57	152.90	159.72
52.79	57.86	65.89	82.19	81.42	91.29	105.25	71.47
30334	31143	36303	42650	45336	50277	56848	52551
110.2	113.2	111.4	113.1	106.4	110.2	106.0	101.9
1054746	1372678	1555031	2021413	2629433	3026712	3208315	3663213
76015	85312	94053	106436	117871	131976	143825	108050
110881	121655	145627	182449	216626	281407	249721	286122
4157	5051	6379	7483	8493	9529	9090	9835
15771	18486	21906	25520	28378	31358	29777	32039
32130	32221	32152	32450	32382	32431	32360	31201
90842	96850	82648	90198	85023	89917	88917	90011
98796	134951	166710	184619	198094	214744	202553	214136
430075	509042	601629	697014	881976	1014119	1121350	1173856
2830	2888	2624	2702	2631	2753	2816	2991
43496	40760	42913	44803	48836	52960	57839	61470
2768	2789	2684	2669	2720	2678	2815	2994
46918	42127	41206	40972	41052	40427	40759	41524
3093	3558	3787	4636	4901	5103	5776	5837
3741	4014	4671	5519	5901	6026	6622	6786
1508	1686	1685	1854	2034	1984	2138	2249
1304	1417	1732	2279	2494	2627	3091	3269

?1994-2018 China Academic Journal Electronic Publishing House. All rights reserved. http://www.cnki.net

9-3 安塞区经济

指　　标	单　位	2000年	2005年	2006年	2007年	2008年
年底总人口	万人	15.10	16.56	16.57	16.62	16.70
生产总值	亿元	14.16	38.48	53.84	66.06	82.45
第一产业	亿元	1.56	2.52	2.59	3.08	3.49
第二产业	亿元	10.53	31.72	46.31	57.17	71.88
第三产业	亿元	2.07	4.24	4.93	5.81	7.09
# 工业增加值	亿元	10.44	31.25	45.83	56.59	71.18
人均生产总值	元	9308	23610	32491	39796	49491
生产总值指数	上年=100	100.7	108.0	115.2	120.2	116.1
全社会固定资产投资	万元	12709	140169	122824	157460	193697
地方财政收入	万元	11656	36147	57591	66824	70886
地方财政支出	万元	13578	46360	60886	75735	80669
农村居民人均纯收入	元	1706	2399	2868	3295	3904
城镇居民人均可支配收入	元				10170	12926
常用耕地面积	公顷	28279	25800	25800	25800	25800
粮食产量	吨	59048	68307	46169	43479	62515
农林牧渔业总产值	万元	29187	43586	44879	53774	62305
社会消费品零售总额	万元	10948	25112	29124	33864	42718
普通小学专任教师数	人	734	1075	1070	1074	915
普通小学在校学生数	人	29506	18324	15833	14915	13543
普通中学专任教师数	人	561	773	781	772	799
普通中学在校学生数	人	10635	14201	13795	12144	11767
卫生机构床位数	张	294	359	354	345	436
卫生技术人员	人	391	477	440	467	450
# 执业(助理)医师	人	203	217	182	192	173
注册护师、护士	人					

?1994-2018 China Academic Journal Electronic Publishing House. All rights reserved.　http://www.cnki.net

社会主要指标

2009年	2010年	2011年	2012年	2013年	2014年	2015年	2016年
17.09	17.17	17.24	17.29	17.38	17.43	17.54	17.71
64.12	72.24	94.76	105.75	104.45	106.44	96.68	76.66
3.60	4.28	5.17	6.48	7.00	7.32	7.31	8.07
51.66	58.36	78.59	86.90	82.16	82.18	69.19	46.76
8.86	9.60	11.00	12.37	15.29	16.94	20.18	21.83
50.83	57.35	77.33	85.43	80.81	80.29	67.08	44.60
38286	99081	55077	61251	60178	61155	55276	43501
114.0	114.2	115.5	111.6	106.7	106.2	106.5	96.5
294862	457436	602069	829918	1133312	1350074	1466676	1191196
73366	77830	90067	107262	123673	142521	144319	106123
100549	121954	134439	170488	197082	209096	204795	187270
4646	5562	6936	8046	9148	10374	10437	11271
16223	19208	22706	26680	29695	32852	29801	31946
27200	27200	27200	27200	27200	27200	27260	27260
65106	71319	59918	68568	65687	71490	69521	68243
65058	77578	92326	113293	125010	131228	131628	147381
47660	56338	65646	76279	97133	108438	119499	124557
827	1031	1046	995	862	891	843	846
13699	13995	13819	13918	13980	14168	14784	15327
771	830	730	700	662	642	644	648
11556	11359	10615	9532	8735	8633	8486	8877
478	481	535	566	646	683	634	645
585	605	577	630	730	875	885	934
206	219	184	175	218	233	224	232
169	175	182	195	254	326	351	382

?1994-2018 China Academic Journal Electronic Publishing House. All rights reserved.   http://www.cnki.net

9-4 延长县经济

指　　标	单　位	2000年	2005年	2006年	2007年	2008年
年底总人口	万人	14.20	14.41	14.42	14.42	14.47
生产总值	亿元	4.40	12.12	17.53	20.10	23.88
第一产业	亿元	1.15	1.80	2.07	2.44	3.24
第二产业	亿元	1.69	6.88	11.35	12.81	14.95
第三产业	亿元	1.56	3.44	4.11	4.86	5.68
# 工业增加值	亿元	1.61	6.57	11.05	12.39	14.43
人均生产总值	元	3097	8411	12158	13939	16524
生产总值指数	上年=100	106.5	117.2	117.1	111.0	112.4
全社会固定资产投资	万元	4493	75330	40863	61437	131437
地方财政收入	万元	1084	10495	16910	18918	21800
地方财政支出	万元	6025	23072	32869	36019	48530
农村居民人均纯收入	元	956	1666	1844	2199	2904
城镇居民人均可支配收入	元				8810	11303
常用耕地面积	公顷	17925	17077	16413	16237	16237
粮食产量	吨	33287	31655	20200	21431	34337
农林牧渔业总产值	万元	17909	31921	34577	42162	57719
社会消费品零售总额	万元	10074	18457	20231	24001	30677
普通小学专任教师数	人	913	675	688	679	675
普通小学在校学生数	人	26315	10687	10470	10206	9768
普通中学专任教师数	人	453	653	656	664	620
普通中学在校学生数	人	9112	12572	12230	11581	9767
卫生机构床位数	张	260	219	219	209	222
卫生技术人员	人	371	442	379	381	354
# 执业(助理)医师	人	185	171	147	169	152
注册护师、护士	人					

?1994-2018 China Academic Journal Electronic Publishing House. All rights reserved. http://www.cnki.net

社会主要指标

2009年	2010年	2011年	2012年	2013年	2014年	2015年	2016年
12.60	12.55	12.58	12.60	12.61	12.64	12.78	12.81
22.31	25.48	29.60	34.09	36.28	39.48	43.29	37.83
3.35	4.79	5.89	7.31	8.02	8.69	8.79	8.80
11.95	12.83	13.99	15.86	17.59	19.24	21.43	14.42
7.01	7.86	9.72	10.92	10.68	11.55	13.07	14.61
11.34	12.17	13.19	14.95	16.52	18.07	20.16	13.10
15385	20260	23555	27075	29885	31271	34057	29562
110.1	113.3	111.3	113.0	112.1	107.9	108.3	99.3
173115	226308	263890	344507	451605	530201	576617	458395
23330	25686	28259	30543	33130	34562	32700	24385
56058	78767	90619	104224	117890	136330	137408	153477
3473	4258	5378	6282	7124	8007	8671	9286
13920	16333	19305	22529	25007	27615	27076	29106
16181	16510.2	16510	16510	16530	16570	16570	14867
36336	38742	33263	38374	35598	40077	27832	27047
59866	85331	104211	128152	141856	153645	152321	147698
34198	40353	46899	54168	68369	76493	84372	89109
687	682	638	633	621	559	324	393
9361	8850	8404	7426	6112	6425	7284	7739
554	523	539	495	413	393	314	339
8559	7290	7183	5244	4660	4225	4274	4623
248	289	322	327	333	464	432	541
377	374	414	428	494	514	545	705
156	151	102	121	131	118	150	185
103	108	120	122	142	191	182	248

?1994-2018 China Academic Journal Electronic Publishing House. All rights reserved. http://www.cnki.net

9-5 延川县经济

指标	单位	2000年	2005年	2006年	2007年	2008年
年底总人口	万人	18.03	18.36	18.37	18.51	18.60
生产总值	亿元	10.65	37.13	44.04	45.32	51.67
第一产业	亿元	0.91	1.47	1.60	1.88	2.74
第二产业	亿元	7.79	31.22	37.37	37.32	41.82
第三产业	亿元	1.94	4.44	5.07	6.12	7.12
# 工业增加值	亿元	6.67	29.71	35.64	35.08	38.93
人均生产总值	元	6066	20366	23973	24577	27840
生产总值指数	上年=100	118.7	106.4	109.7	106.9	112.6
全社会固定资产投资	万元	127539	425193	515423	719538	68066
地方财政收入	万元	2621	11456	13910	15316	16236
地方财政支出	万元	8440	26258	33318	44568	56671
农村居民人均纯收入	元	748	1503	1805	2012	2718
城镇居民人均可支配收入	元				7474	10248
常用耕地面积	公顷	15618	24417	24417	24477	24714
粮食产量	吨	18165	40330	32919	32153	42684
农林牧渔业总产值	万元	15239	23697	26375	32152	47564
社会消费品零售总额	万元	8827	23863	25742	30087	38271
普通小学专任教师数	人	1198	1088	1023	964	1048
普通小学在校学生数	人	32556	19456	17356	15935	15023
普通中学专任教师数	人	525	787	807	773	772
普通中学在校学生数	人	9360	15640	26026	15210	14260
卫生机构床位数	张	353	340	358	379	390
卫生技术人员	人	467	568	544	543	525
# 执业(助理)医师	人	240	270	255	241	235
注册护师、护士	人					

?1994-2018 China Academic Journal Electronic Publishing House. All rights reserved. http://www.cnki.net

社会主要指标

2009年	2010年	2011年	2012年	2013年	2014年	2015年	2016年
16.85	16.85	16.89	16.93	16.97	17.01	17.03	17.10
52.53	65.05	81.26	82.38	91.95	97.69	69.49	68.11
2.94	3.54	4.28	4.80	5.35	5.94	6.73	7.55
41.00	52.06	65.74	64.80	71.53	74.95	43.95	39.13
8.59	9.45	11.24	12.78	15.07	16.81	18.81	21.43
37.58	48.39	61.32	59.88	69.49	72.19	40.98	36.03
28189	38612	48165	48714	55785	57499	40828	39912
106.5	106.0	107.5	96.3	102.7	110.6	88.5	108.7
84529	110034	120772	167027	237162	346565	469079	633618
17376	18420	19200	20224	42799	34133	30810	42864
64251	83300	103900	131695	139296	181568	188311	191282
3278	4002	4970	5815	6530	7634	8354	9063
12933	15183	17977	20961	23497	26089	26212	28072
24712	24712	24838	24838	24805	24805	24138	23684
45723	45917	40212	43263	40054	43018	26797	31767
51926	62222	74289	83862	97488	109300	118827	133672
42618	50059	58047	66966	84996	95486	106093	118251
1048	959	927	709	762	772	552	569
13648	13158	12600	12328	11904	10591	10461	10469
722	713	649	653	653	611	450	423
12614	11872	10679	8470	8232	7985	6901	6175
425	425	438	349	504	554	580	722
526	504	527	390	462	479	537	579
238	226	180	152	147	156	169	198
125	123	156	117	159	180	194	233

?1994-2018 China Academic Journal Electronic Publishing House. All rights reserved. http://www.cnki.net

9-6 子长县经济

指　标	单　位	2000年	2005年	2006年	2007年	2008年
年底总人口	万人	22.92	24.65	24.67	25.14	25.25
生产总值	亿元	8.06	21.11	30.56	34.63	43.89
第一产业	亿元	2.07	2.60	2.90	3.41	4.64
第二产业	亿元	3.91	12.50	20.50	23.12	29.57
第三产业	亿元	2.07	6.01	7.16	8.10	9.68
# 工业增加值	亿元	3.69	12.16	20.11	22.61	28.98
人均生产总值	元	3524	8719	12393	13902	17417
生产总值指数	上年=100	110.0	117.9	121.2	112.9	115.4
全社会固定资产投资	万元	16112	68854	82416	132796	321078
地方财政收入	万元	5139	25555	27468	35032	39040
地方财政支出	万元	9747	37680	46515	56381	91363
农村居民人均纯收入	元	1219	1948	2249	2519	3368
城镇居民人均可支配收入	元		5398	7915	9996	12895
常用耕地面积	公顷	29362	28413	28612	28737	28968
粮食产量	吨	76418	77318	56811	56749	75877
农林牧渔业总产值	万元	34503	43156	46984	57164	78410
社会消费品零售总额	万元	19186	32241	36018	43382	55199
普通小学专任教师数	人	1251	1469	1427	1434	1493
普通小学在校学生数	人	43411	27059	26271	25581	23695
普通中学专任教师数	人	578	940	947	948	1037
普通中学在校学生数	人	13965	20542	20628	20965	18360
卫生机构床位数	张	333	337	423	450	478
卫生技术人员	人	463	505	533	587	632
# 执业(助理)医师	人	345	239	226	288	274
注册护师、护士	人					

?1994-2018 China Academic Journal Electronic Publishing House. All rights reserved. http://www.cnki.net

社会主要指标

2009年	2010年	2011年	2012年	2013年	2014年	2015年	2016年
21.66	21.71	21.76	21.80	21.72	21.76	21.88	22.00
50.54	59.44	68.93	74.56	81.61	79.75	74.50	74.97
4.71	5.49	6.47	6.61	7.34	7.20	6.35	6.99
34.51	41.86	48.57	52.23	57.87	53.72	45.15	42.07
11.33	12.09	13.89	15.72	16.70	18.83	23.00	25.91
33.82	41.10	47.66	51.23	57.02	52.46	43.78	40.65
19971	27412	31714	34234	38205	36684	34145	34172
114.1	113.2	108.8	112.7	115.5	106.2	104.5	102.0
398664	519338	645574	764434	980242	1161298	1221006	1101865
70416	84199	92697	64380	70146	65998	44837	39368
127255	136472	153731	159055	168409	157032	160224	182394
4129	5050	6277	7357	8357	9335	9021	9752
16119	18988	22669	26387	29395	32570	28679	30772
29145	29618	29994	30370	30834	31263	31521	31567
80432	82239	67727	77259	75025	77657	31848	75785
80294	93722	111102	114146	125566	128972	115647	128305
61585	72669	85016	98050	124394	139086	153968	170615
1441	1456	1401	1388	1271	1165	1161	1110
20532	19746	19781	19842	18140	17340	17845	18505
1009	949	998	1011	1114	1096	1002	908
16435	15682	15219	15071	13338	12897	12527	12656
511	553	585	655	889	948	993	1020
693	799	839	827	956	989	1095	1247
268	315	255	284	344	362	301	332
222	218	258	265	276	305	328	506

?1994-2018 China Academic Journal Electronic Publishing House. All rights reserved. http://www.cnki.net

9-7 志丹县经济

指 标	单 位	2000年	2005年	2006年	2007年	2008年
年底总人口	万人	11.80	13.44	13.45	13.54	13.61
生产总值	亿元	14.36	69.05	106.77	129.65	151.08
第一产业	亿元	1.38	1.80	2.20	2.59	3.01
第二产业	亿元	11.24	61.57	97.54	118.93	138.13
第三产业	亿元	1.74	5.68	7.03	8.14	9.94
# 工业增加值	亿元	10.82	60.96	96.78	118.03	137.08
人均生产总值	元	12189	52268	79385	96038	111249
生产总值指数	上年=100	112.7	135.9	127.3	118.2	125.2
全社会固定资产投资	万元	28228	108341	148643	219175	459430
地方财政收入	万元	11116	76557	95850	113642	124498
地方财政支出	万元	13319	57267	88416	124045	146824
农村居民人均纯收入	元	1398	2270	2392	2796	3447
城镇居民人均可支配收入	元	4489	7535	8650	10147	12796
常用耕地面积	公顷	24680	20019	20161	20338	20426
粮食产量	吨	49605	56884	39456	36531	54322
农林牧渔业总产值	万元	23400	28190	33308	38878	45799
社会消费品零售总额	万元	13653	24361	27974	33957	43218
普通小学专任教师数	人	705	993	1018	998	1040
普通小学在校学生数	人	22607	17499	16645	15868	15237
普通中学专任教师数	人	274	482	551	591	528
普通中学在校学生数	人	5838	11935	12315	11782	10227
卫生机构床位数	张	211	342	374	391	414
卫生技术人员	人	319	416	406	477	496
# 执业(助理)医师	人	162	162	151	156	164
注册护师、护士	人					

?1994-2018 China Academic Journal Electronic Publishing House. All rights reserved. http://www.cnki.net

社会主要指标

2009年	2010年	2011年	2012年	2013年	2014年	2015年	2016年
13.92	14.06	14.11	14.16	14.24	14.31	14.48	14.66
121.08	138.63	167.28	175.97	168.07	166.62	132.21	107.59
3.05	3.83	4.36	4.45	4.71	5.02	5.03	5.14
105.92	121.87	148.09	154.76	143.51	140.13	103.22	76.08
12.11	12.93	14.82	16.76	19.85	21.48	23.95	26.37
104.67	120.41	146.26	152.57	141.67	137.54	100.19	72.75
88729	99081	118741	124496	118233	116722	91874	73841
114.5	114	111.8	110.1	105.6	105.6	98.9	101.7
569417	758848	993582	1292650	1736502	2058818	2177816	872237
140112	154516	177127	200275	225084	254706	231913	165304
150650	170746	207264	235356	265512	283425	275173	218000
4203	5116	6792	7872	8872	10001	9971	10688
16110	18978	22678	26670	30004	33403	29547	31585
20400	21248	21252	21200	21197	24417	27547	30187
55913	56027	46012	55146	51455	53180	54024	56488
47300	59115	69157	72235	80143	85421	93086	101813
48318	57106	67009	77914	99128	111056	122938	133322
944	914	871	812	802	804	815	794
13766	12806	12251	12315	12912	13297	14370	15268
503	508	473	457	445	437	424	431
9262	8445	7977	7024	6515	6681	6659	6383
403	464	503	563	663	744	744	749
555	601	681	677	824	817	996	999
187	189	152	184	216	166	195	270
145	192	229	239	302	319	404	407

?1994-2018 China Academic Journal Electronic Publishing House. All rights reserved. http://www.cnki.net

9-8 吴起县经济

指　　标	单　位	2000年	2005年	2006年	2007年	2008年
年底总人口	万人	12.10	12.30	12.31	12.35	12.40
生产总值	亿元	6.48	38.44	55.92	68.48	84.67
第一产业	亿元	1.46	2.41	2.61	3.03	3.35
第二产业	亿元	2.88	31.28	47.56	58.50	72.91
第三产业	亿元	2.14	4.75	5.75	6.94	8.41
# 工业增加值	亿元	2.61	30.67	46.73	57.49	71.74
人均生产总值	元	5379	31050	45390	55539	68393
生产总值指数	上年=100	109.1	116.0	124.5	121.1	122.9
全社会固定资产投资	万元	26153	201782	299293	460306	531586
地方财政收入	万元	4725	73408	122905	136888	148480
地方财政支出	万元	7632	72080	115764	146404	146618
农村居民人均纯收入	元	1396	2086	2363	2745	3619
城镇居民人均可支配收入	元				10380	13006
常用耕地面积	公顷	20000	20090	20090	20090	20262
粮食产量	吨	51949	51089	33040	37386	32773
农林牧渔业总产值	万元	24477	40932	41704	48989	56990
社会消费品零售总额	万元	11143	18227	19841	23688	29724
普通小学专任教师数	人	635	619	672	687	776
普通小学在校学生数	人	21787	14291	13936	13388	12328
普通中学专任教师数	人	333	674	717	812	822
普通中学在校学生数	人	6764	13859	14493	14127	14082
卫生机构床位数	张	204	316	426	442	422
卫生技术人员	人	273	317	399	445	475
# 执业(助理)医师	人	137	170	108	168	160
注册护师、护士	人					

?1994-2018 China Academic Journal Electronic Publishing House. All rights reserved. http://www.cnki.net

社会主要指标

2009年	2010年	2011年	2012年	2013年	2014年	2015年	2016年
14.21	14.52	14.54	14.56	14.70	14.76	14.96	15.15
82.87	120.93	171.61	201.07	208.80	211.51	137.27	108.65
3.37	3.56	4.03	4.29	4.64	4.94	4.89	4.71
69.24	106.38	154.66	182.11	187.18	187.65	110.73	81.65
10.26	10.99	12.92	14.67	16.98	18.92	21.65	22.29
67.87	104.84	152.79	179.86	185.90	185.07	107.87	78.35
66715	84178	118128	138193	143333	142591	92374	72168
114.9	118.1	114.5	110.2	105.9	104.1	101.7	100.2
646426	841570	1067603	1400536	1825088	2198550	2243489	995371
160717	181108	207388	263388	311389	355688	288168	175888
185612	210513	234699	281933	333299	371574	316301	248158
4415	5298	6878	8005	9110	10358	10302	11064
16361	19322	22781	26744	29873	33198	30168	32218
20262	20262	20263	24520	24881	25066	25327	25320
51036	58877	49699	58662	56742	62146	61065	62318
57373	60037	68180	70880	79048	83532	87264	84473
33201	39237	46024	53167	67763	75889	83554	97018
785	748	833	845	897	836	823	825
12178	11834	11638	11491	11638	12098	12545	13165
856	675	546	565	558	563	563	563
13289	12388	11256	8959	8259	7845	7326	7230
418	398	385	415	415	580	627	694
567	578	567	603	690	731	823	923
177	192	151	174	202	212	220	231
145	157	170	193	244	257	311	388

?1994-2018 China Academic Journal Electronic Publishing House. All rights reserved. http://www.cnki.net

9-9 甘泉县经济

指　　标	单　位	2000年	2005年	2006年	2007年	2008年
年底总人口	万人	7.46	7.91	7.92	7.98	8.02
生产总值	亿元	3.30	10.63	13.06	15.78	19.38
第一产业	亿元	0.75	1.19	1.31	1.57	2.06
第二产业	亿元	1.16	6.68	8.45	10.38	13.24
第三产业	亿元	1.39	2.76	3.30	3.84	4.08
# 工业增加值	亿元	1.05	6.37	8.20	10.09	12.90
人均生产总值	元	4460	13524	16489	19845	24225
生产总值指数	上年=100	114.5	114.3	110.2	112.3	116.1
全社会固定资产投资	万元	26044	80488	69862	90063	108461
地方财政收入	万元	2398	18234	24818	29080	32602
地方财政支出	万元	4943	24032	30734	35456	41676
农村居民人均纯收入	元	1678	2604	2829	3136	3671
城镇居民人均可支配收入	元		6720	7639	9300	11579
常用耕地面积	公顷	10754	9314	9294	9292	9485
粮食产量	吨	26172	32061	22869	26406	35008
农林牧渔业总产值	万元	12209	20122	21202	26372	35578
社会消费品零售总额	万元	6612	12383	13695	16102	20624
普通小学专任教师数	人	686	745	714	669	647
普通小学在校学生数	人	13647	8044	7533	6830	6445
普通中学专任教师数	人	219	438	457	483	466
普通中学在校学生数	人	4471	7582	7533	6867	6167
卫生机构床位数	张	155	184	205	209	213
卫生技术人员	人	248	337	339	350	346
# 执业(助理)医师	人	125	133	105	120	118
注册护师、护士	人					

?1994-2018 China Academic Journal Electronic Publishing House. All rights reserved.　http://www.cnki.net

社会主要指标

2009年	2010年	2011年	2012年	2013年	2014年	2015年	2016年
7.74	7.73	7.75	7.78	7.82	7.84	7.88	7.93
18.08	20.60	18.31	20.50	22.02	22.65	21.77	20.59
2.14	2.58	3.05	3.63	3.98	4.22	4.21	4.42
11.08	12.69	9.55	10.45	9.81	9.70	8.01	5.28
4.87	5.33	5.71	6.42	8.23	8.73	9.55	10.89
10.69	12.24	9.01	9.82	9.24	9.20	7.33	4.42
22511	26649	23655	26399	27309	28927	27699	26031
108.7	113.4	98.3	109.1	106.5	104.4	95.8	98.8
126982	166041	77771	106647	148609	190374	224949	153823
35216	39456	32018	34607	35347	33017	29016	16806
45583	57095	63242	76644	89377	91394	100133	105458
4331	5301	6594	7689	8642	9662	9778	10462
14304	16779	19750	23108	25835	28607	27291	29419
9492	9533	9586	9586	9558	6110	6006	6023
35921	45474	37119	41577	39597	42427	43489	49973
37732	45387	53936	64988	73320	78243	79187	81123
22980	27169	31329	36218	45795	51286	56928	58501
607	609	605	594	572	580	567	566
6241	6232	6405	5977	6252	6719	7114	7382
435	415	399	385	401	383	381	399
5643	5379	4749	4205	3863	3659	3615	3640
218	220	221	257	263	308	317	323
366	392	435	423	426	428	510	526
129	116	138	142	147	142	146	156
95	132	141	143	146	146	191	200

?1994-2018 China Academic Journal Electronic Publishing House. All rights reserved. http://www.cnki.net

9-10 富县经济

指　　标	单　位	2000年	2005年	2006年	2007年	2008年
年底总人口	万人	14.26	14.83	14.84	14.86	14.93
生产总值	亿元	3.65	8.94	11.07	10.18	12.68
第一产业	亿元	1.72	2.36	2.90	3.52	4.35
第二产业	亿元	0.39	2.91	3.45	1.44	2.48
第三产业	亿元	1.53	3.67	4.72	5.22	5.85
# 工业增加值	亿元	0.28	2.80	3.32	1.24	2.20
人均生产总值	元	2580	6058	7463	6856	8516
生产总值指数	上年=100	110.2	116.1	109.4	97.0	114.7
全社会固定资产投资	万元	8295	19933	26344	46650	70140
地方财政收入	万元	2485	3174	3737	4505	5373
地方财政支出	万元	6882	16253	22178	30026	47960
农村居民人均纯收入	元	1704	2380	2690	3126	3859
城镇居民人均可支配收入	元				8100	10498
常用耕地面积	公顷	14833	10167	9949	9947	9847
粮食产量	吨	57802	50139	33723	30370	47490
农林牧渔业总产值	万元	30114	44898	50968	64458	82405
社会消费品零售总额	万元	11560	22065	25542	30362	38607
普通小学专任教师数	人	1139	1313	1316	1268	1215
普通小学在校学生数	人	23066	15282	15368	14947	14880
普通中学专任教师数	人	574	852	870	885	889
普通中学在校学生数	人	8950	14358	14800	13997	13152
卫生机构床位数	张	280	318	196	283	285
卫生技术人员	人	489	496	500	505	511
# 执业(助理)医师	人	199	210	166	183	190
注册护师、护士	人					

?1994-2018 China Academic Journal Electronic Publishing House. All rights reserved.　http://www.cnki.net

社会主要指标

2009年	2010年	2011年	2012年	2013年	2014年	2015年	2016年
14.93	14.98	15.02	15.05	15.15	15.21	15.47	15.69
14.56	19.39	25.32	30.12	34.54	40.48	41.07	44.71
5.03	7.17	8.73	10.37	11.42	12.47	12.21	13.39
2.44	4.48	6.75	8.93	9.59	13.66	13.38	13.77
7.09	7.75	9.84	10.82	13.53	14.35	15.48	17.55
2.11	4.12	6.31	8.45	8.23	12.24	11.84	12.11
9736	12965	16879	20033	21519	26667	26775	28697
116.3	125.5	115.1	112.3	106.9	112.0	109.5	108.9
87973	119151	170678	300393	445067	519674	587474	958350
6565	11793	17051	22508	26751	30844	27707	21431
54565	78555	93900	102100	115300	114500	137700	144300
4573	5497	6981	8084	9086	10313	10282	11134
13101	15498	18454	21647	24331	27136	26552	28649
9847	10069	9847	9847	9864	9864	9895	9999
47240	45256	36807	37304	35389	36257	36426	37408
94132	141870	171521	185474	208475	227397	217842	241647
42969	50519	59021	68223	86492	97440	106892	115030
1162	1132	1096	1087	1057	857	853	831
15013	14661	14220	11379	10230	10214	10883	11548
774	696	688	701	727	734	708	691
11715	11166	10511	8239	6984	6681	6703	6808
364	457	455	530	551	571	576	665
517	508	503	508	517	602	726	714
196	191	130	161	170	145	169	177
121	125	145	153	154	187	262	265

?1994-2018 China Academic Journal Electronic Publishing House. All rights reserved. http://www.cnki.net

9-11 洛川县经济

指　　标	单　位	2000年	2005年	2006年	2007年	2008年
年底总人口	万人	19.09	20.11	20.13	20.21	20.30
生产总值	亿元	15.00	48.02	65.11	85.47	94.61
第一产业	亿元	3.40	6.62	7.57	8.98	10.81
第二产业	亿元	9.15	35.95	51.46	69.29	75.31
第三产业	亿元	2.44	5.45	6.08	7.20	8.49
# 工业增加值	亿元	8.99	35.75	51.19	68.94	74.85
人均生产总值	元	7889	24307	32359	42377	46708
生产总值指数	上年=100	111.6	120.8	112.9	119.0	103.7
全社会固定资产投资	万元	9700	70858	99934	203685	289422
地方财政收入	万元	5914	4542	6314	7661	8617
地方财政支出	万元	8431	19519	27719	34566	63181
农村居民人均纯收入	元	2131	3080	3303	4020	4500
城镇居民人均可支配收入	元				9216	11428
常用耕地面积	公顷	22898	12197	11956	11896	11384
粮食产量	吨	95220	84299	59762	51573	76753
农林牧渔业总产值	万元	81322	111236	126268	157670	194974
社会消费品零售总额	万元	21398	41543	48608	57852	73438
普通小学专任教师数	人	1735	1541	1422	1247	1321
普通小学在校学生数	人	31717	22568	23175	22984	22104
普通中学专任教师数	人	847	1250	1185	1212	1281
普通中学在校学生数	人	14107	21785	22121	21880	21691
卫生机构床位数	张	501	510	510	504	483
卫生技术人员	人	647	613	514	538	544
# 执业(助理)医师	人	299	309	255	256	260
注册护师、护士	人					

?1994-2018 China Academic Journal Electronic Publishing House. All rights reserved.　http://www.cnki.net

社会主要指标

2009年	2010年	2011年	2012年	2013年	2014年	2015年	2016年
21.92	22.08	22.14	22.19	22.32	22.38	22.76	22.96
104.62	128.15	177.02	213.04	248.48	231.16	182.07	151.73
11.33	14.30	17.99	19.14	20.67	22.05	19.40	21.14
82.98	102.73	146.39	179.41	209.57	190.28	141.06	106.83
10.32	11.12	12.64	14.49	18.25	18.83	21.60	23.76
82.45	102.14	145.65	178.59	209.64	188.54	139.19	104.86
51476	58243	80058	96115	111140	103427	80669	66380
108.9	113.0	108.1	112.5	101.5	97.9	100.5	95.5
310890	376963	375282	493777	645291	556379	584924	668448
11410	14876	16927	21031	24401	26100	24701	24755
64330	91701	104149	127918	152218	140935	174179	164414
5301	6352	7978	9278	10410	11742	10785	11713
14095	16660	19875	23294	26136	28993	28086	29967
11460	11535	11322	11237	10657	10471	10286	10138
97282	108610	88175	94413	94540	99282	99886	105975
202713	255664	322117	342897	375878	400197	360148	386336
81736	96243	111348	128415	163235	183797	203647	219456
1313	1242	1234	1191	1058	1096	1160	1117
21645	20209	20069	16428	15182	14414	15280	17108
1227	1179	1161	1148	1243	1239	1226	1225
20389	18675	17665	16287	14799	13473	12241	11445
595	528	549	592	704	678	783	718
585	637	764	778	899	942	975	989
290	278	232	252	276	265	273	290
127	171	262	266	316	353	367	376

?1994-2018 China Academic Journal Electronic Publishing House. All rights reserved. http://www.cnki.net

9-12 宜川县经济

指　　标	单　位	2000年	2005年	2006年	2007年	2008年
年底总人口	万人	11.22	11.55	11.56	11.63	11.68
生产总值	亿元	1.93	4.72	5.41	6.32	8.41
第一产业	亿元	0.70	1.55	1.81	2.14	3.50
第二产业	亿元	0.19	0.49	0.60	0.62	0.77
第三产业	亿元	1.04	2.67	3.00	3.56	4.14
# 工业增加值	亿元	0.12	0.42	0.50	0.50	0.62
人均生产总值	元	1704	4138	4678	5448	7215
生产总值指数	上年=100	100.3	113.5	112.4	112.3	118.6
全社会固定资产投资	万元	8287	18377	25272	40048	51537
地方财政收入	万元	1170	1337	1708	2106	2806
地方财政支出	万元	4991	15217	20295	28423	42082
农村居民人均纯收入	元	1004	1762	2071	2486	3406
城镇居民人均可支配收入	元			6892	8806	11322
常用耕地面积	公顷	16559	11684	11693	11796	11941
粮食产量	吨	39790	36175	25059	26739	42782
农林牧渔业总产值	万元	12803	25136	30301	36537	60197
社会消费品零售总额	万元	7395	13318	14268	17731	22658
普通小学专任教师数	人	944	654	611	614	551
普通小学在校学生数	人	19579	9735	8741	7932	7036
普通中学专任教师数	人	352	474	505	553	564
普通中学在校学生数	人	6600	11144	11306	10722	9850
卫生机构床位数	张	171	171	201	202	208
卫生技术人员	人	302	305	332	347	450
# 执业(助理)医师	人	137	174	159	154	152
注册护师、护士	人					

?1994-2018 China Academic Journal Electronic Publishing House. All rights reserved.　http://www.cnki.net

社会主要指标

2009年	2010年	2011年	2012年	2013年	2014年	2015年	2016年
11.69	11.73	11.77	11.79	11.86	11.92	12.09	12.18
9.66	13.24	15.74	18.58	22.33	24.22	25.32	27.92
4.02	6.53	7.71	9.35	10.26	11.08	11.17	11.73
0.73	0.89	0.93	1.12	1.69	1.74	1.71	1.98
4.90	5.82	7.10	8.11	10.39	11.40	12.44	14.21
0.56	0.69	0.69	0.85	1.07	1.16	1.06	1.28
8267	11308	13397	15776	17573	20370	21096	23006
114.1	112.3	110.9	109.9	106.5	107.4	106.6	108.5
68149	88893	91192	123603	181101	234681	277587	321558
4028	6180	7119	8258	9505	11152	12616	15162
52350	63905	88595	100650	113693	105068	115119	138806
4193	5104	6477	7565	8496	9626	9644	10376
14198	16653	19668	22972	25867	28772	27237	29252
12851	11759	11748	11854	11807	14851	14669	14683
40932	41030	33715	37083	36614	37235	36322	35047
70063	111822	132110	162137	181592	196008	197875	202531
25196	29704	34621	40079	50902	57255	63209	71248
468	421	388	369	346	315	299	377
6488	6201	6128	6101	6386	6639	6982	7504
550	519	505	550	552	557	581	588
9369	9346	9175	8753	9360	9089	8991	9545
227	285	305	350	387	416	461	509
381	374	348	417	450	465	515	558
157	153	130	149	156	133	136	147
103	104	98	145	158	185	224	238

?1994-2018 China Academic Journal Electronic Publishing House. All rights reserved. http://www.cnki.net

9-13 黄龙县经济

指 标	单 位	2000年	2005年	2006年	2007年	2008年
年底总人口	万人	4.70	4.88	4.88	4.88	4.89
生产总值	亿元	1.28	2.59	2.91	3.55	4.60
第一产业	亿元	0.77	1.17	1.27	1.61	2.19
第二产业	亿元	0.14	0.21	0.22	0.25	0.32
第三产业	亿元	0.38	1.20	1.41	1.70	2.09
# 工业增加值	亿元	0.08	0.08	0.09	0.07	0.08
人均生产总值	元	2748	5389	5967	7277	9403
生产总值指数	上年=100	104.5	107.6	106.8	115.8	117.3
全社会固定资产投资	万元	6066	11152	11457	18294	26569
地方财政收入	万元	767	530	630	780	904
地方财政支出	万元	4192	12020	14400	19010	25018
农村居民人均纯收入	元	1346	2081	2269	2725	3200
城镇居民人均可支配收入	元				6983	8750
常用耕地面积	公顷	9481	10762	10760	10751	10699
粮食产量	吨	27728	75490	51826	52128	67491
农林牧渔业总产值	万元	13028	20341	21293	27305	36553
社会消费品零售总额	万元	3487	6277	6697	7961	9747
普通小学专任教师数	人	503	441	444	370	337
普通小学在校学生数	人	6625	4528	4410	4090	3484
普通中学专任教师数	人	179	242	241	254	255
普通中学在校学生数	人	2712	3829	3869	3765	3553
卫生机构床位数	张	188	160	166	165	165
卫生技术人员	人	149	146	116	132	170
# 执业(助理)医师	人	81	81	62	65	67
注册护师、护士	人					

?1994-2018 China Academic Journal Electronic Publishing House. All rights reserved. http://www.cnki.net

社会主要指标

2009年	2010年	2011年	2012年	2013年	2014年	2015年	2016年
4.95	4.94	4.95	4.95	4.96	4.97	4.97	4.99
5.51	6.35	7.64	8.86	11.15	12.16	13.27	13.97
2.60	2.76	3.45	3.72	3.92	4.56	4.81	4.88
0.38	0.47	0.53	0.89	1.32	1.07	1.23	1.07
2.52	3.12	3.66	4.25	5.91	6.45	7.23	8.02
0.11	1.56	0.15	0.46	0.80	0.51	0.63	0.41
11247	12834	15454	17895	20496	24512	26695	28040
114.2	111.2	110.1	111.1	108.3	108.2	108.4	106.8
32789	42533	46600	61109	82000	105400	135025	166004
1041	1295	1809	2410	3224	4226	4750	5690
34153	49974	56487	74078	94321	99232	100810	93137
3821	4570	5813	6766	7585	8503	8958	9710
10876	12779	15181	17671	19721	21975	24889	26879
10747	10885	11636	11865	11891	12050	11950	11756
73964	79483	67014	77375	82121	86149	95918	90572
43472	45806	57232	63990	69163	80656	82839	85086
10831	12722	14689	16915	21331	23777	26155	29075
338	329	322	338	316	292	240	245
3288	3101	2810	2496	2227	2186	2276	2262
272	268	271	260	271	262	254	230
3150	3014	2955	2572	2110	2012	1795	1738
165	156	157	159	161	167	167	192
171	108	196	202	234	238	230	246
97	43	59	60	68	72	76	78
28	18	32	34	56	60	70	72

?1994-2018 China Academic Journal Electronic Publishing House. All rights reserved.   http://www.cnki.net

9-14　黄陵县经济

指　　标	单　位	2000年	2005年	2006年	2007年	2008年
年底总人口	万人	11.72	12.30	12.31	12.37	12.42
生产总值	亿元	7.11	17.57	26.19	34.41	48.16
第一产业	亿元	1.31	1.83	2.16	2.54	2.94
第二产业	亿元	3.21	10.93	18.10	24.61	36.73
第三产业	亿元	2.58	4.81	5.92	7.26	8.50
# 工业增加值	亿元	2.77	10.45	17.45	23.80	35.62
人均生产总值	元	6062	14296	21221	27816	38840
生产总值指数	上年=100	106.9	111.9	110.3	118.2	124.6
全社会固定资产投资	万元	11947	76025	124882	161668	238765
地方财政收入	万元	4977	12546	15332	19099	24348
地方财政支出	万元	8393	21446	28080	35667	55198
农村居民人均纯收入	元	2047	2690	3024	3516	4285
城镇居民人均可支配收入	元			7836	9347	11964
常用耕地面积	公顷	14022	9622	9681	9450	9113
粮食产量	吨	44778	46787	35079	32129	48452
农林牧渔业总产值	万元	22037	32068	37309	45707	53786
社会消费品零售总额	万元	23946	35313	39727	46014	58471
普通小学专任教师数	人	1244	1338	1125	1140	1106
普通小学在校学生数	人	22251	15953	14120	13964	12067
普通中学专任教师数	人	551	696	697	707	705
普通中学在校学生数	人	9243	12457	12326	12115	12062
卫生机构床位数	张	668	704	670	630	616
卫生技术人员	人	773	771	646	734	892
# 执业(助理)医师	人	304	355	293	327	240
注册护师、护士	人					

?1994-2018 China Academic Journal Electronic Publishing House. All rights reserved.　http://www.cnki.net

社会主要指标

2009年	2010年	2011年	2012年	2013年	2014年	2015年	2016年
13.06	12.99	13.00	13.01	13.03	13.05	13.12	13.17
56.94	69.90	83.56	103.40	109.57	112.61	87.56	95.85
3.03	4.29	5.49	6.23	6.83	7.98	7.70	7.97
43.79	54.65	65.11	82.57	84.54	85.05	57.12	62.58
10.12	10.97	12.96	14.60	18.20	19.58	22.73	25.30
42.47	53.09	63.20	80.36	82.42	82.22	53.99	59.34
45828	53677	64308	79509	83359	86357	66940	72911
117.1	114.1	111.6	111.6	110.2	110.8	98.4	107.3
375456	495320	633456	823193	1037461	723916	768097	855318
46560	60464	79797	94946	103688	88293	85239	89842
75570	102533	121781	147243	156013	127301	145943	155397
5091	6130	7723	8974	10096	11267	10581	11405
14897	17578	20904	24479	27392	30213	27784	29922
9084	9016	8938	8938	8945	9774	9619	9241
50286	50552	42437	46616	45638	47939	50013	49456
55410	78114	100021	110239	122464	142682	137067	143451
65235	76983	90154	103960	131332	148109	162274	179431
1060	1126	1005	992	839	1056	1051	1049
10040	9408	8947	8520	8231	8439	8630	8705
702	672	684	685	684	619	627	627
11422	10357	9922	8785	7976	7609	7024	6848
645	705	720	720	755	755	775	769
702	758	764	769	811	729	755	824
243	258	250	281	293	281	269	258
204	235	234	233	246	216	228	310

?1994-2018 China Academic Journal Electronic Publishing House. All rights reserved. http://www.cnki.net
