# Supplementary material for: A study on the tourism efficiency of tourism destination based on DEA model: A case of ten cities in Shaanxi province
Source: PLoS One. 2024 Jan 19;19(1):e0296660. doi: 10.1371/journal.pone.0296660 (PMC10798521; doi:10.1371/journal.pone.0296660)
Supplement: S1 File — (ZIP) [file pone.0296660.s001.zip › Supporting information/Statistical yearbook/Yulin.caj]

十一、榆林市

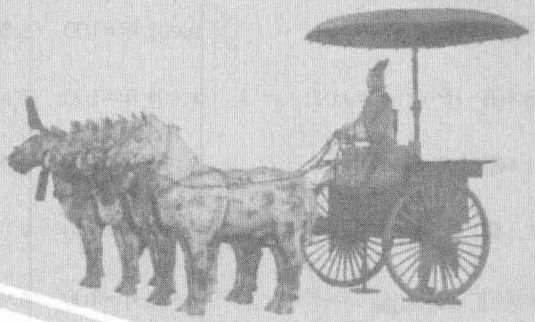

资料整理：王　燕　马　艳　潘英杰

?1994-2018 China Academic Journal Electronic Publishing House. All rights reserved.　http://www.cnki.net

11-1 榆林市经济

指　标	单 位	2000年	2005年	2006年	2007年	2008年
年底总人口	万人	326.04	329.93	330.33	332.80	333.98
人口自然增长率	‰		5.07	5.03	5.03	5.07
年底总户数	万户	83.74	96.74	99.99	107.36	112.47
生产总值	亿元	105.05	447.63	592.34	795.98	1172.76
第一产业	亿元	13.98	28.34	35.33	47.47	66.11
第二产业	亿元	46.60	260.06	358.05	503.92	796.10
第三产业	亿元	44.47	159.23	198.96	244.59	310.55
# 工业增加值	亿元	40.25	249.71	345.87	489.77	777.58
人均生产总值	元	3264	13602	17943	24007	35177
生产总值指数	上年=100	114.3	120.0	119.3	121.4	125.3
第一产业	上年=100	128.0	103.1	108.7	106.4	108.3
第二产业	上年=100	113.3	124.7	122.6	124.3	125.7
第三产业	上年=100	109.1	115.8	115.9	118.9	127.1
# 工业增加值	上年=100	113.8	124.9	123.1	124.9	125.9
人均生产总值指数	上年=100	112.5	119.4	119.0	120.9	124.6
非公有制经济增加值	亿元		134.13	172.52	204.28	390.18
文化产业增加值	亿元					
单位GDP能耗	吨标准煤/万元		2.510	2.426	2.343	2.195
单位GDP能耗比上年增长	%			-3.35	-3.42	-6.30
就业人员	万人	151.07	163.20	166.44	167.31	173.10
城镇单位就业人员	万人	19.53	20.20	20.32	20.94	21.51
# 国有单位	万人	17.12	17.63	16.68	17.16	17.55
集体单位	万人	1.23	0.91	0.85	0.97	0.96
# 在岗职工人数	万人	18.35	19.22	19.45	19.97	20.48
城镇单位就业人员平均工资	元					
城镇单位在岗职工平均工资	元	6569	14257	17487	23547	30786

?1994-2018 China Academic Journal Electronic Publishing House. All rights reserved.　http://www.cnki.net

社会主要指标

2009年	2010年	2011年	2012年	2013年	2014年	2015年	2016年
334.60	335.42	335.24	335.69	337.03	338.39	340.11	338.20
4.89	5.00	5.02	5.30	5.41	5.15	5.15	5.31
118.86	125.38	129.73	130.96	130.84	131.08	132.89	134.81
1302.31	1756.67	2292.25	2669.88	2779.46	2920.58	2491.89	2773.05
70.09	92.16	111.91	125.88	134.88	145.04	143.69	162.44
860.78	1205.77	1629.66	1928.53	1915.09	1966.78	1523.69	1684.69
371.44	458.74	550.68	615.47	729.49	808.76	824.51	925.92
837.34	1178.34	1597.96	1892.07	1880.51	1933.96	1484.49	1641.21
38950	52436	68358	79587	82633	86482	73453	81764
113.3	118.3	115.0	112.0	108.8	109.0	104.0	106.5
106.6	107.8	106.0	105.9	104.5	105.4	104.4	104.8
111.6	119.1	116.3	113.6	109.6	109.9	104.3	104.1
117.2	118.2	113.5	108.8	107.4	107.2	103.0	111.2
111.2	119.4	116.5	113.6	109.6	109.9	104.3	103.8
113.0	118.3	114.9	111.9	108.5	108.6	103.5	106.3
467.57	632.37	828.49	1004.65	1081.25	1179.98	1021.56	1164.48
					29.67	32.07	35.60
2.072	1.020	0.983	0.948	0.912	0.877	0.847	1.151
-5.60	-3.46	-3.60	-3.60	-3.80	-3.80	-3.50	-3.30
181.39	193.03	194.41	191.68	199.32	204.55	203.47	202.36
23.98	25.91	26.88	27.52	41.05	42.10	41.69	42.06
19.83	21.79	21.79	22.62	21.62	22.41	21.95	21.63
0.91	1.01	1.14	1.14	1.22	0.89	0.80	0.76
22.50	24.25	25.25	26.01	38.11	39.11	38.86	39.37
			51607	55597	56321	58442	62140
36406	40629	45053	53216	57647	58631	60194	63979

?1994-2018 China Academic Journal Electronic Publishing House. All rights reserved. http://www.cnki.net

11-1 续表 1

指 标	单 位	2000年	2005年	2006年	2007年	2008年
全社会固定资产投资	亿元	39.71	154.47	312.75	452.79	600.52
# 房地产开发	亿元	0.70	4.83	9.01	13.67	24.49
商品房销售面积	万平方米	3.71	24.43	37.41	38.82	61.15
# 住宅	万平方米	3.30	22.30	34.33	34.38	54.87
地方财政收入	亿元	6.15	23.84	35.66	50.12	70.01
地方财政支出	亿元	13.01	45.40	65.57	96.75	123.18
金融机构人民币各项存款余额	亿元	97.26	357.24	483.44	594.65	886.93
金融机构人民币各项贷款余额	亿元	82.97	196.67	293.40	406.14	467.93
农村居民人均纯收入	元	1062	1803	2094	2621	3402
城镇居民人均可支配收入	元	3505	6100	6691	8851	12197
城市人均公园绿地面积	平方米			3.0	4.8	5.4
城市人均道路面积	平方米		10.9	12.0	10.9	12.1
城市用水普及率	%		91.0	93.1	93.8	94.7
城市用气普及率	%		68.4	79.2	75.5	66.9
常用耕地面积	千公顷	596.42	500.72	511.32	559.94	566.08
农林牧渔业总产值	亿元	24.01	48.84	60.28	82.04	110.24
农作物总播种面积	千公顷	590.07	574.48	577.61	549.19	563.71
# 粮食作物	千公顷	493.76	474.86	482.10	458.97	469.64
粮食产量	万吨	70.90	106.23	106.50	103.16	132.24
棉花产量	吨	1	61	47	55	86
油料产量	吨	27841	47405	45786	32252	58246
蔬菜产量	吨	160400	335060	342140	404934	407216

?1994-2018 China Academic Journal Electronic Publishing House. All rights reserved. http://www.cnki.net

2009年	2010年	2011年	2012年	2013年	2014年	2015年	2016年
850.33	1105.46	1378.73	1771.23	1827.91	1647.04	1384.37	1467.45
22.98	28.33	29.12	52.49	89.65	73.93	46.99	40.55
83.54	92.32	96.82	121.95	139.10	106.76	52.01	55.79
78.67	84.60	88.09	112.97	119.50	101.85	51.04	54.36
91.18	125.54	180.25	249.06	260.73	267.85	295.58	232.69
171.08	237.19	316.14	398.88	422.22	423.32	465.36	471.13
1168.67	1452.72	1844.51	2252.82	2428.70	2618.24	2793.87	3026.38
677.04	898.52	1179.39	1548.08	1803.81	1935.71	2032.89	1979.33
4127	5113	6520	7681	8687	8967	9802	10582
14856	17545	20721	24140	26820	25676	27765	29781
7.5	7.1	7.7	10.3	10.6	16.6	18.7	12.4
13.7	12.8	13.2	12.8	15.0	20.8	27.8	18.2
96.3	95.1	95.1	88.4	93.7	93.9	97.7	86.7
90.9	77.1	90.0	90.8	92.1	92.2	92.6	77.7
573.46	574.33	575.00	580.63	594.87	602.62	653.68	685.46
120.01	152.88	187.06	209.72	232.74	250.03	250.08	282.31
585.48	594.99	563.38	576.01	581.88	590.47	601.21	609.51
488.78	498.75	466.29	471.16	474.00	476.37	482.37	487.48
152.83	165.13	142.03	154.03	154.77	158.10	142.90	160.08
77	77	113	121	114	120	131	107
64921	64921	69821	84884	79469	83182	73885	86379
470082	566039	588314	636375	706794	756600	797928	873688

?1994-2018 China Academic Journal Electronic Publishing House. All rights reserved. http://www.cnki.net

11-1 续表 2

指　　标	单 位	2000年	2005年	2006年	2007年	2008年
水果产量	吨	84554	144098	343104	190665	496232
# 苹果	吨	34385	36538	114673	95734	107414
肉类产量	吨	96171	142343	160995	100291	121461
# 猪牛羊肉	吨	88621	127504	150752	93098	113884
奶类产量	吨	16069	46852	53791	59364	66393
# 牛奶	吨	12047	42201	50612	54944	62546
禽蛋产量	吨	26006	29392	31048	28317	32031
水产品产量	吨	2511	5694	4091	2062	2197
规模以上工业企业单位数	个	140	363	610	593	639
规模以上工业总产值	亿元	36.61	360.04	528.20	773.87	1269.86
原煤产量	万吨	1640.00	6560.00	10588.00	11992.00	15533.00
天然原油产量	万吨	89.00	476.40	536.00	654.00	749.00
天然气产量	亿立方米		60.50	65.23	80.00	87.00
发电量	亿千瓦小时	15.04	68.46	86.61	166.51	238.05
水泥产量	万吨	24.00	29.30	60.13	65.00	97.50
建筑业企业单位数	个	80	95	118	114	149
建筑业企业年末从业人员	万人	0.80	1.26	1.91	1.16	2.04
建筑业总产值	亿元	8.79	28.64	35.07	50.10	67.09
房屋建筑施工面积	万平方米	122.70	317.54	390.69	455.07	451.91
房屋建筑竣工面积	万平方米	48.31	168.02	222.37	233.98	247.61
公路里程	公里	5711	7555	15508	14919	19331
# 等级公路	公里	4575	6717	6789	10988	16192
# 高速公路	公里		325	387	576	576
民用汽车拥有量	辆	19774	48254	106918	120657	132243
# 私人汽车	辆	8945	25662	73080	79935	92900
邮电业务总量	亿元	4.62	28.96	41.48	53.91	69.09
邮政业务总量	亿元	0.23	0.79	0.93	1.18	1.34
电信业务总量	亿元	4.39	28.17	40.55	52.73	67.76

?1994-2018 China Academic Journal Electronic Publishing House. All rights reserved. http://www.cnki.net

2009年	2010年	2011年	2012年	2013年	2014年	2015年	2016年
631541	509604	701276	736228	616671	606125	680241	782310
123048	140053	149227	167646	163751	200161	189728	210070
139280	154682	164523	171509	178961	184294	183924	183951
130171	144219	154532	161141	168172	173543	172584	172785
99115	82014	83232	85405	90203	92029	82291	84288
93352	79078	77927	82339	87583	89286	78387	77584
37908	41983	48101	49862	52331	52260	50067	50971
2305	5027	7699	6491	7299	8310	8497	9600
731	651	569	665	693	704	712	761
1392.56	1917.70	2613.53	3130.98	3120.82	3449.20	3206.37	3272.50
20929.00	25732.00	28354.95	32004.80	33854.89	36263.31	36103.50	36216.21
852.00	983.00	1082.64	1161.30	1229.58	1231.60	1186.94	1092.31
101.00	109.83	120.76	128.20	149.62	163.36	151.13	159.70
293.51	356.27	396.77	438.30	475.72	592.16	629.34	693.02
110.00	109.31	301.21	390.50	477.51	459.72	391.74	367.79
155	163	180	248	300	356	391	419
2.88	3.46	4.76	4.41	4.64	5.12	5.02	7.79
89.59	108.86	126.83	156.63	206.03	201.27	148.94	181.06
634.59	551.37	649.43	931.63	1205.65	1244.15	798.62	953.21
260.23	263.52	291.58	423.05	488.89	502.89	252.01	236.97
21986	22372	23393	25869	27176	27773	28641	28942
21395	22066	23083	25066	26083	26548	27083	27646
666	682	739	857	936	936	1005	1006
246606	309016	375889	448015	492129	509566	546932	571689
141394	255161	317502	385680	433290	457959	495766	520733
76.02	27.73	38.69	41.82	46.60	58.18	74.53	98.89
1.64	1.53	1.80	1.47	1.63	1.77	2.32	3.10
74.38	26.19	36.89	40.35	44.96	56.40	72.21	95.78

?1994-2018 China Academic Journal Electronic Publishing House. All rights reserved. http://www.cnki.net

11-1 续表 3

指 标	单 位	2000年	2005年	2006年	2007年	2008年
固定电话用户	万户	15.31	55.81	71.58	73.02	63.55
移动电话用户	万户	10.00	93.63	110.75	160.77	204.63
互联网宽带用户	万户	0.64	5.84	5.84	9.87	14.60
限额以上企业数	个					
批发业	个					
零售业	个					
住宿业	个					
餐饮业	个					
社会消费品零售总额	亿元	35.77	76.33	91.98	116.02	154.11
进出口总额	万美元		1909	1956	2916	4977
# 出口	万美元		1907	1956	2788	4550
实际外商直接投资额	万美元				414	
入境旅游人数	万人次	0.04	0.08	0.27	0.11	0.35
# 外国人	万人次	0.03	0.06	0.20	0.03	0.31
国际旅游外汇收入	万美元	8	77	124	271	23
国内旅游人数	万人次		279	292	311	350
国内旅游收入	亿元		6.18	9.63	14.08	15.60
星级饭店数	个		13	13	29	29
幼儿园数	所	79	143	182	192	247
在园儿童数	万人	5.88	4.55	5.10	5.20	5.94
普通小学学校数	所	5574	2630	2087	1590	1163
普通小学专任教师数	人	19584	20262	19815	19775	19702
普通小学在校学生数	万人	55.22	35.94	32.93	30.36	28.23
普通中学学校数	所	255	273	263	261	252
普通中学专任教师数	人	10011	15635	15979	16447	17001
普通中学在校学生数	万人	22.41	33.91	33.96	32.11	30.63
卫生机构数	个	549	486	499	467	457
卫生机构床位数	张	6274	7885	8211	9481	10756
卫生技术人员	人	7717	9424	9627	10978	10964
# 执业(助理)医师	人	4800	4774	4861	5080	4539
注册护师、护士	人	1573	2192	2320	2699	2944

?1994-2018 China Academic Journal Electronic Publishing House. All rights reserved. http://www.cnki.net

2009年	2010年	2011年	2012年	2013年	2014年	2015年	2016年
56.34	56.65	59.65	58.27	57.50	53.75	51.25	47.69
236.21	260.42	292.66	319.51	323.53	338.04	349.41	348.43
19.47	20.16	24.55	28.61	34.48	34.56	37.07	52.70
384	443	469	505	551	559	528	557
69	84	92	106	115	118	113	129
184	222	237	257	282	293	281	297
50	50	32	62	75	75	73	71
81	87	108	80	79	73	61	60
176.72	213.64	259.18	307.70	348.27	374.74	396.41	422.05
3674	8003	10048	4619	4611	2.93(亿元)	1.99(亿元)	9.68(亿元)
3503	7386	7544	4141	4194	2.55(亿元)	1.83(亿元)	4.09(亿元)
660	1950	2090	3000	3051	7266	10653	3558
0.24	0.26	0.23	0.20	0.19	0.19	0.30	0.33
0.23	0.24	0.23	0.19	0.16	0.16	0.17	0.30
12	14	14	16	17	20	25	30
350	530	675	1170	1470	1770	1990	2480
18.00	23.00	29.50	58.50	75.00	95.25	107.50	142.00
34	33	31	32	32	29	29	27
251	363	475	542	674	933	1026	956
5.89	7.97	10.27	12.72	15.01	16.37	17.58	18.62
739	653	593	549	490	438	384	364
19248	18886	16362	16488	16721	15781	15761	15379
26.36	25.02	24.39	21.77	21.71	22.86	24.59	26.54
241	238	231	217	213	215	212	198
17186	17363	18910	16271	18444	18421	15839	17293
28.31	25.34	22.53	19.50	18.20	17.47	16.86	16.55
465	467	5198	4993	4921	4939	4861	4631
11529	13106	14390	15762	16778	17248	18301	20436
12256	13561	15785	17791	20499	21245	22191	24890
4690	4853	4922	5230	5714	5852	6151	6659
3849	4627	5838	6918	7690	8098	8681	9942

?1994-2018 China Academic Journal Electronic Publishing House. All rights reserved. http://www.cnki.net

11-2 榆阳区经济

指　　标	单 位	2000年	2005年	2006年	2007年	2008年
年底总人口	万人	43.07	50.27	51.65	52.06	52.24
生产总值	亿元	23.30	77.92	95.50	106.17	140.92
第一产业	亿元	3.03	4.75	5.11	7.80	11.01
第二产业	亿元	6.79	26.86	40.35	42.65	61.10
第三产业	亿元	13.49	46.31	50.05	55.72	68.81
# 工业增加值	亿元	5.21	22.15	34.01	35.00	52.28
人均生产总值	元	5768	15501	18491	20394	26975
生产总值指数	上年=100	106.0	114.5	116.0	116.4	126.5
全社会固定资产投资	万元	258891	417044	649387	905014	1271416
地方财政收入	万元	7573	17100	26022	31281	43524
地方财政支出	万元	11558	34250	48326	67258	92711
农村居民人均纯收入	元	1575	2416	2921	3305	4185
城镇居民人均可支配收入	元	4023	6518	7035	8965	12210
常用耕地面积	公顷	57549	52455	52460	50977	51436
粮食产量	吨	158797	180034	153642	215100	221672
农林牧渔业总产值	万元	46344	82623	88692	134424	190005
社会消费品零售总额	万元	77628	131947	159273	206060	275600
普通小学专任教师数	人	3025	2704	2658	2743	2819
普通小学在校学生数	人	69268	58649	57824	56029	54169
普通中学专任教师数	人	1814	2381	2483	2682	3164
普通中学在校学生数	人	37138	59178	61727	63465	66081
卫生机构床位数	张	1421	2135	2351	2473	2677
卫生技术人员	人	1852	2151	2300	3100	3138
# 执业(助理)医师	人		1043	1095	1438	1193
注册护师、护士	人		576	638	864	1036

?1994-2018 China Academic Journal Electronic Publishing House. All rights reserved.　http://www.cnki.net

社会主要指标

2009年	2010年	2011年	2012年	2013年	2014年	2015年	2016年
63.68	63.82	63.83	64.07	64.42	64.89	64.91	65.37
183.31	249.00	325.02	401.09	461.26	515.03	516.50	556.71
11.20	13.82	19.44	21.17	22.48	24.37	23.72	28.53
92.26	137.15	189.57	246.62	258.63	290.80	285.95	302.01
79.86	98.03	116.01	133.30	180.15	199.86	206.83	226.18
82.22	126.91	177.85	233.24	233.44	258.79	255.22	262.13
35067	47360	50924	62719	71798	79657	79584	85464
113.8	119.6	113.6	112.3	121.6	111.2	106.0	108.5
1547201	2282367	2843940	3729290	4593529	5110300	3600897	3705184
66293	93249	139158	172955	210948	239352	243348	215935
140059	184757	271719	314069	335598	405025	409384	450187
5321	6605	8428	10001	11331	12656	11015	11929
15478	18868	22359	26071	29069	32217	29110	31206
51522	51600	51689	53168	63415	65069	63710	77227
236877	258608	222432	243070	237364	239639	218894	239738
200504	232872	330869	359612	395612	430033	444494	503933
342628	434916	545471	705370	1127829	1177959	1185171	1250501
2962	2917	3012	3129	3114	2962	3442	3693
52602	53155	53971	51944	53971	59148	65250	72077
3355	3571	3647	3576	3618	3732	3859	4275
64413	58664	53695	48119	47164	46998	47824	48716
2815	3061	3608	3614	3742	4042	4517	4568
3710	3919	4229	5150	5661	6140	6535	7109
1292	1385	1475	1587	1691	1857	2056	2148
1342	1415	1490	2015	2149	2413	2613	2835

?1994-2018 China Academic Journal Electronic Publishing House. All rights reserved. http://www.cnki.net

11-3 横山区经济

指 标	单 位	2000年	2005年	2006年	2007年	2008年
年底总人口	万人	32.71	28.86	28.51	28.54	28.72
生产总值	亿元	6.56	23.78	29.05	34.79	61.88
第一产业	亿元	1.47	3.41	3.81	5.42	7.01
第二产业	亿元	3.05	12.33	16.81	19.80	41.63
第三产业	亿元	2.04	8.04	8.43	9.56	13.24
# 工业增加值	亿元	2.68	11.90	16.24	19.17	40.57
人均生产总值	元	2196	8618	10521	12157	21615
生产总值指数	上年=100	153.1	112.4	117.7	119.8	123.2
全社会固定资产投资	万元	5024	43914	66772	179011	708104
地方财政收入	万元	2763	5694	3520	5189	10530
地方财政支出	万元	7474	22029	23198	42913	66322
农村居民人均纯收入	元	921	1520	1839	2250	3381
城镇居民人均可支配收入	元				8047	12163
常用耕地面积	公顷	59221	57303	58370	58433	58498
粮食产量	吨	49056	115065	108330	134752	133721
农林牧渔业总产值	万元	22431	55265	60153	90064	117004
社会消费品零售总额	万元	20560	38500	49791	67284	92700
普通小学专任教师数	人	1239	1807	1668	1625	1664
普通小学在校学生数	人	58843	37846	33340	29957	28600
普通中学专任教师数	人	809	1529	1556	1567	1592
普通中学在校学生数	人	20054	33588	33161	30295	28924
卫生机构床位数	张	359	390	390	712	712
卫生技术人员	人	369	313	283	426	467
# 执业(助理)医师	人	151	169	147	212	234
注册护师、护士	人	82	62	59	86	93

?1994-2018 China Academic Journal Electronic Publishing House. All rights reserved. http://www.cnki.net

社会主要指标

2009年	2010年	2011年	2012年	2013年	2014年	2015年	2016年
28.74	28.83	28.83	28.95	29.26	29.55	29.66	29.97
60.75	83.00	93.11	104.22	110.65	118.41	110.80	121.16
7.20	9.33	10.85	12.16	13.35	14.19	13.75	16.06
36.17	52.63	55.96	61.76	66.18	70.06	60.75	63.85
17.38	21.04	26.30	30.30	31.12	34.16	36.30	41.26
34.78	50.74	53.67	59.13	62.71	66.78	58.19	61.11
21139	28860	32296	36075	38019	40268	37427	40639
111.0	122.6	112.2	110.7	112.9	109.4	105.6	107.7
851400	892795	1136591	1554232	1757287	1025300	746433	1068770
14985	20992	20295	37381	44442	37826	40071	27689
92015	125072	156051	198881	213483	197425	264717	234524
4215	5264	6701	7860	8725	9364	9760	10522
14696	17400	20515	23920	26527	29021	25905	27951
59370	59460	59540	59573	60855	60619	60623	65408
153352	164047	141099	152742	156732	161313	158227	167004
124991	157063	184325	205689	232421	246554	248690	111732
118832	183616	279849	382095	437790	486403	509750	539063
1783	1696	1651	1643	1587	1349	1353	1641
26055	24862	22996	18350	17473	18313	19518	38606
1557	1539	1602	1601	1546	1521	1239	1791
26124	23012	20496	16703	14571	13397	12587	21167
704	720	859	880	1066	1190	1206	776
502	488	715	899	1064	1053	1071	2145
250	222	240	254	277	319	293	319
110	115	230	290	338	335	336	828

?1994-2018 China Academic Journal Electronic Publishing House. All rights reserved.   http://www.cnki.net

11-4 神木县经济

指 标	单 位	2000年	2005年	2006年	2007年	2008年
年底总人口	万人	37.24	39.36	40.53	41.25	41.36
生产总值	亿元	23.42	140.25	191.13	267.07	381.75
第一产业	亿元	1.80	3.59	4.65	5.27	6.36
第二产业	亿元	11.76	75.94	105.56	161.54	249.98
第三产业	亿元	9.86	60.72	80.92	100.26	125.42
# 工业增加值	亿元	11.14	74.23	104.25	159.49	247.15
人均生产总值	元	6436	37109	47394	64325	92299
生产总值指数	上年=100	139.9	142.9	152.0	121.4	118.2
全社会固定资产投资	万元	218775	402165	506679	690143	1340716
地方财政收入	万元	13302	68267	67659	100120	164475
地方财政支出	万元	16334	76532	112623	156056	236415
农村居民人均纯收入	元	1458	2788	3295	5034	6029
城镇居民人均可支配收入	元				12636	16075
常用耕地面积	公顷	56566	40310	45420	45220	45080
粮食产量	吨	105117	121848	101220	125150	122625
农林牧渔业总产值	万元	32685	64528	80519	90182	109770
社会消费品零售总额	万元	53672	120857	141053	168814	227600
普通小学专任教师数	人	2345	1887	1817	1930	1898
普通小学在校学生数	人	60710	40473	37443	34473	31237
普通中学专任教师数	人	1220	2128	2207	2200	2093
普通中学在校学生数	人	30056	47405	46230	42825	41207
卫生机构床位数	张	654	1190	1257	1317	1328
卫生技术人员	人	689	917	1052	1373	1454
# 执业(助理)医师	人		464	546	653	664
注册护师、护士	人		264	313	314	361

?1994-2018 China Academic Journal Electronic Publishing House. All rights reserved. http://www.cnki.net

社会主要指标

2009年	2010年	2011年	2012年	2013年	2014年	2015年	2016年
45.47	45.58	45.58	45.65	45.86	45.92	45.95	46.34
452.64	604.94	771.01	1003.89	882.12	914.33	769.53	904.80
6.52	8.10	9.67	10.63	11.46	11.97	11.99	13.24
299.72	417.32	545.27	761.65	650.54	654.33	514.50	614.37
146.40	179.52	216.07	231.61	220.11	248.03	243.04	277.19
296.68	414.04	541.18	757.17	656.76	663.75	522.08	624.29
109360	146051	169155	220079	192792	199244	16753	196078
114.0	116.3	111.7	115.0	101.2	111.3	106.7	107.6
1885649	2227331	2742559	3474440	2855752	2254563	2483685	2660638
209069	268558	420152	535610	501146	540203	585202	530624
276980	392265	528048	678458	636886	688726	809134	773602
7223	8672	10798	12537	13225	13622	12046	12875
19103	22301	26064	29316	30100	32610	28450	30384
45080	45110	45153	45155	45155	48474	81855	81990
126187	130010	111824	121133	123983	124121	122762	125266
115920	136925	164526	180649	205324	214584	215555	280183
266836	298868	371276	415989	436268	460490	472693	510277
1928	1818	1800	1919	1902	1649	1769	1014
28047	27216	27337	30132	32017	34447	37512	19624
2081	2042	2307	2020	2022	2135	1922	1643
37295	31385	26530	23147	21129	20012	18717	12322
1656	2256	2319	2564	2424	2458	2702	2571
1593	2112	2736	2935	3200	3153	3139	3359
707	768	714	776	878	908	976	998
452	679	1073	1269	1308	1302	1393	1564

?1994-2018 China Academic Journal Electronic Publishing House. All rights reserved. http://www.cnki.net

11-5 府谷县经济

指 标	单 位	2000年	2005年	2006年	2007年	2008年
年底总人口	万人	22.20	23.47	23.89	24.36	24.44
生产总值	亿元	8.83	28.12	42.24	69.29	120.06
第一产业	亿元	0.60	1.22	1.45	1.73	2.07
第二产业	亿元	4.17	15.41	26.78	49.74	95.05
第三产业	亿元	4.06	11.49	14.01	17.82	22.94
# 工业增加值	亿元	3.93	15.05	26.20	48.82	93.59
人均生产总值	元	4210	14988	20803	22270	49124
生产总值指数	上年=100	110.5	124.1	129.3	132.7	138.1
全社会固定资产投资	万元	37821	162498	271605	613877	1020202
地方财政收入	万元	7156	21620	21319	33851	66432
地方财政支出	万元	10941	32111	40547	57971	109260
农村居民人均纯收入	元	1037	1745	2126	2580	4703
城镇居民人均可支配收入	元				9934	14035
常用耕地面积	公顷	47372	44562	44510	42538	42599
粮食产量	吨	22067	51967	29323	63940	62509
农林牧渔业总产值	万元	10469	18378	19655	24129	36000
社会消费品零售总额	万元	40416	80101	97306	126238	168700
普通小学专任教师数	人	1436	1752	1761	1627	1602
普通小学在校学生数	人	34113	22630	21154	20036	19265
普通中学专任教师数	人	802	1250	1239	1232	1248
普通中学在校学生数	人	16534	22503	22197	21431	20007
卫生机构床位数	张	505	534	602	640	731
卫生技术人员	人	705	706	723	866	864
# 执业(助理)医师	人				430	324
注册护师、护士	人				201	236

?1994-2018 China Academic Journal Electronic Publishing House. All rights reserved. http://www.cnki.net

社会主要指标

2009年	2010年	2011年	2012年	2013年	2014年	2015年	2016年
26.03	26.08	26.09	26.18	26.36	26.30	26.31	26.53
162.56	265.97	407.95	450.52	463.82	459.98	365.83	392.57
2.38	3.75	4.54	5.21	5.43	5.91	5.84	6.50
132.69	226.85	360.25	396.05	368.72	355.33	257.58	272.31
27.50	35.37	43.16	49.26	89.68	98.73	102.41	113.76
45.86	223.79	356.84	392.25	364.37	352.71	255.44	270.08
66365	108338	156393	172382	176560	174697	139072	148589
118.4	122.6	124.3	110.8	111.1	108.4	105.7	104.0
1531684	2140650	2709395	3357128	2403641	1506600	1059561	1140084
105716	176022	236423	274226	230690	231055	164586	140184
136478	254811	297134	356939	324713	298998	252564	283489
5615	7786	9927	11783	13001	13409	10871	11610
16922	21415	25312	29083	30392	32732	28417	30321
43077	43150	43204	43260	43263	43263	43263	43263
70908	73456	63181	68411	65176	67214	32388	58391
40900	61760	75455	86360	92635	101206	100327	238197
203106	244991	313225	385810	400128	440059	469953	474508
1578	1451	1424	1210	1317	951	979	1226
17632	17268	16975	16985	17074	17707	18493	21107
1224	1242	1669	1621	1597	1654	1254	1450
19277	17366	15255	14234	13146	12695	12576	12048
832	709	809	1198	1511	1361	1280	929
1012	1230	1519	1624	1766	1794	1854	1922
385	344	372	433	417	392	410	441
314	500	687	755	790	790	863	903

?1994-2018 China Academic Journal Electronic Publishing House. All rights reserved. http://www.cnki.net

11-6 靖边县经济

指　标	单 位	2000年	2005年	2006年	2007年	2008年
年底总人口	万人	28.14	29.95	30.09	30.57	30.68
生产总值	亿元	11.47	106.87	131.46	205.65	256.48
第一产业	亿元	1.74	3.42	3.88	5.33	8.58
第二产业	亿元	6.03	95.08	117.43	187.29	230.92
第三产业	亿元	3.70	8.37	10.15	13.02	16.97
# 工业增加值	亿元	5.19	94.23	116.56	186.38	229.98
人均生产总值	元	4303	36992	45129	69634	84109
生产总值指数	上年=100	143.4	116.8	126.5	120.0	127.5
全社会固定资产投资	万元	46032	54752	153550	251751	708456
地方财政收入	万元	7265	29504	25550	36175	62448
地方财政支出	万元	11023	44745	55027	72304	103602
农村居民人均纯收入	元	1215	1842	2022	3007	4850
城镇居民人均可支配收入	元				9860	14891
常用耕地面积	公顷	56946	53803	57730	73427	75579
粮食产量	吨	84945	159108	149957	195992	200186
农林牧渔业总产值	万元	27359	63380	77364	106316	149003
社会消费品零售总额	万元	37109	112435	131793	159814	211164
普通小学专任教师数	人	1348	1685	1634	1892	1823
普通小学在校学生数	人	53288	38364	35415	33140	31465
普通中学专任教师数	人	881	1693	1693	1767	1700
普通中学在校学生数	人	19232	33143	33848	31667	29219
卫生机构床位数	张	436	533	533	1016	1077
卫生技术人员	人	457	549	562	1142	1203
# 执业(助理)医师	人		260	306	433	448
注册护师、护士	人		83	119	232	221

?1994-2018 China Academic Journal Electronic Publishing House. All rights reserved. http://www.cnki.net

社会主要指标

2009年	2010年	2011年	2012年	2013年	2014年	2015年	2016年
35.53	35.62	35.62	35.73	35.94	36.32	36.51	36.86
203.29	240.39	292.96	317.05	320.05	339.79	253.67	244.94
8.93	11.68	14.70	16.65	17.63	19.60	19.75	22.13
171.01	198.61	242.22	259.48	246.16	255.57	167.80	149.12
23.35	30.11	36.04	40.92	56.26	64.62	66.12	73.69
169.73	196.65	240.23	257.16	247.73	259.46	169.98	149.57
66176	78150	82246	88872	89312	94046	69480	66767
104.5	119.0	118.6	108.8	109.5	109.3	99.9	102.3
1022097	1364192	1656764	2165035	2709061	2175558	1565161	1659312
80078	120083	131051	152115	185040	200008	187001	121870
138538	208167	234968	282473	313363	327688	331043	311727
6031	7599	9689	11413	12680	13086	11058	11910
18026	21132	24915	28652	30085	33205	29577	31558
77751	77870	77966	80154	80154	80154	85194	85751
230746	245275	210964	229913	231758	237461	217160	235818
159226	199310	251958	284670	317568	350910	355569	399931
238871	312500	374174	449924	496624	534294	596719	641528
1899	1878	1821	1795	1911	1697	1655	1417
30792	28982	29761	28952	30371	32353	35509	22283
1768	1826	1940	1793	1795	1783	1597	1565
28809	26344	24544	22567	21686	21288	21195	16034
1122	1105	1156	1161	1466	1456	1572	1212
1252	1286	1328	1340	1680	1892	1938	2020
399	413	289	337	423	468	465	546
325	341	451	434	513	566	580	649

?1994-2018 China Academic Journal Electronic Publishing House. All rights reserved.   http://www.cnki.net

11-7 定边县经济

指　　标	单 位	2000年	2005年	2006年	2007年	2008年
年底总人口	万人	31.21	29.49	29.52	29.61	29.71
生产总值	亿元	5.11	36.64	53.60	71.16	121.73
第一产业	亿元	1.41	3.71	3.71	6.14	7.33
第二产业	亿元	1.89	25.21	40.22	53.12	97.53
第三产业	亿元	1.81	7.72	9.67	11.90	16.87
# 工业增加值	亿元	1.76	25.02	40.01	52.89	97.29
人均生产总值	元	1771	12427	18163	24069	41042
生产总值指数	上年=100	110.2	134.7	135.6	137.2	125.9
全社会固定资产投资	万元	19667	11876	167108	247337	365610
地方财政收入	万元	4915	16877	13131	26780	55207
地方财政支出	万元	10898	30917	33770	60077	86102
农村居民人均纯收入	元	1131	1641	1621	2660	3411
城镇居民人均可支配收入	元				8260	13015
常用耕地面积	公顷	102178	87075	93170	128279	129620
粮食产量	吨	55199	187847	128512	239691	192514
农林牧渔业总产值	万元	26797	67088	66527	109911	127532
社会消费品零售总额	万元	23605	58415	68786	83797	107000
普通小学专任教师数	人	1456	1666	1597	1578	1640
普通小学在校学生数	人	51256	40966	34951	30108	26464
普通中学专任教师数	人	670	1260	1239	1321	1399
普通中学在校学生数	人	15891	25243	25477	24432	23632
卫生机构床位数	张	297	397	417	572	572
卫生技术人员	人	441	497	504	574	573
# 执业(助理)医师	人				272	219
注册护师、护士	人				149	166

?1994-2018 China Academic Journal Electronic Publishing House. All rights reserved. http://www.cnki.net

社会主要指标

2009年	2010年	2011年	2012年	2013年	2014年	2015年	2016年
31.89	31.96	31.93	31.91	32.11	32.20	32.40	32.71
128.83	172.72	232.77	291.01	320.56	346.56	244.91	230.03
8.12	12.66	13.29	14.34	16.38	18.14	17.91	19.64
99.16	133.97	188.75	240.85	252.20	274.05	169.49	143.96
21.55	26.09	30.73	35.82	51.98	54.37	57.51	66.42
35.22	133.68	188.25	240.32	252.91	274.50	169.56	143.81
43341	58077	72866	91169	100144	107776	75824	70658
110.3	117.5	117.6	108.1	108.3	108.1	96.2	102.2
887228	1112980	1313372	1667118	2080917	2431000	2263379	1901190
71036	107000	132001	160039	185767	210812	230022	131592
142705	193990	230704	277248	303421	327717	343155	323574
4524	6233	8010	9492	10744	11829	10926	11789
15228	18336	21655	25271	28152	30911	28895	30885
132220	132530	132692	134162	136878	138925	152968	161502
259156	304368	261791	282210	293789	298472	270559	298428
142871	220498	233905	249735	293266	323520	323223	354825
124564	151860	182825	214616	242480	271418	301731	328398
2267	1642	1587	1407	1662	1393	1305	1339
23712	21848	20474	18986	18807	19324	20540	18088
1458	1434	1455	1436	1616	1631	1513	1603
22240	21510	20738	20703	18830	18536	17127	14264
711	931	995	1171	1328	1398	1496	1355
628	626	865	975	1335	1374	1459	1709
226	220	273	276	337	340	351	383
184	192	290	370	457	492	574	651

?1994-2018 China Academic Journal Electronic Publishing House. All rights reserved.   http://www.cnki.net

11-8 绥德县经济

指 标	单 位	2000年	2005年	2006年	2007年	2008年
年底总人口	万人	35.64	30.94	29.95	29.99	30.09
生产总值	亿元	3.51	11.38	13.83	16.86	20.20
第一产业	亿元	0.96	1.69	3.27	4.01	4.60
第二产业	亿元	0.47	1.72	2.18	2.41	2.74
第三产业	亿元	2.08	7.97	8.38	10.44	12.86
# 工业增加值	亿元	0.35	1.17	1.14	1.27	1.46
人均生产总值	元	990	3251	4543	5625	6724
生产总值指数	上年=100	165.0	117.5	114.8	120.2	116.8
全社会固定资产投资	万元	4407	6651	12611	37194	179505
地方财政收入	万元	2830	2570	2408	2956	3364
地方财政支出	万元	8272	22571	30522	46772	68438
农村居民人均纯收入	元	1300	1677	2013	2341	2941
城镇居民人均可支配收入	元	2986	5682	5687	7904	11037
常用耕地面积	公顷	50689	41078	40250	40723	41066
粮食产量	吨	47418	60596	82092	81180	77960
农林牧渔业总产值	万元	15596	26198	49536	59873	72384
社会消费品零售总额	万元	49699	112134	136465	174977	220900
普通小学专任教师数	人	2291	2396	2369	2276	2173
普通小学在校学生数	人	55946	31055	28778	25857	24257
普通中学专任教师数	人	1086	1715	1768	1813	1822
普通中学在校学生数	人	24153	34756	35284	33456	31105
卫生机构床位数	张	978	804	804	918	1528
卫生技术人员	人	1168	1326	1304	1588	1507
# 执业(助理)医师	人	244	348	347	687	652
注册护师、护士	人	128	225	228	445	448

?1994-2018 China Academic Journal Electronic Publishing House. All rights reserved. http://www.cnki.net

社会主要指标

2009年	2010年	2011年	2012年	2013年	2014年	2015年	2016年
29.55	29.64	29.65	29.66	29.52	29.64	29.89	29.09
24.78	33.05	40.47	47.62	51.95	54.76	59.41	67.15
5.07	6.19	8.43	9.08	9.61	10.46	10.30	10.98
3.55	5.54	6.75	9.35	9.24	7.53	7.01	8.91
16.16	21.32	25.29	29.19	33.11	36.77	42.10	47.26
1.62	2.94	4.02	5.82	6.54	5.43	4.82	6.64
8229	10969	13652	16058	17558	18511	19960	22770
121.5	120.1	110.5	111.6	107.4	107.9	109.6	108.5
201088	299576	303932	376186	427452	493978	516820	592813
3808	4018	5188	6198	7307	8261	9955	11839
90700	122000	158000	192100	235100	236000	235266	262600
3686	4564	5623	6630	7326	8227	8240	8949
14009	16617	19492	22708	25138	27426	24664	26760
41407	41470	41517	41557	41727	41741	41741	43083
90370	93733	80621	87932	87412	89895	83902	90981
82481	99970	136345	146772	158733	171872	167692	183963
213619	228635	247503	269008	94727	102557	115684	125236
2150	1998	1932	1998	2050	1590	1346	823
21220	18516	17319	16258	15784	16305	17246	9158
1861	1872	2031	2017	2147	2035	1473	950
26539	23319	20346	17834	17176	16053	15108	7012
1275	1806	1946	2262	2320	2426	2528	2810
1738	1871	2090	2195	2644	2736	2855	2966
641	693	681	679	764	713	704	736
678	816	957	1034	1196	1265	1341	1365

?1994-2018 China Academic Journal Electronic Publishing House. All rights reserved.   http://www.cnki.net

11-9 米脂县经济

指　标	单 位	2000年	2005年	2006年	2007年	2008年
年底总人口	万人	23.40	21.84	21.53	21.61	21.68
生产总值	亿元	3.29	6.73	9.60	15.35	20.98
第一产业	亿元	1.11	1.04	1.56	2.18	3.00
第二产业	亿元	0.82	0.98	1.75	4.87	6.76
第三产业	亿元	1.36	4.71	6.29	8.30	11.23
# 工业增加值	亿元	0.69	0.45	1.21	4.33	6.22
人均生产总值	元	1581	3082	4426	7116	9690
生产总值指数	上年=100	155.7	111.3	121.7	120.6	119.8
全社会固定资产投资	万元	6541	74690	32288	84565	91985
地方财政收入	万元	1155	861	1243	2015	2785
地方财政支出	万元	6863	18382	23329	36455	52200
农村居民人均纯收入	元	898	1487	1886	2408	3368
城镇居民人均可支配收入	元				7856	11981
常用耕地面积	公顷	32410	29448	24350	25316	26909
粮食产量	吨	60000	31185	46162	66766	80194
农林牧渔业总产值	万元	13135	18014	24391	35148	47195
社会消费品零售总额	万元	12959	25798	32525	46621	69600
普通小学专任教师数	人	1544	1482	1505	1441	1433
普通小学在校学生数	人	32827	15671	12883	10515	9478
普通中学专任教师数	人	644	847	873	907	958
普通中学在校学生数	人	13656	18301	17176	15213	13436
卫生机构床位数	张	308	351	351	465	475
卫生技术人员	人	363	369	359	469	468
# 执业(助理)医师	人				235	203
注册护师、护士	人				116	125

?1994-2018 China Academic Journal Electronic Publishing House. All rights reserved. http://www.cnki.net

社会主要指标

2009年	2010年	2011年	2012年	2013年	2014年	2015年	2016年
15.46	15.51	15.51	15.51	15.54	15.54	15.92	15.94
22.64	27.13	33.56	40.12	36.21	37.84	39.48	44.84
3.26	4.02	4.73	5.28	5.52	5.96	5.67	6.99
6.54	7.26	9.58	12.97	11.91	10.79	12.97	14.99
12.84	15.85	19.25	21.87	18.78	21.10	20.84	22.86
5.88	6.65	8.83	12.16	10.52	10.02	11.98	13.88
10427	12479	21638	25867	23323	24352	24799	28151
111.1	111.3	114.9	108.7	109.4	107.1	105.2	107.8
202582	211942	264266	326142	262416	272500	318613	365071
3228	4026	4444	4876	5601	8041	8160	9265
70005	97852	111380	139849	150677	160265	181363	191770
4209	5209	6407	7509	8290	8903	8894	9658
14375	17063	19998	23178	25681	27710	24667	26813
27715	27760	27794	27859	27859	27865	27865	27866
94208	88168	75834	83638	85418	88080	86282	100218
53404	64919	77104	86083	93653	100865	97077	118896
77500	96424	101550	77646	81672	91219	101158	110044
1526	1059	1015	886	1176	955	858	768
8805	8539	8800	8124	8015	8236	8669	6446
898	883	978	887	980	1002	711	687
11529	11092	10246	9251	8565	7696	7279	4150
477	487	570	665	606	601	614	512
471	511	530	529	635	660	652	827
201	208	215	212	199	190	189	210
143	155	161	184	225	232	225	315

?1994-2018 China Academic Journal Electronic Publishing House. All rights reserved.   http://www.cnki.net

11-10 佳县经济

指　　标	单 位	2000年	2005年	2006年	2007年	2008年
年底总人口	万人	26.44	24.66	24.33	24.37	24.45
生产总值	亿元	2.76	6.10	7.79	10.56	13.97
第一产业	亿元	1.03	1.82	3.00	4.10	5.52
第二产业	亿元	0.40	0.88	0.62	1.00	2.23
第三产业	亿元	1.33	3.40	4.17	5.46	6.22
# 工业增加值	亿元	0.19	1.82	0.45	0.75	1.87
人均生产总值	元	1126	2420	3180	4300	5720
生产总值指数	上年=100	103.6	186.2	118.2	124.8	114.4
全社会固定资产投资	万元	4217	20935	15765	30083	43323
地方财政收入	万元	1081	721	369	1158	1473
地方财政支出	万元	6715	18630	22335	35875	47943
农村居民人均纯收入	元	859	1393	1668	1962	2731
城镇居民人均可支配收入	元				7597	10403
常用耕地面积	公顷	40199	31333	31330	31334	31333
粮食产量	吨	36945	38270	75174	63331	57908
农林牧渔业总产值	万元	13899	29848	48874	63212	86852
社会消费品零售总额	万元	10214	15506	19027	24041	30400
普通小学专任教师数	人	1577	1746	1691	1602	1603
普通小学在校学生数	人	43405	26498	26056	25074	23412
普通中学专任教师数	人	645	866	893	897	934
普通中学在校学生数	人	14745	20887	20366	16711	15317
卫生机构床位数	张	481	525	444	319	376
卫生技术人员	人	469	503	424	485	425
# 执业(助理)医师	人				263	230
注册护师、护士	人				105	90

?1994-2018 China Academic Journal Electronic Publishing House. All rights reserved. http://www.cnki.net

社会主要指标

2009年	2010年	2011年	2012年	2013年	2014年	2015年	2016年
20.44	20.48	20.42	20.42	20.49	20.50	19.91	18.98
16.32	22.73	27.01	31.86	32.59	34.27	32.75	39.80
5.86	7.37	7.69	8.91	8.92	9.40	9.06	10.60
3.32	7.10	7.37	9.73	10.83	10.49	8.79	12.28
7.14	8.26	11.95	13.22	12.85	14.38	14.90	16.92
2.84	6.57	6.80	9.15	10.15	10.11	8.29	11.75
6666	9274	13208	5602	15934	16723	17046	20469
111.7	120.0	102.2	114.1	108.9	106.9	108.6	108.6
52273	103306	301677	423944	562603	573544	569225	615818
1860	2833	5429	7625	8205	9002	10260	8845
82363	96917	109301	157239	171363	176687	178702	206511
3435	4301	5458	6408	7254	8162	8174	8893
13018	15545	18262	21412	23810	26405	23484	25668
31333	31330	31346	31225	30838	30844	30844	33590
72889	80600	69325	70730	72629	74931	75530	81633
94649	115940	120924	141701	147758	155538	151820	176965
35884	32770	28801	25766	21833	24017	26850	29601
1554	1513	1348	1472	1433	1198	1040	489
21921	17909	15581	7719	6852	6409	6440	4132
887	900	936	993	1001	1014	792	229
13547	11026	9351	6514	5785	5650	4754	2904
499	526	552	591	593	594	600	303
433	460	475	509	767	766	731	847
214	211	228	243	268	252	234	253
107	128	128	138	178	182	180	201

?1994-2018 China Academic Journal Electronic Publishing House. All rights reserved. http://www.cnki.net

11-11 吴堡县经济

指 标	单 位	2000年	2005年	2006年	2007年	2008年
年底总人口	万人	8.31	7.60	7.49	7.52	7.55
生产总值	亿元	1.13	3.16	4.07	5.03	5.89
第一产业	亿元	0.15	0.36	0.71	0.91	1.21
第二产业	亿元	0.48	0.66	0.96	1.34	1.56
第三产业	亿元	0.50	2.14	2.40	2.78	3.13
# 工业增加值	亿元	0.15	0.36	0.71	0.91	1.21
人均生产总值	元	1580	3342	4242	5771	7485
生产总值指数	上年=100	119.0	127.4	128.7	127.2	128.5
全社会固定资产投资	万元	3666	6785	15041	29842	88886
地方财政收入	万元	670	835	700	1025	1156
地方财政支出	万元	3600	11288	13130	21462	30171
农村居民人均纯收入	元	680	1386	1767	2077	2706
城镇居民人均可支配收入	元				7873	10187
常用耕地面积	公顷	9924	8685	8080	8080	8060
粮食产量	吨	10034	5577	12307	12507	13399
农林牧渔业总产值	万元	2356	6058	10915	13852	19006
社会消费品零售总额	万元	7638	14273	18831	24580	32900
普通小学专任教师数	人	496	550	547	602	630
普通小学在校学生数	人	13588	7325	6550	5677	5017
普通中学专任教师数	人	218	343	345	344	341
普通中学在校学生数	人	4998	7459	6846	6168	5123
卫生机构床位数	张	125	213	249	263	278
卫生技术人员	人	160	186	200	250	233
# 执业(助理)医师	人	98	109	117	125	94
注册护师、护士	人	17	30	30	49	54

?1994-2018 China Academic Journal Electronic Publishing House. All rights reserved. http://www.cnki.net

社会主要指标

2009年	2010年	2011年	2012年	2013年	2014年	2015年	2016年
7.56	7.58	7.58	7.56	7.47	7.48	7.94	6.49
7.66	9.64	11.52	13.77	16.22	16.62	15.93	17.97
1.37	1.43	1.99	2.21	2.27	2.49	2.40	3.21
2.81	3.86	4.44	5.81	7.88	7.18	6.44	6.66
3.48	4.36	5.09	5.75	6.06	6.95	7.09	8.10
1.86	2.95	3.05	4.26	6.32	5.61	5.19	5.30
10086	12634	15198	18190	21580	22237	20662	24901
122.8	118.8	109.0	108.1	101.6	107.1	104.0	108.4
98531	109369	133888	160747	148126	90854	104968	150127
1387	1656	2015	2441	2873	3256	2836	3616
34816	53703	62537	83721	102286	105906	109127	112210
3515	4403	5582	6558	7352	8242	8155	8881
13227	16135	18975	21992	24367	26904	23516	25656
8076	8100	8120	8120	8113	8101	8098	8092
17319	19004	16346	17754	18227	18816	18460	22925
21631	22574	31629	34621	36487	39133	38156	50695
39409	40931	40877	41748	38793	42828	48310	53140
626	607	589	633	590	500	482	372
4799	4726	4746	4553	4243	3911	4071	4538
326	305	278	252	317	319	312	467
4722	4470	3874	3565	3075	2975	2975	2629
333	343	359	366	359	329	321	210
234	241	242	242	351	321	357	382
93	98	105	98	115	103	112	126
48	48	46	47	95	89	95	104

?1994-2018 China Academic Journal Electronic Publishing House. All rights reserved.   http://www.cnki.net

11-12 清涧县经济

指　　标	单 位	2000年	2005年	2006年	2007年	2008年
年底总人口	万人	22.07	17.23	17.01	17.05	17.11
生产总值	亿元	2.56	7.09	9.11	9.94	13.56
第一产业	亿元	1.04	1.50	2.90	3.16	5.23
第二产业	亿元	0.42	1.79	1.77	1.79	2.44
第三产业	亿元	1.09	3.80	4.44	4.99	5.89
# 工业增加值	亿元	0.35	1.42	1.39	1.40	1.99
人均生产总值	元	1225	3191	3953	4238	6270
生产总值指数	上年=100	135.6	130.0	120.6	107.7	122.7
全社会固定资产投资	万元	4512	12382	17558	26673	75683
地方财政收入	万元	1230	1009	704	1129	1502
地方财政支出	万元	6134	16288	22774	34597	49855
农村居民人均纯收入	元	689	1393	1845	1993	2767
城镇居民人均可支配收入	元				7667	11286
常用耕地面积	公顷	41477	25265	26260	26212	26212
粮食产量	吨	42293	44036	66424	60958	64212
农林牧渔业总产值	万元	16476	27416	40852	44841	81643
社会消费品零售总额	万元	11581	21470	25670	30322	39500
普通小学专任教师数	人	1147	1022	1050	1014	1011
普通小学在校学生数	人	30440	15992	13281	12337	9555
普通中学专任教师数	人	644	695	710	681	678
普通中学在校学生数	人	12136	15842	15022	14013	11238
卫生机构床位数	张	358	370	370	332	516
卫生技术人员	人	379	406	401	335	261
# 执业(助理)医师	人				119	64
注册护师、护士	人				68	54

?1994-2018 China Academic Journal Electronic Publishing House. All rights reserved.　http://www.cnki.net

社会主要指标

2009年	2010年	2011年	2012年	2013年	2014年	2015年	2016年
12.88	12.90	12.79	12.63	12.45	12.40	12.88	12.89
16.15	20.34	26.13	30.68	40.17	41.25	39.04	43.92
5.73	7.50	9.25	11.41	12.31	12.13	11.66	12.62
3.48	4.83	6.86	7.72	12.65	11.88	10.12	10.74
6.94	8.01	10.02	11.55	15.21	17.24	17.26	20.55
2.93	4.27	6.20	6.64	10.86	10.04	8.26	8.75
9417	11830	20343	24138	32034	33199	30311	34084
116.6	112.5	114.6	112.7	106.4	105.0	105.0	110.6
94562	138652	169462	217421	266752	324600	374045	440671
1951	2662	3282	4150	5352	6600	7896	9048
65440	93475	107085	145274	180168	161229	175256	184055
3502	4386	5465	6500	7352	8234	8234	8918
13456	15890	18718	21675	24124	26705	23529	25459
26217	26220	26244	26647	26820	27078	27079	27099
69056	76410	65721	71759	72893	75119	74361	79135
91486	118619	146992	180483	198024	197172	191669	207813
43474	46783	47552	51170	53399	59734	67880	82213
1023	933	939	889	752	579	501	739
9278	9420	9670	6753	4169	4012	4207	8513
633	609	650	622	684	520	377	620
9737	8365	7000	4496	3190	2813	2562	5405
607	607	607	642	689	714	738	380
295	371	538	596	697	653	695	739
72	76	101	111	119	101	121	225
77	116	182	222	245	227	254	251

?1994-2018 China Academic Journal Electronic Publishing House. All rights reserved. http://www.cnki.net

11-13　子洲县经济

指　　标	单 位	2000年	2005年	2006年	2007年	2008年
年底总人口	万人	32.12	26.26	25.83	25.87	25.95
生产总值	亿元	2.27	7.56	9.40	11.00	16.13
第一产业	亿元	0.76	1.66	2.01	2.73	4.19
第二产业	亿元	0.38	1.67	1.94	2.38	4.17
第三产业	亿元	1.13	4.24	5.45	5.89	7.77
# 工业增加值	亿元	0.25	1.54	1.79	2.14	3.73
人均生产总值	元	762	2880	3608	4253	6224
生产总值指数	上年=100	135.8	114.1	123.9	120.3	128.9
全社会固定资产投资	万元	6183	27658	15545	29259	111313
地方财政收入	万元	1084	1605	1374	1766	2786
地方财政支出	万元	6813	20606	23754	35352	50143
农村居民人均纯收入	元	764	1668	1828	2117	2946
城镇居民人均可支配收入	元				7956	11360
常用耕地面积	公顷	41887	29398	29400	29398	29689
粮食产量	吨	37093	66791	100611	71485	100246
农林牧渔业总产值	万元	12556	29607	35336	48404	66005
社会消费品零售总额	万元	12653	31785	39081	47350	65000
普通小学专任教师数	人	1680	1565	1518	1445	1406
普通小学在校学生数	人	48556	23946	21658	20415	19395
普通中学专任教师数	人	578	928	973	1036	1072
普通中学在校学生数	人	15472	20766	22258	21375	21025
卫生机构床位数	张	352	443	443	454	486
卫生技术人员	人	413	337	337	370	371
# 执业(助理)医师	人	132	241	240	213	214
注册护师、护士	人	45	73	73	70	60

?1994-2018 China Academic Journal Electronic Publishing House. All rights reserved.　http://www.cnki.net

社会主要指标

2009年	2010年	2011年	2012年	2013年	2014年	2015年	2016年
17.37	17.41	17.41	17.42	17.61	17.65	17.83	17.03
23.39	27.75	33.58	40.16	43.84	44.74	41.97	55.71
4.46	6.32	7.33	8.82	9.52	10.39	9.48	11.93
10.06	10.65	12.66	15.89	20.14	18.80	16.37	25.23
8.87	10.78	13.58	15.45	14.18	15.56	16.12	18.55
9.90	9.16	11.06	14.12	18.21	16.79	14.87	23.62
9005	10678	19282	23061	25028	25379	23539	31962
132.6	115.9	109.4	109.4	109.2	104.9	102.5	109.7
128960	171396	211498	260586	211601	211627	240952	374881
2508	3136	3858	4550	5551	8258	7945	12427
65607	101922	121154	158170	173704	176603	196627	235341
3693	4570	5621	6582	7394	8296	8322	9046
13644	16103	18921	21967	24406	26822	23598	25768
29689	29730	29735	29748	29789	30484	30440	30594
106713	117621	101167	110985	102361	105898	70489	101312
72010	98330	116523	140797	155906	168958	166563	196008
62520	64149	58678	57902	51136	56385	68188	76007
1508	1374	1321	1280	1225	958	1031	15379
18704	17799	16284	9354	8301	8397	8421	265420
1138	1140	1417	1226	1121	1075	790	17293
18825	16878	13247	7850	7662	6551	5919	165505
498	555	610	648	674	679	727	490
388	446	518	526	699	703	786	865
210	215	229	225	226	209	240	274
69	122	143	160	196	205	227	276

?1994-2018 China Academic Journal Electronic Publishing House. All rights reserved. http://www.cnki.net
